# Methodological considerations and cost to measure coverage of multisectoral nutrition interventions: protocol for the One Nutrition Coverage Survey in Bangladesh

**DOI:** 10.1136/bmjopen-2025-099314

**Published:** 2025-12-29

**Authors:** Swetha Manohar, Phuong Hong Nguyen, Sumanta Neupane, Melinda Kay Munos, Rebecca Heidkamp, Archis Banerjee, Niharika Pandya, Sunny S Kim

**Affiliations:** 1 Nutrition, Diets and Health, International Food Policy Research Institute, Washington, District of Columbia, USA; 2 Nutrition, Diets, and Health, International Food Policy Research Institute, Kathmandu, Nepal; 3 Department of International Health, Johns Hopkins University Bloomberg School of Public Health, Baltimore, Maryland, USA; 4 Nutrition, Diets, and Health, International Food Policy Research Institute, Dhaka, Bangladesh; 5 Nutrition, Diets and Health, International Food Policy Research Institute, New Delhi, India; 6 International Food Policy Research Institute Poverty Health and Nutrition Division, Washington, District of Columbia, USA

**Keywords:** PUBLIC HEALTH, Health, NUTRITION & DIETETICS

## Abstract

**Abstract:**

**Introduction:**

Despite global commitments to eliminate malnutrition, over half the world’s population remains affected. Multisectoral nutrition interventions targeting both proximate and distal causes of malnutrition are essential across the lifespan. Yet, current data collection lacks comprehensive nutrition intervention coverage measures, risking inaccuracies in tracking progress. The One Nutrition Coverage Survey (ONCS) aims to test new and refined coverage measurement methods, assess coverage equity and guide integration into large-scale household surveys.

**Methods and analysis:**

The ONCS will be a cross-sectional, population-representative household survey conducted in four districts of Bangladesh (Rangpur, Sylhet, Dhaka and Khulna), selected for their geographic spread and urban–rural balance. A stratified multistage sampling approach will be used to select enumeration areas, and a total of approximately 3280 households randomly selected within each EA will be included in the survey. The survey will interview women of reproductive age (15–49 years), caregivers of children (0–9 years), adolescents (10–19 years) and pregnant women, collecting data on multisectoral nutrition interventions relevant to these groups. It will use both existing and new measures, while also capturing monetary and non-monetary costs for survey design to implementation. Data will be analysed to assess coverage, co-coverage and equity by sociodemographic characteristics, as well as the feasibility, accuracy and costs of the survey approach.

**Ethics and dissemination:**

The study protocol and instruments were reviewed and approved by the International Centre for Diarrhoeal Disease Research, Bangladesh’s (icddr,b) Ethical Review Board in Bangladesh and the International Food Policy Research Institute’s Institutional Review Board in Washington, DC, USA. Adults provided signed informed consent and adolescents their assent. Findings will be shared through peer-reviewed publications, conferences and presentations in Bangladesh with key stakeholders. This study will yield new tools, methods and evidence for measuring multisectoral nutrition intervention coverage, applicable to other low-income and middle-income countries. Learnings from ONCS will enhance data collection aligned with national strategies, helping governments improve coverage assessments, inform decisions and strengthen programme monitoring.

**Strengths and limitations of this study**

- The One Nutrition Coverage Survey offers population-representative coverage estimates for a range of multisectoral nutrition interventions across the life course for four geographically diverse locations in Bangladesh.
- This methods survey captures comprehensive nutrition intervention coverage measures in single household survey, applies new coverage metrics (eg, for nutrition-sensitive social protection), and refines existing measures—such as those for maternal micronutrient supplementation during pregnancy—which have known validity issues.
- Given budgetary restrictions, there are sample size constraints; therefore, some indicators have a slightly lower degree of precision.
- Despite using a life stage approach to select key indicators, nutrition interventions targeted to older adults (<65 years) were not measured.

## Introduction

 Sustainable Development Goal 2 (SDG) aims to end hunger and malnutrition in all its forms by 2030. However, the triple burden of malnutrition—undernutrition, micronutrient deficiencies and obesity—continues to affect countries worldwide, regardless of income level.^1^ Food insecurity remains a significant challenge, with undernourishment affecting up to 9.4% of the global population (757 million people).^2^ Poor dietary quality contributes to a large proportion of global death and disability,^3 4^ with over 50% of preschool-aged children and non-pregnant women of reproductive age in low- and middle-income countries (LMICs) suffering from micronutrient deficiencies such as iron, zinc and vitamin A.^5^

Given the multifactorial, complex and interlinked determinants of malnutrition, governments in LMICs have developed multisectoral nutrition policies and strategies to address these issues,^6^ incorporating sectors such as health, agriculture, education and social protection.^7^,^9^ These interventions are often delivered through multiple service platforms and need to be monitored frequently beyond the facility level to address inequities in access, delivery and coverage.^10^ While progress has been made, gaps remain in measuring the effectiveness and reach of these nutrition interventions.^11 12^

A key challenge in assessing the impact of multisectoral nutrition programmes is the lack of comprehensive and accurate coverage data. Coverage refers to the proportion of a population in need of an intervention(s) who receive it. For nutrition, these populations typically include pregnant and lactating women,^13 14^ children during their first 1000 days, young children (2–5 years), and adolescents—groups with higher or special nutritional needs.^15 16^ There is evidence that school-age children also need to be supported by a range of nutrition interventions.^17 18^ Many nutrition interventions are delivered through the health system, and their coverage at the household level is often assessed through population-based surveys. However, these surveys are resource-intensive, covering a wide range of topics and placing a considerable burden on respondents, often women, who are asked to recall information on a variety of topics and household members.

Existing nationally representative household surveys, such as the Demographic and Health Surveys (DHS) and Multiple Indicator Cluster Surveys (MICS), provide some coverage data but are not designed to track the full spectrum of nutrition interventions across sectors, particularly those beyond the health sector. While these population-based household surveys are high quality and are often the most common and best source of coverage data on a range of topics,^19 20^ they can still be prone to measurement errors such as recall bias and inconsistencies in reporting, which can affect the reliability of coverage estimates. For example, questions on the adherence to daily oral iron and folic acid supplementation during pregnancy have been found to produce biased estimates due to recall limitations.^21^ Measurement challenges also arise for nutrition-sensitive interventions—such as large-scale food fortification (LSFF), nutrition-sensitive social protection (NSSP) and nutrition-sensitive agriculture (NSA)—which are often not included in these surveys. Furthermore, there is a lack of standardised coverage indicators for these interventions, making it difficult to track progress toward global targets like the SDGs, and the costs of data collection are not generally available. Finally, while 118 countries conduct one or both these surveys periodically, not all countries consistently carry out these surveys.^22^

The One Nutrition Coverage Survey (ONCS) aims to address these gaps by testing and documenting methods for measuring coverage of a comprehensive range of multisectoral nutrition interventions. The ONCS is a research study under the Data for Decisions in Nutrition (DataDENT),^23^ which is a project that aims to transform the availability and use of nutrition data by addressing gaps in nutrition measurement and advocating for stronger nutrition data systems. The goal of ONCS is to generate high-quality coverage data on key nutrition interventions across the life course, aligned with national nutrition and multisectoral strategies, and to share a set of survey tools and learnings on coverage measurement using a household survey. The methods-focused survey has four main objectives: (1) to pilot new and standardised coverage measurement questions that provide a comprehensive assessment of household coverage for multisectoral nutrition interventions; (2) to generate coverage and co-coverage estimates for these interventions and cross-validate them with other available data sources; (3) to assess equity in coverage of multisectoral nutrition interventions, highlighting analytical approaches that produce disaggregated estimates by gender, location, economic status, ethnicity, education and other contextually relevant social factors and (4) to develop guidance on the implementation and cost (both monetary and non-monetary) considerations for integrating these questions and modules into large-scale multitopic household surveys.

## Methods

### Key nutrition interventions across the life course

**Figure 1:**
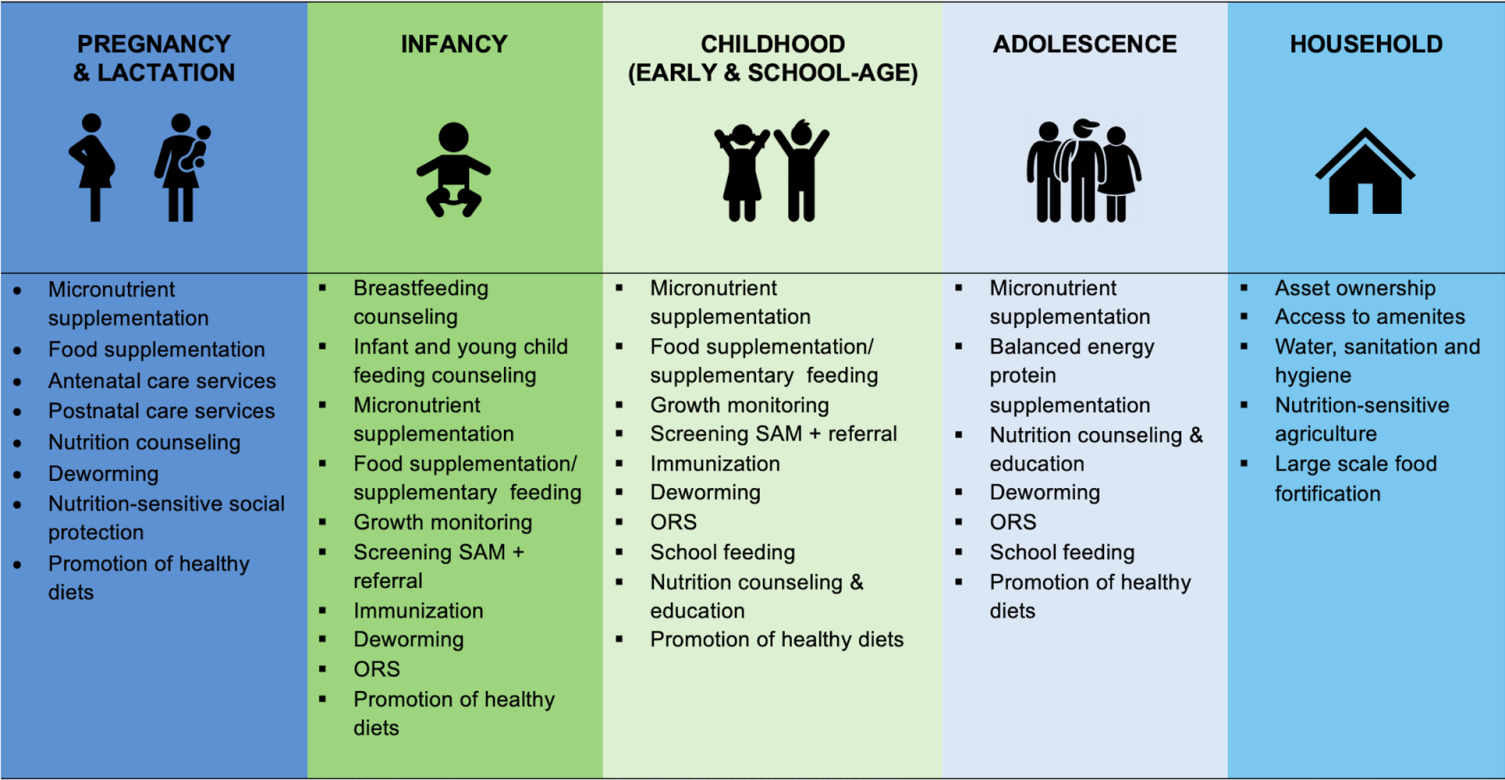
Key nutrition interventions across the life course. Interventions identified and informed by WHO's Essential Nutrition Actions Mainstreaming Nutrition through the Life Course, Every Woman Every Child: The Global Strategy for Women’s, Children’s and Adolescents’ Health 2016–2030, Lancet Series on Maternal and Child Nutrition 2021. ORS, oral rehydration supplement; SAM, severe acute malnutrition.

The ONCS is designed to capture the receipt of recommended nutrition interventions across the life course from pregnancy to adolescence, including both nutrition-specific and nutrition-sensitive interventions^8^ (figure 1). Figure 1 reflects the major globally recommended interventions, primarily the WHO’s Essential Nutrition Actions, at the household and life stage levels and the range of coverage data that the survey will collect.^9^ The WHO’s Essential Nutrition Actions are evidence-based interventions across the life course, focused on mothers and children to prevent mortality and ‘support physical and mental growth and development and improve productivity’.^9^ These interventions are primarily delivered through the health sector, but also include those implemented via other sectors such as agriculture, water, sanitation and hygiene (WASH), and social protection—that is they are multisectoral nutrition interventions.^9^ Multiple interventions may be co-located within a household and/or targeted to a nutritionally vulnerable household member such as a pregnant woman (co-coverage).^24^ ONCS focuses on interventions for the most nutritionally vulnerable household members—women of reproductive age (WRA), pregnant and lactating women, young children and adolescent boys and girls. The survey will capture the coverage of globally prioritised nutrition interventions implemented in the country by the government, development partners and other local entities.

### Landscaping nutrition policies and programmes

To begin with, a comprehensive landscaping of the current nutrition programmes and policies in Bangladesh was conducted. This exercise aimed to provide an overview of existing policies and programmes addressing food security and nutrition across multiple sectors, including health, agriculture, social protection and education (table 1). The landscaping exercise had two primary purposes: (1) to document existing national, regional and sub-national policies and implementation plans designed to address food security and malnutrition in all its forms in Bangladesh and (2) to identify ongoing household-level nutrition interventions aimed at improving food security and addressing malnutrition across life stages. The landscaping efforts also captured how some global recommendations have been adapted to the local context.

**Table 1:** Multisectoral nutrition interventions by target population, based on global and national nutrition policies and programmes

Intervention	Target population						
	Household	Pregnancy and lactation		Infancy	Childhood		Adolescent 10–19 years
		Pregnant women 15–49 years	Postnatal women 15–49 years	Child <2 years	Child 2–5 years	Child 5–9 years	
Agriculture, education, WASH, private food sectors							
Healthy diets							
NSA–home gardens*							
NSA–improved seed and nutrition counselling*							
NSA–livestock provision*							
NSA–hydroponic ponds*							
NSA–provision of seeds indigenous foods*							
Education on healthy diets*†							
Food fortification of staple foods							
Universal salt iodisation*†							
Vit A fortified oil*†	Indirect‡						
Vit A, B_1_, B_12_, Fe, FA, zinc in rice*†	Indirect‡						
Vit A and D of milk*†	Indirect‡						
Fe, FA and Vit B_12_ of wheat flour*†	Indirect‡						
Fe, FA and B_12_ in fortified wheat flour*†	Indirect‡						
Zinc, Vit A, B_1_, B_2_, B_3_ and B_6_ fortified wheat flour*†	Indirect‡						
Improved sanitation*							
Availability of drinking water*							
Handwashing facilities with water and soap*							
Health sector							
Umbilical cord clamping							
Optimal timing of umbilical cord clamping*†§							
Protecting, promoting and supporting breastfeeding							
Early BF support and counselling—facility and community*†							
Support for EBF and skin-to-skin contact*†							
Prevent breastmilk substitutes and formula distribution at hospitals*†							
Paid maternity leave*†							
Continued BF counselling*†							
Care of LBW infants							
Optimal feeding of LBW and VLBW*†							
Kangaroo care for LBW*†							
Assessment and management of wasting							
Identify infants <6 months with SAM*†							
Inpatient management of infants <6 months with SAM*†							
Outpatient management of infants <6 months with SAM and MAM*†							
Vit A supplementation*†				6–23 m¶	23–59 m¶		
Appropriate infant and child feeding							
Nutrition counselling on complementary feeding*†							
Growth monitoring assessment							
Weight and height/length assessment*†							
Nutrition counselling for children*†							
Preventative deworming–child*†							
Zinc during diarrhoea*†							
Oral rehydration salts during diarrhoea–child*†							
Immunisation*†							
Iron-containing micronutrient supplementation							
Provision of iron-containing MNP for point-of-use fortification of foods†							<12 years¶
Daily iron supplementation for infants and young children aged 6–23 months†							<12 years¶
Intermittent Fe and IFA supplementation for non-pregnant women†							
Fe and IFA supplementation for pregnant women*†							
Daily Fe supplementation for women*†							
Social protection, education sectors							
School feeding*†							
Nutrition-sensitive social protection–cash transfer*							
Nutrition-sensitive social protection–food/in-kind*							
Health sector							
Nutritional care during pregnancy and post partum							
Nutritional counselling on healthy diet to reduce the risk of low birth weight†							
Nutrition counselling–general*†				<6 months¶			
Energy and protein dietary supplements in undernourished populations†							
Vitamin A supplementation for pregnant women*†							
Calcium supplementation*†							
Assessment of blood pressure*†							
Blood glucose testing*†							
Weight monitoring*†							
Advice on weight monitoring*†							
Anaemia assessment*†							
Anaemia treatment (iron supplementation)*†							
Antenatal care screening by a trained provider*†							
Tetanus toxoid vaccination*†				<6 months¶			
Institutional delivery*†							
Presence of skilled birth attendant*†							
Postnatal care for women*†							
Postnatal care for babies*†							
Contraception*†							
Preventative deworming–women*†							

Grey-shaded cells indicate which target populations each intervention (listed on the left) applies to, and which policies include those interventions.

* Bangladesh policy recommendations based on policy review (list not exhaustive): Community Based Management of Acute Malnutrition in Bangladesh (programme), National Nutrition Policy, National Strategy for Infant and Young Child Feeding in Bangladesh, Essential Services Package (Urban), National Vitamin A Plus Campaigns (Programme), National Strategy for Infant and Young Child Feeding in Bangladesh (Policy), National Strategy for Anaemia Prevention and Control in Bangladesh, Multisectoral action plan for prevention and control of Non-communicable diseases 2018–2025, National Food and Nutrition Security Policy (NFNSP) 2020–2030, National Immunisation Policy, National Social Protection of Bangladesh Policy, National Strategy for Adolescent Health 2017–2030, Second Plan of Action for Nutrition 2017–2025, National Nutrition Services Operational Plan, Food Safety Act 2013.

† Global policy recommendation: WHO Health Organization Essential Nutrition Actions mainstreaming nutrition through the life course.

‡ ONCS will not directly measure whether a food is fortified but instead whether a household uses a fortifiable food vehicle at home.

§ This intervention will not be measured by ONCS given there is no standard coverage measure for household-level assessment, typically assessed at the facility-level.

¶ Age range the intervention pertains to.

BF, breastfeeding; EBF, exclusive BF; FA, folic acid; Fe, iron; IFA, iron and folic acid; LBW, low birth weight; MAM, moderate acute malnutrition; MNP, micronutrient powder; NSA, nutrition-sensitive agriculture; ONCS, One Nutrition Coverage Survey; SAM, severe acute malnutrition; Vit, vitamin; VLBW, very LBW; WASH, water, sanitation and hygiene.

The desk review on nutrition-specific policies, such as legislation, strategies and plans that directly address nutrition and food security.^25 26^ We also conducted a parallel search for nutrition-sensitive policies, which included policies in sectors like agriculture, health, education, social protection and poverty reduction (citations included above for landscaping desk review are not comprehensive; the review included the review of a more extensive set of policies and implementation documents).^27^,^29^ The review also included grey literature and programme documents from key development partners (eg, Food and Agriculture Organisation, World Food Programme, United Nations International Children’s Emergency Fund, Helen Keller International) to capture ongoing programmes and interventions by life stage. The results of this landscaping were verified with key in-country stakeholders from government and development sectors to address any gaps and ensure the comprehensive coverage of all relevant interventions.

### Survey design

The ONCS is a cross-sectional household survey, collecting comprehensive data on a range of multisectoral nutrition interventions to assess coverage across the life course. The survey will combine both existing and newly developed quantitative measures related to nutrition intervention coverage and co-coverage of multiple interventions, aligning with international standards and adapting to local context. The survey design was informed by input from a range of government and non-governmental stakeholders—specifically on sampling methodology and ongoing multisectoral nutrition policies and programme interventions. The survey is planned to begin in mid-March 2025 and end mid-June 2025.

### Survey setting

The ONCS will be conducted in Bangladesh, selected for its unique context of rapid economic development alongside persistent nutritional challenges. Despite significant progress in poverty reduction, agricultural production and food security, a third of children under five experience stunting,^30^ and micronutrient deficiencies such as anaemia and zinc deficiencies are widespread.^27 31^ The country also faces issues like early marriage and childbearing, contributing to intergenerational malnutrition,^30^ and coverage of key nutrition interventions is inconsistent.^31 32^ Although Bangladesh has a strong policy framework and substantial public investment in nutrition, implementation is hindered by overburdened health systems and sectoral coordination challenges. This complex landscape makes Bangladesh an ideal setting to examine multisectoral nutrition interventions and evaluate strategies for improving coverage and health and nutrition outcomes.

The survey is designed to be representative at the district level — Rangpur, Sylhet, Dhaka and Khulna (each district belonging to divisions with the same name, respectively). These districts are selected to ensure geographic diversity and balance between urban and rural areas, as well as the absence of recent large-scale disasters that could impact the results. The selected areas represent a variety of geographic, socioeconomic and nutritional contexts across Bangladesh. A map highlighting the four study districts is presented in online supplemental figure 1. The districts are further selected based on criteria such as the presence of active multi-sectoral nutrition programming, the proportion of women reporting ‘currently pregnant’ status, and a prevalence of childhood stunting greater than 20%, thus areas with varying levels of nutrition and food security challenges.

### Sampling methodology

**Figure 2:**
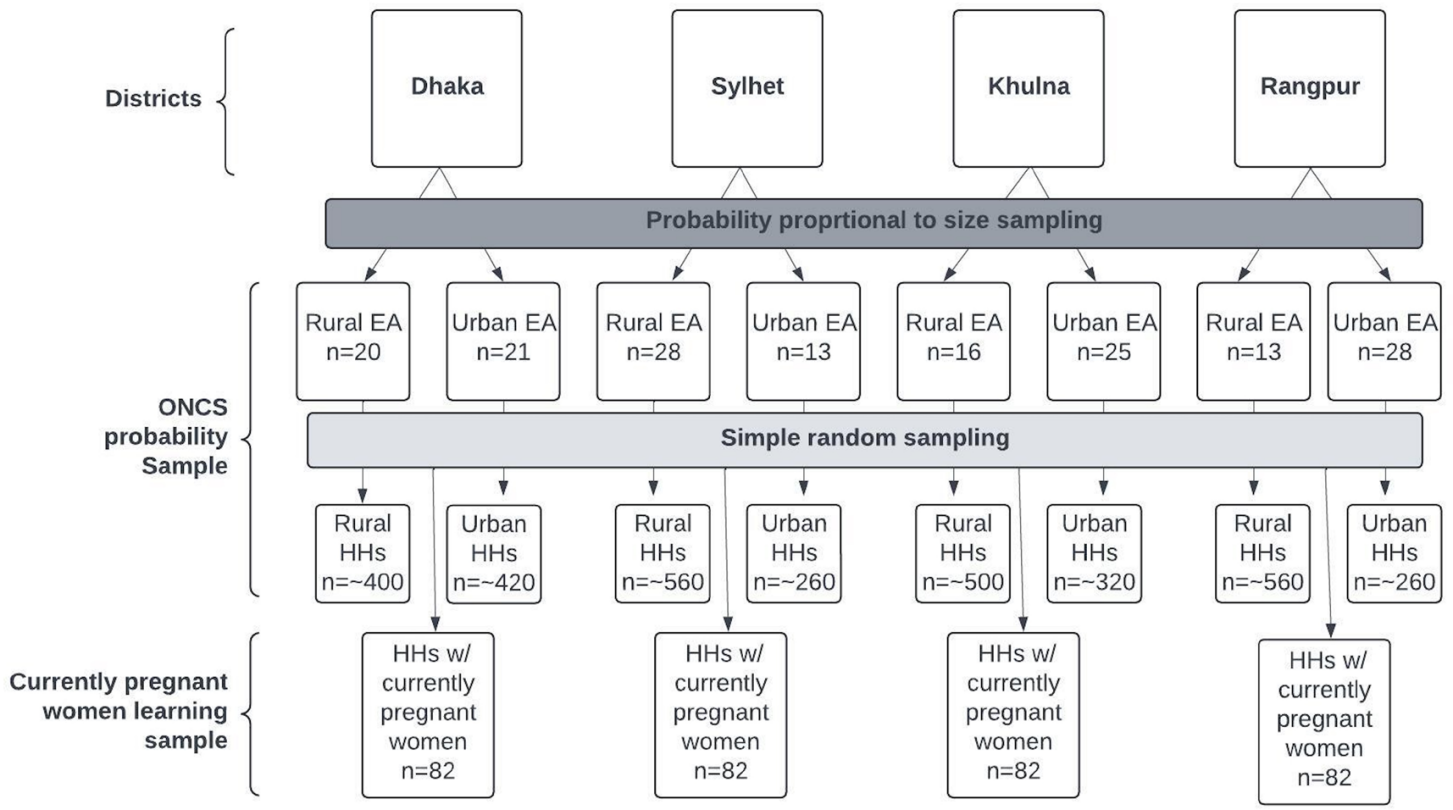
Sampling schema for the One Nutrition Coverage Survey (ONCS). EA, enumeration areas; HHs, households.

The survey will use a stratified multistage sampling approach to ensure that the sample is representative at both the district and urban–rural levels (figure 2). The sampling process and sample selection are coordinated closely with the Bangladesh Bureau of Statistics (BBS).

#### Enumeration area (upazila) selection

The first stage of sampling involves selecting enumeration areas (EAs), which are the primary sampling units (PSUs) of the survey. EAs will be drawn from the 2022 Bangladesh Census, using the selected districts stratified into their corresponding urban and rural sub-units as the sampling frame. 41 EAs will be proportionally allocated to urban and rural strata within each district to ensure that the sample represents both urban and rural areas, for a total of 164 EAs across the four districts. This stratification will allow for the calculation of urban and rural coverage estimates with adequate precision. Sampling procedures are coordinated with the BBS for the first stage of sampling, ie, the EA selection process. Following the selection of EAs, a household listing exercise will be conducted within each sampled EA to create a detailed map and list of households by the survey team. This step will involve engagement with local and community officials and leaders to ensure appropriate permissions are secured.

#### Household selection

In the second stage, households within each EA will be selected using simple random sampling. A total of 20 households per EA will be selected for the survey; the recruitment process will be carried out by the enumerator team led by the field supervisor. Once sampled, all households will be administered the household questionnaire (described below) to ascertain whether the survey’s target population is a household member. The full set of questionnaires (described below) will only be administered to households with at least one of the following member types: (1) women of reproductive age (WRA), 15–49 years; (2) children aged 0–9 years and (3) adolescents aged 10–19 years. The full set of questionnaires will not be administered to households if they lack WRA, children under 10 years, or adolescents. Additionally, households that do not provide consent to participate in the survey will be excluded, as will households located within refugee camps, as these settings are outside the scope of the survey.

#### Learning sample for currently pregnant women

In addition to the random sample of households, a learning sample of 328 currently pregnant women will be selected. This sample will be drawn from households that report having a pregnant woman during the household listing process. These additional women will form a target group for testing newly developed indicators related to nutrition interventions during pregnancy. These women will not be included in the coverage estimates, but their data will be used for methodological learning and testing analytical purposes.

### Sample size calculation

The sample size is calculated using a standard formula, accounting for a 5% margin of error, 10% non-response rate and a design effect of 1.5.


$$n=Z_{(\frac{\alpha }{2}{)}^{2}}\times \frac{pq}{d^{2}}\times deff\times \frac{1}{1-r}$$

where n=the required sample size, expressed as number of households

p=estimated proportion

q=1–p

2=desired precision

r=survey non-response rate

Z _(∝/2)_=critical value of normal distribution

∝=alpha or probability of committing a type 1 error

deff=design effect for the indicator.

The key indicators used to estimate the minimum sample size required to produce reasonable estimates for each district are receipt of vitamin A supplementation among 6–59 months children, receipt of 4+antenatal care (ANC) visits among women pregnant in the past 2 years and taking any iron supplement among women pregnant with a live birth in the past 2 years. Data for these indicators, survey non-response rate and design effect were obtained from BDHS 2017.^30^ The final sample for the ONCS will comprise 3280 households, with an additional 328 currently pregnant women selected as part of the learning sample, yielding a total sample size of 3608 households.

### Study participants

This survey focuses on key nutritionally vulnerable groups, prioritising specific life stages when multisectoral essential nutrition interventions are needed. These include WRA, including women who have been pregnant in the past 2 years (both women with live births and a subset of interventions pertaining to those with stillbirths), currently pregnant women, and non-pregnant women who are mothers of young children <10 years); infants and young children (0–59 months); school-age children (6–9 years) and adolescents (10–19 years).

Survey respondents include the head of the household, who will provide information on household characteristics, assets, food insecurity and exposure to food fortification. WRA will answer questions on nutrition interventions relevant to their maternal or pregnancy status. To capture coverage of maternal health indicators among married young women given the prevalence of child marriage among girls (10–14 years) in Bangladesh (~6%),^33^ married adolescents aged 10–14 years will also be administered the Women’s questionnaire. If a WRA is not the primary caregiver of a child under 10, the caregiver will respond on behalf of the child. Adolescent boys and girls aged 10–19 years will be asked about their participation in nutrition programmes such as micronutrient supplementation, deworming and school feeding. Adolescent girls aged 15–19 years will be administered both the Women’s questionnaire (relevant modules only) and the Adolescent questionnaire.

### Data collection

Data will be collected at both the household and individual levels using pretested electronic questionnaires on tablets, programmed with CAPI (Computer-Assisted Personal Interviewing) software. The survey instruments will include three key questionnaires: (1) Household questionnaire, (2) Women’s questionnaire and (3) Adolescent questionnaire (online supplemental material 1).

The survey questions were developed based on a combination of existing measures from routine population-based health and nutrition surveys (such as DHS, MICS and Bangladesh Integrated Household Survey (BIHS)), large-scale programme evaluations of nutrition interventions and newly developed or adapted indicators under the DataDENT project. The indicators are classified as follows: new and improved indicators (developed by the DataDENT project to pilot in ONCS),^34^,^36^ standard measures (used in DHS or MICS surveys) and adapted measures (modified from population-based surveys like DHS, MICS, BIHS or other programme evaluations and studies that measure intervention coverage at the household and individual levels) (table 2). The questionnaires and specific modules are designed based on the life stage and include coverage measures for globally recommended multisectoral nutrition interventions relevant for each life stage. Iterative cognitive testing of questions related to maternal micronutrient supplementation, NSSP and LSSF questions to improve face and content validity, but the study was not funded to conduct criterion validity studies.

**Table 2:** ONCS modules by respondent type and measurement status

Topic	Respondents					New/adapted/standard measure*
	Head of HH	Pregnant women	Women with children <2 years	Women of reproductive age	Adolescents	
HH identification	x					Standard^48^
HH composition and demographics	x					Standard^48^
Asset ownership	x					Standard^48^
Access to amenities and financial services	x					Standard^48^
HH Food Insecurity (Food Insecurity Experience Scale)	x					Standard^38^
Large scale food fortification (fortifiable food vehicle use)	x					Adapted^37^
Nutrition-sensitive agriculture	x					Adapted
Women’s demographics		x	x	x		Standard^48^
Barriers to healthcare		x	x	x		Standard^48^
Birth history		x	x	x		Adapted^48^
Interventions for WRA						
Iron folic acid supplementation				x		Adapted^36 48^
Deworming				x		Standard^48^
Contraception use				x		Standard^48^
Nutrition-sensitive social protection				x		New^34 49 50^
Diet Quality Questionnaire				x		Standard^40^
Interventions during current pregnancy						
Frequency and timing of ANC						Standard^48^
Services/messages received during ANC		x				Standard^48^
Iron folic acid supplementation		x				New^36^
Micronutrient nutrition counselling		x				Adapted^36^
Food supplements		x				New
Insecticide treated net		x				Standard^48^
Deworming		x				Standard^48^
Nutrition-sensitive social protection		x				New^34^
Diet Quality Questionnaire		x				Standard^40^
Interventions during pregnancy in past 2 years†						
Iron folic acid supplementation			x			Adapted^36 48^
Calcium supplementation			x			New^36 48^
Food supplements			x			Adapted^48^
Antenatal care			x			Standard^48^
Postnatal care			x			Standard^48^
Nutrition counselling			x			New
Deworming			x			Standard^48^
Nutrition support			x			Adapted
Nutrition sensitive social protection			x			New^34^
Diet Quality Questionnaire			x			Standard^40^
Interventions for children <5 years‡						
Breastfeeding and IYCF counselling		x	x	x		New^51^
Food supplementation		x	x	x		New
Growth monitoring		x	x	x		Adapted^1^
SAM community screening and referral		x	x	x		Adapted^48^
Immunisation		x	x	x		Standard^48^
Micronutrient supplementation		x	x	x		Standard^48^
Deworming		x	x	x		Standard^48^
ORS		x	x	x		Standard^48^
Diet Quality Questionnaire		x	x	x		Standard^40^
Interventions for children 5–9 years‡						
Micronutrient supplementation		x	x	x		Adapted^48^
Deworming		x	x	x		Standard^48^
ORS		x	x	x		Standard^48^
Diet Quality Questionnaire		x	x	x		Standard^40^
School feeding		x	x	x		New^34^
Interventions for adolescents						
Micronutrient supplementation					x	Adapted^48^
Deworming					x	Adapted^48^
ORS					x	Adapted^48^
Diet Quality Questionnaire					x	Standard^40^
School feeding					x	New^34^

Adapted: measures adapted from survey questions used in programme evaluations that measure impact of interventions or other population-based surveys. Certain measures are categorised as ‘adapted’ because the standard measures are being used in a target population for which the measure is not typically used.

New: measures that do not currently exist that are being developed or are existing measures that are being improved and are being piloted by the DataDENT project.

* Standard: existing measures used in population-based surveys, specifically the Demographic and Health Surveys and the Multiple Indicator Cluster Surveys.

† Administered to women with a live birth in the past 2 years.

‡ Modules administered conditioned on age of the children in sampled household and whether woman is mother/caretaker of reference child.

ANC, antenatal care; HH, household; IYCF, infant and young child feeding; ONCS, One Nutrition Coverage Survey; ORS, oral rehydration salts; WRA, women of reproductive age.

The Household questionnaire will include a roster of all household members and collect data on their demographics such as sex, age, education, religion, occupation, as well as household assets and access to basic amenities and services. It will also capture information on nutrition-sensitive interventions, including WASH, NSA and LSFF.^35 37^ Food insecurity will be measured using the Food Insecurity Experience Scale (FIES).^38^

The Women’s questionnaire includes modules for WRA, with questions for different life stages, such as for mothers of children under 10, pregnant women and those with recent pregnancies. All WRA will be asked about socio-demographic information, healthcare barriers and birth history, and dietary diversity using the Diet Quality Questionnaire (DQQ) which has been shown to have cross-cultural validity and was specifically designed to be a low-cost tool for use in population-based surveys for women, children and adolescents.^39^ For women who were pregnant in the last 2 years, questions will cover ANC frequency, timing and provider type; micronutrient supplementation during pregnancy^36^; delivery; postnatal care; blood tests for anaemia and diabetes; and breastfeeding support. New questions will inquire about nutrition counselling, receipt of NSSP programmes^34^ and food supplements during pregnancy. Additionally, modules for mothers with children under 5 years will include questions on counselling on child feeding, growth monitoring, immunisation, deworming and dietary diversity using an adapted Child Diet Diversity Questionnaire for Bangladesh.^40^ Currently, pregnant women will receive a specific set of questions on ANC service coverage, micronutrient supplementation and iron-folic acid use, with recall periods tailored to capture recent data.

The Adolescent questionnaire includes modules on interventions and dietary diversity using the DQQ. The intervention module includes questions on iron supplements received or purchased in the last 3 months, deworming tablets and food supplements received through school or other platforms in Bangladesh. It will also assess awareness of healthy eating practices, such as the importance of a diverse diet and avoiding unhealthy foods. Additionally, questions will cover participation in school feeding programmes in the past year, including whether the food was fortified, and if other services like nutrition education, health referrals or micronutrient supplements were provided.

### Data quality control and data management

To ensure high-quality data, the data collection process will be managed by trained field staff under continuous supervision. Prior to the training of the survey team, a master training of trainers and pretest period, where questionnaires will be field tested for clarity and relevance, will be completed. All instruments will be translated into Bangla and undergo back-translation to ensure linguistic accuracy. Data will be entered using electronic tablets programmed for CAPI. The CAPI system will include built-in checks, such as logic checks, valid values, skip patterns and range checks, to enhance data quality at the point of entry. Global Positioning System (GPS) tracking, interview duration and interview completion data will also be recorded within the software for monitoring purposes.

Field supervisors and quality control personnel will closely oversee the data collection, with regular oversight from the study’s investigators. Data quality checks will be conducted daily, with immediate feedback provided to enumerators to address errors such as outliers, missing data and data inconsistencies. Unannounced field visits will be made by field monitors to ensure adherence to protocols. Daily briefings and debriefings will allow the data collection teams to discuss challenges and ensure timely corrective actions. Supervisors will liaise regularly with field monitors and the field coordinator, while the field coordinator will maintain frequent communication with the study investigators and research assistants to ensure continuous support and guidance throughout the data collection process.

A secure data file structure will be established on a protected server for use by the research team. All data will be anonymised and de-identified to ensure the privacy of participants. Identification codes will replace any personal identifiers, and no names, GPS coordinates, dates of birth or other identifying information will be stored in the databases. Changes to the raw data output files from the CAPI software will be fully documented. This includes detailed records of all formatting, reshaping and labelling of the data, which will be tracked using well-annotated do-files to ensure transparency and reproducibility of the data processing steps.

### Costing data

Cost data will include monetary and non-monetary costs (table 3). Monetary costs will be collected through itemised tracking of the survey budget and expenses across different line items. Non-monetary costs include the perceived level of effort required to design and implement the survey, the time burden to the respondent and the respondent fatigue. The perceived level of effort will be assessed by a team of survey personnel, including the investigators, in-country partners and data collectors. The level of effort required for survey design and implementation will be scored using either a 0–5 or 1–5 scale for each questionnaire module across several dimensions, including module customisation (0–5), survey length (0–5), topic relevance (0–5), logistics (0–5), design or eligibility changes (0–5), sample size adjustments (0–5), training (1–5), supervision (0–5), data processing and analysis efforts (0–5) and burden on respondent (1-5). To assess the time burden to the respondents, we will programme survey CAPI to capture this automatically by each questionnaire module.

**Table 3:** Summary of data source and type of analysis for monetary and non-monetary costs

	Data source	Analysis
Monetary cost		
Cost by various study phases (design, preparatory, survey, postsurvey, dissemination)	Budget template (salaries, per-diems, logistics, travel, equipment, supplies, consulting fees for each study phase)	% share of phases Per unit (sample) cost Per minute cost
Non-monetary cost		
Perceived level of effort for each questionnaire module	Scoring* of questionnaire module by key stakeholders involved in the study	Total level of effort score for each questionnaire module, calculated by summing the ratings
Time burden to the respondent	Collected during the interview with the respondent (automated in CAPI)	Average time per questionnaire module Association between survey duration and number of reported interventions received by women
Respondent fatigue	Respondents’ perception of the difficulty in answering the questions and trying to participate in the survey, measured on a Likert scale	% of respondents reporting too difficult or very tiring Correlation of rating with total ‘don’t know’ responses

* Scoring will be based on (1) Challenging to customise (0–5); (2) Length (0–5); (3) Exogenous topic (0–5); (4) Extra logistics (0–5); (5) Changes in survey design/eligibility (0–5); (6) Increases sample size (0–5); (7) Burden on training (1–5); (8) Burden on supervision (0–5); (9) Burden on data processing and analysis (0–5) and (10) Burden on respondent (1–5).

CAPI, Computer-Assisted Personal Interview.

Finally, respondent fatigue will be assessed using an image-based Likert scale administered at the end of the survey for two questions, how tiring it was to participate in the survey and how difficult it was to answer the questions.

### Data analysis

Descriptive statistics will be generated for a list of key coverage indicators (see detailed definitions, specifying numerators, denominators and recall periods online supplemental table 1), disaggregated by district, urban–rural location and sociodemographic characteristics. Standard indicators, specifically DHS indicators related to maternal health and nutrition interventions, will be computed using standard conventions.^41^ Pilot indicators will also be computed to inform learning on coverage measurement of multisectoral nutrition interventions targeted across the life cycle that are not typically measured but captured in country policies (eg, NSSP or iron supplementation among adolescents). All analysis will adjust for the design effect given the study design and sampling weights applied. In addition to the coverage of individual interventions, we will assess co-coverage—defined as the clustering of multiple interventions at the individual or household level. Co-coverage will be calculated using the count of the interventions for which an individual is eligible and which they received.^42^ Key constructs for co-coverage analysis will include essential nutrition services by life stage (ie, adolescence, pregnancy, lactation and early childhood), as well as targeted public health programmes or initiatives such as anaemia control programme. Multisectoral integration at the household level will also be considered, incorporating indicators of health and nutrition interventions, WASH, LSFF and NSA. For co-coverage analysis, we will apply different estimation methods including means of simple counts of interventions received based on a life stage or public health programme or proportions that achieved at least one intervention, all interventions, or 50% of interventions within a specified period. Additionally, the analysis will assess the validity, feasibility and accuracy of the survey measures, including non-response rates.

For the costing analysis, using the monetary cost data, the percentage share of cost for each survey phase (preparation, training, field data collection, data management, etc) will be calculated, as well as the percentage share of cost per respondent^43^ cost and cost per minute.^44^ Monetary costs include salary, per diem, logistics, travel, equipment and consulting fee costs. Using the non-monetary perceived level of effort data, a standardised score of level of effort will be calculated for each of the dimensions described and each questionnaire module. This will offer information on which dimension of the survey planning and implementation as well as which questionnaire modules require more of an effort to undertake (table 3).

To estimate the time burden related to the survey, first the average time to conduct the household listing per district will be calculated as well as the average time to complete the survey (questionnaires) per respondent and per household. The survey duration data will be used in one additional novel way. Previous literature has shown that survey duration is negatively associated with the number of benefits reported by households, including various transfers received.^45^ By applying a similar approach, an examination of whether the number of reported nutrition interventions received by women at different life stages is negatively associated with time for survey completion will be examined. Our survey is designed to administer the same modules to a woman multiple times, depending on the number of live births reported. For instance, if a woman has had two births in the past 2 years and an additional one in the past 5 years, the modules related to antenatal care will be administered twice. The modules related to children under 5 years of age will also be administered. Non-response bias may result given the repetitive nature of the questions a woman with multiple young children would have to answer, particularly by the second round and if she is overwhelmed by the survey. This variation in non-response will be explored and the association between survey duration and the number of reported interventions received examined based on the target population in a household. A limitation of this analysis is possibly a limited sample size if there are few women who report having multiple births and/or multiple children.

All data management, cleaning and statistical analyses will be conducted using Stata V.18 (StataCorp).

### Ethical considerations and dissemination

This study has obtained ethical clearance from the Institutional Review Boards of the International Food Policy Research Institute (NDH-24-0931) and from the International Centre for Diarrhoeal Disease Research, Bangladesh (icddr,b) Ethical Review Board (PR-24154) in Bangladesh. Written informed consent will be obtained from all participants, with parental consent and adolescent assent required for minors. Participation is voluntary, and respondents may withdraw at any time without consequence. Consent forms will be translated into Bangla and read aloud to participants, with time allowed for questions before the interview.

Findings from the survey will be disseminated in-country with relevant government and non-government stakeholders and globally to prompt careful consideration of not only the coverage data itself but how coverage data is collected (to improve validity of estimates), analysed and can inform multisectoral nutrition policy and programming. Collaborating investigators will disseminate results among local district authorities through meetings of stakeholders. Local partners will be encouraged to use the data within Bangladesh to raise awareness of the need for high-quality nutrition coverage data and its use. The results of the study will be published in scientific journals.

## Discussion

Coverage data are imperative to track global progress and assess the reach and effectiveness of policies and programmes and offer fundamental information on who is receiving what intervention(s) and who is not. Surveys that collect coverage data on a full range of multi-sectoral nutrition interventions do not currently exist,^46^ and important considerations must be made to ensure that high-quality data is collected through proper survey design and implementation such that they can offer meaningful and actionable information.^47^ As a methods survey, ONCS allows a learning framework to test the design and implementation of comprehensive coverage data collection using a household survey, generate evidence for improved measures and offer transparent information about costs/burden. Design elements include the inclusion of appropriate coverage indicators mapped to a country’s policy landscape, measures adapted for context, probability sampling design, sample size estimations when considering coverage of a broad range of interventions, well-designed and accurately translated questionnaires, and rigorous data management systems.

The ONCS will provide high-quality data on the coverage of nutrition interventions, aligned with national nutrition/multisectoral strategies, and will explore the feasibility of new measures and indicators, particularly for interventions without standardised metrics. A complete documentation of the ONCS design, questionnaires and implementation with cost, measurement and analysis considerations will be published serving as a template for implementation for governments, researchers and development partners interested in conducting population-based coverage assessments of multisectoral nutrition interventions.

A limitation to this study includes budgetary constraints, which limit the sampling frame and sample size of the survey. The sample size estimation was based on estimates for households with recently pregnant WRA; thus, we may not have power to estimate coverage of interventions targeted to other specific life stages (eg, adolescents). Additionally, we anticipate analytical challenges in estimating co-coverage of certain interventions where the denominators vary (eg, curative interventions such as oral rehydration solutions during diarrhoeal illness). For certain interventions (eg, wasting prevention), there is no global consensus on the intervention itself, so coverage measures in those cases have not been included in the ONCS. The ONCS methodology will allow for estimates of key nutrition intervention coverage indicators with sufficient precision, ensuring that its findings can be generalised to the larger population.

Application of the ONCS in Bangladesh, a country with a robust nutrition policy landscape and ongoing multisectoral nutrition interventions, will produce new tools, methods and evidence for multisectoral nutrition intervention coverage measurement. The results from this methods-focused survey will inform similar efforts in other LMICs and contribute to the global goal of improving nutrition and achieving the SDGs.

## Supplementary material

10.1136/bmjopen-2025-099314 online supplemental file 1
