# Supplementary material for: Methodological considerations and cost to measure coverage of multisectoral nutrition interventions: protocol for the One Nutrition Coverage Survey in Bangladesh
Source: BMJ Open. 2025 Dec 29;15(12):e099314. doi: 10.1136/bmjopen-2025-099314 (PMC12750752; doi:10.1136/bmjopen-2025-099314)
Supplement: online supplemental file 1 [file bmjopen-15-12-s001.docx]

**Supplemental Table 1: One Nutrition Coverage Survey indicators list**

| **Household Indicators** | **Definition/Indicator** | **Numerator** | **Denominator** |
| --- | --- | --- | --- |
| **Handwashing facility with water and soap** | % de jure household members with a fixed place on HH premises for hand washing where water and soap or detergent is present | Number of household members with a fixed place for hand washing where water and soap or detergent is present | Total number of household members |
| **Handwashing facility with water and soap  (limited)** | % de jure household members with a fixed place on HH premises for hand washing but no water and soap or detergent is present | Number of household members with a fixed place for hand washing where water and soap or detergent is present | Total number of household members |
| **Handwashing facility with water and soap  (no facility)** | % de jure household members with no fixed place for hand washing on HH premises | Number of household members with a fixed place for hand washing where water and soap or detergent is present | Total number of household members |
| **Use of improved drinking water sources** | % de jure household members using improved sources of drinking water | Number of household members using improved sources of drinking water | Total number of household members |
| **Use of unimproved drinking water sources** | % de jure household members using unimproved sources of drinking water | Number of household members using improved sources of drinking water | Total number of household members |
| **Use of drinking water service (basic)** | % de jure household members using improved sources of drinking water located within 30 minutes of the household | Number of household members using improved sources of drinking water either in their dwelling/yard/plot or within 30 minutes round trip collection time | Total number of household members |
| **Availability of drinking water** | % household members with a water source that is available when needed | Number of household members with a water source that is available when needed | Total number of household members |
| **Use of improved sanitation facilities** | % household members using improved sanitation facilities | Number of household members using improved sanitation facilities | Total number of household members |
| **Use of improved sanitation facilities (basic)** | % household members using improved sanitation facilities which are not shared | Number of household members using improved sanitation facilities which are not shared | Total number of household members |
| **Crop-growing households** | % of crop-growing households | Number of crop-growing households | Total number of households interviewed |
| **Improved seed among crop-growing households** | % of crop-growing households that received improved crop seeds | Number of households that received improved crop seeds | Total number of households that grow crops |
| **NSA Coverage - Improved seed + nutrition/health intervention** | % of crop-growing households that received improved crop seeds and a health/nutrition intervention | Number of households that received improved crop seeds and nutrition counseling or training | Total number of households that grow crops |
| **Fruit-growing households** | % of fruit-growing households | Number of fruit-growing households | Total number of households interviewed |
| **Fruit sapling among fruit-growing households** | % of fruit-growing households that received fruit saplings | Number of households that received fruit saplings | Total number of households that grow fruits |
| **NSA coverage – fruit sapling + nutrition/health intervention** | % of fruit-growing households that received fruit saplings and a health/nutrition intervention | Number of households that received fruit saplings and nutrition counseling or training | Total number of households that grow fruits |
| **Vegetable-growing households** | % of vegetable-growing households | Number of vegetable-growing households | Total number of households interviewed |
| **Veg seeds among vegetable-growing households** | % of vegetable-growing households that received vegetable seeds | Number of households that received vegetable seeds | Total number of households that grow vegetables |
| **NSA coverage – veg seeds + nutrition/health intervention** | % of vegetable-growing households that received vegetable seeds and a health/nutrition intervention | Number of households that received vegetable seeds and nutrition counseling or training | Total number of households that grow vegetables |
| **Aquatic-farming households** | % of households involved in fish/shrimp farming | Number of households involved in fish/shrimp farming | Total number of households interviewed |
| **Aquatic stock among aquatic-farming households** | % of aquaculture households that received fish/shrimp stock | Number of households that received fish/shrimp stock | Total number of households that raise small aquatic animals |
| **NSA coverage – aquatic stock + nutrition/health intervention** | % of aquaculture households that received stock and a health/nutrition intervention | Number of households that received fish/shrimp stock and nutrition counseling or training | Total number of households that raise small aquatic animals |
| **Poultry-raising households** | % of households involved in poultry rearing | Number of households involved in raising poultry | Total number of households interviewed |
| **Poultry stock among poultry-raising households** | % of poultry households that received poultry stock | Number of households that received poultry stock | Total number of households that raise poultry |
| **NSA coverage – poultry stock + nutrition/health intervention** | % of poultry households that received poultry stock and a health/nutrition intervention | Number of households that received poultry stock and nutrition counseling or training | Total number of households that raise poultry |
| **Goats/sheep-raising households** | % of households involved in raising goats/sheep | Number of households involved in raising goats/sheep | Total number of households interviewed |
| **Goats/sheep stock among goats/sheep-raising households** | % of goats/sheep households that received goats/sheep stock | Number of households that received goats/sheep or cattle stock | Total number of households that raise goats/sheep |
| **NSA coverage – goats/sheep stock + nutrition/health intervention** | % of goats/sheep households that received stock and a health/nutrition intervention | Number of households that received goats/sheep stock and nutrition counseling or training | Total number of households that raise goats/sheep |
| **Cattle-raising households** | % of households involved in raising cattle | Number of households involved in raising cattle | Total number of households interviewed |
| **Cattle stock among cattle-raising households** | % of cattle households that received cattle stock | Number of households that received cattle stock | Total number of households that raise cattle |
| **NSA coverage – cattle stock + nutrition/health intervention** | % of cattle households that received cattle stock and a health/nutrition intervention | Number of households that received cattle stock and nutrition counseling or training | Total number of households that raise cattle |
| **Home-garden households** | % of households with a home garden | Number of households with a home garden | Total number of households interviewed |
| **Home-garden support among home-garden households** | % of home-garden households that received gardening support | Number of households that received home-garden support | Total number of households that have a home garden |
| **NSA coverage – home-garden support + nutrition/health intervention** | % of home-garden households that received support and a health/nutrition intervention | Number of households that received home-garden support and nutrition counseling or training | Total number of households that have a home garden |
| **Agricultural or livestock raising households** | % of households that grow crops/fruits/veg or raise animals or have a home garden | Number of households that grow crops/fruits/veg or raise animals or have a home garden | Total number of households interviewed |
| **NSA coverage – any agricultural input receipt** | % of households that received any agriculture/livestock inputs | Number of households receiving any agricultural or livestock inputs | Total number of households that grow crops/fruits/veg or raise animals or have a home garden |
| **NSA coverage – any intervention + nutrition/health** | % of households that received any agriculture/livestock inputs and a health/nutrition intervention | Number of households receiving any agricultural or livestock inputs and nutrition counseling or training | Total number of households that grow crops/fruits/veg or raise animals or have a home garden |
| **Ag extension coverage** | % of households meeting an agricultural extension/NGO worker at least once per week | Number of households that met an extension/NGO worker at least once a week | Total number of households interviewed |
| **Nutritionally vulnerable HHs receiving cash +nutr/health intervention - poorest HHs** | % of households with a nutritionally vulnerable HH member that received cash transfer + receipt of nutr/health action | Number of households with a nutritionally vulnerable HH member that received cash transfer + receipt of nutr/health action | Total number of households interviewed with a nutritionally vulnerable HH member |
| **Poorest nutritionally vulnerable HHs receiving cash +nutr/health intervention - poorest HHs** | % of households with a nutritionally vulnerable HH member in lowest wealth quintile that received food transfer + receipt of nutr/health action | Number of households with a nutritionally vulnerable HH member in bottom wealth quintile that received food transfer + receipt of nutr/health action | Total households with a nutritionally vulnerable HH member in lowest wealth quintile |
| **Nutritionally vulnerable HHs receiving food +nutr/health intervention - poorest HHs** | % of households with a nutritionally vulnerable HH member that received food transfer + receipt of nutr/health action | Number of households with a nutritionally vulnerable HH member that received food transfer + receipt of nutr/health action | Total number with a nutritionally vulnerable HH member of households interviewed |
| **Poorest nutritionally vulnerable HHs receiving food +nutr/health intervention - poorest HHs** | % of households with a nutritionally vulnerable HH member in bottom wealth quintile that received food transfer + receipt of nutr/health action | Number of households with a nutritionally vulnerable HH member in bottom wealth quintile that received food transfer + receipt of nutr/health action | Total households with a nutritionally vulnerable HH member in lowest wealth quintiles |
| **Nutritionally vulnerable HHs receiving in-kind transfer +nutr/health intervention - poorest HHs** | % of households with a nutritionally vulnerable HH member that received in-kind transfer + receipt of nutr/health action | Number of households with a nutritionally vulnerable HH member that received in-kind transfer + receipt of nutr/health action | Total number of households with a nutritionally vulnerable HH member interviewed |
| **Poorest nutritionally vulnerable HHs receiving in-kind transfer +nutr/health intervention - poorest HHs** | % of households with a nutritionally vulnerable HH member in bottom wealth quintile that received in-kind transfer + receipt of nutr/health action | Number of household with a nutritionally vulnerable HH member s in bottom wealth quintile that received in-kind transfer + receipt of nutr/health action | Total households with a nutritionally vulnerable HH member in lowest wealth quintile |
| **HHs receiving school meal+nutr/health intervention - all HHs** | % of households with school-aged children that received school meal transfer + receipt of nutr/health action | Number of households that received school meal transfer + receipt of nutr/health action | Total number of households interviewed with school-aged children (5-19 years) |
| **HHs receiving school meal +nutr/health intervention - poorest HHs** | % of households in bottom wealth quintile that received school meal transfer + receipt of nutr/health action | Number of households in bottom wealth quintile that received school meal transfer + receipt of nutr/health action | Total households in lowest wealth quintiles |
| **Use of food vehicle** | % of HHs that use the food vehicle at home | Number of households that consume a fortifiable food vehicle | Number of households interviewed |
| **Use of fortifiable food vehicle** | % of HHs that purchased or received (from SNP) a fortifiable food vehicle | Number of households that got a food vehicle from a source that sells fortifiable food vehicles | Number of households interviewed |
| **Coverage of food vehicle labelled as fortified – based on observation of package** | % of HHs that used fortified FV (based on observed packaging) | Number of households whose last-obtained food vehicle was in its original manufacturer packaging and on which a fortification logo and/or statement was visibly present. | Number of households interviewed; number HHs with FV available |
| **Coverage of iodized salt** | % of households with iodized salt (defined as salt containing 15-40 parts per million of iodine) available at home | Number of households with salt tested that has 15+ ppm | Number of households interviewed |
| **Maternal Indicators**  ***Current pregnancy*** | **Definition/Indicator** | **Numerator** | **Denominator** |
| **Supplementation - Any iron supplementation** | % of non-pregnant women (aged 15-49 years) who received or bought any supplements that contain iron in the past three months preceding the survey | Number of non-pregnant women (aged 15-49 years) who received or bought any supplements that contain iron in the past three months preceding the survey | Total number of non-non-pregnant women (aged 15-49 years) |
| **Supplementation type-supplements with multiple micronutrients** | % of non-pregnant women (aged 15-49 years) who received or bought supplements with multiple micronutrients in the past three months preceding the survey | Number of non-pregnant women (aged 15-49 years) who received or bought iron tablet in the past three months preceding the survey | Total number of non-non-pregnant women (aged 15-49 years) |
| **Supplementation - Iron tablet/ iron folic acid** | % of non-pregnant women (aged 15-49 years) who received or bought iron tablet/iron folic acid in the past three months preceding the survey | Number of non-pregnant women (aged 15-49 years) who received or bought iron tablet/iron folic acid in the past three months preceding the survey | Total number of non-non-pregnant women (aged 15-49 years) |
| **Deworming** | % of non-pregnant women (aged 15-49 years) who received deworming tablets in the past six months preceding the survey | Number of non-pregnant women (aged 15-49 years) who received deworming tablets in the past six months | Total number of non-pregnant women (aged 15-49 years) |
| **Contraceptive use** | % of non-pregnant women (aged 15-49 years) who use a modern contraceptive | Number of non-pregnant women (aged 15-49 years) who are currently using a modern contraceptive | Total number of non-pregnant women (aged 15-49 years) |
| **Antenatal care- At least one (currently pregnant)** | % of currently pregnant women (aged 15–49 years) who received ANC at least once during their current pregnancy by any provider. | Number of currently pregnant women (aged 15–49 years) who received ANC least once during their current pregnancy by any provider. | Total number of currently pregnant women (aged 15-49 years) |
| **Antenatal care- >1 ANC**  **(currently pregnant)** | % of currently pregnant women (aged 15–49 years) who had >1 ANC visit during their current pregnancy by any provider. | Number of currently pregnant women (aged 15–49 years) who had >1 ANC visit during their current pregnancy by any provider. | Total number of currently pregnant women (aged 15-49 years) |
| **Antenatal care- Early ANC (currently pregnant)** | % of currently pregnant women (aged 15–49 years) who attended ANC early (in 1st trimester) during current pregnancy | Number of currently pregnant women (aged 15–49 years) who attended ANC early in 1st trimester) during current pregnancy | Total number of currently pregnant women (aged 15-49 years) |
| **Supplementation - Any iron-containing supplementation**  **(currently pregnant)** | % of currently pregnant women (aged 15-49 years) who received or bought any tablet or syrup that contains iron | Number of currently pregnant women (aged 15-49 years) who received or bought any tablet (iron tablet/folic acid, MMS, FullCare) or syrup that contains iron | Number of currently pregnant women aged 15-49 years) |
| **Supplementation – multiple micronutrient supplement**  **(currently pregnant)** | % of currently pregnant women (aged 15-49 years) who received or bought a multiple micronutrient supplement during this pregnancy | Number of currently pregnant women (aged 15-49 years) who received or bought MMS tablet/ FullCare, during their current pregnancy | Total number of currently pregnant women (aged 15-49 years) |
| **Iron supplementation - Iron tablet/Iron folic acid**  **(currently pregnant)** | % of currently pregnant women (aged 15-49 years) who received or bought iron tablet/iron folic acid during this pregnancy | Number of currently pregnant women (aged 15-49 years) who received or bought Iron tablet/Iron folic acid during their current pregnancy | Total number of currently pregnant women (aged 15-49 years) |
| **Iron supplementation - timing of initiation**  **(currently pregnant)** | % of currently pregnant women (aged 15-49 years) who received or bought iron- containing supplements during [1st Trimester] | Number of currently pregnant women (aged 15-49 years) started to consume iron-containing supplements in [1st Trimester] | Total number of currently pregnant women (aged 15-49 years) |
| **Iron supplementation - adherence in previous month**  **(currently pregnant)** | % of currently pregnant women (aged 15-49 years) who consumed any iron containing supplements for at least X days during their current pregnancy | Number of currently pregnant women (aged 15 to 49 years) who consumed iron-containing supplements for at least X days in previous month | Total number of currently pregnant women (aged 15-49 years) |
| **Iron supplementation -typical adherence per month**  **(currently pregnant)** | % of currently pregnant women (aged 15-49 years) who reported typical consumption of iron containing supplements for at least following days per month during their current pregnancy  i. 1-9 days   ii. 10-19 days  iii. 20-30 days | % of currently pregnant women (aged 15-49 years) who reported typical consumption of iron containing supplements for at least following days per month during their current pregnancy  i. 1-9 days   ii. 10-19 days  iii. 20-30 days | Total number of currently pregnant women (aged 15-49 years) |
| **Counseling- taking iron supplements**  **(currently pregnant)** | % of currently pregnant women (aged 15 to 49 years) who were counseled on consuming iron supplements and its benefits and side effects during their current pregnancy by any provider | Number of currently pregnant women (aged 15 to 49 years) who were counseled on consuming iron supplements and its benefits and side effects during their current pregnancy by any provider | Total number of currently pregnant women (aged 15-49 years) |
| **Calcium supplementation - any Calcium**  **(currently pregnant)** | % of currently pregnant women (aged 15-49 years) who received or bought any tablets or syrup that contained calcium during their current pregnancy | Number of currently pregnant women (aged 15-49 years) who received or bought any tablets or syrup that contained calcium during their current pregnancy | Total number of currently pregnant women (aged 15-49 years) |
| **Calcium supplementation - timing of initiation**  **(currently pregnant)** | % of currently pregnant women (aged 15-49 years) started to consume calcium supplements in 1st trimester during their current pregnancy | Number of currently pregnant women (aged 15-49 years) started to consume calcium supplements in 1st trimester | Total number of currently pregnant women (aged 15-49 years) |
| **Calcium supplementation - adherence in previous month**  **(currently pregnant)** | % of currently pregnant women (aged 15-49 years) who reported typical consumption of calcium containing supplements for at least following days per month during their current pregnancy  i. 1-9 days   ii. 10-19 days  iii. 20-30 days | Number of currently pregnant women (aged 15 to 49 years) who consumed calcium supplements for at least X days in previous month | Total number of currently pregnant women (aged 15-49 years) |
| **Counseling- taking calcium supplements**  **(currently pregnant)** | % of currently pregnant women (aged 15 to 49 years) who were counseled on consuming calcium supplements and its benefits and side effects during their current pregnancy by any provider | Number of currently pregnant women (aged 15 to 49 years) who were counseled on consuming calcium supplements and its benefits and side effects during their current pregnancy by any provider | Total number of currently pregnant women (aged 15-49 years) |
| **Vitamin A**  **(currently pregnant)** | % of currently pregnant women (aged 15-49 years) who received or bought any vitamin A capsule during their current pregnancy | Number of currently pregnant women (aged 15-49 years) who received or bought any vitamin A capsule during their current pregnancy | Total number of currently pregnant women (aged 15-49 years) |
| **Deworming**  **(currently pregnant)** | % of currently pregnant women (aged 15-49 years) who received any deworming tablets during their current pregnancy | Number of currently pregnant women (aged 15-49 years) who received any deworming tablets during their current pregnancy | Total number of currently pregnant women (aged 15-49 years) |
| **Tetanus toxoid**  **(currently pregnant)** | % of currently pregnant women (aged 15-49 years) who received 2 tetanus shots during their current pregnancy | Number of currently pregnant women (aged 15-49 years) who received 2 tetanus shots during their current pregnancy | Total number of currently pregnant women (aged 15-49 years) |
| **Antenatal care- Hypertension screening**  **(currently pregnant)** | % of currently pregnant women (aged 15–49 years) who had their blood pressure measured during their current pregnancy. | Number of currently pregnant women (aged 15–49) who had their blood pressure measured during their current pregnancy. | Total number of currently pregnant women (aged 15-49 years) |
| **Antenatal care- Hypertension screening**  **(currently pregnant)** | % of currently pregnant women (aged 15–49) who had their blood glucose level tested during their current pregnancy. | Number of currently pregnant women (aged 15–49) who had their blood glucose level tested during their current pregnancy. | Total number of currently pregnant women (aged 15-49 years) |
| **Antenatal care- Hypertension screening**  **(currently pregnant)** | % of currently pregnant women (aged 15–49) who had their hemoglobin level tested during their current pregnancy. | Number of currently pregnant women (aged 15–49) who had their hemoglobin level tested during their current pregnancy. | Total number of currently pregnant women (aged 15-49 years) |
| **Anemia screening**  **(currently pregnant)** | % of currently pregnant women (aged 15–49 years) identified as anemic during their current pregnancy | Number of currently pregnant women (aged 15–49) identified as anemic during their current pregnancy | Total number of currently pregnant women (aged 15-49 years) |
| **Anemia treatment**  **(currently pregnant)** | % of currently pregnant women (aged 15 to 49 years) who received treatment for anemia after being identified as anemic during their current pregnancy | Number of currently pregnant women (aged 15 to 49 years) who received treatment for anemia after being identified as anemic during their current pregnancy | Total number of currently pregnant women (aged 15-49 years) who were identified as anemic |
| **Weighing**  **(currently pregnant)** | % of currently pregnant women (aged 15 to 49 years) who weighed during their current pregnancy by any provider | Number of currently pregnant women (aged 15 to 49 years) who weighed during their current pregnancy by any provider | Total number of currently pregnant women (aged 15-49 years) |
| **Counseling- about weight**  **(currently pregnant)** | % of currently pregnant women (aged 15 to 49 years) who were counseled after weighing during their current pregnancy by any provider | Number of currently pregnant women (aged 15 to 49 years) who were counseled after weighing during their current pregnancy by any provider | Total number of currently pregnant women (aged 15-49 years) |
| **Underweight**  **(currently pregnant)** | % of currently pregnant women (aged 15 to 49 years) who were identified as malnourished during their current pregnancy | Number of currently pregnant women (aged 15 to 49 years) who were identified as underweight during their current pregnancy | Total number of currently pregnant women (aged 15-49 years) |
| **Supplementary nutrition**  **(currently pregnant)** | % of currently pregnant women (aged 15 to 49 years) who were identified as malnourished and received supplementary nutrition information during their current pregnancy | Number of currently pregnant women (aged 15 to 49 years) who were identified as malnourished and received information on how to prepare nutritious foods like khichuri and halwa from a health care provider | Total number of currently pregnant women (aged 15-49 years) who were identified as malnourished during their current pregnancy |
| **Regular use of mosquito net**  **(currently pregnant)** | % of currently pregnant women (aged 15 to 49 years) who are using mosquito net regularly during their current pregnancy | Number of currently pregnant women (aged 15 to 49 years) who are using mosquito net regularly during their current pregnancy | Total number of currently pregnant women (aged 15-49 years) |
| **Malaria prevention**  **(currently pregnant)** | % of currently pregnant women (aged 15 to 49 years) who are taking FP Fansidar during their current pregnancy | Number of currently pregnant women (aged 15 to 49 years) who are taking FP Fansidar during their current pregnancy | Total number of currently pregnant women (aged 15-49 years) |
| **Counseling- diet**  **(currently pregnant)** | % of currently pregnant women (aged 15 to 49 years) who were counseled on eating an additional amount of food and a variety of foods during their current pregnancy | Number of currently pregnant women (aged 15 to 49 years) who were counseled on eating an additional amount of food and a variety of foods during their current pregnancy | Total number of currently pregnant women (aged 15-49 years) |
| **Counseling- institutional delivery**  **(currently pregnant)** | % of currently pregnant women (aged 15 to 49 years) in their third trimester who were counseled on importance of institutional delivery during their current pregnancy | Number of currently pregnant women (aged 15 to 49 years) in their third trimester who were counseled on importance of institutional delivery during their current pregnancy | Total number of currently pregnant women (aged 15-49 years) in their 3rd trimester |
| **Counseling- cord care**  **(currently pregnant)** | % of currently pregnant women (aged 15 to 49 years) in their third trimester who were counseled about cord care during their current pregnancy | Number of currently pregnant women (aged 15 to 49 years) in their third trimester who were counseled on cord care during their current pregnancy | Total number of currently pregnant women (aged 15-49 years) in their 3rd trimester |
| **Counseling- Exclusive breastfeeding**  **(currently pregnant)** | % of currently pregnant women (aged 15 to 49 years) in their third trimester who were counseled about exclusive breastfeeding during their current pregnancy | Number of currently pregnant women (aged 15 to 49 years) in their third trimester who were counseled about exclusive breastfeeding during their current pregnancy | Total number of currently pregnant women (aged 15-49 years) in their 3rd trimester |
| **Counseling- keeping baby warm**  **(currently pregnant)** | % of currently pregnant women (aged 15 to 49 years) in their third trimester who were counseled about keeping baby warm during their current pregnancy | Number of currently pregnant women (aged 15 to 49 years) in their third trimester who were counseled about keeping baby warm during their current pregnancy | Total number of currently pregnant women (aged 15-49 years) in their 3rd trimester |
| **Maternal Indicators**  ***Previous pregnancy*** | **Definition/Indicator** | **Numerator** | **Denominator** |
| **Antenatal care- At least once** | % of women (aged 15-49 years) with a live and/or still birth in the last 2 years preceding the survey who received at least once during their pregnancy that led to a live and/or still birth by any provider | Number of women (aged 15-49 years) with a live and/or still birth in the last 2 years preceding the survey who were attended for ANC at least once during their pregnancy that led to a live and/or still birth by any provider | Total number of women (aged 15–49 years) with a live and/or still birth in the last 2 years |
| **Antenatal care- Four ANC** | % of women (aged 15-49 years) with a live and/or still birth in the last 2 years preceding the survey who received ANC at least four times during their pregnancy that led to a live and/or still birth by any provider | Number of women (aged 15-49 years) with a live and/or still birth in the last 2 years preceding the survey who were attended ANC at least four times during their pregnancy that led to a live and/or still birth by any provider | Total number of women (aged 15–49 years) with a live and/or still birth in the last 2 years |
| **Antenatal care- Early ANC** | % of women (aged 15-49 years) with a live and/or still birth in the last 2 years preceding the survey who were attended early (in the first trimester) during their pregnancy that led to a live and/or still birth by any provider | Number of women (aged 15-49 years) with a live and/or still birth in the last 2 years preceding the survey who were attended ANC early (in the first trimester) during their pregnancy that led to a live and/or still birth by any provider | Total number of women (aged 15–49 years) with a live and/or still birth in the last 2 years |
| **Supplementation - Any iron supplementation** | % of women (aged 15-49 years) with a live and/or still birth in the last 2 years preceding the survey who bought or received any tablet or syrup that contains iron during their pregnancy that led to a live and/or still birth | Number of women (aged 15-49 years) with a live and/or still birth in the last 2 years preceding the survey who bought or received any tablet or syrup that contains iron during their pregnancy that led to a live and/or still birth | Total number of women (aged 15–49 years) with a live and/or still birth in the last 2 years |
| **Supplementation type-supplements with multiple micronutrients** | % of women (aged 15-49 years) with a live and/or still birth in the last 2 years preceding the survey who bought or received supplements with multiple micronutrients (MMS or FullCare) during their pregnancy that led to live and/or still birth | Number of women (aged 15-49 years) with a live and/or still birth in the last 2 years preceding the survey who bought or received supplements with multiple micronutrients (MMS or FullCare) during their pregnancy that led to live and/or still birth | Total number of women (aged 15–49 years) with a live and/or still birth in the last 2 years |
| **Supplementation type- iron tablet/iron folic acid** | % of women (aged 15-49 years) with a live and/or still birth in the last 2 years preceding the survey who bought or received iron tablet/iron folic acid during their pregnancy that led to live and/or still birth | Number of women (aged 15-49 years) with a live and/or still birth in the last 2 years preceding the survey who bought or received iron tablet/iron folic acid during their pregnancy that led to live and/or still birth | Total number of women (aged 15–49 years) with a live and/or still birth in the last 2 years |
| **Supplementation type- others** | % of women (aged 15-49 years) with a live and/or still birth in the last 2 years preceding the survey who bought or received any other supplements containing iron or folic acid during their pregnancy that led to live and/or still birth | Number of women (aged 15-49 years) with a live and/or still birth in the last 2 years preceding the survey who bought or received any other supplements containing iron or folic acid during their pregnancy that led to live and/or still birth | Total number of women (aged 15–49 years) with a live and/or still birth in the last 2 years |
| **Iron supplementation type- timing of initiation** | % of women (aged 15-49 years) with a live and/or still birth in the last 2 years preceding the survey started consuming any iron-containing supplements during [1st Trimester] | Number of women (aged 15-49 years) with a live and/or still birth in the last 2 years preceding the survey started consuming any iron-containing supplements during [1st Trimester] | Total number of women (aged 15–49 years) with a live and/or still birth in the last 2 years |
| **Iron supplementation - days consumed- 90+ days** | % of women (aged 15-49 years) with a live or still birth in the last 2 years preceding the survey who consumed any iron containing supplements for at least 90/180 days during their pregnancy that led to live or still birth | Number of women (aged 15-49 years) with a live or still birth in the last 2 years preceding the survey who consumed any iron containing supplements for at least 90+ or 180+ days during their pregnancy that led to live or still birth: | Total number of women (aged 15–49 years) with a live and/or still birth in the last 2 years |
| **Iron supplementation - months of supplementation** | % of women (aged 15-49 years) with a live and/or still birth in the last 2 years preceding the survey who consumed any iron containing supplements for at least following months of their pregnancy that led to a live and/or still birth  I. 1-3 months  ii. 4-6 months  iii. 7-9 months | Number of women (aged 15-49 years) with a live and/or still birth in the last 2 years preceding the survey who consumed any iron containing supplements for at least following months of their pregnancy that led to a live and/or still birth  I. 1-3 months  ii. 4-6 months  iii. 7-9 months | Total number of women (aged 15–49 years) with a live and/or still birth in the last 2 years |
| **Iron supplementation -typical adherence per month** | % of women (aged 15-49 years) with a live and/or still birth in the last 2 years preceding the survey who reported typical consumption of iron containing supplements for at least following days per month during their pregnancy that led to live and/or still birth  i. 1-9 days   ii. 10-19 days  iii. 20-30 days | Number of women (aged 15-49 years) with a live and/or still birth in the last 2 years preceding the survey who reported typical consumption of iron containing supplements for at least following days per month during their pregnancy that led to live and/or still birth  i. 1-9 days   ii. 10-19 days  iii. 20-30 days | Total number of women (aged 15–49 years) with a live and/or still birth in the last 2 years |
| **Iron supplementation - counseling** | % of women (aged 15-49 years) with a live and/or still birth in the last 2 years preceding the survey who were counseled on taking iron containing supplements and its benefits and side effects during their pregnancy that led to live and/or still birth | Number of women (aged 15-49 years) with a live and/or still birth in the last 2 years preceding the survey who were counseled on taking iron containing supplements and its benefits and side effects during their pregnancy that led to live and/or still birth | Total number of women (aged 15–49 years) with a live and/or still birth in the last 2 years |
| **Supplementation - calcium** | % of women (aged 15-49 years) with a live and/or birth in the last 2 years preceding the survey who received or bought any tablets that contained calcium during their pregnancy that led to a live and/or still birth | Number of women (aged 15-49 years) with a live and/or birth in the last 2 years preceding the survey who received or bought any tablets that contained calcium during their pregnancy that led to a live or still birth | Total number of women (aged 15–49 years) with a live and/or still birth in the last 2 years |
| **Calcium supplementation type- timing of initiation** | % of women (aged 15-49 years) with a live or still birth in the last 2 years preceding the survey started consuming calcium supplements during [1st Trimester] | Number of women (aged 15-49 years) with a live and/or still birth in the last 2 years preceding the survey started consuming calcium supplements during [1st Trimester] | Total number of women (aged 15–49 years) with a live and/or still birth in the last 2 years |
| **Calcium supplementation - months of supplementation** | % of women (aged 15-49 years) with a live and/or still birth in the last 2 years preceding the survey who consumed any calcium supplements for at least following months of their pregnancy that led to a live and/or still birth  I. 1-3 months  ii. 4-6 months  iii. 7-9 months | Number of women (aged 15-49 years) with a live and/or still birth in the last 2 years preceding the survey who consumed any calcium supplements for at least following months of their pregnancy that led to a live and/or still birth  I. 1-3 months  ii. 4-6 months  iii. 7-9 months | Total number of women (aged 15–49 years) with a live and/or still birth in the last 2 years |
| **Calcium supplementation - typical adherence per month** | % of women (aged 15-49 years) with a live and/or still birth in the last 2 years preceding the survey who reported typical consumption of calcium supplements for at least following days per month during their pregnancy that led to live and/or still birth  i. 1-9 days   ii. 10-19 days  iii. 20-30 days | % of women (aged 15-49 years) with a live and/or still birth in the last 2 years preceding the survey who reported typical consumption of calcium supplements for at least following days per month during their pregnancy that led to live and/or still birth  i. 1-9 days   ii. 10-19 days  iii. 20-30 days | Total number of women (aged 15–49 years) with a live and/or still birth in the last 2 years |
| **Calcium supplementation - days consumed- 1-89 days** | % of women (aged 15-49 years) with a live and/or still birth in the last 2 years preceding the survey who consumed any calcium supplements for at least following days during their pregnancy that led to live and/or still birth:  i. 90+ days  ii. 180+ days | Number of women (aged 15-49 years) with a live and/or still birth in the last 2 years preceding the survey who consumed any calcium supplements for at least following days during their pregnancy that led to live and/or still birth:  i. 90+ days  ii. 180+ days | Total number of women (aged 15–49 years) with a live and/or still birth in the last 2 years |
| **Iron supplementation - counseling** | % of women (aged 15-49 years) with a live and/or still birth in the last 2 years preceding the survey who were counseled on taking iron containing supplements and its benefits and side effects during their pregnancy that led to live and/or still birth | Number of women (aged 15-49 years) with a live and/or still birth in the last 2 years preceding the survey who were counseled on taking iron containing supplements and its benefits and side effects during their pregnancy that led to live and/or still birth | Total number of women (aged 15–49 years) with a live and/or still birth in the last 2 years |
| **Supplementation- vitamin A** | % of women (aged 15-49 years) with a live and/or still birth in the last 2 years preceding the survey who bought or received vitamin A capsule during their pregnancy that led to a live and/or still birth | Number of women (aged 15-49 years) with a live and/or still birth in the last 2 years preceding the survey who bought or received vitamin A capsule during their pregnancy that led to a live and/or still birth | Total number of women (aged 15–49 years) with a live and/or still birth in the last 2 years |
| **Deworming** | % of women (aged 15-49 years) with a live and/or still birth in the last 2 years preceding the survey who bought or received deworming tablets during their pregnancy that led to a live and/or still birth | Number of women (aged 15-49 years) with a live and/or still birth in the last 2 years preceding the survey who bought or received deworming tablets during their pregnancy that led to a live and/or still birth | Total number of women (aged 15–49 years) with a live and/or still birth in the last 2 years |
| **Tetanus toxoid** | % of women (aged 15-49 years) with a live birth in the last 2 years preceding the survey who received 2 tetanus shots during their pregnancy that led to a live birth | Number of women (aged 15-49 years) with a live birth in the last 2 years preceding the survey who received 2 tetanus shots during their pregnancy that led to a live birth | Total number of women (aged 15–49 years) with a live birth in the last 2 years |
| **Antenatal care- Hypertension screening** | % of women (aged 15-49 years) with a live and/or still birth in the last 2 years preceding the survey who had their blood pressure measured during their pregnancy that led to a live and/or still birth | Number of women (aged 15-49 years) with a live and/or still birth in the last 2 years preceding the survey who had their blood pressure measured during their pregnancy that led to a live and/or still birth | Total number of women (aged 15–49 years) with a live and/or still birth in the last 2 years |
| **Antenatal care- Diabetes screening** | % of women (aged 15-49 years) with a live and/or still birth in the last 2 years preceding the survey who had their blood glucose level tested during their pregnancy that led to a live and/or still birth | Number of women (aged 15-49 years) with a live and/or still birth in the last 2 years preceding the survey who had their blood glucose level tested during their pregnancy that led to a live and/or still birth | Total number of women (aged 15–49 years) with a live and/or still birth in the last 2 years |
| **Antenatal care- Anemia screening** | % of women (aged 15-49 years) with a live and/or still birth in the last 2 years preceding the survey who had their hemoglobin level tested during their pregnancy that led to a live and/or still birth | Number of women (aged 15-49 years) with a live and/or still birth in the last 2 years preceding the survey who had their hemoglobin level tested during their pregnancy that led to a live and/or still birth | Total number of women (aged 15–49 years) with a live and/or still birth in the last 2 years |
| **Antenatal care- Anemia diagnosis** | % of women (aged 15-49 years) with a live and/or still birth in the last 2 years preceding the survey who were diagnosed with anemia during their pregnancy that led to a live and/or still birth | Number of women (aged 15-49 years) with a live and/or still birth in the last 2 years preceding the survey who were diagnosed with anemia during their pregnancy that led to a live and/or still birth | Total number of women (aged 15–49 years) with a live and/or still birth in the last 2 years |
| **Antenatal care- Anemia treatment** | % of women (aged 15-49 years) with a live and/or still birth in the last 2 diagnosed as anemic during their during their pregnancy that led to a live and/or still birth also received treatment for anemia | Number of women (aged 15-49 years) with a live and/or still birth in the last 2 diagnosed as anemic during their during their pregnancy that led to a live and/or still birth also received treatment for anemia | Total number of women (aged 15–49 years) with a live and/or still birth in the last 2 years identified as anemic during their last pregnancy |
| **Weighing** | % of women (aged 15-49 years) with a live and/or still birth in the last 2 years preceding the survey who were weighed during their pregnancy that led to a live and/or still birth | Number of women (aged 15-49 years) with a live and/or still birth in the last 2 years preceding the survey who were weighed during their pregnancy that led to a live and/or still birth | Total number of women (aged 15–49 years) with a live and/or still birth in the last 2 years |
| **Counseling- about weight** | % of women (aged 15-49 years) with a live and/or still birth in the last 2 years preceding the survey who were counseled about their weight after being weighed during their pregnancy that led to a live and/or still birth by any provider | Number of women (aged 15-49 years) with a live and/or still birth in the last 2 years preceding the survey who were counseled about their weight after being weighed during their pregnancy that led to a live and/or still birth by any provider | Total number of women (aged 15–49 years) with a live and/or still birth in the last 2 years |
| **Malnourished- past pregnancy** | % of women (aged 15-49 years) with a live and/or still birth in the last 2 years preceding the survey who were identified as malnourished during their pregnancy that led to a live and/or still birth | Number of women (aged 15-49 years) with a live and/or still birth in the last 2 years preceding the survey who were identified as malnourished during their pregnancy that led to a live and/or still birth | Total number of women (aged 15–49 years) with a live and/or still birth in the last 2 years |
| **Supplementary nutrition- past pregnancy** | % of women (aged 15-49 years) with a live and/or still birth in the last 2 years preceding the survey who were identified as malnourished also received a counseling on how to prepare foods like khichuri and halwa by any provider | Number of women (aged 15-49 years) with a live and/or still birth in the last 2 years preceding the survey who were identified as malnourished also received a counseling on how to prepare foods like khichuri and halwa by any provider | Total number of women (aged 15–49 years) with a live and/or still birth in the last 2 years who were identified as malnourished during their last pregnancy |
| **Counseling- diet** | % of women (aged 15-49 years) with a live and/or still birth in the last 2 years preceding the survey who were counseled on eating an additional amount of food during their pregnancy that led to a live and/or still birth | Number of women (aged 15-49 years) with a live and/or still birth in the last 2 years preceding the survey who were counseled on eating an additional amount of food during their pregnancy that led to a live and/or still birth | Total number of women (aged 15–49 years) with a live and/or still birth in the last 2 years |
| **Counseling- institutional delivery** | % of women (aged 15-49 years) with a live and/or still birth in the last 2 years preceding the survey who were counseled on importance of institutional delivery during their pregnancy that led to a live and/or still birth | Number of women (aged 15-49 years) with a live and/or still birth in the last 2 years preceding the survey who were counseled on importance of institutional delivery during their pregnancy that led to a live and/or still birth | Total number of women (aged 15–49 years) with a live and/or still birth in the last 2 years |
| **Counseling- cord care** | % of women (aged 15-49 years) with a live and/or still birth in the last 2 years preceding the survey who were counseled on cord care during their pregnancy that led to a live and/or still birth | Number of women (aged 15-49 years) with a live and/or still birth in the last 2 years preceding the survey who were counseled on cord care during their pregnancy that led to a live and/or still birth | Total number of women (aged 15–49 years) with a live and/or still birth in the last 2 years |
| **Counseling- Exclusive breastfeeding** | % of women (aged 15-49 years) with a live and/or still birth in the last 2 years preceding the survey who were counseled on exclusive breastfeeding during their pregnancy that led to a live and/or still birth | Number of women (aged 15-49 years) with a live and/or still birth in the last 2 years preceding the survey who were counseled on exclusive breastfeeding during their pregnancy that led to a live and/or still birth | Total number of women (aged 15–49 years) with a live and/or still birth in the last 2 years |
| **Counseling- keeping baby warm** | % of women (aged 15-49 years) with a live and/or still birth in the last 2 years preceding the survey who were counseled on keeping baby warm during their pregnancy that led to a live and/or still birth | Number of women (aged 15-49 years) with a live and/or still birth in the last 2 years preceding the survey who were counseled on keeping baby warm during their pregnancy that led to a live and/or still birth | Total number of women (aged 15–49 years) with a live and/or still birth in the last 2 years |
| **Regular use of mosquito net** | % of women (aged 15-49 years) with a live and/or still birth in the last 2 years preceding the survey who regularly used mosquito net during their pregnancy that led to a live and/or still birth | Number of women (aged 15-49 years) with a live and/or still birth in the last 2 years preceding the survey who regularly used mosquito net during their pregnancy that led to a live and/or still birth | Total number of women (aged 15–49 years) with a live and/or still birth in the last 2 years |
| **Malaria prevention** | % of women (aged 15-49 years) with a live and/or still birth in the last 2 years preceding the survey who took FP Fansidar during their pregnancy that led to a live and/or still birth | Number of women (aged 15-49 years) with a live and/or still birth in the last 2 years preceding the survey who took FP Fansidar during their pregnancy that led to a live and/or still birth | Total number of women (aged 15–49 years) with a live and/or still birth in the last 2 years |
| **Institutional delivery** | % of live and/or still birth in the last 2 years delivered in a health facility | Number of live and/or still birth in the last 2 years delivered in a health facility | Total live and/or still birth in the last 2 years |
| **Skilled birth attendant** | % of live and/or still birth in the last 2 years assisted by a skilled birth attendant | Number of live and/or still birth in the last 2 years assisted by a skilled birth attendant | Total live and/or still birth in the last 2 years |
| **Delivery- Caesarean section** | % of live and/or still birth in the last 2 years delivered operatively | Number of live and/or still birth in the last 2 years delivered operatively | Total live and/or still birth in the last 2 years |
| **Skin to skin contact within 1 hour of delivery** | % of most recent live births in the last 2 years preceding the survey who were put in mother's bare skin within an hour following the delivery | Number of most recent live births in the last 2 years preceding the survey who were put in mother's bare skin within an hour following the delivery | Total most recent live births in the last 2 years |
| **Weighed** | % of live births in the last 2 years preceding the survey who were weighed following the delivery | Number of live births in the last 2 years preceding the survey who were weighed following the delivery | Total live births in the last 2 years |
| **Low birthweight** | % of live births in the last 2 years preceding the survey who had low birthweight (birthweight<2500 grams) | Number of live births in the last 2 years preceding the survey who had low birthweight (birthweight<2500 grams) | Total live births in the last 2 years |
| **Kangaroo mother care- counseling** | % of women (aged 15-49 years) whose most recent live birth in the last 2 years was low birthweight (birthweight<2500 grams) and received information about kangaroo mother care (either early, continous and prolonged skin to skin contact or frequent and exclusive breastfeeding) by any provider | Number of women (aged 15-49 years) whose most recent live birth in the last 2 years was low birthweight (birthweight<2500 grams) and received information about kangaroo mother care by any provider | Total number of women (aged 15–49 years) with a most recent live birth in the last 2 years that were low birthweight |
| **Postnatal care for women** | % of women (aged 15-49 years) with a most recent live and/or stillbirth in the 2 years preceding the survey who received a postnatal check during the first 2 days after giving birth by any provider. | Number of women (aged 15-49 years) with a most recent live and/or stillbirth in the 2 years preceding the survey who received a postnatal check during the first 2 days after giving birth by any provider. | Total number of women (aged 15–49 years) with a most recent live or still birth in the last 2 years |
| **Postnatal care for women- Blood pressure measurement** | % of women (aged 15-49 years) with a most recent live birth in the 2 years preceding the survey whose blood pressure was measured during the first 2 days after giving birth by any provider. | Number of women (aged 15-49 years) with a most recent live birth in the 2 years preceding the survey whose blood pressure was measured during the first 2 days after giving birth by any provider. | Total number of women (aged 15–49 years) with a most recent live birth in the last 2 years |
| **Postnatal care for women- Family planning** | % of women (aged 15-49 years) with a most recent live birth in the 2 years preceding the survey who was told about the family planning during the first 2 days after giving birth by any provider. | Number of women (aged 15-49 years) with a most recent live birth) in the 2 years preceding the survey who was told about the family planning during the first 2 days after giving birth by any provider. | Total number of women (aged 15–49 years) with a most live birth in the last 2 years |
| **Postnatal care for newborn** | % of most recent live births in the last 2 years preceding the survey who received a postnatal check-up during the first 2 days of birth by any provider | Number of most recent live births in the last 2 years preceding the survey who received a postnatal check-up during the first 2 days of birth by any provider | Total most recent live births in the last 2 years |
| **Postnatal care for newborn- Cord examination** | % of most recent live births in the last 2 years preceding the survey who received a cord examination during the first 2 days of birth by any provider | Number of most recent live births in the last 2 years preceding the survey who received a cord examination during the first 2 days of birth by any provider | Total most recent live births in the last 2 years |
| **Postnatal care for newborn- Temperature measurement** | % of most recent live births in the last 2 years preceding the survey whose temperature was measured during the first 2 days of birth by any provider | Number of most recent live births in the last 2 years preceding the survey whose temperature was measured received during the first 2 days of birth by any provider | Total most recent live births in the last 2 years |
| **Postnatal care for newborn- Recognizing need for immediate medical attention** | % of most recent live births in the last 2 years preceding the survey whose caretaker was told how to recognize if child needs immediate medical attention during the first 2 days of birth by any provider | Number of most recent live births in the last 2 years preceding the survey whose caretaker was told how to recognize if child needs immediate medical attention during the first 2 days of birth by any provider | Total most recent live births in the last 2 years |
| **Postnatal care for newborn- Breastfeeding counseling** | % of most recent live births in the last 2 years preceding the survey whose mother was told about breast feeding during the first 2 days of birth by any provider | Number of most recent live births in the last 2 years preceding the survey whose mother was told about breast feeding during the first 2 days of birth by any provider | Total most recent live births in the last 2 years |
| **Postnatal care for newborn- Breastfeeding observation** | % of most recent live births in the last 2 years preceding the survey whose breastfeeding was observed during the first 2 days of birth by any provider | Number of most recent live births in the last 2 years preceding the survey whose breastfeeding was observed during the first 2 days of birth by any provider | Total most recent live births in the last 2 years |
| **Postpartum IFA supplementation** | % of women (aged 15 to 49 years) with live births in the past two years who received or bought any tablets or syrup that contained iron after the delivery of all live births in the last two years | Number of women (aged 15 to 49 years) with live births in the past two years who received or bought any tablets or syrup that contained iron after the delivery of all live births in the last two years | Total number of women (aged 15–49 years) with live birth in the last 2 years |
| **Postpartum IFA supplementation- months consumed** | % of women (aged 15 to 49 years) with live births in the past two years who consumed any tablets or syrup that contained iron for six months after the delivery of all live births in the last two years | Number of women (aged 15 to 49 years) with live births in the past two years who consumed any tablets or syrup that contained iron for six months after the delivery of all live births in the last two years | Total number of women (aged 15–49 years) with live birth in the last 2 years |
| **Malnourished-postpartum** | % of women (aged 15 to 49 years) with live births in the past two years who were diagnosed as malnourished after the delivery of all live births in the last two years | Number of women (aged 15 to 49 years) with live births in the past two years who were diagnosed as malnourished after the delivery of all live births in the last two years | Total number of women (aged 15–49 years) with a most recent live birth in the last 2 years |
| **Supplementary nutrition-counseling** | % of women (aged 15 to 49 years) with live births in the past two years who were diagnosed as malnourished and received information on preparing nutritious food after the delivery of all live births in the last two years | Number of women (aged 15 to 49 years) with live births in the past two years who were diagnosed as malnourished and received information on preparing nutritious food after the delivery of all live births in the last two years | Total number of women (aged 15–49 years) with a most recent live birth in the last 2 years who were identified as malnourished |
| **Paid maternity leave** | % of women (aged 15 to 49 years) with live births in the past two years who were offered a paid maternity leave after the delivery of all live births in the last two years | Number of women (aged 15 to 49 years) with live births in the past two years who were offered a paid maternity leave after the delivery of all live births in the last two years | Total number of women (aged 15–49 years) with a most recent live birth in the last 2 years |
| **Child Indicators** | **Definition/Indicator** | **Numerator** | **Denominator** |
| **Supplementation- Vitamin A** | % of children aged 6-59 months who received vitamin A in the last 6 months | Number of children aged 6-59 months who received vitamin A in the last 6 months | Total number of children 6-59 months |
| **Deworming** | % of children aged 6-59 months who received any deworming tablets in the last 6 months | Number of children aged 6-59 months who received any deworming tablets in the last 6 months | Total number of children 6-59 months |
| **SQLNS** | % of children aged 6-59 months who received Small Quantity-Lipid based Nutritional Supplements (SQ-LNS) such as Sonamoni | Number of children aged 6-59 months who received any deworming tablets in the last 6 months | Total number of children 6-59 months |
| **Supplementation- Iron (Fe)** | % of children aged 6-59 months who received iron pill or syrup in the past 7 days | Number of children aged 6-59 months who received iron pill or syrup in the last 7 days | Total number of children 6-59 months |
| **IYCF counseling- any** | % of children 6-23 months whose primary caregivers received any IYCF counseling in the last 6 months | Number of children 6-23 months whose caregiver talked with a healthcare provider about how or what to feed their child in the last 6 months surveyed | Total number of children 6-23months |
| **IYCF counseling- age appropriate** | % of children 6-23 months whose primary caregivers received age-appropriate IYCF counseling in the last 6 months | Number of children 6-23 months whose caregiver talked with a healthcare provider about how or what to feed their child in the last 6 months surveyed | Total number of children 6-23months |
| **ORS during diarrhea** | % of children 0-59 months who had diarrhea in the past 2 weeks were given ORS. | Number of children aged 0-59 months who had diarrhea in the past two weeks were given ORS | Total number of children 0-59 months who had diarrhea in the past 2 weeks |
| **Zinc supplementation during diarrhea** | % of children 0-59 months who had diarrhea in the past 2 weeks were given Zinc supplementation. | Number of children aged 0-59 months who had diarrhea in the past two weeks were given Zinc | Total number of children 0-59 months who had diarrhea in the past 2 weeks |
| **Growth monitoring** | % of children 0-59 months whose either height, weight or mid-upper arm circumference was Measured in the last 3 months by any provider | Number of children 0-59 months whose either height, weight or mid-upper arm circumference was measured in the last 3 months by any provider | Total number of children aged 0-59 months |
| **Counseling after growth monitoring** | % of children 0-59 months whose primary care giver received information about how their children is growing after measuring either height, weight or mid-upper arm circumference in the last 3 months by any provider | Number of children 0-59 months whose primary care giver received information about how their children is growing after measuring either height, weight or mid-upper arm circumference in the last 3 months by any provider | Total number of children aged 0-59 months |
| **Undernourished** | % of children 0-59 months who were identified as undernourished in the past 3 months | Number of children 0-59 months who were identified as undernourished in the past 3 months | Total number of children aged 0-59 months who were measured (i.e., valid height, weight, or MUAC recorded in the last 3 months by any provider (identified as malnourished) |
| **Undernourished- counseling on food intake** | % of children 0-59 months who were identified as malnourished in the past 3 months and received information about food intake | Number of children 0-59 months who were identified as malnourished in the past 3 months and received information about food intake | Total children aged 0-59 months who were measured (i.e., valid height, weight, or MUAC recorded in the last 3 months by any provider (identified as malnourished) |
| **Undernourished- treatment** | % of children 0-59 months who were identified as malnourished in the past 3 months and received treatment | Number of children 0-59 months who were identified as malnourished in the past 3 months and received treatment | Total children aged 0-59 months who were measured (i.e., valid height, weight, or MUAC recorded in the last 3 months by any provider (identified as malnourished) |
| **Immunization** | % of children 12-23 months who received one dose of BCG, three doses of DPT containing vaccine, three doses of polio and one dose of measles containing vaccine | Number of children 12-23 months who received one dose of BCG, three doses of DPT containing vaccine, three doses of polio and one dose of measles containing vaccine | Total children aged 12-23 months |
| **Adolescent Indicators** | **Definition/Indicator** | **Numerator** | **Denominator** |
| **Iron supplementation - received any IFA** | % of adolescent girls (aged 10-19 years) who received or bought any tablets or syrup that contained iron during the last 3 months | Number of adolescents girls (aged 10-19 years) who received or bought any tablets or syrup that contained iron during the last 3 months | Total number of female adolescents (aged 10-19 years) |
| **Iron supplementation - received any IFA** | % of adolescent girls (aged 10-19 years) who received or bought any tablets or syrup that contained iron during the last week | Number of adolescent girls (aged 10-19 years) who received or bought any tablets or syrup that contained iron during the last week | Total number of female adolescents (aged 10-19 years) |
| **Iron supplementation - MMS tablet, FullCare, or other supplements with multiple micronutrients** | % of adolescent girls (aged 10-19 years) who received or bought MMS tablet, FullCare, or other supplements with multiple micronutrients during the last 3 months | Number of adolescent girls (aged 10-19 years) who received or bought MMS tablet, FullCare, or other supplements with multiple micronutrients during the last 3 months | Total number of female adolescents (aged 10-19 years) |
| **Iron supplementation - adherence in previous month** | % of adolescent girls (aged 10-19 years) who consumed iron-containing supplements for at least X days in previous month | Number of adolescent girls (aged 10-19 years) who consumed iron-containing supplements for at least X days in previous month | Total number of female adolescents (aged 10-19 years) |
| **Deworming** | % of adolescents (aged 10-19 years) who received any deworming tablets in the last 6 months | Number of adolescent (aged 10-19 years) who received any deworming tablets in the last 6 months | Total number of adolescents (aged 10-19 years) |
| **Food supplements in the last 1 month** | % of adolescents (aged 10-19 years) who received food supplements in the last one month | Number of adolescents (aged 10-19 years) who received food supplements in the last one month | Total number of adolescents (aged 10-19 years) |
| **Information on diverse diet** | % of adolescents (aged 10-19 years) who received information about eating five different food groups or eating a diverse diet | Number of adolescents (aged 10-19 years) who received information about eating five different food groups or eating a diverse diet | Total number of adolescents (aged 10-19 years) |
| **Information on unhealthy foods** | % of adolescents (aged 10-19 years) who received information about unhealthy foods | Number of adolescents (aged 10-19 years) who received information about uhealthy foods | Total number of adolescents (aged 10-19 years) |
| **School feeding** | % of adolescents (aged 10-19 years) who received free food from school | Number of adolescents (aged 10-19 years) who received free food from school | Total number of adolescents (aged 10-19 years) |

Preg, pregnancy; y, years old; nutr, nutrition; m, months old; Adol, adolescent; NSA, nutrition-sensitive agriculture; Vit, vitamin; FFV, fortified food vehicle, Fe, iron; FA, folic acid; BF, breastfeeding; EBF, exclusive breastfeeding; LBW, low birth weight; SAM, severe acute malnutrition; MAM, moderate acute malnutrition; MNP, micronutrient powder; Fe, iron; IFA, iron and folic acid.

**Supplemental Figure 1: Map of One Nutrition Coverage Study districts**

*
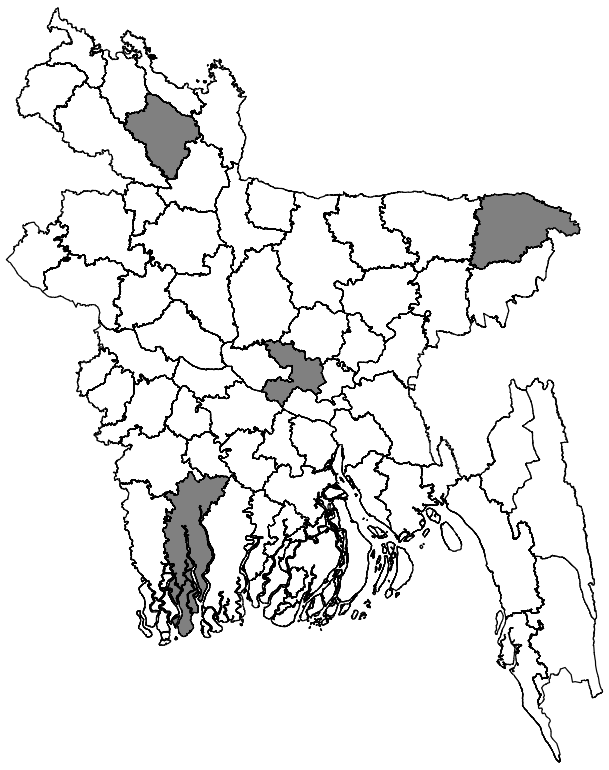
*

**KHULNA**

**RANGPUR**

**SYLHET**

**DHAKA**

**Supplemental Material 1: Questionnaires**

**ONE NUTRITION COVERAGE SURVEY - BANGLADESH**

**household listing FORM**

| **Administration level 1 (Division)**……………. [Select from drop-down list]  **Administration level 2 (District)**……….…. . [Select from drop-down list]  **Administration level 3 (City corporation)** … [Select from drop-down list]  **Administration level 4 (Upazila)**………….…. [Select from drop-down list]  **Administration level 5 (Union)**………………..[Select from drop-down list]  **Administration level 6 (Mouza)**……………….[Select from drop-down list]  **Administration level 7 (Village)**………………[Select from drop-down list] | **Administration level 8 (Enumeration Area)** …[Select from drop-down list]  **Interviewer ID** …………………….……. [Select from drop-down list]  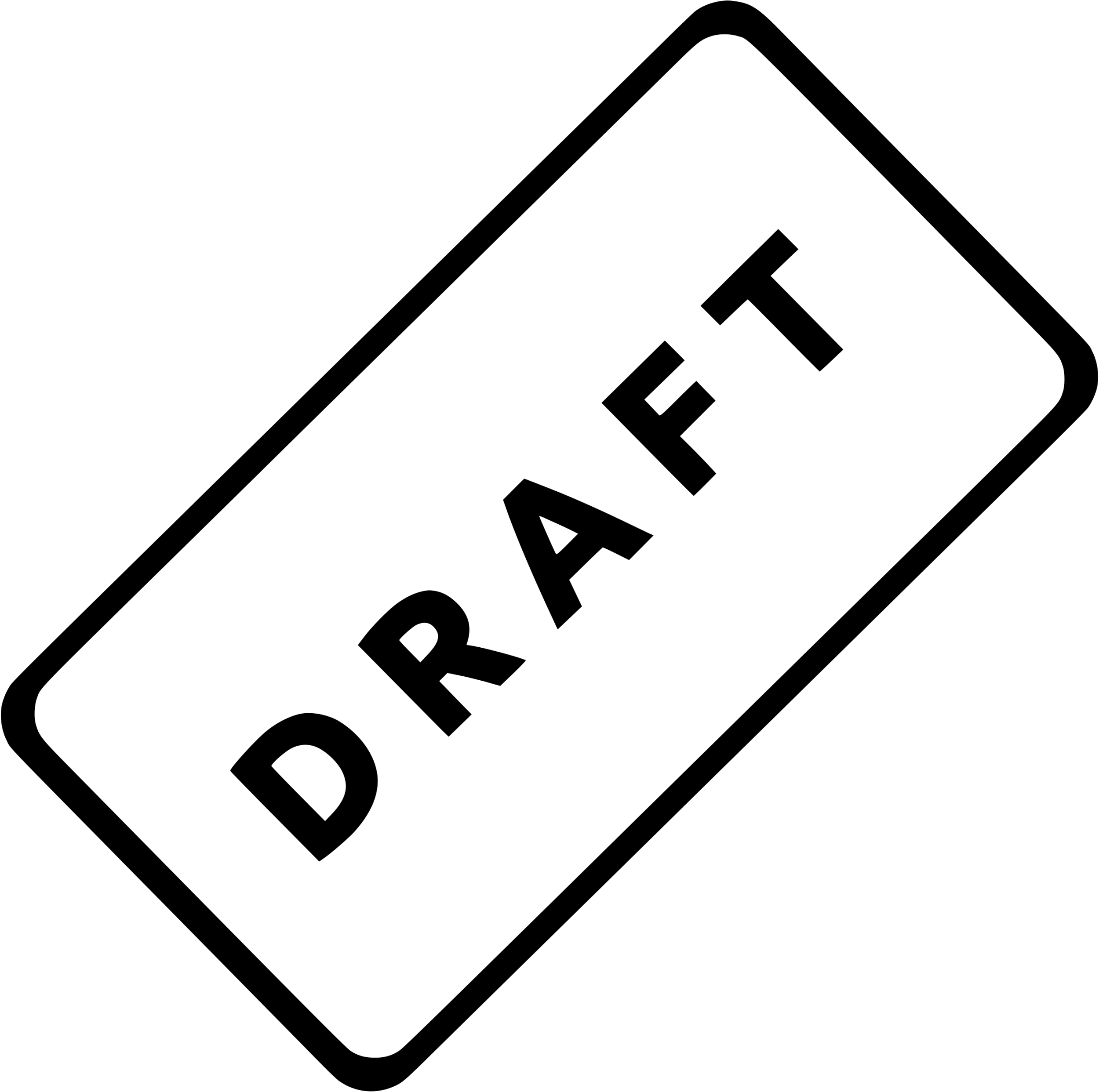**Supervisor ID**…………………………… [Select from drop-down list] | **Region of residence**: 01. Rural 02. Urban  **Listing Date** ………..……….… [Auto filled] |
| --- | --- | --- |

| **Dwelling number**  **[auto generated]** | **Address / location of dwelling** | **Type of Dwelling** | **Household number**  **[auto generated]** | **Residence?**  **Y / N** | **Full name of household head** | **Head of household phone #** | **Currently pregnant woman in household?**  **Y/N** | **GPS Coordinates**  **(Latitude, Longitude, Accuracy)** | **Observations** |
| --- | --- | --- | --- | --- | --- | --- | --- | --- | --- |
| **(1)** | **(2)** | **(3)** | **(4)** | **(5)** | **(6)** | **(7)** | **(8)** | **(9)** | **(10)** |
| *__ __ __ __*  (4-digit integer) | 1. [free text] | 1. Single HH 2. Multiple HHs FOR MULTIPLE HHs, LIST ALL HOUSEHOLDS 3. Dormitory/Hostel/Mess>>SKIP TO NEXT DWELLING | *__ __ __ __*  (4-digit integer) | 1. No>>SKIP TO NEXT STRUCTURE 2. Yes 3. Yes, but dwelling locked >> SKIP TO NEXT STRUCTURE | [free text] | (11-digit integer) | 1. No 2. Yes | Latitude: [numeric integer]  Longitude: [numeric integer]  Acc: [numeric integer] | [free text] |

# **HOUSEHOLD QUESTIONNAIRE**

Module start time XX: XX


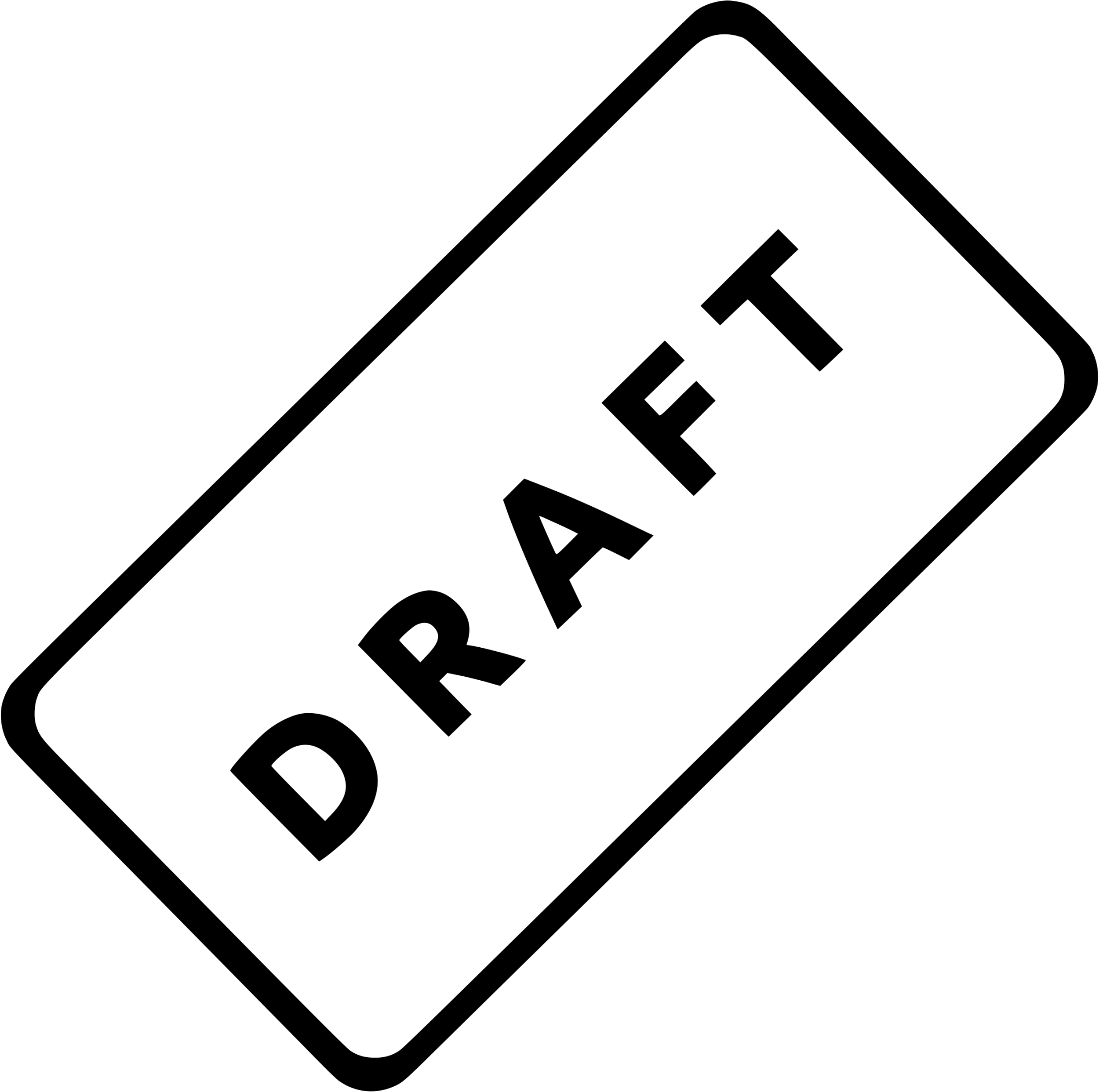


| **Survey Information (SI)** | | |
| --- | --- | --- |
| INSTRUCTION: INFORMATION IS AUTO FILLED/ TO BE FILLED BY INTERVIEWER FOR EACH INTERVIEW | | |
| **Q.no** | **Q. label** | **Response** |
| SI.1 | Interview Date | Auto filled |
| SI.2 | Interview Start Time | Auto filled |
| SI.3 | Interview End Time | Auto filled |
| SI.4 | Supervisor ID  [SINGLE SELECT] | Select from drop-down list |
| SI.5 | Interviewer ID  [SINGLE SELECT] | Select from drop-down list |
| SI.6 | Number of visits to complete questionnaire set at household  [SINGLE SELECT] | 1. One visit 2. Two visits 3. Three visits 4. Four or more visits |

Module end time XX: XX

Module start time XX: XX

| **Household Identification (HI)** | | |
| --- | --- | --- |
| CAPI Instruction: Options for HI.2 – HI.5 to be filtered based on selection in HI.1 | | |
| INSTRUCTION: INFORMATION IS TO BE FILLED BY INTERVIEWER FOR EACH INTERVIEW | | |
| **Q.no** | **Q. label** | **Response** |
| HI.1 | Administration Level 1 (Division)  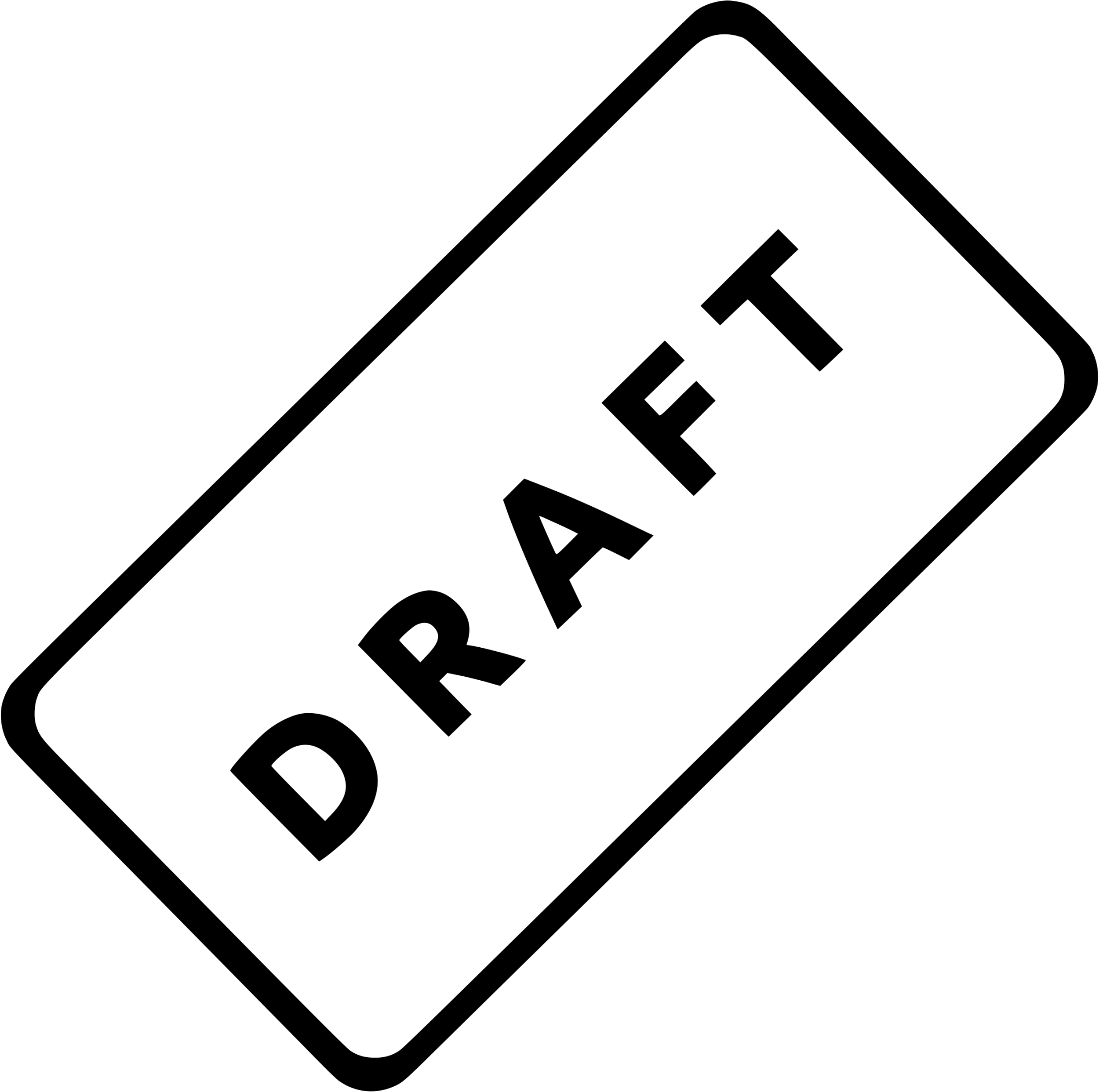  [SINGLE SELECT] | 1. Dhaka 2. Khulna 3. Rangpur 4. Sylhet |
| HI.2 | Administration Level 2 (District)  [SINGLE SELECT, CONDITIONED ON H1.1] | 1. Dhaka 2. Khulna 3. Rangpur 4. Sylhet |
| HI.3 | Administration Level 3 (City corporation)  SINGLE SELECT, CONDITIONED ON H1.2] | 1. Dhaka south city corporation 2. Dhaka north city corporation 3. Khulna city corporation 4. Sylhet city corporation 5. Rangpur city corporation 6. Other area |
| HI.4 | Administration Level 4 (Upazila)  [SINGLE SELECT, CONDITIONED ON H1.2] | Refer sampling list for name and codes  [Select from drop-down list] |
| HI.5 | Administration Level 5 (Union)  [SINGLE SELECT, CONDITIONED ON H1.4] | Refer sampling list for name and codes  [Select from drop-down list] |
| HI.6 | Administration Level 6 (Mouza)  [SINGLE SELECT, CONDITIONED ON H1.5] | Refer sampling list for name and codes  [Select from drop-down list] |
| HI.7 | Administration Level 7 (Village)  [SINGLE SELECT, CONDITIONED ON H1.6] | Refer sampling list for name and codes  [Select from drop-down list] |
| HI.8 | Administration Level 8 (Enumeration Area)  [SINGLE SELECT, CONDITIONED ON H1.7] | Refer sampling list for name and codes  [Select from drop-down list] |
| HI.9 | Region of residence | 01. Rural  02. Urban |
| HI.10 | Household Number | *___ ___*  [numeric integer, select from drop-down list (1-20)] |
| HI.11 | Is the household available for the interview?  [SINGLE SELECT] | 01. No  02. Yes >> skip to HI.13 |
| HI.12 | Why can’t the household be interviewed?  [SINGLE SELECT]  **END THE INTERVIEW** | 01. Refused  02. Not at home (door locked)  88. Other (specify) |

Module end time XX: XX

Module start time XX: XX

| **Household eligibility and consent (HE)** | | | |
| --- | --- | --- | --- |
| **Respondent: Head of household** | | | |
| INSTRUCTION: ASK TO SPEAK TO HEAD OF HOUSEHOLD AND ADMINISTER QUESTIONS BELOW TO THEM | | | |
| HE.1 | Are you the head of the household?  By “head of the household”, I mean a person who primarily makes decisions regarding household matters.  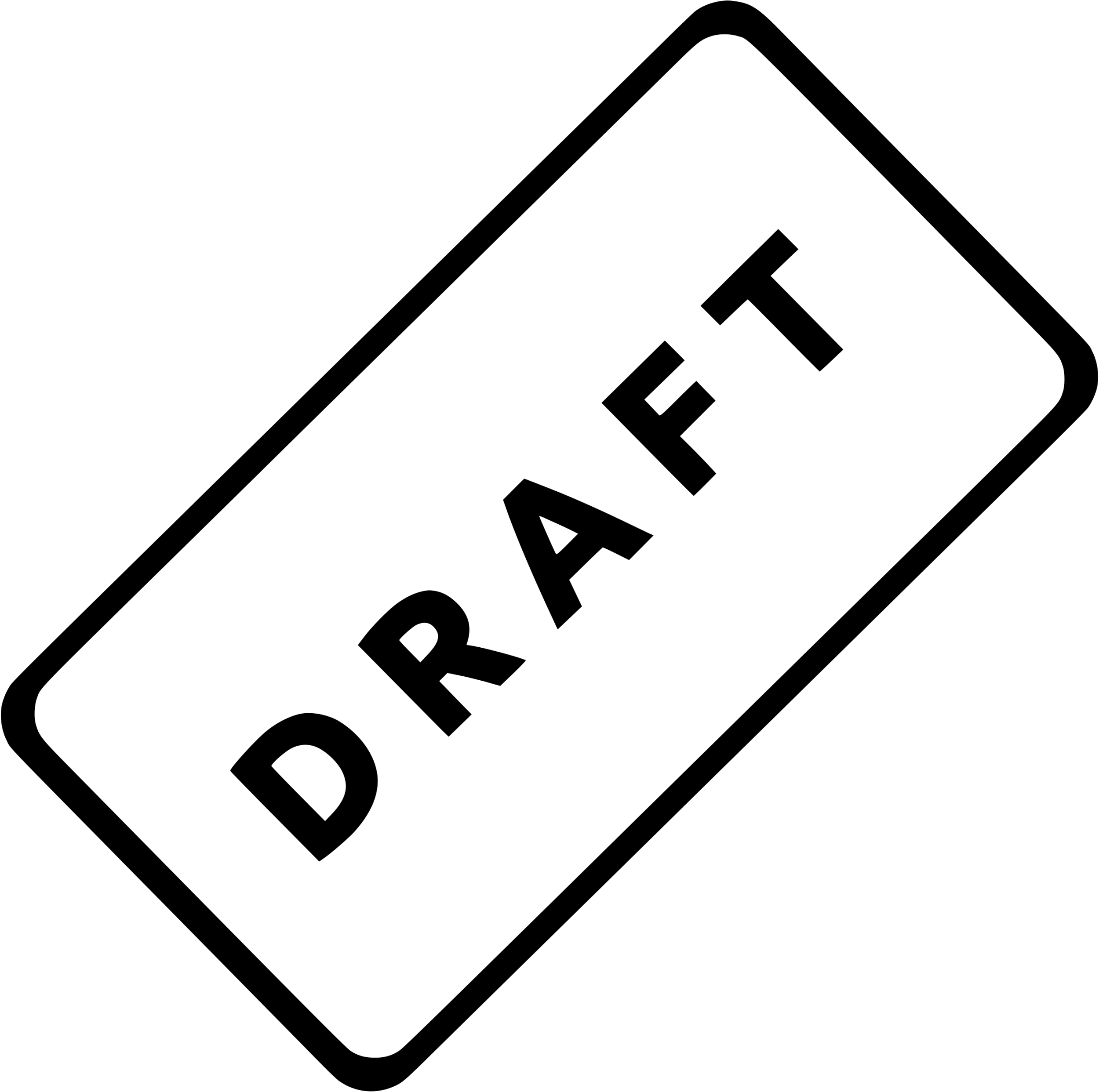[SINGLE SELECT] | 01. No >> Find Head  02. Yes>> Continue the interview | |
| **Household consent**  **Respondent: Head of household**  *Administer consent form* | | | |
| HE.2 | Do you agree to participate in the study?  [SINGLE SELECT] | | 01. No >> skip to end of survey  02. Yes |
| HE. 3 | Respondent Name | | _____________  [Name of Respondent/Household Head] |
| HE.4 | What is your religion? | | 1. Muslim 2. Hindu 3. Buddhist 4. Christian 5. Other (specify) 6. Don’t know/Don’t want to disclose |
| HE.5 | What is your ethnic group? | | 1. Bengali 2. Bihari 3. Sawtal 4. Khasia 5. Rakhain 6. Bowm 7. Chak 8. Chakma 9. Khumi 10. Kheyang 11. Lusai/pankho 12. Marma 13. Mru(murong) 14. Tonchonga 15. Tripura 16. Bonojogi 17. Other (specify) |
| HE.6 | Could you please share your phone number with me? | | ________________________  (Record phone number)  (11- digit integer) |
| HE.7 | Could you please provide an alternative number as well in case this number is unreachable? | | ________________________  (Record phone number)  (11- digit integer) |

Module end time XX: XX

Module start time XX: XX

| **Household roster and demographics (HR)** | | | | | | | |
| --- | --- | --- | --- | --- | --- | --- | --- |
| **Respondent: Head of household** | | | | | | | |
| INSTRUCTION:  Description of a household: In this survey, a household is a person or a group of persons who usually live and eat together. This is not the same as a family. A family only includes people who are related, but a household member includes any people who live together whether or not they are related. For example, if three unrelated people live and cook meals together, they would not be considered family but would be considered from the same household.  Even those persons who are not blood relations (such as servants, lodgers, or agricultural laborers) are members of the household if they stay in the household.  Consider the following when listing household members.   - A woman lists her husband as head of household, but he lives somewhere else. If he does not usually live in the household you are interviewing, and he did not stay there not previous night, he should not be included in the listing. - If a person eats in one household and sleeps in another, consider the person to be the member of the household where he/she sleeps. - A person living alone is a household. - A domestic worker is a member of the household if he/she usually lives in the household. - A visitor is not someone who is a usual member of the household but who stayed in the household the night before the day you are conducting the interview. If an individual stayed in the household the previous night, he or she should be listed in household listing. This would include a woman who has recently given birth and is now staying in her pre-marital home.   This definition of the household is very important. The criteria could be different from other studies you may be familiar with, but you should keep in mind that you should not include those people who do not meet these criteria. Please discuss any questions with your supervisor. | | | | | | | |
| 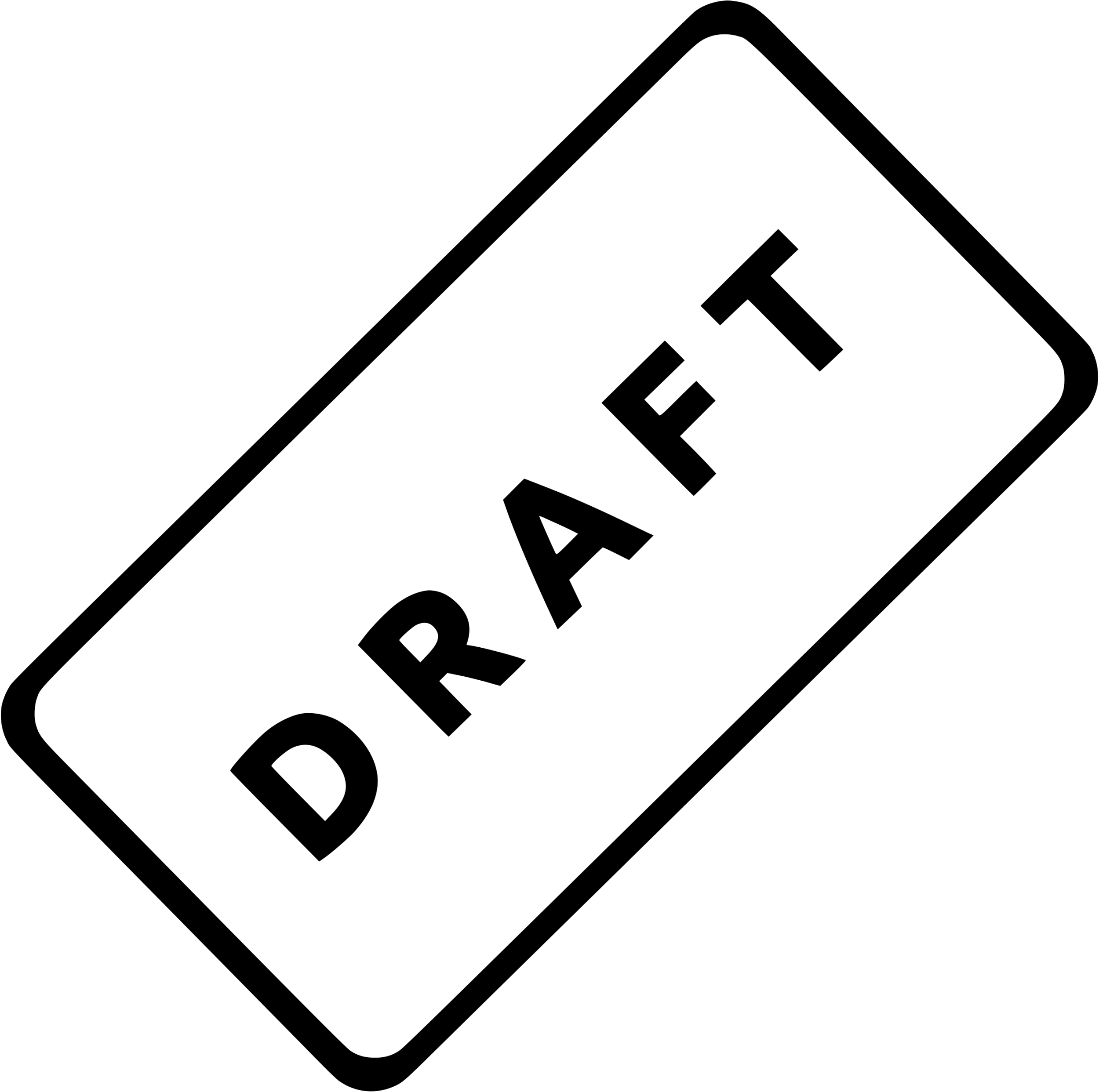I would like to ask you some questions about all the members of your household. I will describe what we mean by a household member in this survey.  In our survey, a household member is a person or a group of persons who usually live and eat together. This is not the same as a family. A family only includes people who are related, but a household member includes any people who live together whether or not they are related. For example, if three unrelated people live and cook meals together, they would not be considered family but would be considered from the same household.  Even those persons who are not blood relations (such as servants, lodgers, or agricultural laborers) are members of the household. Please also tell us about visitors who stayed in your house the night before. If someone does not sleep in this household, they are not considered a household member. | | | | | | | |
| **Q.no** | **Q. label** | **Response** | | | | | |
| HR.1 | How many people (including yourself) live in this household? | ___ ___  (Record no. HH member)  (1-50) | | | | | |
| Please give me the names of the persons who usually live in your household and guests of the household who stayed here last night. Start with the head of the household, the person who primarily makes decisions regarding household matters, next, list the spouse of the household head, followed by their children. Finally, include other members, such as servants or guests. In the case of a joint family where the household head has multiple children, along with their spouses and children, list the family of the eldest child first (eldest child, their spouse, and their children) after the household head and spouse. Then proceed with the families of the other children in order of age. Finally, include other members, such as servants or guests. | | | | | | | |
|  | *Number of columns populated from HR. 1* | Member  1  (**HH Head)** | 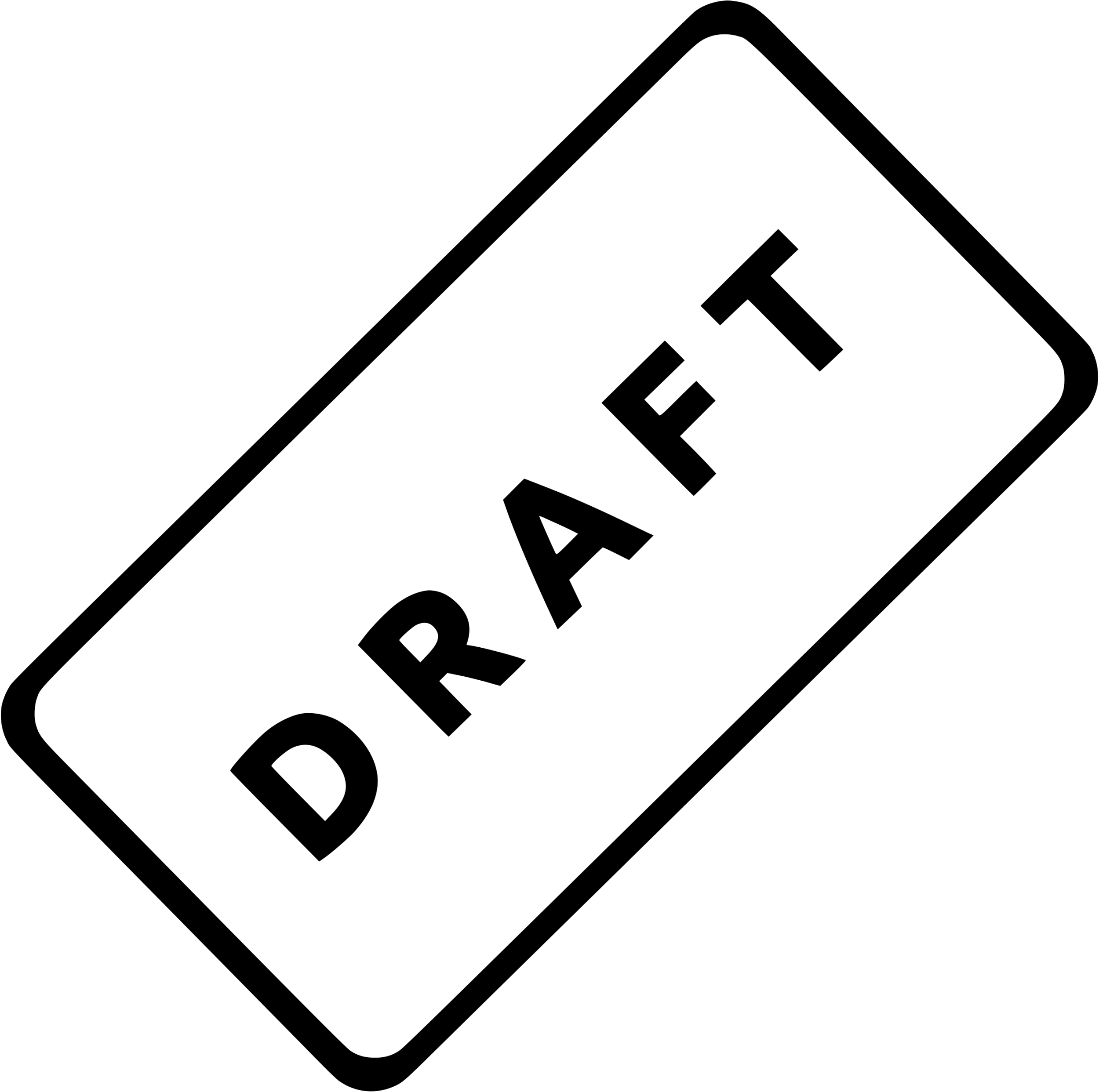Member 2 | Member 3 | Member 4 | Member 5 | Member 6 |
| HR.2 | *Unique member ID generated for each person* | Member  ID | Member ID | Member ID | Member ID | Member ID | Member ID |
| HR.3 | Member Name |  |  |  |  |  |  |
| HR.4 | Is [NAME] a usual resident of this household?   1. No 2. Yes |  |  |  |  |  |  |
| HR.5 | How old is [NAME]?  WRITE IN COMPLETED YEARS  (0-99 years)  INSTRUCTION: IF LESS THAN 12 MONTHS, THEN RECORD ‘00’. |  |  |  |  |  |  |
| HR.6 | What is [NAME’s] gender?  [SINGLE SELECT]  01. Male  02. Female  03. Hijra  88. Other |  |  |  |  |  |  |
| HR.7 | How is [NAME] related to the [Name of Head of the Household]?  [SINGLE SELECT]  01. Household Head  02. Spouse  03. Child  04. Sibling  05. Daughter/Son in law  06. Grandchild  07. Parent  08. Nephew/Niece  09. Brother/ Sister-in-law  10. Grandfather/Grandmother  11. Father-in-law/Mother-in-law  12. Employee/Domestic help  88. Other (Specify) |  |  |  |  |  |  |
| HR.8 | What is the highest education level or grade completed by [NAME]?  [SINGLE SELECT]   1. No schooling 2. Literate without schooling 3. Pre-primary 4. Class 1 completed 5. Class 2 completed 6. Class 3 completed 7. Class 4 completed 8. Class 5 completed 9. Class 6 completed 10. Class 7 completed 11. Class 8 completed 12. Class 9 completed 13. Class 10 completed 14. Class 11 completed 15. Class 12 completed 16. Undergraduate degree completed 17. Postgraduate degree completed 18. Vocational studies completed   88. Other (Specify_   1. Don’t know 2. Not applicable (for children<5 years) |  |  |  |  |  |  |
| Skip HR.9 – HR.10 if HR.5 <= 10 years | | | | | | | |
| HR.9 | What is [NAME’s] marital status?  [SINGLE SELECT]  01. Married  02. Single  03. Widowed  04. Divorced/Separated   1. Not Applicable |  |  |  |  |  |  |
| HR.10 | 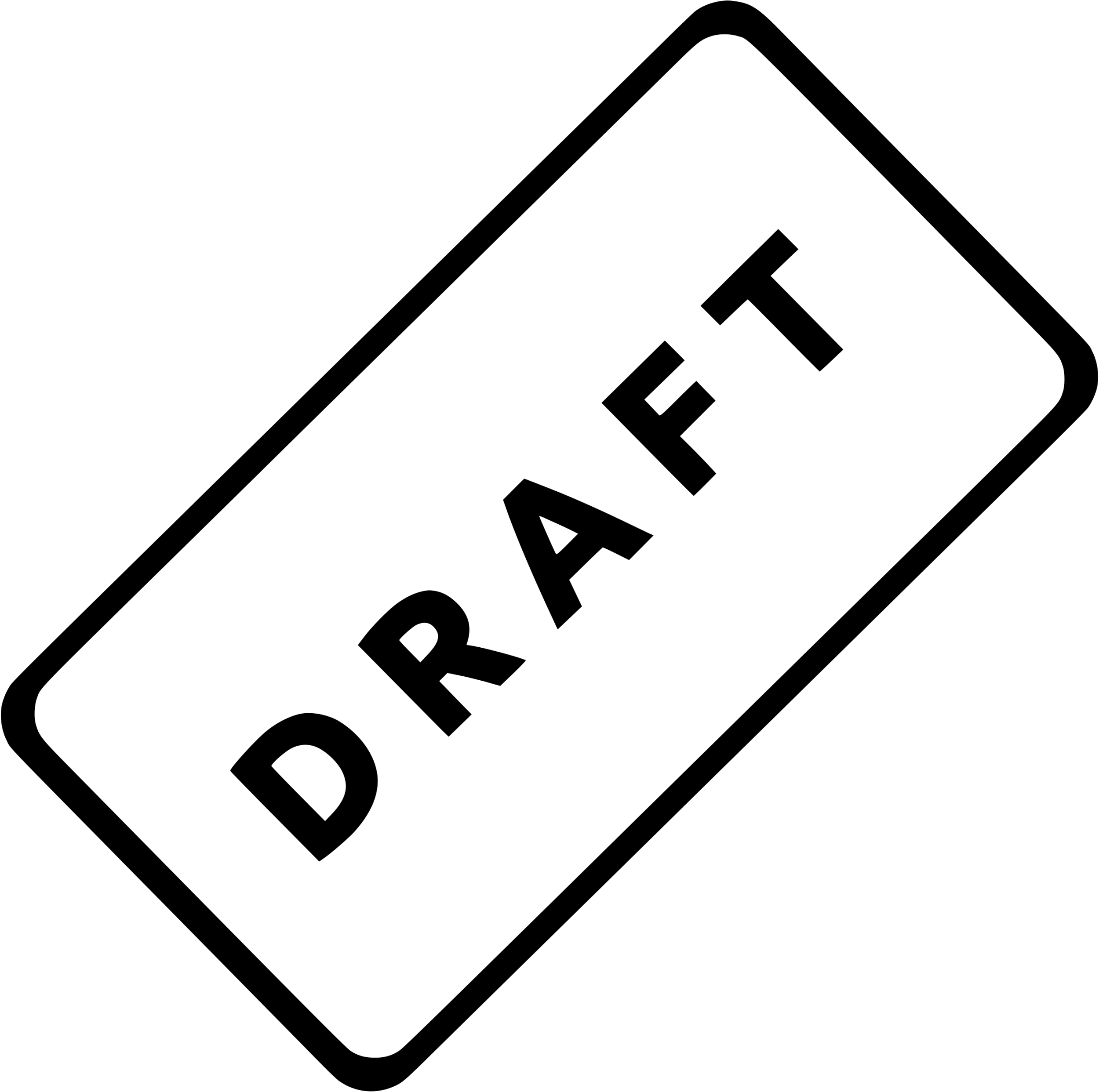What is [NAME’s] primary occupation in the last 12 months?  [SINGLE SELECT]  INSTRUCTION: THE ACTIVITY THE PERSON SPENT THE LONGEST TIME IN DURING LAST 365 DAYS PRECEDING THE SURVEY  01. Student  02. Unpaid Household Work  03. Own enterprise/ business  04. Farming (Crop cultivation, livestock, aquaculture)  05. Casual farm labour (paid)  06. Casual non-farm labour (paid)  07. Self-employed  08. Salaried Employment  09. Retired with pension  10. Retired without pension  88. Other (Specify) |  |  |  |  |  |  |

Module end time XX: XX

Module start time XX: XX

| **Asset ownership (AO)** | | |
| --- | --- | --- |
| **Respondent: Head of household** | | |
| CAPI instruction: Complete this section with the name listed in S.N.1.a of the respondent matrix.  Add Respondent ID __ | | |
| Now, I would like to ask you about assets owned by your household | | |
| **Q. no** | **Q. label** | **Response** |
| AO.1 | Does your household have the following?  INSTR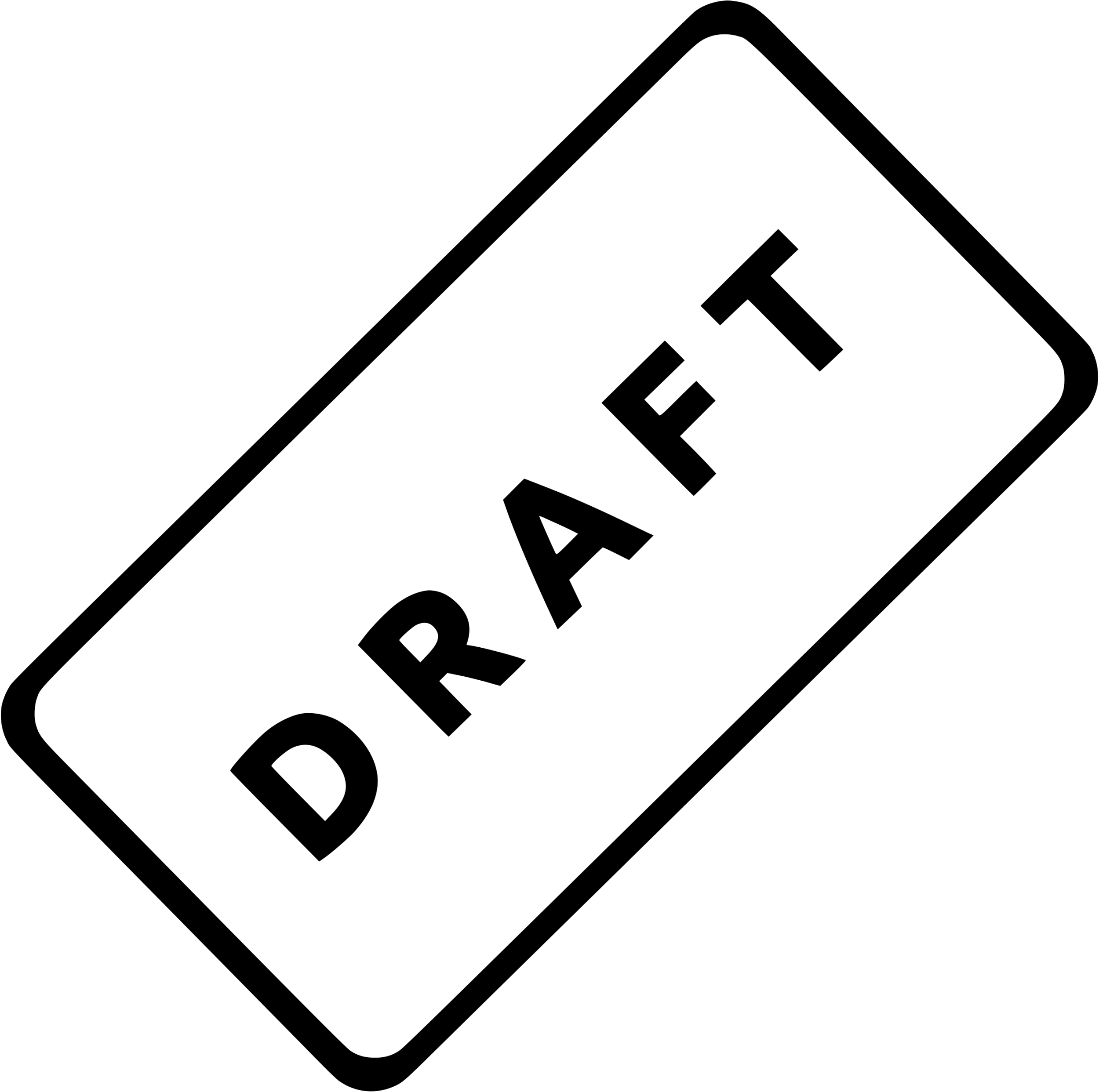UCTION: RECORD ‘YES’ IF THEY HAVE THE ITEM EVEN IF IT IS NOT IN WORKING CONDITION. IF AN ITEM CAN BE OBSERVED, YOU DO NOT NEED TO ASK THE RESPONDENT IF THE HOUSEHOLD HAS THAT ITEM. | 1. No 2. Yes   98. Don’t know |
| AO.1.1 | Electricity |  |
| AO.1.2 | Solar electricity |  |
| AO.1.3 | Radio |  |
| AO.1.4 | Television |  |
| AO.1.5 | Mobile telephone |  |
| AO.1.6 | Non-mobile telephone |  |
| AO.1.7 | Computer/laptop |  |
| AO.1.8 | Refrigerator |  |
| AO.1.9 | Almirah/wardrobe |  |
| AO.1.10 | Electric fan |  |
| AO.1.11 | DVD/CD player |  |
| AO.1.12 | Water pump |  |
| AO.1.13 | IPS generator |  |
| AO.1.14 | Air conditioner |  |
| Does any member of this household own: | | |
| AO.1.15 | Car/truck/microbus |  |
| AO 1.16 | Auto bike/tempo/CNG |  |
| AO.1.17 | Rickshaw/van |  |
| AO.1.18 | Bicycle |  |
| AO.1.19 | Motorcycle/motor scooter |  |
| AO.1.20 | Boat with motor |  |
| AO.1.21 | Canoe/boat without motor |  |

Module end time XX: XX

Module start time XX: XX

| **Access to amenities (AA)** | | |
| --- | --- | --- |
| **Respondent: Head of household** | | |
| CAPI instruction: Complete this section with the name listed in S.N.1.a of the respondent matrix.  Add Respondent ID __ | | |
| Now, I would like to ask you about the amenities available in your household. | | |
| **Q. no** | **Q. label** | **Response** |
| AA.1 | Main material of the floor  INSTRUCTION: RECORD OBSERVATION | **NATURAL FLOOR**   1. Earth/sand 2. Dung   **RUDIMENTARY FLOOR**   1. Wood planks 2. Palm/bamboo   **FINISHED FLOOR**   1. Parquet or polished wood 2. Vinyl or asphalt 3. Ceramic tiles 4. Cement 5. Carpet   88. Others (specify) |
| AA.2 | 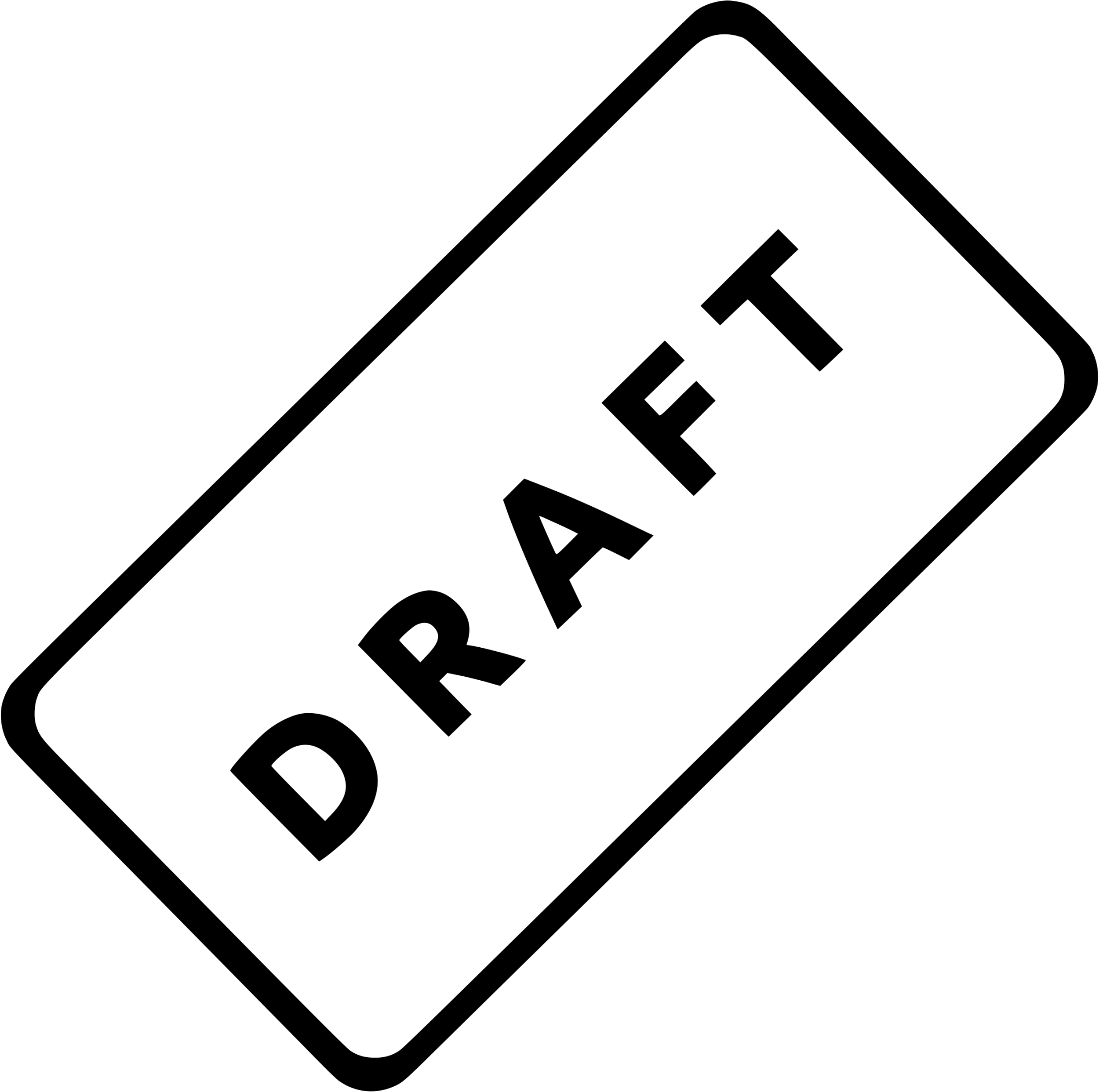Main material of the roof.  INSTRUCTION: RECORD OBSERVATION | **NATURAL ROOFING**   1. No roof 2. Thatch/palm leaf 3. SOD   **RUDIMENTARY ROOFING**   1. Mat 2. Palm/bamboo 3. Wood planks 4. Cardboard   **FINISHED ROOFING**   1. Tin/Metal 2. Wood 3. Calamine/cement fiber 4. Ceramic tiles 5. Cement 6. Roofing shingles 7. Others (specify) |
| AA.3 | Main material of the exterior walls  INSTRUCTION: RECORD OBSERVATION | **NATURAL WALLS**   1. No walls 2. Cane/palm/trunks 3. Dirt   **RUDIMENTARY WALLS**   1. Bamboo with mud 2. Stone with mud 3. Uncovered abode 4. Plywood 5. Cardboard 6. Reused wood   **FINISHED WALLS**   1. Tin 2. Cement 3. Stone with lime/cement 4. Bricks 5. Cement blocks 6. Wood planks/shingles   88. Others (specify) |
| AA.4 | What is the main source of drinking water for members in your household? | **Piped Water**   1. Piped into dwelling 2. Piped into yard/plot 3. Piped to neighbor 4. Public Taps/Standpipe 5. Tube well (Handpump) or Borehole   **Dug well**   1. Protected well 2. Unprotected well   **Water from spring**   1. Protected spring 2. Unprotected spring 3. Rainwater 4. Tanker truck 5. Cart with small tank 6. Surface water (river/dam/lake/pond/stream canal/irrigation channel) 7. Bottled water   88. Others (specify)  98. Don’t know |
| CAPI INSTRUCTION- Skip AA.5, AA.6, and AA.7 if response to AA.4 is 01, 02, or 11, 12, 14 | | |
| AA.5 | Where is that water collected from? | 1. In own dwelling 2. In own yard/plot 3. Elsewhere |
| AA.6 | How long does it take to get there, collect water and come back?  (INSTRUCTION: Record the total time including the queuing) | ___ ___ ___  (Record no. minutes) (1- 300 minutes)   1. Members do not collect   998. Don’t know |
| AA.7 | In the last month has there been anytime when your household did not have sufficient quantities of drinking water when needed? | 1. Yes, at least once 2. No, always sufficient water 3. Don’t know |
| AA.8 | Do you do anything to the water to make it safer to  drink? | 1. 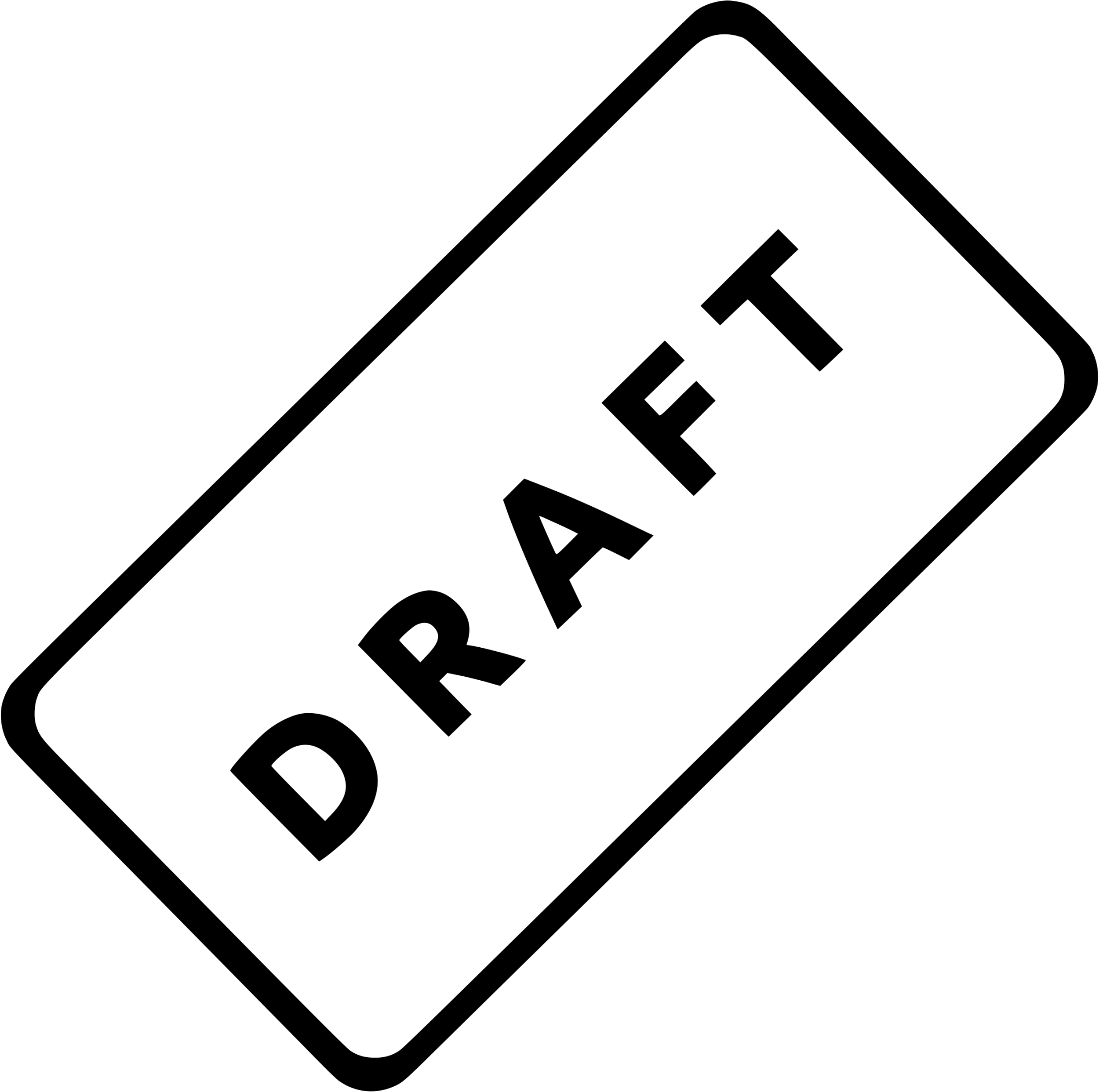No >> skip to AA.10 2. Yes   98. Don’t know>> skip to AA.10 |
| AA.9 | What do you usually do to make the water safer to  drink?  [PROBE: ANYTHING ELSE?]  [MULTI-SELECT] | 1. Boil 2. Add bleach/chlorine 3. Strain through a cloth 4. Use water filter (ceramic/sand/composite) 5. Solar disinfection 6. Let it stand and settle   88. Others (specify)  98. Don’t know |
| AA.10 | Can you please show me where members of your household most often wash their hands? | 1. In dwelling 2. In yard/plot 3. Bucket/jug/kettle 4. No handwashing place in dwelling/yard/plot 5. Others (specify) |
| AA.11 | Observe availability of water at the place for handwashing  INSTRUCTION: VERIFY BY CHECKING THE TAP/PUMP, OR BASIN, BUCKET, WATER CONTAINER OR SIMILAR OBJECTS FOR PRESENCE OF WATER | 1. Water is available 2. Water is not available |
| AA.12 | Observe availability of soap or detergent at the place for handwashing | 1. Soap is available 2. Soap is not available |
| AA.13 | What types of fuel does your household mainly use for cooking?  INSTRUCTION: RANK FIRST, SECOND AND THIRD MOST COMMONLY REPORTED  [RANK MULTI-SELECT] | 1. Electricity 2. LPG 3. Natural gas 4. Biogas 5. Kerosene 6. Coal/Lignite 7. Charcoal 8. Wood 9. Straw/grass 10. Agricultural crop waste 11. Dung cakes 12. No food cooked in the house   88. Others (specify)  98. Don’t know |
| AA.14 | Do you have a separate room which is used as a kitchen? | 1. No >> skip to AA.16 2. Yes   98. Don’t know>> skip to AA.16 |
| AA.15 | Does the room used for cooking have any ventilation? | 1. No 2. Yes   98. Don’t know |
| AA.16 | How many rooms does your home have for sleeping?  INSTRUCTION: RECORD OBSERVATION | ___ ___  (Record no. rooms)  (0-25 rooms)  98. Don’t know |
| AA.17 | 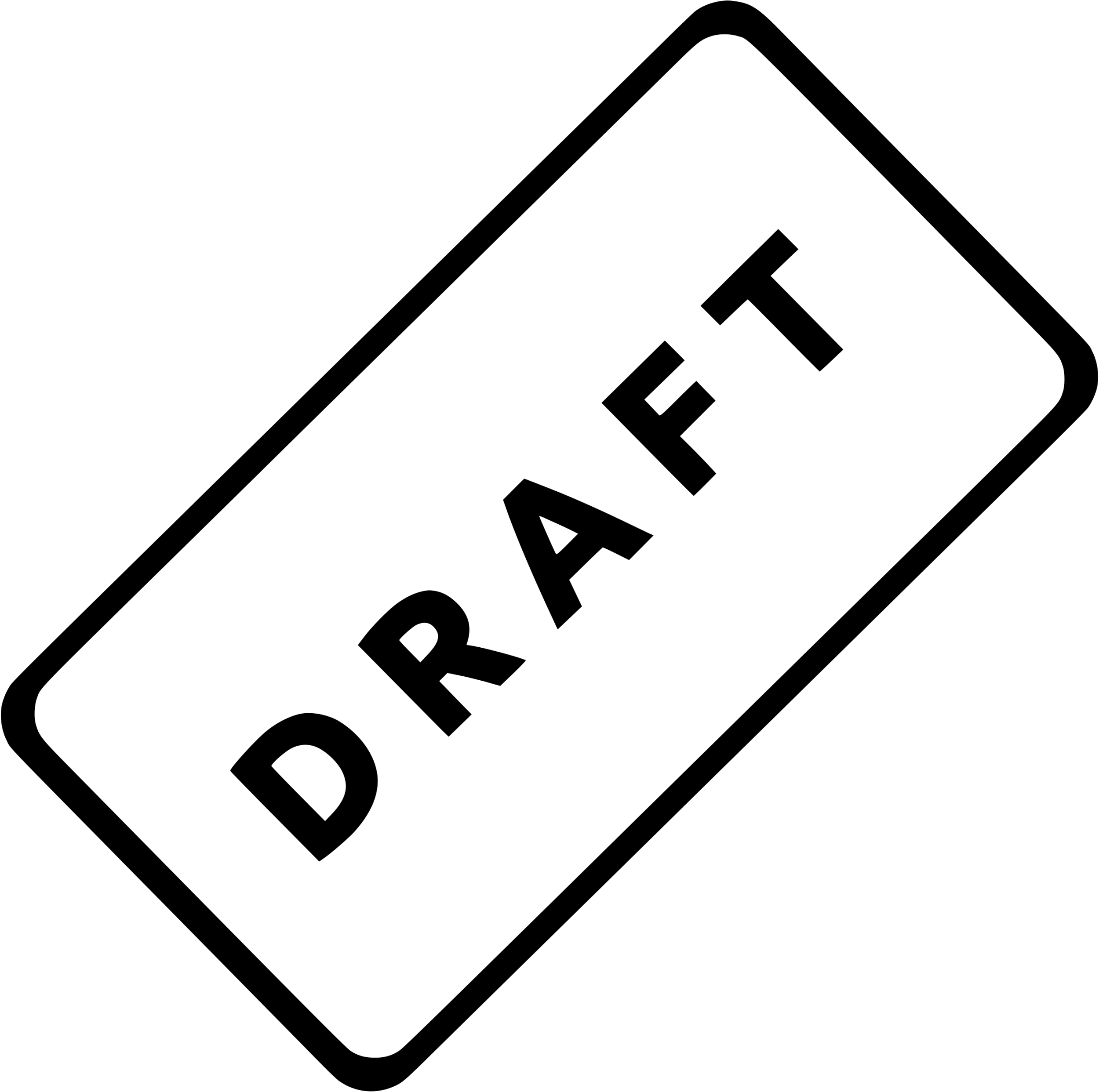What kind of toilet facility do members of your household usually use?  INSTRUCTION: REMIND RESPONDENT YOU ARE TALKING ABOUT THEIR HOUSEHOLD, NOT WHEN THEY’RE OUTSIDE | **Flush or pour flush toilet**   1. Flush to piped sewer system 2. Flush to septic tank 3. Flush to pit latrine 4. Flush to somewhere else 5. Flush don’t know where   **Pit Latrine**   1. Ventilated improved pit latrine 2. Pit latrine with slab 3. Pit latrine without slab/open pit 4. Composting toilet 5. Bucket toilet 6. Hanging toilet/hanging latrine 7. No facility/bush/field   88. Others (specify)  98. Don’t know |
| CAPI INSTRUCTION: Ask AA.18 if response to AA.17 is 01-08 | | |
| AA.18 | Do you share this toilet facility with other households? | 1. No 2. Yes |
| AA.19 | Where is this toilet facility located? | 1. In own dwelling 2. In your yard/plot 3. Elsewhere |
| AA.20 | Where do you usually dispose child (0-3 years)’s stool?  [PROBE: ANYWHER ELSE?]  [MULTI-SELECT] | 1. 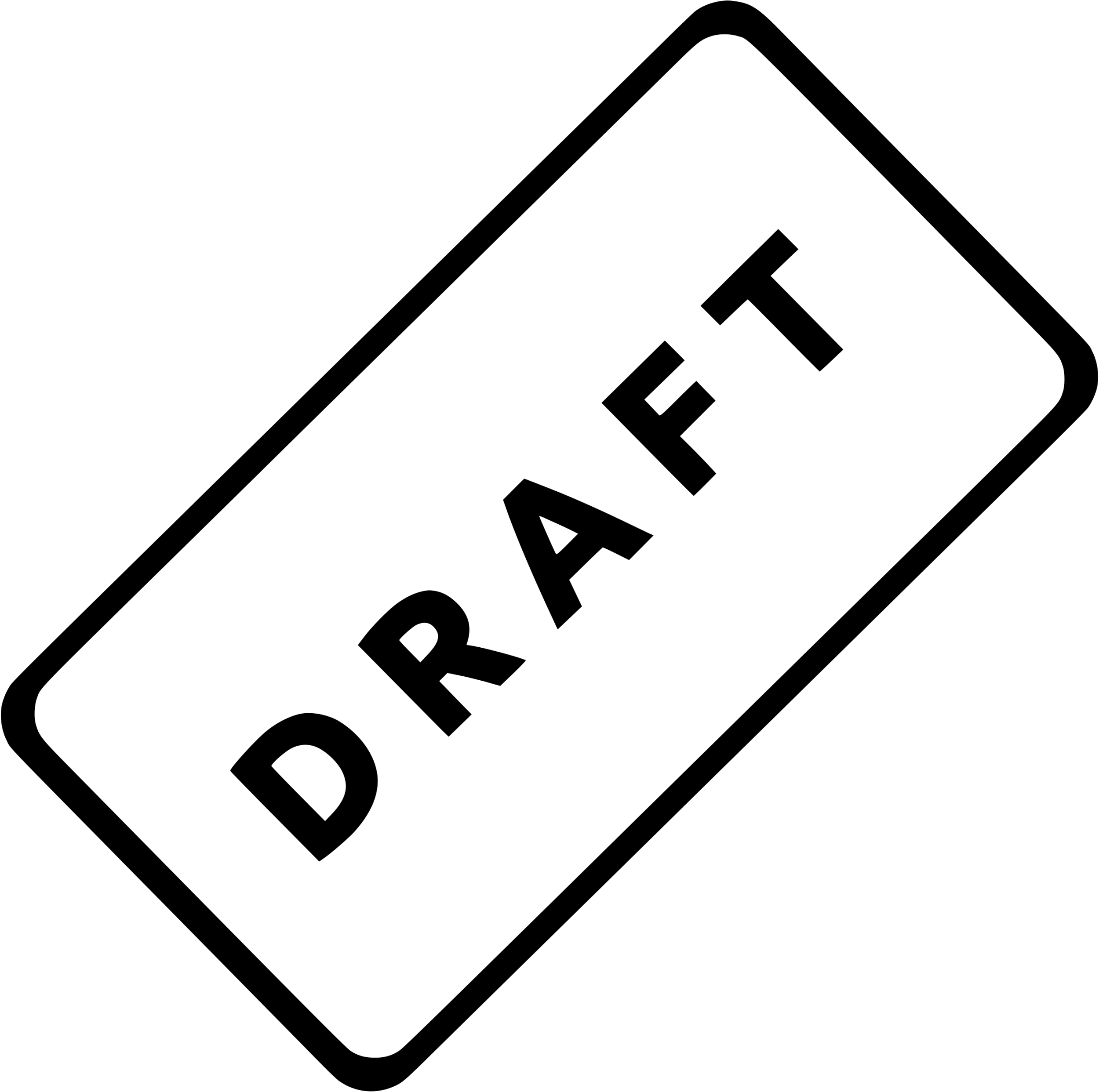Child use toilet or latrine 2. Into toilet or latrine 3. Into drain or ditch 4. Throw into garbage 5. Buried 6. Left in open 7. No child (0-3 years) in the house 8. Others (specify)   98. Don’t know |

| **Access to services (AS)** | | |
| --- | --- | --- |
| **Respondent: Head of household** | | |
| CAPI instruction: Complete this section with the name listed in S.N.1.a of the respondent matrix.  Add Respondent ID __ | | |
| AS.1 | Does any member of this household have a bank account or an account in another financial institution? | 1. No 2. Yes |
| AS.2 | Is any member of your household a member of a savings club? | 1. No 2. Yes   98. Don’t know |
| AS.3 | In the past 12 months, have you or any household member received any money from someone not living in your household at the time of sending? (This means remittance) | 1. No 2. Yes   98. Don’t know |

Module end time XX: XX


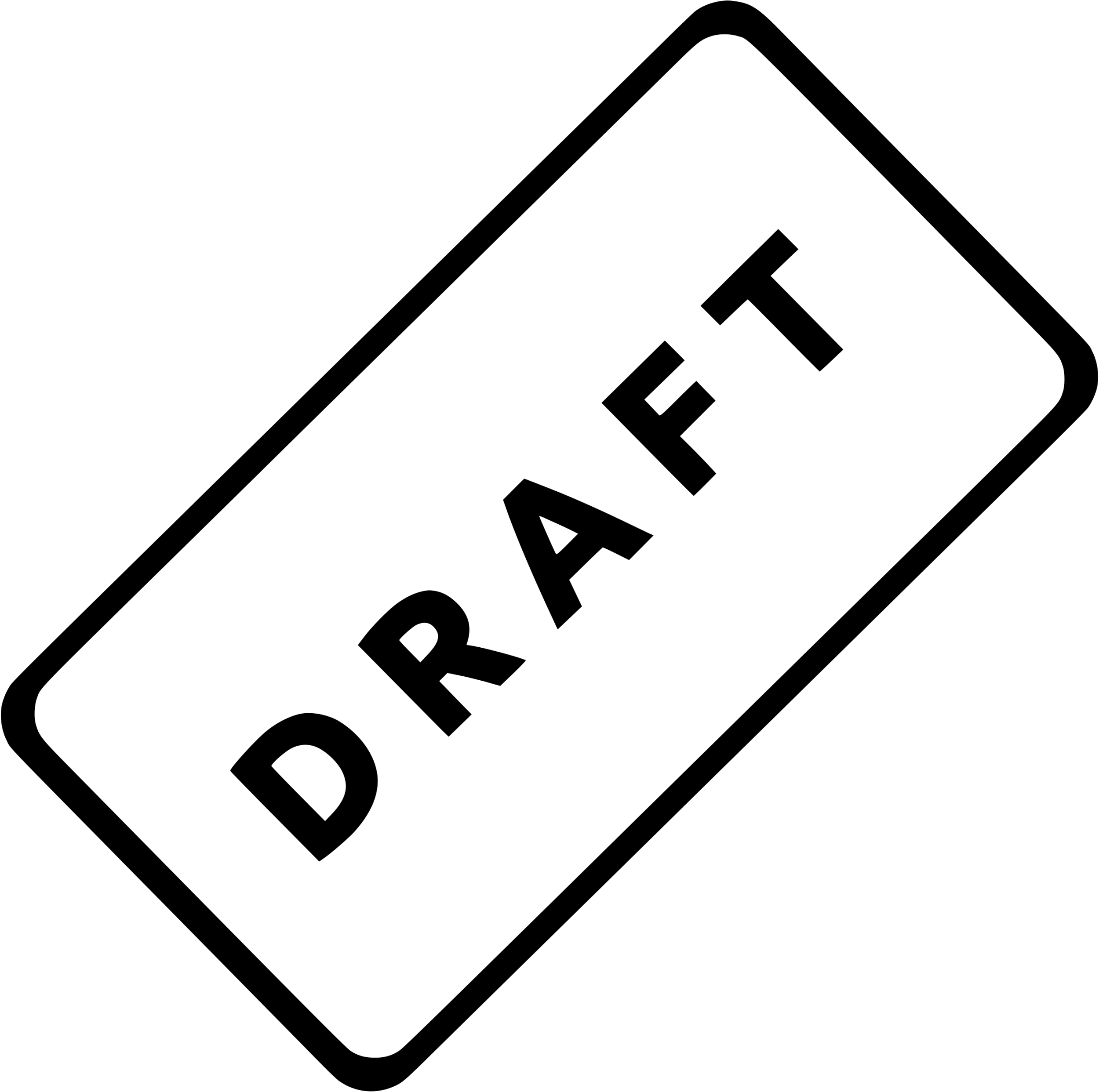
Module start time XX: XX

| **Household food insecurity experience scale (HF)** | | |
| --- | --- | --- |
| **Respondent: Head of household** | | |
| CAPI instruction: Complete this section with the name listed in S.N.1.a of the respondent matrix.  Add Respondent ID __ | | |
| Now, I would like to ask you some questions about food. During the last 12 months, was there a time when _____________? | | |
| **Q.no** | **Q. label** | **Response** |
| HF.1 | You or others in your household were worried you would not have enough food to eat because of a lack of money or other resources | 1. No 2. Yes 3. Refused to answer   98. Don’t know |
| HF.2 | Still thinking about the last 12 MONTHS, was there a time when you or others in your household were unable to eat healthy and nutritious food because of a lack of money or other resources? | 1. No 2. Yes 3. Refused to answer   98. Don’t know |
| HF.3 | You or others in your household ate only a few kinds of foods because of a lack of money or other resources. | 1. No 2. Yes 3. Refused to answer   98. Don’t know |
| HF.4 | You or others in your household had to skip a meal because there was not enough money or other resources to get food? | 1. No 2. Yes 3. Refused to answer   98. Don’t know |
| HF.5 | Still thinking about the last 12 MONTHS, was there a time when you or others in your household ate less than you thought you should because of a lack of money or other resources? | 1. No 2. Yes 3. Refused to answer   98. Don’t know |
| HF.6 | Your household ran out of food because of a lack of money or other resources? | 1. No 2. Yes 3. Refused to answer   98. Don’t know |
| HF.7 | You or others in your household were hungry but did not eat because there was not enough money or other resources for food? | 1. No 2. Yes 3. Refused to answer   98. Don’t know |
| HF.8 | During the last 12 MONTHS, was there a time when you or others in your household went without eating for a whole day because of a lack of money or other resources | 1. No 2. Yes 3. Refused to answer 4. Don’t know |

Module end time XX: XX

Module start time XX: XX

| **Nutrition-sensitive agriculture program receipt (NS)** | | |
| --- | --- | --- |
| **Respondent: Head of household** | | |
| CAPI instruction: Complete this section with the name listed in S.N.1.a of the respondent matrix.  Add Respondent ID __ | | |
| Now, I am going to ask you some questions about support you or your household might have received related to agriculture and livestock. | | |
| **Q.no** | **Q. label** | **Response** |
| NS.1 | In the last 12 months, have you or anyone in your household been involved in growing crops? | 1. No > Skip to NS.2 2. Yes   98. Don’t know > Skip to NS.2 |
| NS1.1 | Have you or anyone in your household received improved seeds for crops (such as: rice, wheat, potato, maize) from an agriculture extension officer or NGO worker? | 1. No > Skip to NS.2 2. Yes   98. Don’t know > Skip to NS.2 |
| NS1.2 | 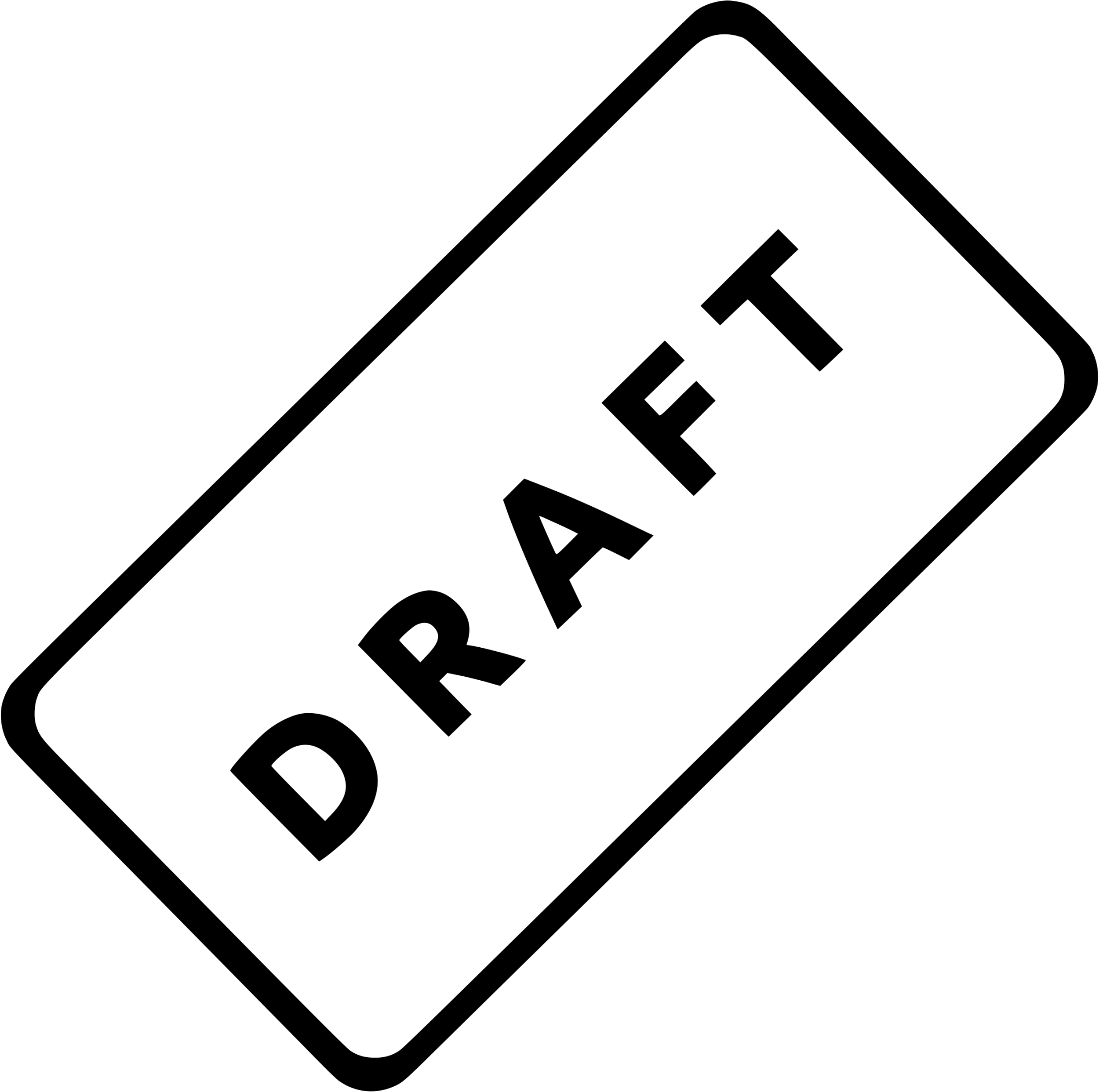With the improved seed received for crops, did you also receive nutrition counseling or training? | 1. No 2. Yes   98. Don’t know |
| NS.2 | In the last 12 months, have you or anyone in your household been involved in growing fruits? | 1. No > Skip to NS.3 2. Yes 3. 98. Don’t know > Skip to NS.3 |
| NS.2.1 | Have you or anyone in your household received sapling for fruits (such as: guava, jackfruit, mango) from an agriculture extension officer or NGO worker? | 1. No > Skip to NS.3 2. Yes 3. 98. Don’t know > Skip to NS.3 |
| NS.2.2 | With the sapling received for fruits, did you also receive nutrition counseling or training? | 1. No 2. Yes   98. Don’t know |
| NS.3 | In the last 12 months, have you or anyone in your household been involved in growing vegetables? | 1. No > Skip to NS.4 2. Yes   98. Don’t know > Skip to NS.4 |
| NS.3.1 | Have you or anyone in your household received seeds for vegetables (such as: gourds, eggplant, spinach) from an agriculture extension officer or NGO worker? | 1. No > Skip to NS.4 2. Yes   98. Don’t know > Skip to NS.4 |
| NS.3.2 | With the seed received for vegetables, did you also receive nutrition counseling or training? | 1. 01. No 2. Yes   98. Don’t know |
| NS.4 | In the last 12 months, have you or anyone in your household been involved in fish or shrimp/crab farming? | 1. 01. No > Skip to NS.5 2. Yes 3. 98. Don’t know > Skip to NS.5 |
| NS.4.1 | Have you or anyone in your household received fish or shrimp (such as: Rohu, Catla, Mriga) from an agriculture extension officer or NGO worker? | 1. No > Skip to NS.5 2. Yes   98. Don’t know > Skip to NS.5 |
| NS.4.2 | With the fish received, did you also receive nutrition counseling or training? | 1. 01. No 2. Yes   98. Don’t know |
| NS.5 | In the last 12 months, have you or anyone in your household been involved in raising poultry such as chicken or ducks? | 1. 01. No > Skip to NS.6 2. Yes   98. Don’t know > Skip to NS.6 |
| NS.5.1 | 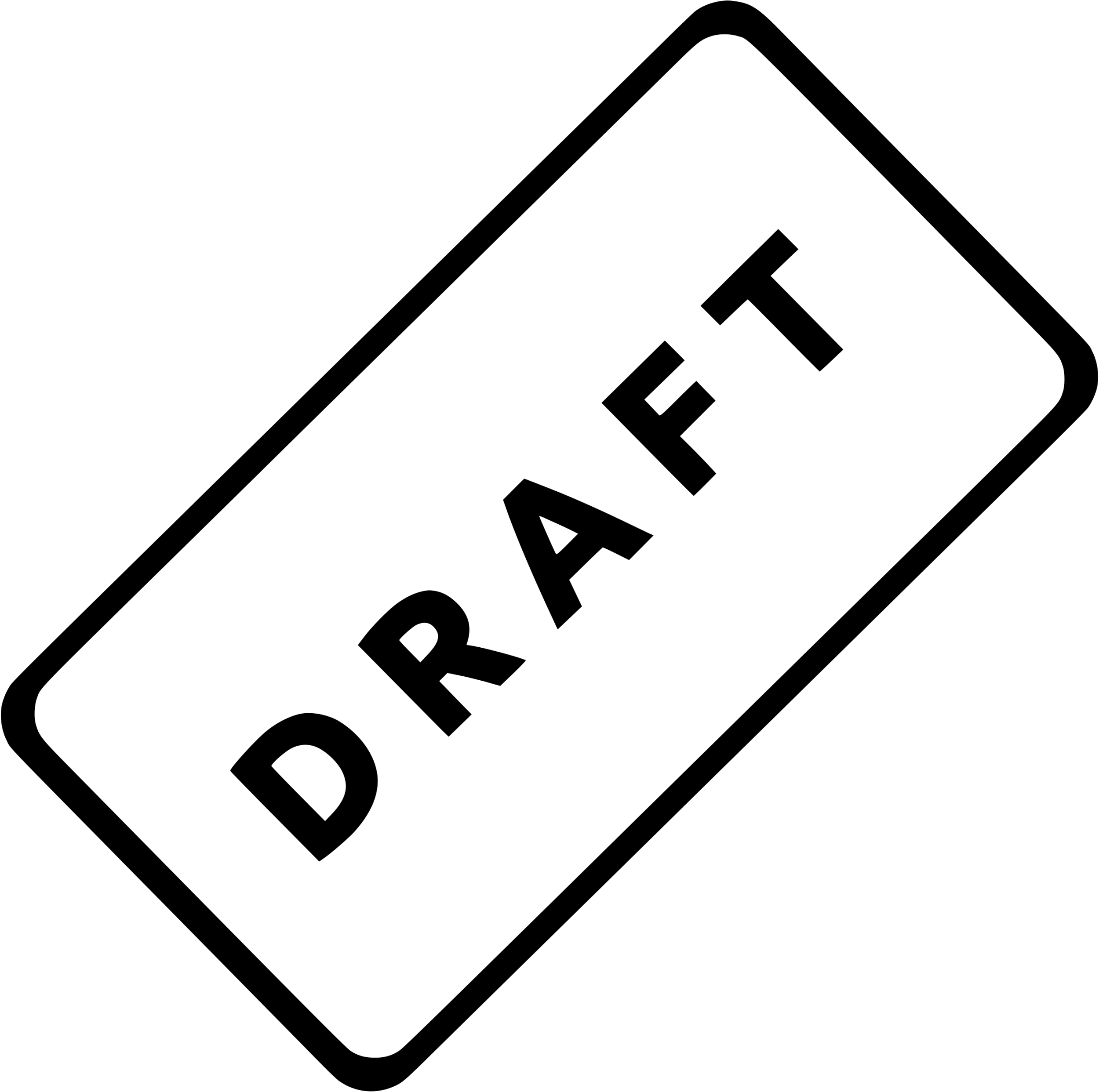Have you or anyone in your household received poultry from an agriculture extension officer or NGO worker? | 1. 01. No > Skip to NS.6 2. Yes   98. Don’t know > Skip to NS.6 |
| NS.5.2 | With the poultry received, did you also receive nutrition counseling or training? | 1. 01. No 2. 02. Yes   98. Don’t know |
| NS.6 | In the last 12 months, have you or anyone in your household been involved in raising goats? | 1. 01. No > Skip to NS.7 2. 02. Yes   98. Don’t know > Skip to NS.7 |
| NS.6.1 | Have you or anyone in your household received goats from an agriculture extension officer or NGO worker? | 1. 01. No > Skip to NS.7 2. Yes   98. Don’t know > Skip to NS.7 |
| NS.6.2 | With the goats received, did you also receive nutrition counseling or training? | 1. 01. No 2. Yes   98. Don’t know |
| NSA.7 | In the last 12 months, have you or anyone in your household been involved in raising cattle such as cows or buffalos? | 1. 01. No > Skip to NS.8 2. Yes   98. Don’t know > Skip to NS.8 |
| NS.7.1 | Have you or anyone in your household received cattle from an agriculture extension officer or NGO worker? | 1. 01. No > Skip to NS.8 2. Yes   98. Don’t know > Skip to NS.8 |
| NS.7.2 | With the cattle received, did you also receive nutrition counseling or training? | 1. 01. No 2. Yes   98. Don’t know |
| NS.8 | In the last 12 months, have you or anyone in your household been involved in growing fruits and vegetables in a home garden i.e. a small garden where your grow fruits and vegetables? | 1. 01. No > Skip to NS.9 2. Yes   98. Don’t know > Skip to NS.9 |
| NS.8.1 | Have you or anyone in your household received training or other support from an agriculture extension officer or NGO worker for your home garden? | 01. No > Skip to NS.9  02. Yes  98. Don’t know > Skip to NS.9 |
| NS.8.2 | With the inputs received for your home garden, did you also receive nutrition counseling or training? | 1. 01. No   02. Yes  98. Don’t know |
| NS.9 | In the past 12 months, how often did you meet with an agriculture extension officer or agriculture NGO worker? | 1. Never 2. Once a week 3. Once or more a month   88. Other (specify)  98. Don’t know |

Module end time XX: XX

| Module start time XX: XX | | | | | |
| --- | --- | --- | --- | --- | --- |
| **Nutrition sensitive social protection programs - CASH (SPC)** | | | | | |
| **Respondent: Head of household** | | | | | |
| Now, I would like to ask you about various external cash assistance programs provided to households and their members. By external assistance, I mean, support that comes from government or non-governmental organizations such as religious, charitable or community organizations. This excludes the support of the family, other relatives, friends, or neighbors. | | | | | |
| SPC.1 | In the past 12 months, has any member of your household received any cash or monetary assistance from the government or any other non-governmental organizations?  [SINGLE SELECT] | 1. No >> *skip to section SPF.1* 2. Yes 3. 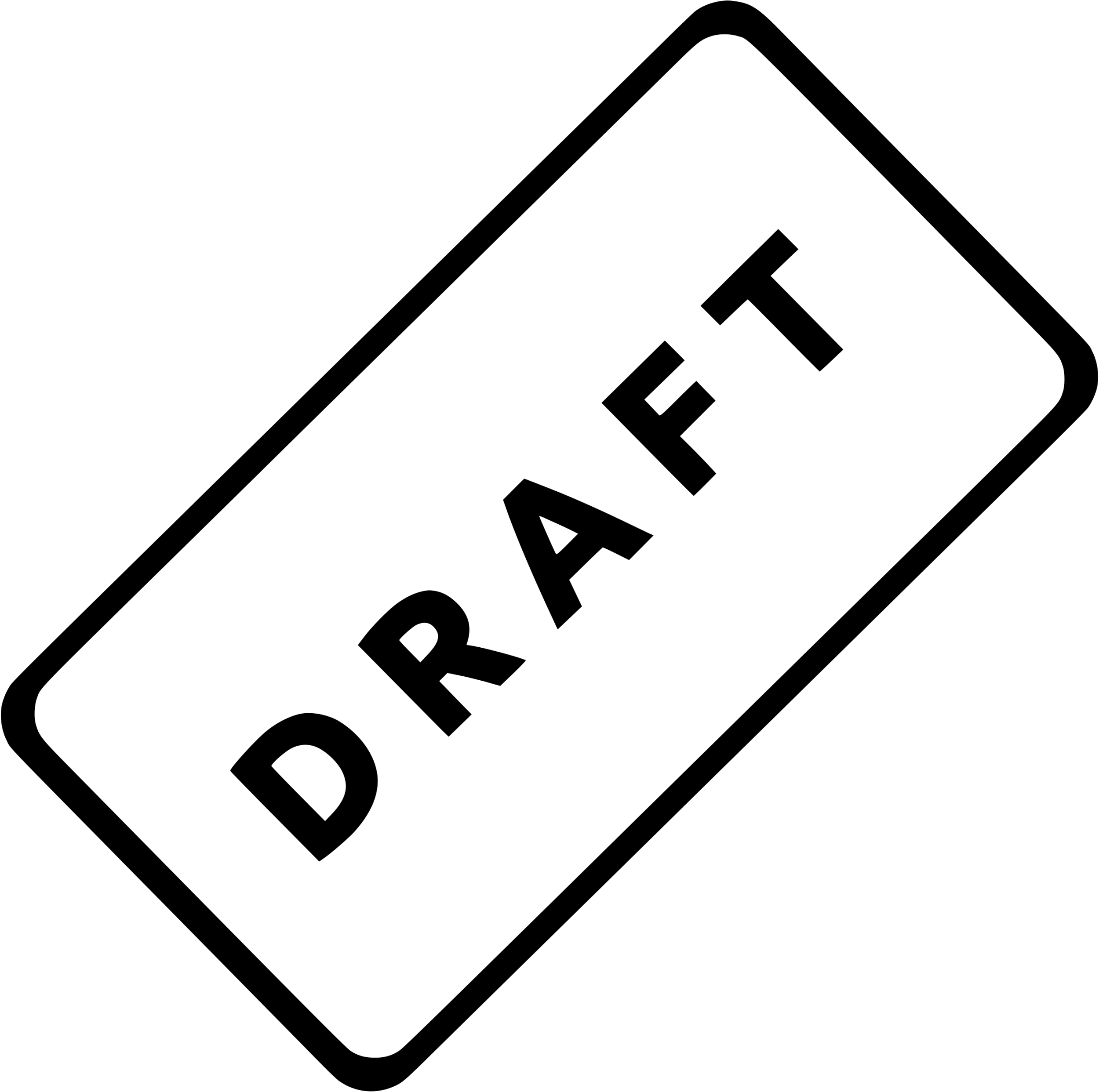Don’t know>> *skip to section SPF.1* | | | |
| SPC.2 | In the past 12 months, who in your household received cash or monetary assistance from the government or any other non-governmental organizations?  Record member ID from HR.1  Entire household = 96  Don’t’ know = 98  [SINGLE SELECT] | Mem 1 | Mem 2 | Mem 3 | Mem 4 |
|  |  | Mem ID | Mem ID | Mem ID | Mem ID |
|  |  |  |  |  |  |
| SPC.3 | With the cash or monetary assistance that [INSERT NAME FROM SPC.2] received, did they also receive the following?  **[read aloud**]   1. Nutrition or health counseling 2. Told to go to a health facility to receive health or nutrition services 3. Tablets to treat intestinal worms 4. Iron ablets or other nutrient supplements 5. Food with extra nutrients added to it to benefit health   88. Other (specify)  [MULTI-SELECT] | 1. No 2. Yes 3. Don’t know | 1. No 2. Yes 3. Don’t know | 1. No 2. Yes   98. Don’t know | 1. No 2. Yes 3. Don’t know |
| 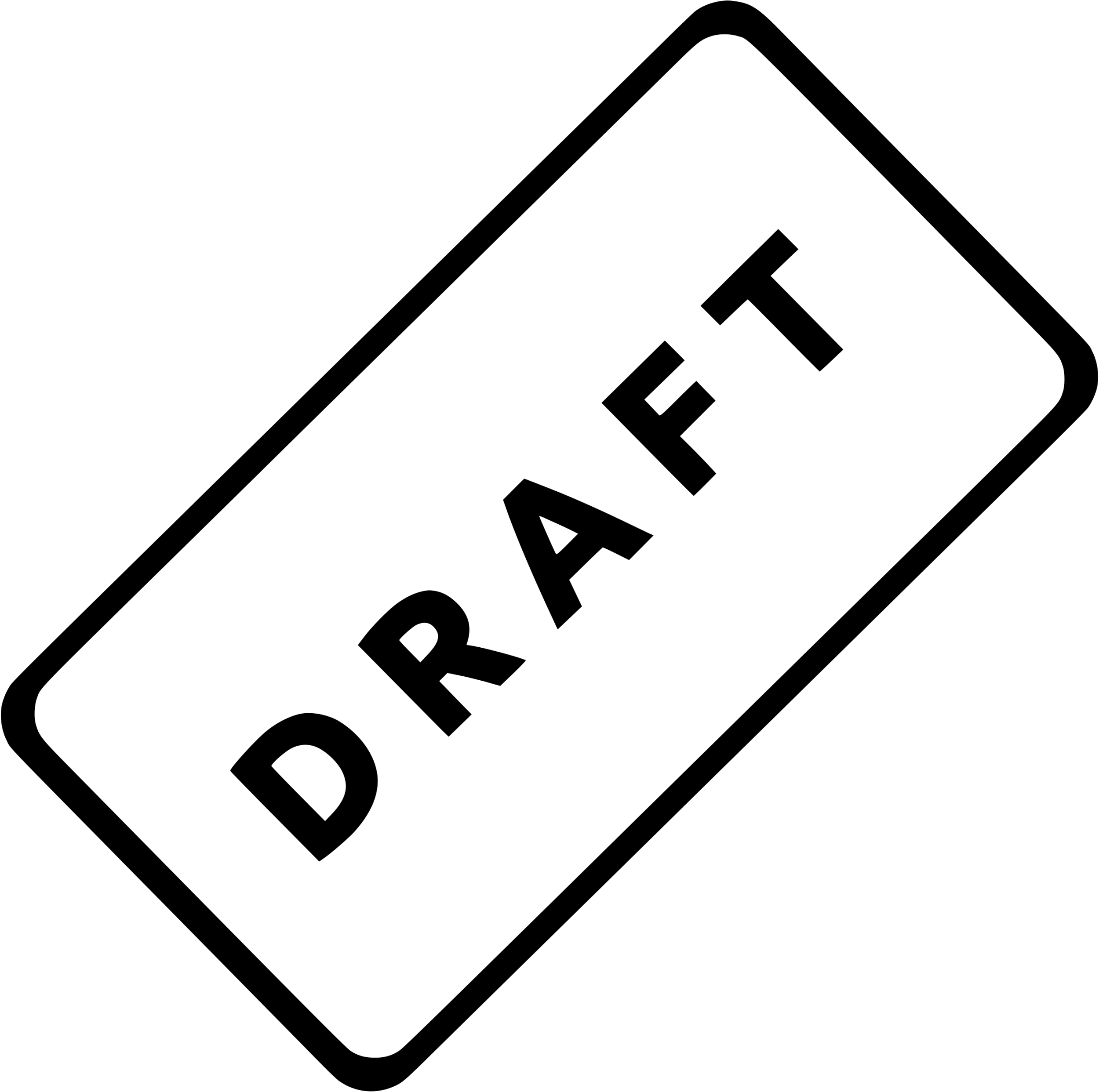SPC.4 | To receive the cash or monetary assistance, did [INSERT NAME FROM SPC.2] or anyone in your household have to do any specific activity such as attend a meeting, take a child to the health facility or anything else? If so, what was it? | 1. No 2. Yes, (specify) _________ | 1. No 2. Yes, (specify) ___________ | 1. No   02. Yes, (specify) _____________ | 1. No   02. Yes, (specify) ______________ |
| SPC.5 | When was the last time [INSERT NAME FROM SPC.2] received cash or monetary assistance from the government or any other non-governmental organizations? | [MM/YYYY] | [MM/YYYY] | [MM/YYYY] | [MM/YYYY] |
| SPC.6 | How often did [INSERT NAME FROM SPC.2] receive cash or monetary assistance from the government or any other non-governmental organizations?  [SINGLE SELECT] | 1. Daily 2. Few times a week but not daily 3. Weekly 4. Monthly 5. Annually 6. Other (specify) 7. Don’t know | 1. Daily 2. Few times a week but not daily 3. Weekly 4. Monthly 5. Annually 6. Other (specify) 7. Don’t know | 1. Daily 2. Few times a week but not daily 3. Weekly 4. Monthly 5. Annually 6. Other (specify) 7. Don’t know | 1. Daily 2. Few times a week but not daily 3. Weekly 4. Monthly 5. Annually 6. Other (specify) 7. Don’t know |

Module end time XX: XX

Module start time XX: XX

| **Nutrition sensitive social protection programs - FOOD (SPF)** | | | | | |
| --- | --- | --- | --- | --- | --- |
| **Respondent: Head of household** | | | | | |
| Now, I would like to ask you about various external food assistance programs provided to households and their members. By external assistance, I mean support that comes from government or non-governmental organizations such as religious, charitable or community organizations. This excludes the support of the family, other relatives, friends, or neighbors. | | | | | |
| SPF.1 | In the past 12 months, has any member of your household received any free food or subsidized food from the government or any other non-governmental organizations?  [SINGLE SELECT] | 1. No >> *skip to section SPI.1* 2. Yes 3. Don’t know>> *skip to section SPI.1* | | | |
| SPF.2 | In the past 12 months, who in your household received free food or subsidized food from the government or any other non-governmental organizations?  Record member ID from HR.1  Entire household = 96  Don’t’ know = 98  [SINGLE SELECT] | Mem 1 | Mem 2 | Mem 3 | Mem 4 |
|  |  | Mem ID | Mem ID | Mem ID | Mem ID |
|  |  |  |  |  |  |
| SPF.3 | With the free food or subsidized food that [INSERT NAME FROM SPF.2] received, did they also receive the following?  [READ ALOUD]  **[read aloud**]   1. Nutrition or health counseling 2. Told to go to a health facility to receive health or nutrition services 3. Tablets to treat intestinal worms 4. 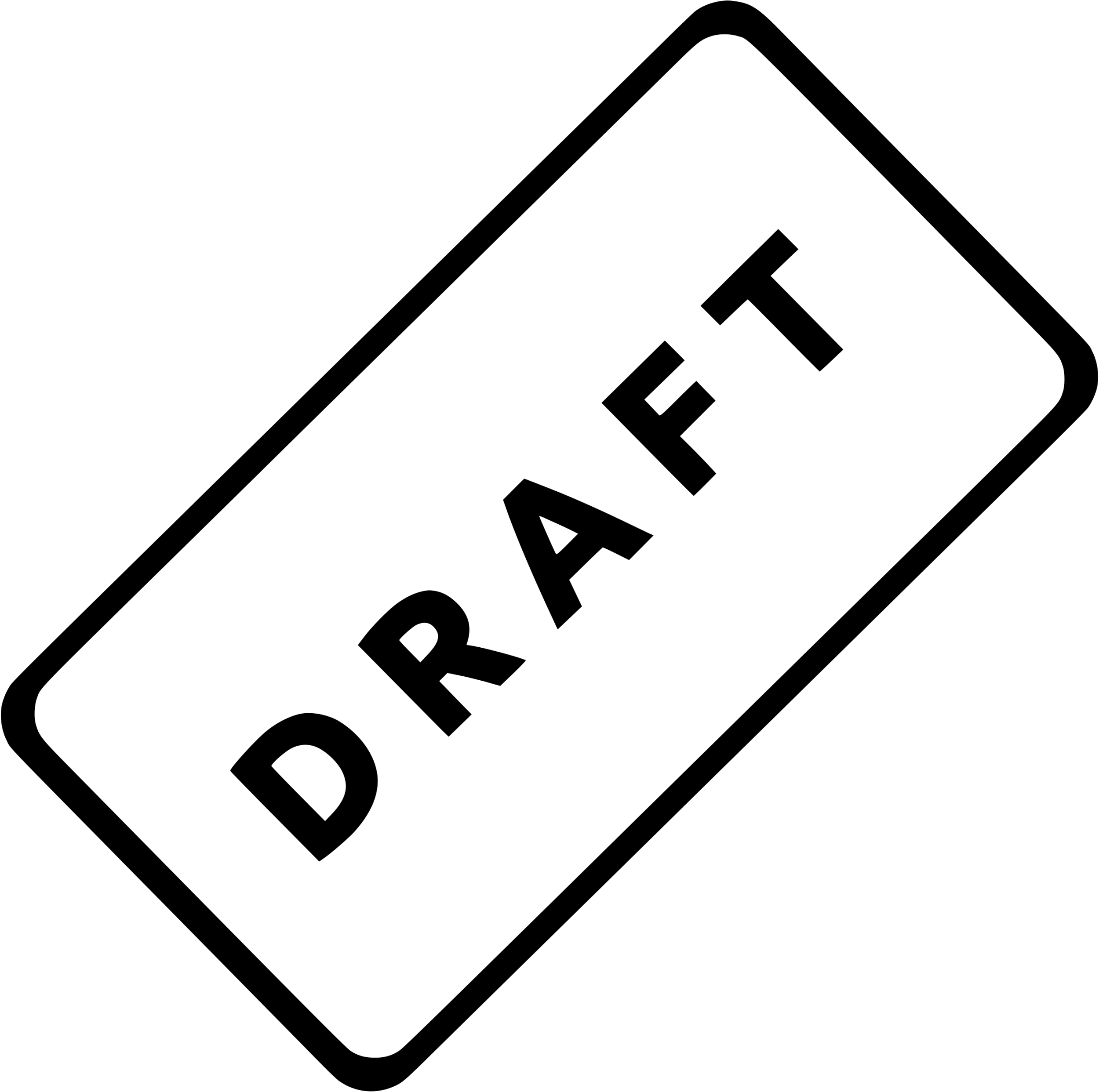Iron tablets or other nutrient supplements 5. Food with extra nutrients added to it to benefit health   88. Other (specify)  [MULTI-SELECT] | 1. 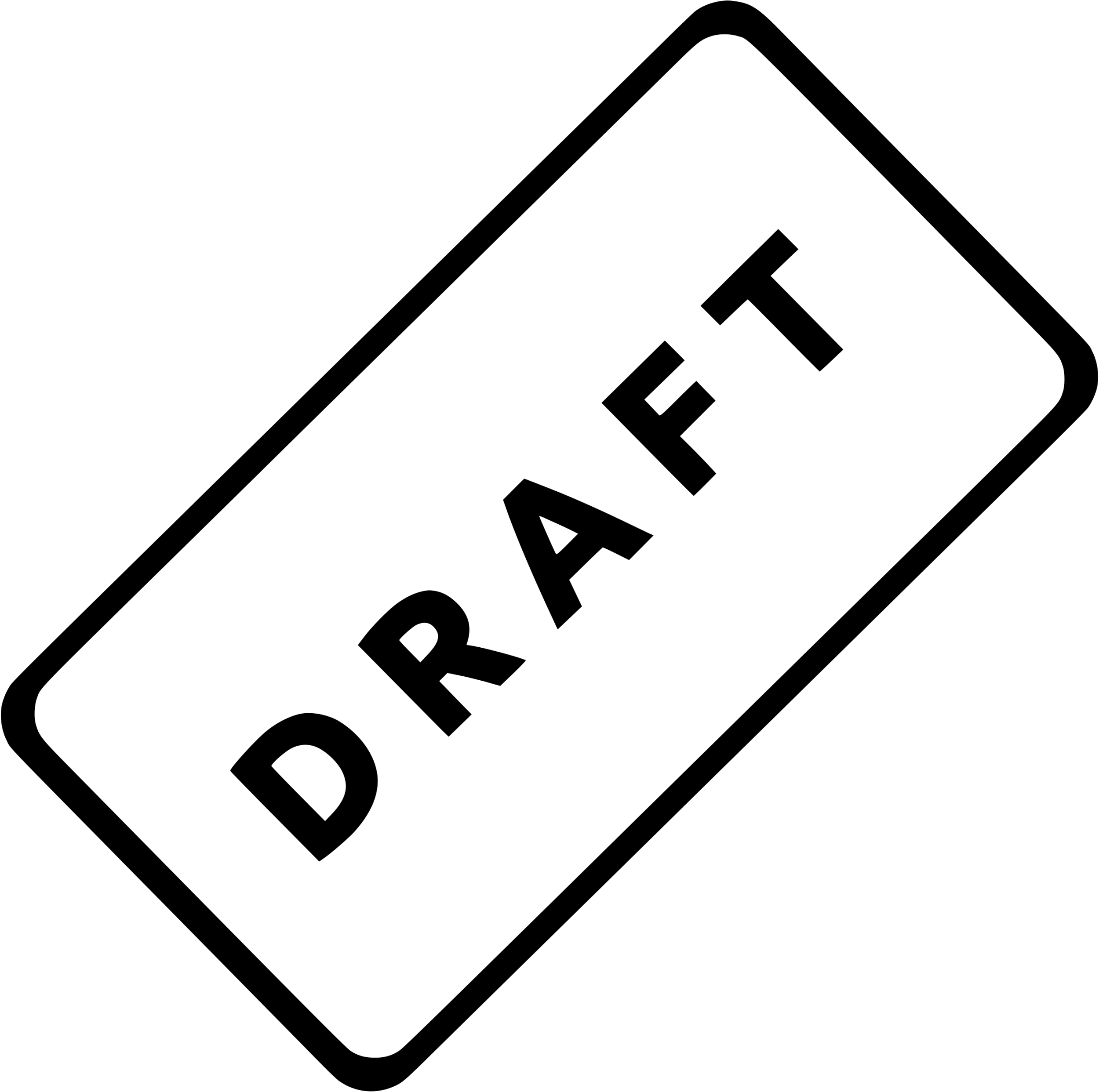No 2. Yes 3. Don’t know | 1. No 2. Yes 3. Don’t know | 1. No 2. Yes 3. Don’t know | 1. Yes 2. No 3. Don’t know |
| SPF.4 | To receive the free food or subsidized food, did [INSERT NAME FROM SPF.2] or anyone in your household have to do any specific activity such as attend a meeting, take a child to the health facility or anything else? If so, what was it? | 1. No 2. Yes, (specify)   ___________ | 1. No   02. Yes,(specify) _____________ | 1. No 2. Yes, (specify)   ___________ | 1. No 2. Yes, (specify)   ___________ |
| SPF.5 | When was the last time [INSERT NAME FROM SPF.2] received free food or subsidized food from the government or any other non-governmental organization? | [MM/YYYY] | [MM/YYYY] | [MM/YYYY] | [MM/YYYY] |
| SPF.6 | How often did INSERT NAME FROM SPF.2] receive free food or subsidized food from the government or any other non-governmental organization?  [SINGLE SELECT] | 1. Daily 2. Few times a week but not daily 3. Weekly 4. Monthly 5. Annually 6. Other (specify) 7. Don’t know | 1. Daily 2. Few times a week but not daily 3. Weekly 4. Monthly 5. Annually 6. Other (specify) 7. Don’t know | 1. Daily 2. Few times a week but not daily 3. Weekly 4. Monthly 5. Annually 6. Other (specify) 7. Don’t know | 1. Daily 2. Few times a week but not daily 3. Weekly 4. Monthly 5. Annually 6. Other (specify) 7. Don’t know |

Module end time XX: XX

Module start time XX: XX

| **Nutrition sensitive social protection programs – IN-KIND (SPI)** | | | | | |
| --- | --- | --- | --- | --- | --- |
| **Respondent: Head of household** | | | | | |
| Now, I would like to ask you about various external in-kind assistance (for example: improved seed) provided to households and their members. By external assistance, I mean support that comes from government or non-governmental organizations such as religious, charitable or community organizations. This excludes the support of the family, other relatives, friends, or neighbors. | | | | | |
| SPI.1 | In the past 12 months, has any member of your household received any in-kind from the government or any other non-governmental organizations?  [SINGLE SELECT] | 1. No >> *skip to section SFY.1* 2. Yes, specify __________________ 3. Don’t know>> *skip to section SFY.1* | | | |
| SPI.2 | 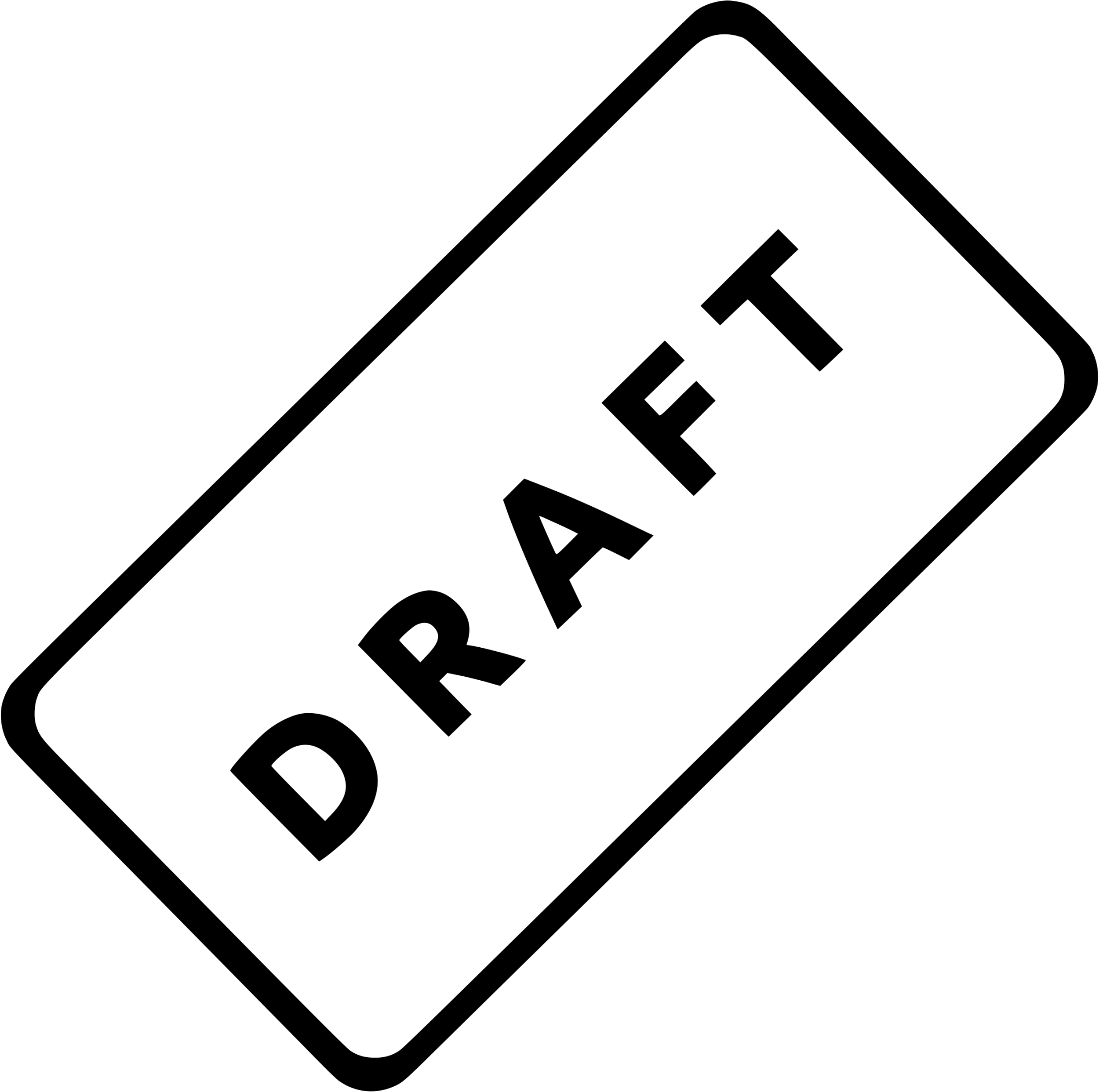In the past 12 months, who in your household received in-kind transfer from the government or any other non-governmental organizations?  Record member ID from HR.1  Entire household = 96  Don’t’ know = 98  [SINGLE SELECT] | Mem 1 | Mem 2 | Mem 3 | Mem 4 |
|  |  | Mem ID | Mem ID | Mem ID | Mem ID |
|  |  |  |  |  |  |
| SPI.3 | With the in-kind transfer that [INSERT NAME FROM SPI.2] received, did they also receive the following?  [READ ALOUD]  **[read aloud**]   1. Nutrition or health counseling 2. Told to go to a health facility to receive health or nutrition services 3. Tablets to treat intestinal worms 4. Iron tablets or other nutrient supplements 5. Food with extra nutrients added to it to benefit health 6. Other (specify)   [MULTI-SELECT] | 1. No 2. Yes 3. Don’t know | 1. No 2. Yes 3. Don’t know | 1. No 2. Yes 3. Don’t know | 1. Yes 2. No 3. Don’t know |
| SPI.4 | To receive the in-kind transfer, did [INSERT NAME FROM SPI.2] or anyone in your household have to do any specific activity such as attend a meeting, take a child to the health facility or anything else? If so, what was it? | 1. No 2. Yes, (specify)   ___________ | 1. No   02. Yes,(specify) _____________ | 1. No 2. Yes, (specify)   ___________ | 1. No 2. Yes, (specify)   ___________ |
| SPI.5 | When was the last time [INSERT NAME FROM SPI.2] received in-kind transfer from the government or any other non-governmental organization? | [MM/YYYY] | [MM/YYYY] | 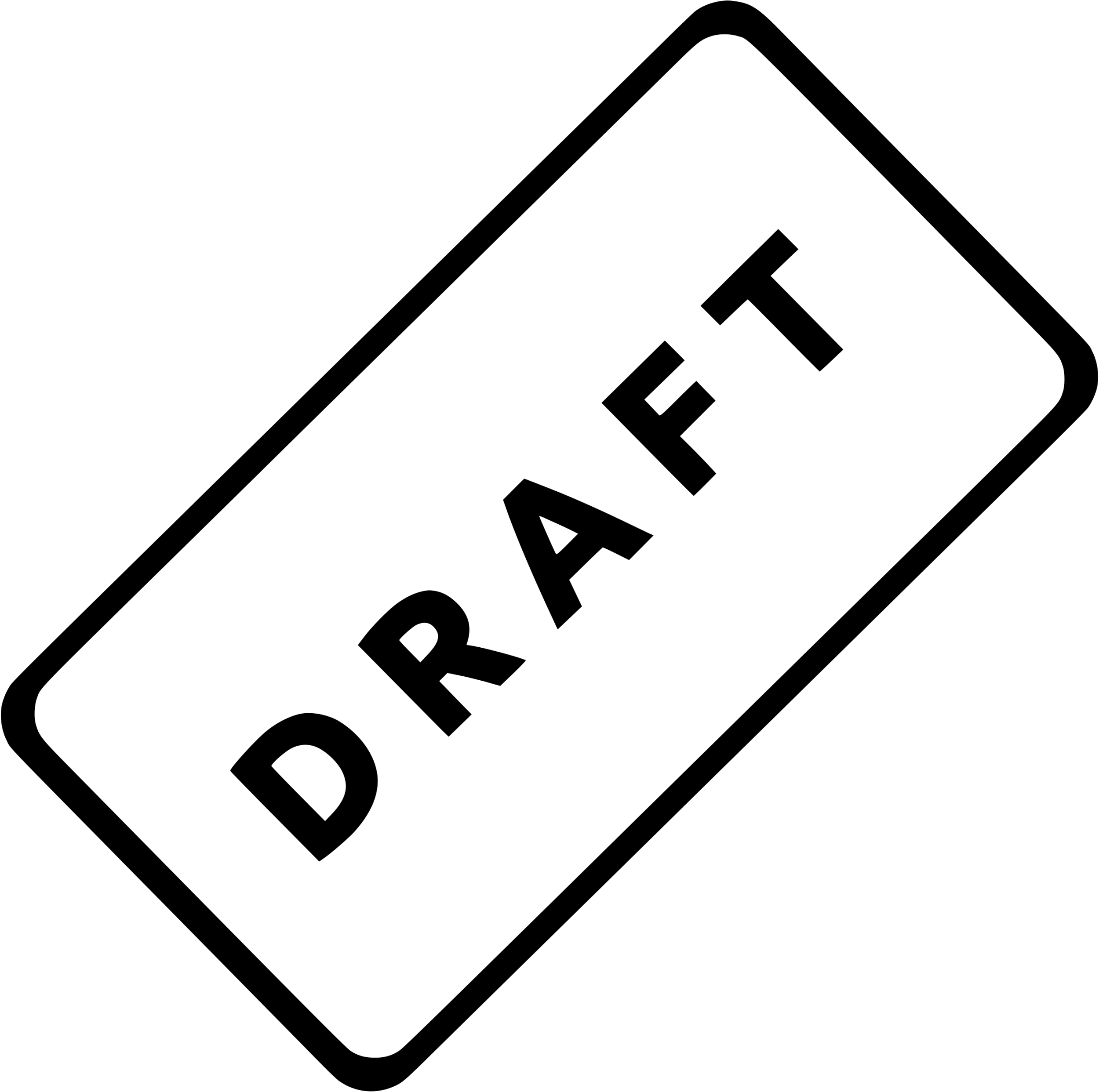[MM/YYYY] | [MM/YYYY] |
| SPI.6 | How often did INSERT NAME FROM SPI.2] receive the in-kind transfer from the government or any other non-governmental organization?  [SINGLE SELECT] | 1. Daily 2. Few times a week but not daily 3. Weekly 4. Monthly 5. Annually 6. Other (specify) 7. Don’t know | 1. Daily 2. Few times a week but not daily 3. Weekly 4. Monthly 5. Annually 6. Other (specify) 7. Don’t know | 1. Daily 2. Few times a week but not daily 3. Weekly 4. Monthly 5. Annually 6. Other (specify) 7. Don’t know | 1. Daily 2. Few times a week but not daily 3. Weekly 4. Monthly 5. Annually 6. Other (specify) 7. Don’t know |

Module end time XX: XX

Module start time XX: XX

| **School Feeding (SFY)** | | | | | |
| --- | --- | --- | --- | --- | --- |
| **Respondent- Head of household** | | | | | |
| Now, I would like to ask you about the meal that your child receives from school. | | | | | |
| SFY.1 | In the past 12 months, has any member of your household received free food from school? | 1. No>>skip to FV 2. Yes 3. Don’t know>>skip to FV | | | |
| SFY.2 | Which child in your household received free food from school?  Record member ID from HR.1  Don’t’ know = 98  [SINGLE SELECT] | Mem 1 | Mem 2 | Mem 3 | Mem 4 |
|  |  | Mem ID | Mem ID | Mem ID | Mem ID |
|  |  |  |  |  | 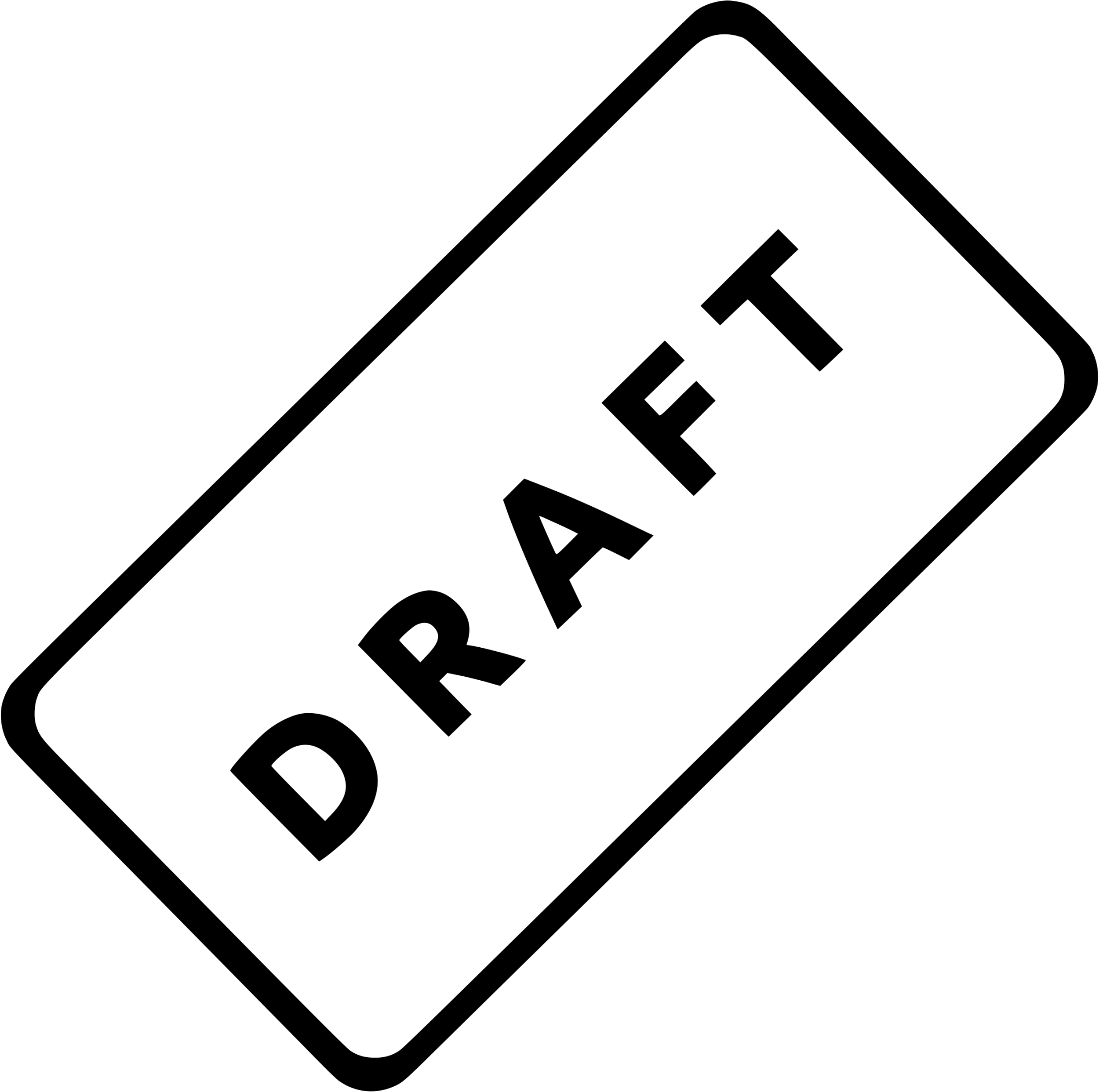 |
| SFY.3 | With the free food that [INSERT NAME FROM SFY.2] received, did they also receive the following?  **READ ALOUD**   1. Nutrition or health education 2. Told to go to a health facility to receive health or nutrition services 3. Tablets to treat intestinal worms 4. Iron tablets or other nutrient supplements 5. Food with extra nutrients added to it to benefit health 6. Other (specify)   [MULTI SELECT] | 1. No 2. Yes 3. Don’t know | 1. No 2. Yes 3. Don’t know | 1. No 2. Yes 3. Don’t know | 1. No 2. Yes 3. Don’t know |
| SFY.4 | When was the last time [INSERT NAME FROM SFY.2] received free food from the school? | [MM/YYYY] | [MM/YYYY] | [MM/YYYY] | [MM/YYYY] |
| SFY.5 | How often did INSERT NAME FROM SFY.2] receive free food from the school?  [SINGLE SELECT] | 1. Daily 2. Few times a week but not daily 3. Weekly 4. Monthly 5. Annually 6. Other (specify) 7. Don’t know | 1. Daily 2. Few times a week but not daily 3. Weekly 4. Monthly 5. Annually 6. Other (specify) 7. Don’t know | 1. Daily 2. Few times a week but not daily 3. Weekly 4. Monthly 5. Annually 6. Other (specify) 7. Don’t know | 1. Daily 2. Few times a week but not daily 3. Weekly 4. Monthly 5. Annually 6. Other (specify) 7. Don’t know |

Module end time XX: XXModule start time XX: XX

| **Food vehicle fortification coverage (FV)** | | |
| --- | --- | --- |
| **Respondent- Person responsible for purchasing food** | | |
| CAPI instruction: Complete this section with the name listed in S.N.1.b of the respondent matrix. Add Respondent ID __ | | |
| Food vehicles:   1. Oil 2. Wheat flour 3. Salt 4. Rice   CAPI instruction: Repeat the module for each food vehicle listed above | | |
| Now I’m going to ask you some questions about food items including | | |
| **Q.no** | **Q. label** | **Response** |
| Skip FV.0.wf in food vehicle is oil, salt, or rice | | |
| FV.0_wf | Does your household purchase foods made from wheat flour, such as bread or [insert other locally available foods made from wheat flour]? | 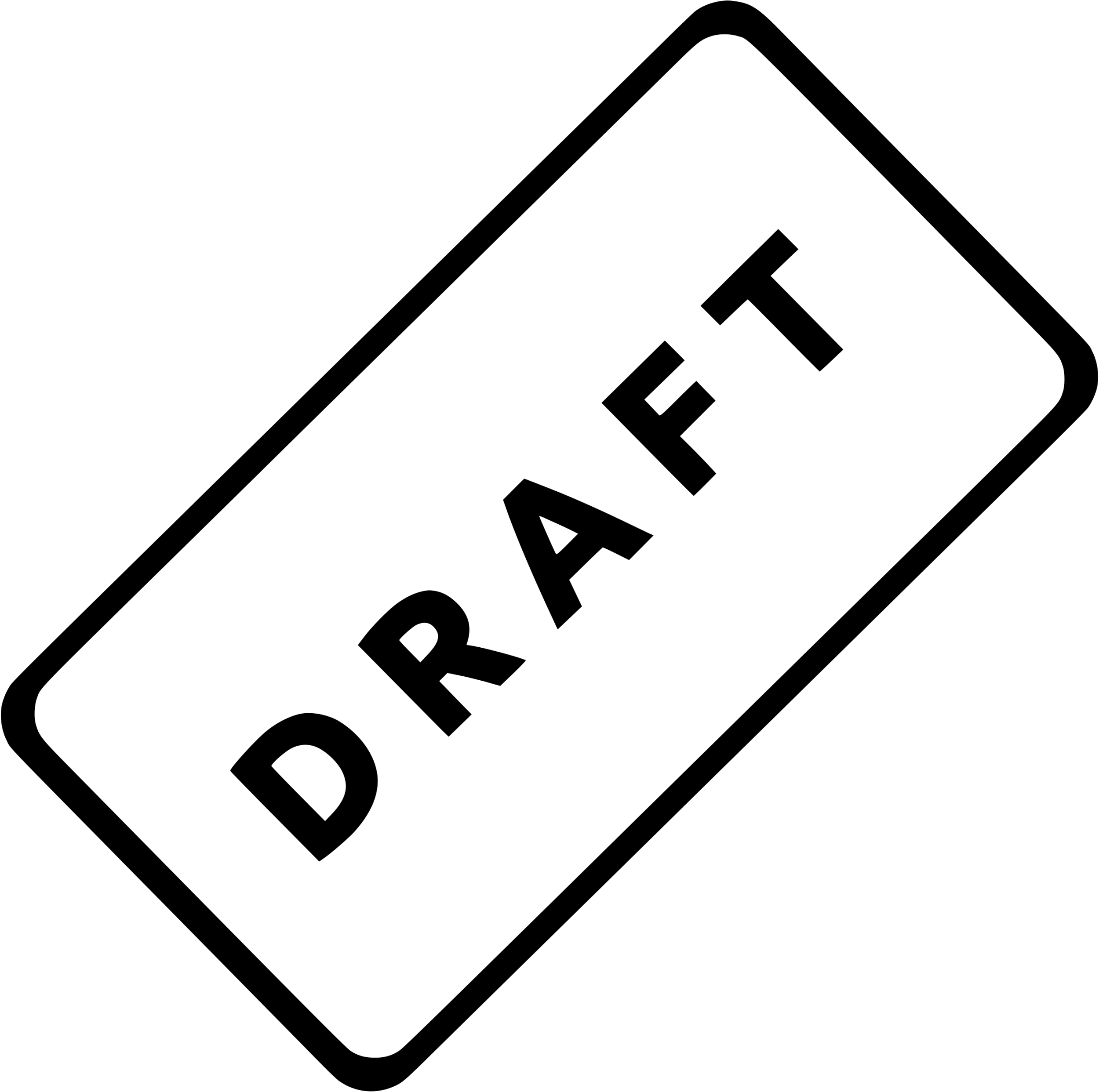 |
| FV.1 | Does your household consume [INSERT FOOD VEHICLE] at home? [SINGLE SELECT] | 1. No>> skip to next food vehicle 2. Yes |
| FV.2 | What are the main types of [INSERT FOOD VEHICLE] that your household uses on most days?  [ANYTHING ELSE?]  [MULTI SELECT] | See code list below |
| FV.2a | Last time your household got [INSERT FOOD VEHICLE], what type was it? | See code list below |
| FV.3 | The last time your household got [PREFILL WITH RESPONSE FROM FV.2a], where did your household get it from? | 1. Purchased at supermarket (large shop; can enter shop and select from the shelf) 2. Purchased at retail shop or kiosk (small shop; may be served) 3. Purchased at market stall in open-air market 4. Purchased at government store 5. Received from food aid/safety net program (examples: Khadda Bandho etc.) 6. Received from relative or friend who purchased it 7. Received from relative or friend who produced it themselves >> Skip to next food vehicle 8. Made/produced/grew it at home >> Skip to next food vehicle 9. Other (specify) 10. Don’t know/Don’t remember |
| FV.4 | The last time your household got [PREFILL WITH RESPONSE FROM FV.2a], did you get it in its original package (the package or container that the company produced)? | 1. No 2. Yes 3. Don’t know |
| FV.5 | The last time your household got [PREFILL WITH RESPONSE FROM FV.2a], what was the brand name or company name on the packaging or container?  CAPI instruction: Recommended to add brands in alphabetical order so that it is easier for the interviewer to find and record the reported brand.  INSTRUCTION: DO NOT LET THE RESPONDENT LOOK FOR THE PACKAGE; DO NOT GIVE EXAMPLES OF BRAND NAMES | 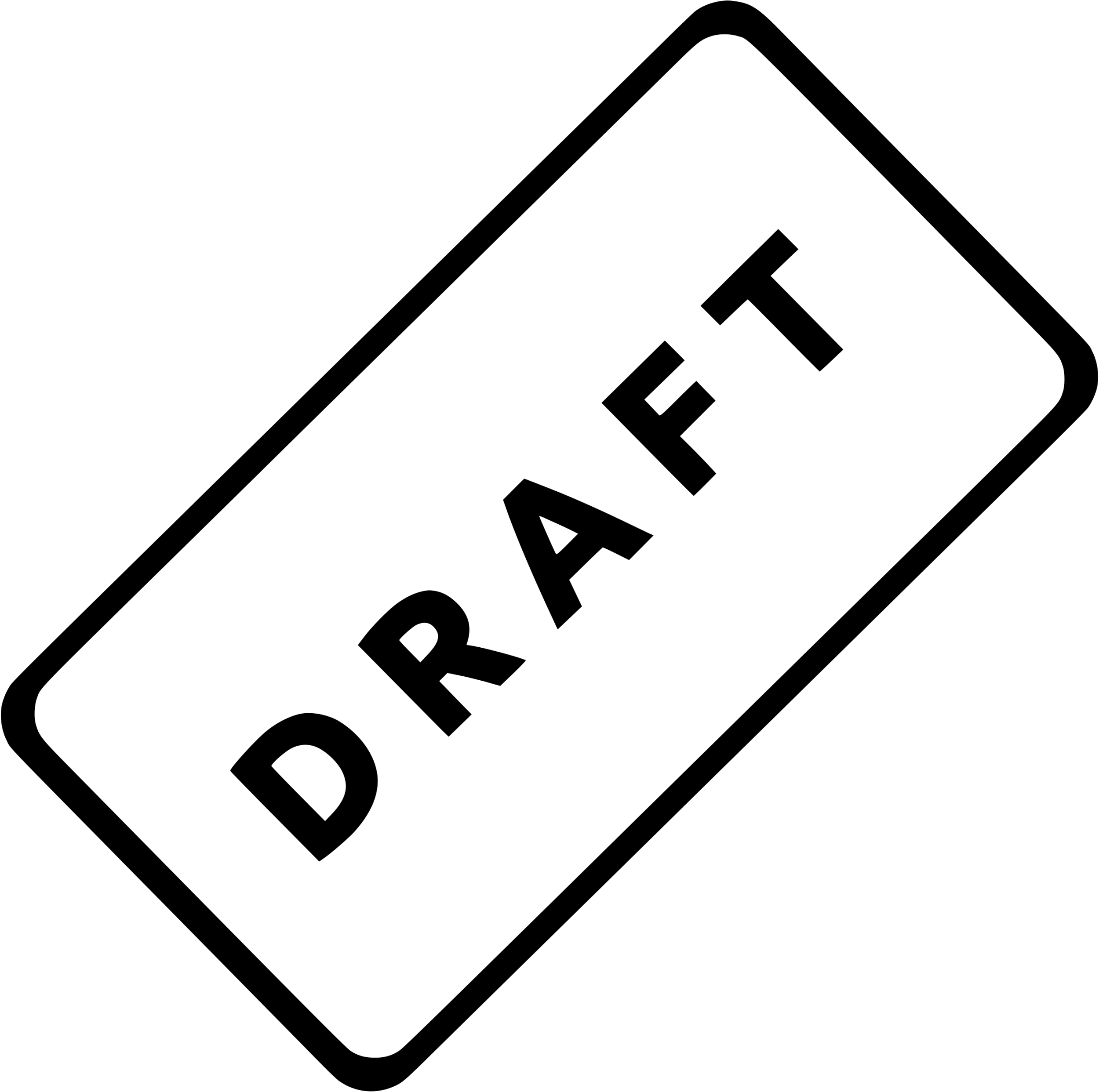See code list below |
| FV.5.1 | Was the response to FV.5 based on recall or on observation of the packaging or container? | 1. Recall 2. Observation |
| FV.6 | Do you have this [PREFILL WITH RESPONSE FROM FV.2a] in your home now? | 1. No>> skip to next food vehicle 2. Yes |
| FV.7 | ASK TO SEE THE [PREFILL WITH RESPONSE FROM FV2a] PACKAGE AND LOOK FOR FORTIFICATION LOGO OR WORDS SUCH AS IODIZED OR FORTIFIED. | 1. [PREFILL WITH RESPONSE FROM FV2a] is in its original package and logo and/or fortification statement were observed 2. [PREFILL WITH RESPONSE FROM FV2a] is in its original package and logo or fortification statement were NOT observed 3. [PREFILL WITH RESPONSE FROM FV2a] is not in its original package >> Skip to FV9 |
| FV.8 | SELECT THE BRAND OBSERVED ON THE ORIGINAL PACKAGE | See code list below |
| FV.9 | I would like to check whether the salt used in your household is iodized. May I have a sample of the salt used to cook meals in your household? | 1. Iodine present 2. No iodine 3. Household uses salt but there is no salt in the household 4. Household does not use salt 5. Salt not tested (specify reason) |

Module end time XX: XX

Code list for FV.2, FV.2a, FV.5, FV.8

| **Food vehicle** | **Food vehicle type** | **Brand** |
| --- | --- | --- |
| 01. Oil | 01. Canola Oil | 01. Heart Light |
| 01. Oil | 01. Canola Oil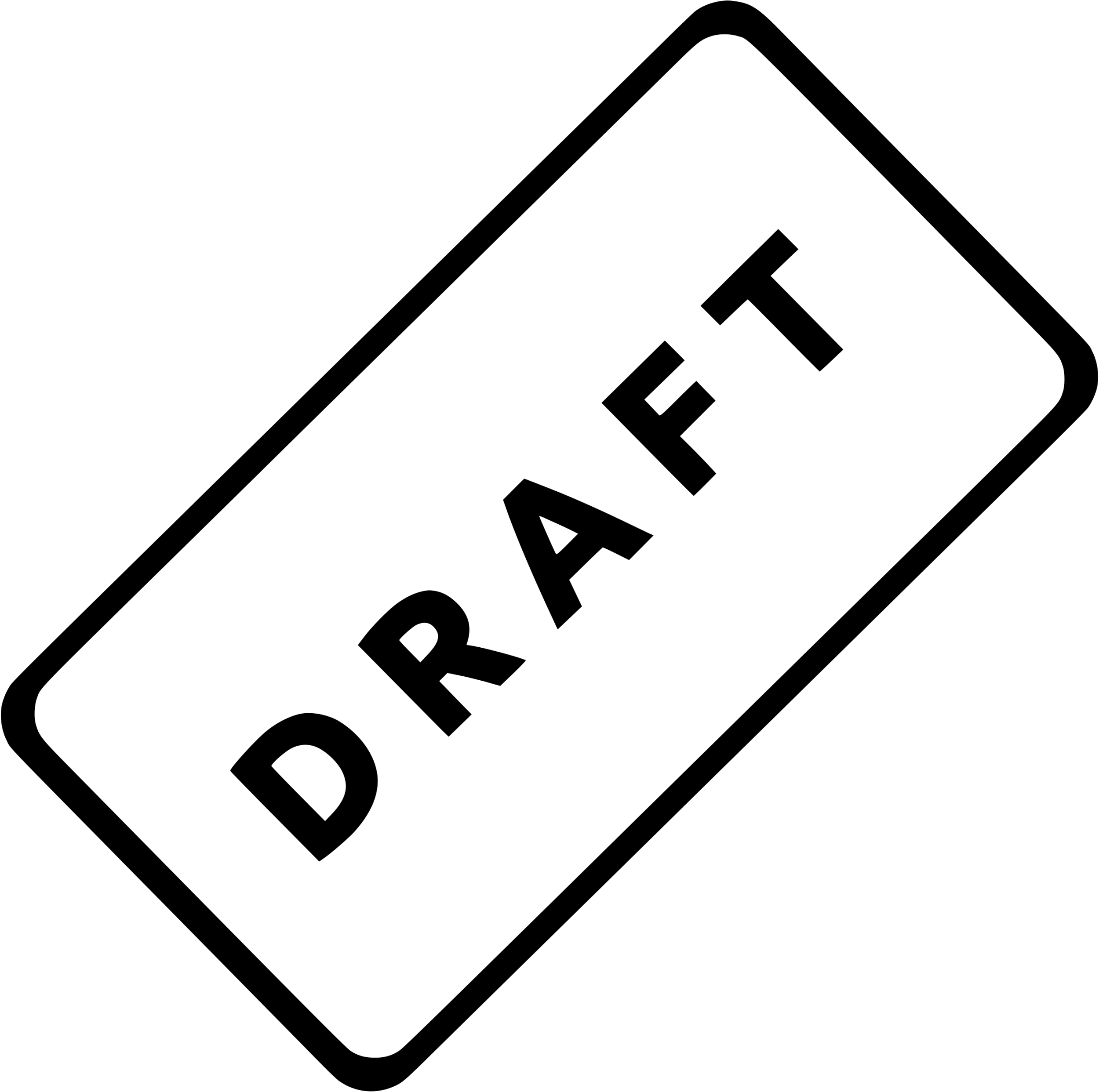 | 02. Kent |
| 01. Oil | 01. Canola Oil | 03. Teer |
| 01. Oil | 01. Canola Oil | 88. Other brands (specify) |
| 01. Oil | 01. Canola Oil | 98. Don't know |
| 01. Oil | 01. Canola Oil | 99. Unbranded |
| 01. Oil | 01. Canola Oil | 999. Unknown |
| 01. Oil | 02. Coconut Oil | 01. Ceylon Naturals |
| 01. Oil | 02. Coconut Oil | 02. Kumkum |
| 01. Oil | 02. Coconut Oil | 03. Modina |
| 01. Oil | 02. Coconut Oil | 88. Other brands (specify) |
| 01. Oil | 02. Coconut Oil | 98. Don't know |
| 01. Oil | 02. Coconut Oil | 99. Unbranded |
| 01. Oil | 02. Coconut Oil | 999. Unknown |
| 01. Oil | 03. Corn Oil | 01. Kent |
| 01. Oil | 03. Corn Oil | 02. Kent Boringer |
| 01. Oil | 03. Corn Oil | 88. Other brands (specify) |
| 01. Oil | 03. Corn Oil | 98. Don't know |
| 01. Oil | 03. Corn Oil | 99. Unbranded |
| 01. Oil | 03. Corn Oil | 999. Unknown |
| 01. Oil | 04. Mustard Oil | 01. ACI Aroma |
| 01. Oil | 04. Mustard Oil | 02. ACI Pure |
| 01. Oil | 04. Mustard Oil | 03. Akij |
| 01. Oil | 04. Mustard Oil | 04. Aroma |
| 01. Oil | 04. Mustard Oil | 05. Bashundhara |
| 01. Oil | 04. Mustard Oil | 06. Boshudha |
| 01. Oil | 04. Mustard Oil | 07. Chaka Marka |
| 01. Oil | 04. Mustard Oil | 08. Cholonto |
| 01. Oil | 04. Mustard Oil | 09. Fortune |
| 01. Oil | 04. Mustard Oil | 10. Fresh |
| 01. Oil | 04. Mustard Oil | 11. Inas |
| 01. Oil | 04. Mustard Oil | 12. Ispahani Parbon |
| 01. Oil | 04. Mustard Oil | 13. Jamai Sumon |
| 01. Oil | 04. Mustard Oil | 14. Madina |
| 01. Oil | 04. Mustard Oil | 15. Mala |
| 01. Oil | 04. Mustard Oil | 16. Mariyam |
| 01. Oil | 04. Mustard Oil | 17. Mehjabin |
| 01. Oil | 04. Mustard Oil | 18. Modina |
| 01. Oil | 04. Mustard Oil | 19. Molla |
| 01. Oil | 04. Mustard Oil | 20. Natura |
| 01. Oil | 04. Mustard Oil | 21. Nishat |
| 01. Oil | 04. Mustard Oil | 22. Nishita |
| 01. Oil | 04. Mustard Oil | 23. Pach Elish Brand |
| 01. Oil | 04. Mustard Oil | 24. Pran |
| 01. Oil | 04. Mustard Oil | 25. Projapoti Marka |
| 01. Oil | 04. Mustard Oil | 26. Pusti |
| 01. Oil | 04. Mustard Oil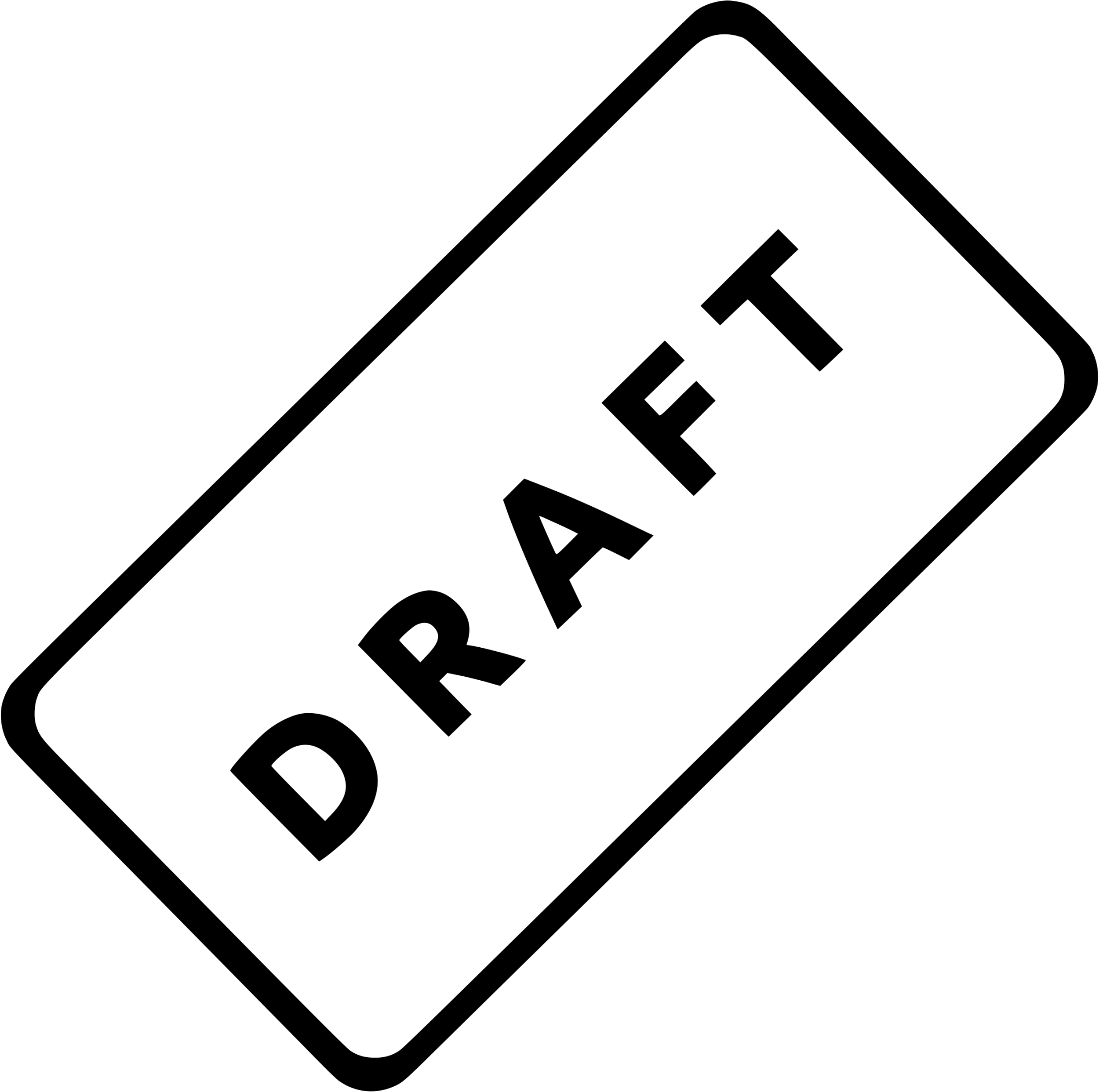 | 27. Radhuni |
| 01. Oil | 04. Mustard Oil | 28. Rashoi |
| 01. Oil | 04. Mustard Oil | 29. Rokto Joba Ful Marka |
| 01. Oil | 04. Mustard Oil | 30. Sajib |
| 01. Oil | 04. Mustard Oil | 31. sajib |
| 01. Oil | 04. Mustard Oil | 32. Sajibb |
| 01. Oil | 04. Mustard Oil | 33. Shwapno |
| 01. Oil | 04. Mustard Oil | 34. Sunshine |
| 01. Oil | 04. Mustard Oil | 35. Suresh |
| 01. Oil | 04. Mustard Oil | 36. Teer |
| 01. Oil | 04. Mustard Oil | 37. Tiya Marka |
| 01. Oil | 04. Mustard Oil | 38. Ummah |
| 01. Oil | 04. Mustard Oil | 88. Other brands (specify) |
| 01. Oil | 04. Mustard Oil | 98. Don't know |
| 01. Oil | 04. Mustard Oil | 99. Unbranded |
| 01. Oil | 04. Mustard Oil | 999. Unknown |
| 01. Oil | 05. Olive Oil | 01. Borges |
| 01. Oil | 05. Olive Oil | 02. Delight |
| 01. Oil | 05. Olive Oil | 03. Kent |
| 01. Oil | 05. Olive Oil | 04. Kent Boringer |
| 01. Oil | 05. Olive Oil | 05. La Oliva |
| 01. Oil | 05. Olive Oil | 06. Lucy Oliva |
| 01. Oil | 05. Olive Oil | 07. Luglio |
| 01. Oil | 05. Olive Oil | 08. Olio Orolio |
| 01. Oil | 05. Olive Oil | 09. Olitalia |
| 01. Oil | 05. Olive Oil | 10. Olive Oils Land |
| 01. Oil | 05. Olive Oil | 11. RS |
| 01. Oil | 05. Olive Oil | 12. Span Oliva |
| 01. Oil | 05. Olive Oil | 88. Other brands (specify) |
| 01. Oil | 05. Olive Oil | 98. Don't know |
| 01. Oil | 05. Olive Oil | 99. Unbranded |
| 01. Oil | 05. Olive Oil | 999. Unknown |
| 01. Oil | 06. Palm Oil | 01. Lucky |
| 01. Oil | 06. Palm Oil | 02. Pabda Gold |
| 01. Oil | 06. Palm Oil | 03. RBD |
| 01. Oil | 06. Palm Oil | 88. Other brands (specify) |
| 01. Oil | 06. Palm Oil | 98. Don't know |
| 01. Oil | 06. Palm Oil | 99. Unbranded |
| 01. Oil | 06. Palm Oil | 999. Unknown |
| 01. Oil | 07. Palm Olein | 01. Ayan |
| 01. Oil | 07. Palm Olein | 02. Family |
| 01. Oil | 07. Palm Olein | 03. Koly |
| 01. Oil | 07. Palm Olein | 04. Lucky |
| 01. Oil | 07. Palm Olein | 05. Mizan |
| 01. Oil | 07. Palm Olein | 06. Moon |
| 01. Oil | 07. Palm Olein | 07. Natural |
| 01. Oil | 07. Palm Olein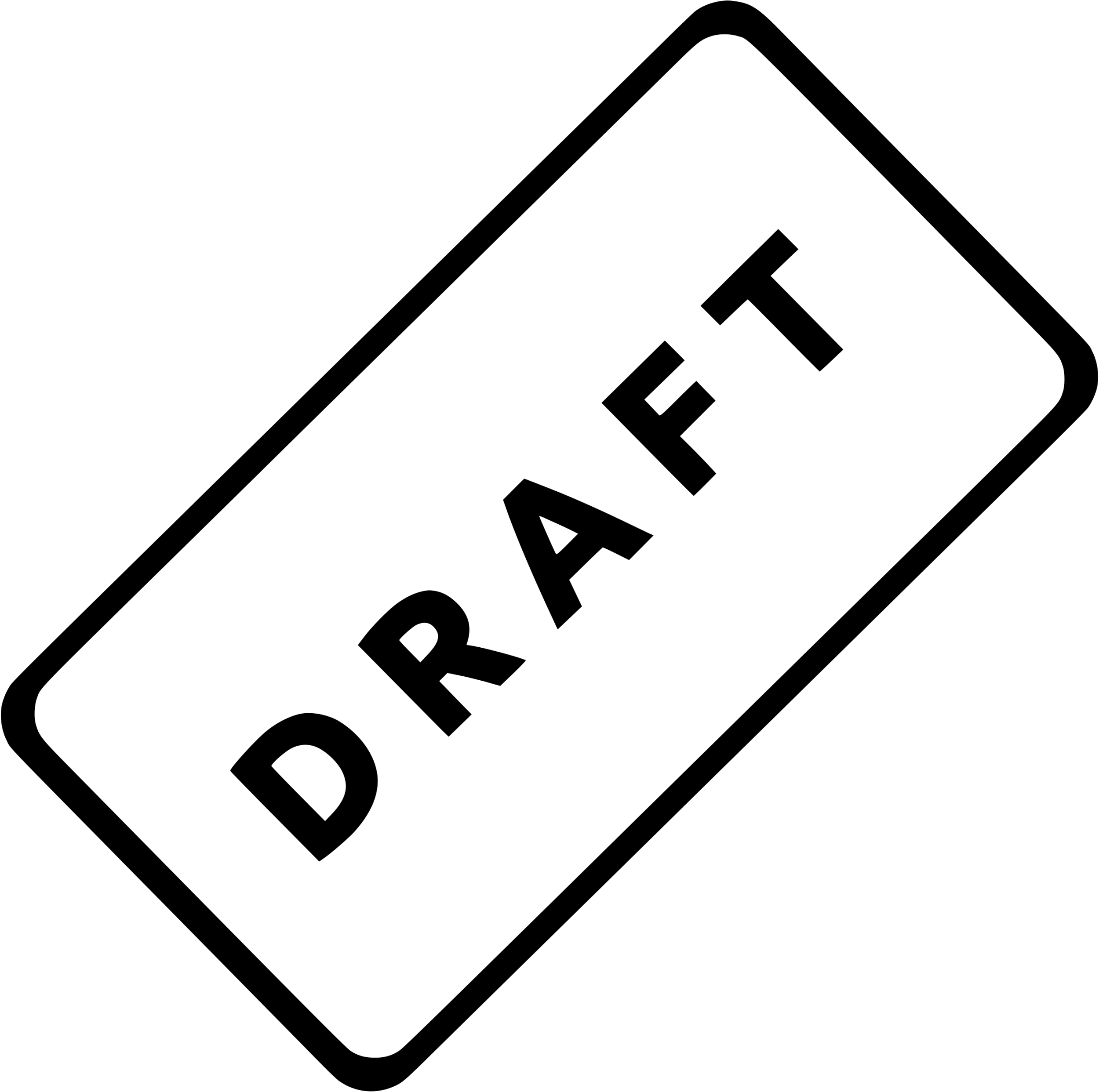 | 08. Oleo |
| 01. Oil | 07. Palm Olein | 09. Pure |
| 01. Oil | 07. Palm Olein | 10. Pure Gold |
| 01. Oil | 07. Palm Olein | 11. Shapla |
| 01. Oil | 07. Palm Olein | 12. Super Pure |
| 01. Oil | 07. Palm Olein | 88. Other brands (specify) |
| 01. Oil | 07. Palm Olein | 98. Don't know |
| 01. Oil | 07. Palm Olein | 99. Unbranded |
| 01. Oil | 07. Palm Olein | 999. Unknown |
| 01. Oil | 08. Rice Bran Oil | 01. ACI Nutrilife |
| 01. Oil | 08. Rice Bran Oil | 02. Fortune |
| 01. Oil | 08. Rice Bran Oil | 03. Mukti |
| 01. Oil | 08. Rice Bran Oil | 04. Saffola |
| 01. Oil | 08. Rice Bran Oil | 05. Saffola Active + |
| 01. Oil | 08. Rice Bran Oil | 88. Other brands (specify) |
| 01. Oil | 08. Rice Bran Oil | 98. Don't know |
| 01. Oil | 08. Rice Bran Oil | 99. Unbranded |
| 01. Oil | 08. Rice Bran Oil | 999. Unknown |
| 01. Oil | 09. Sesame Oil | 01. Fragrance |
| 01. Oil | 09. Sesame Oil | 02. R choice |
| 01. Oil | 09. Sesame Oil | 88. Other brands (specify) |
| 01. Oil | 09. Sesame Oil | 98. Don't know |
| 01. Oil | 09. Sesame Oil | 99. Unbranded |
| 01. Oil | 09. Sesame Oil | 999. Unknown |
| 01. Oil | 10. Soyabean Oil | 01. ACI Pure |
| 01. Oil | 10. Soyabean Oil | 02. Actifit |
| 01. Oil | 10. Soyabean Oil | 03. Active |
| 01. Oil | 10. Soyabean Oil | 04. Bashundhara |
| 01. Oil | 10. Soyabean Oil | 05. City |
| 01. Oil | 10. Soyabean Oil | 06. Fortune |
| 01. Oil | 10. Soyabean Oil | 07. Fresh |
| 01. Oil | 10. Soyabean Oil | 08. Glory |
| 01. Oil | 10. Soyabean Oil | 09. Modina |
| 01. Oil | 10. Soyabean Oil | 10. Pure |
| 01. Oil | 10. Soyabean Oil | 11. Pushti |
| 01. Oil | 10. Soyabean Oil | 12. Pusti |
| 01. Oil | 10. Soyabean Oil | 13. Ranna |
| 01. Oil | 10. Soyabean Oil | 14. Rupchanda |
| 01. Oil | 10. Soyabean Oil | 15. Saudia |
| 01. Oil | 10. Soyabean Oil | 16. Sena |
| 01. Oil | 10. Soyabean Oil | 17. Starship |
| 01. Oil | 10. Soyabean Oil | 18. Starship Zahaj |
| 01. Oil | 10. Soyabean Oil | 19. Teer |
| 01. Oil | 10. Soyabean Oil | 20. Veola |
| 01. Oil | 10. Soyabean Oil | 88. Other brands (specify) |
| 01. Oil | 10. Soyabean Oil | 98. Don't know |
| 01. Oil | 10. Soyabean Oil | 99. Unbranded |
| 01. Oil | 10. Soyabean Oil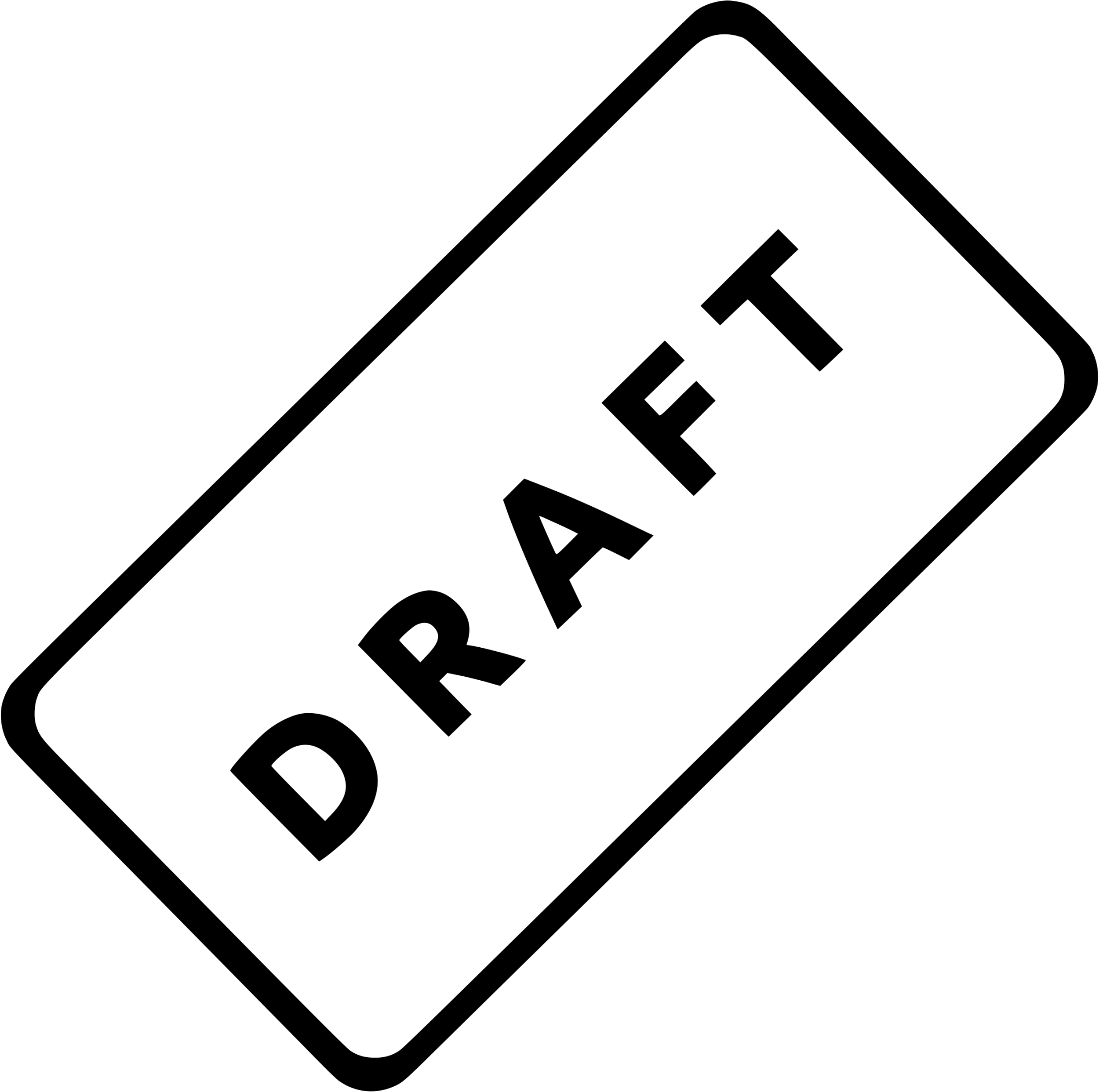 | 999. Unknown |
| 01. Oil | 11. Sunflower Oil | 01. Blanc |
| 01. Oil | 11. Sunflower Oil | 02. Borges |
| 01. Oil | 11. Sunflower Oil | 03. Discovery |
| 01. Oil | 11. Sunflower Oil | 04. Ecorganic |
| 01. Oil | 11. Sunflower Oil | 05. Finedine |
| 01. Oil | 11. Sunflower Oil | 06. Fortune |
| 01. Oil | 11. Sunflower Oil | 07. Kent |
| 01. Oil | 11. Sunflower Oil | 08. kent |
| 01. Oil | 11. Sunflower Oil | 09. Kent boringer |
| 01. Oil | 11. Sunflower Oil | 10. Kernel |
| 01. Oil | 11. Sunflower Oil | 11. Kings |
| 01. Oil | 11. Sunflower Oil | 12. La Espaniola |
| 01. Oil | 11. Sunflower Oil | 13. Le Blanc |
| 01. Oil | 11. Sunflower Oil | 14. Olitalia |
| 01. Oil | 11. Sunflower Oil | 15. Premi |
| 01. Oil | 11. Sunflower Oil | 16. Radhuni |
| 01. Oil | 11. Sunflower Oil | 17. Sun Empire |
| 01. Oil | 11. Sunflower Oil | 18. Truco |
| 01. Oil | 11. Sunflower Oil | 19. Trucq |
| 01. Oil | 11. Sunflower Oil | 88. Other brands (specify) |
| 01. Oil | 11. Sunflower Oil | 98. Don't know |
| 01. Oil | 11. Sunflower Oil | 99. Unbranded |
| 01. Oil | 11. Sunflower Oil | 999. Unknown |
| 01. Oil | 12. Super Palm Oil | 88. Other brands (specify) |
| 01. Oil | 12. Super Palm Oil | 98. Don't know |
| 01. Oil | 12. Super Palm Oil | 99. Unbranded |
| 01. Oil | 12. Super Palm Oil | 999. Unknown |
| 01. Oil | 13. Super Palm Olein | 01. Pure Gold |
| 01. Oil | 13. Super Palm Olein | 88. Other brands (specify) |
| 01. Oil | 13. Super Palm Olein | 98. Don't know |
| 01. Oil | 13. Super Palm Olein | 99. Unbranded |
| 01. Oil | 13. Super Palm Olein | 999. Unknown |
| 01. Oil | 88. Other types (specify) |  |
| 01. Oil | 98. Don't know |  |
| 02. Wheat flour | 01. Brown Wheat Flour/Whole Wheat Flour | 01. ACI Nutrilife |
| 02. Wheat flour | 01. Brown Wheat Flour/Whole Wheat Flour | 02. Bashundhara |
| 02. Wheat flour | 01. Brown Wheat Flour/Whole Wheat Flour | 03. Meena |
| 02. Wheat flour | 01. Brown Wheat Flour/Whole Wheat Flour | 04. Nishita |
| 02. Wheat flour | 01. Brown Wheat Flour/Whole Wheat Flour | 05. Shaad |
| 02. Wheat flour | 01. Brown Wheat Flour/Whole Wheat Flour | 06. Sunshine |
| 02. Wheat flour | 01. Brown Wheat Flour/Whole Wheat Flour | 07. Teer |
| 02. Wheat flour | 01. Brown Wheat Flour/Whole Wheat Flour | 88. Other brands (specify) |
| 02. Wheat flour | 01. Brown Wheat Flour/Whole Wheat Flour | 98. Don't know |
| 02. Wheat flour | 01. Brown Wheat Flour/Whole Wheat Flour | 99. Unbranded |
| 02. Wheat flour | 01. Brown Wheat Flour/Whole Wheat Flour | 999. Unknown |
| 02. Wheat flour | 02. Cake Wheat Flour/All-purpose Wheat | 01. Aci Pure |
| 02. Wheat flour | 02. Cake Wheat Flour/All-purpose Wheat | 02. Century |
| 02. Wheat flour | 02. Cake Wheat Flour/All-purpose Wheat | 03. Nishita |
| 02. Wheat flour | 02. Cake Wheat Flour/All-purpose Wheat | 88. Other brands (specify) |
| 02. Wheat flour | 02. Cake Wheat Flour/All-purpose Wheat | 98. Don't know |
| 02. Wheat flour | 02. Cake Wheat Flour/All-purpose Wheat | 99. Unbranded |
| 02. Wheat flour | 02. Cake Wheat Flour/All-purpose Wheat | 999. Unknown |
| 02. Wheat flour | 03. Rice flour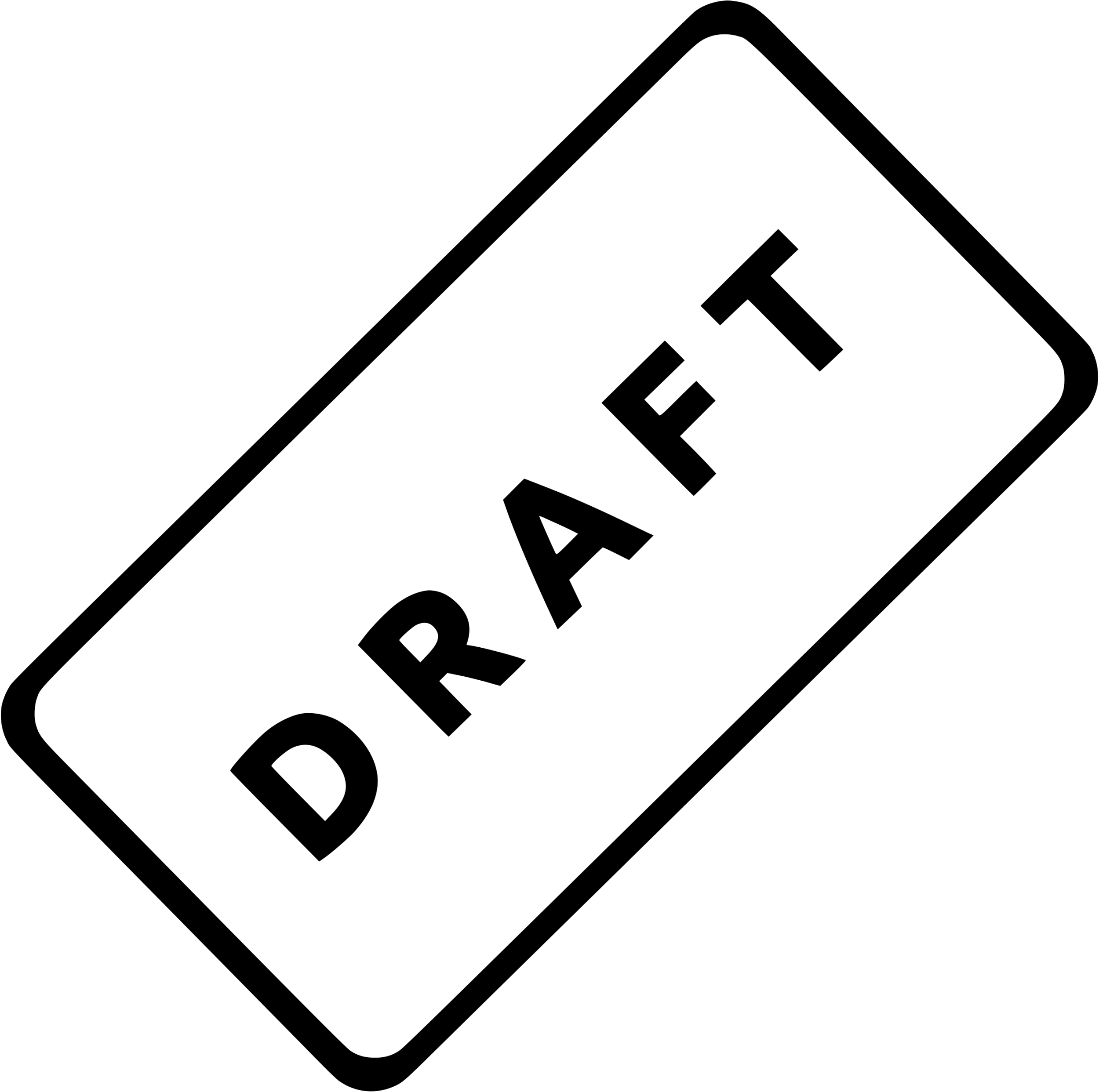 | 01. Vitcare |
| 02. Wheat flour | 03. Rice flour | 88. Other brands (specify) |
| 02. Wheat flour | 03. Rice flour | 98. Don't know |
| 02. Wheat flour | 03. Rice flour | 99. Unbranded |
| 02. Wheat flour | 03. Rice flour | 999. Unknown |
| 02. Wheat flour | 04. Refined Wheat Flour/Moida | 01. Aci Pure |
| 02. Wheat flour | 04. Refined Wheat Flour/Moida | 02. Actifit |
| 02. Wheat flour | 04. Refined Wheat Flour/Moida | 03. Bashundhara |
| 02. Wheat flour | 04. Refined Wheat Flour/Moida | 04. Dalia |
| 02. Wheat flour | 04. Refined Wheat Flour/Moida | 05. Farmland |
| 02. Wheat flour | 04. Refined Wheat Flour/Moida | 06. Fresh |
| 02. Wheat flour | 04. Refined Wheat Flour/Moida | 07. Nishita |
| 02. Wheat flour | 04. Refined Wheat Flour/Moida | 08. Pusti |
| 02. Wheat flour | 04. Refined Wheat Flour/Moida | 09. Ruchi |
| 02. Wheat flour | 04. Refined Wheat Flour/Moida | 10. Sena |
| 02. Wheat flour | 04. Refined Wheat Flour/Moida | 11. Shaad |
| 02. Wheat flour | 04. Refined Wheat Flour/Moida | 12. Sima |
| 02. Wheat flour | 04. Refined Wheat Flour/Moida | 13. Sunshine |
| 02. Wheat flour | 04. Refined Wheat Flour/Moida | 14. Teer |
| 02. Wheat flour | 04. Refined Wheat Flour/Moida | 15. Tin Chabi |
| 02. Wheat flour | 04. Refined Wheat Flour/Moida | 88. Other brands (specify) |
| 02. Wheat flour | 04. Refined Wheat Flour/Moida | 98. Don't know |
| 02. Wheat flour | 04. Refined Wheat Flour/Moida | 99. Unbranded |
| 02. Wheat flour | 04. Refined Wheat Flour/Moida | 999. Unknown |
| 02. Wheat flour | 05. Wheat Flour | 01. ACI Pure |
| 02. Wheat flour | 05. Wheat Flour | 02. Actifit |
| 02. Wheat flour | 05. Wheat Flour | 03. Bashundhara |
| 02. Wheat flour | 05. Wheat Flour | 04. Farmland |
| 02. Wheat flour | 05. Wheat Flour | 05. Fresh |
| 02. Wheat flour | 05. Wheat Flour | 06. Jonoprio Major |
| 02. Wheat flour | 05. Wheat Flour | 07. Kawsar Flour Mil |
| 02. Wheat flour | 05. Wheat Flour | 08. Kosmic Major |
| 02. Wheat flour | 05. Wheat Flour | 09. Machranga |
| 02. Wheat flour | 05. Wheat Flour | 10. Manjil |
| 02. Wheat flour | 05. Wheat Flour | 11. No. 1 |
| 02. Wheat flour | 05. Wheat Flour | 12. One |
| 02. Wheat flour | 05. Wheat Flour | 13. Palki |
| 02. Wheat flour | 05. Wheat Flour | 14. Pusti |
| 02. Wheat flour | 05. Wheat Flour | 15. Saworna |
| 02. Wheat flour | 05. Wheat Flour | 16. Sena |
| 02. Wheat flour | 05. Wheat Flour | 17. Shaad |
| 02. Wheat flour | 05. Wheat Flour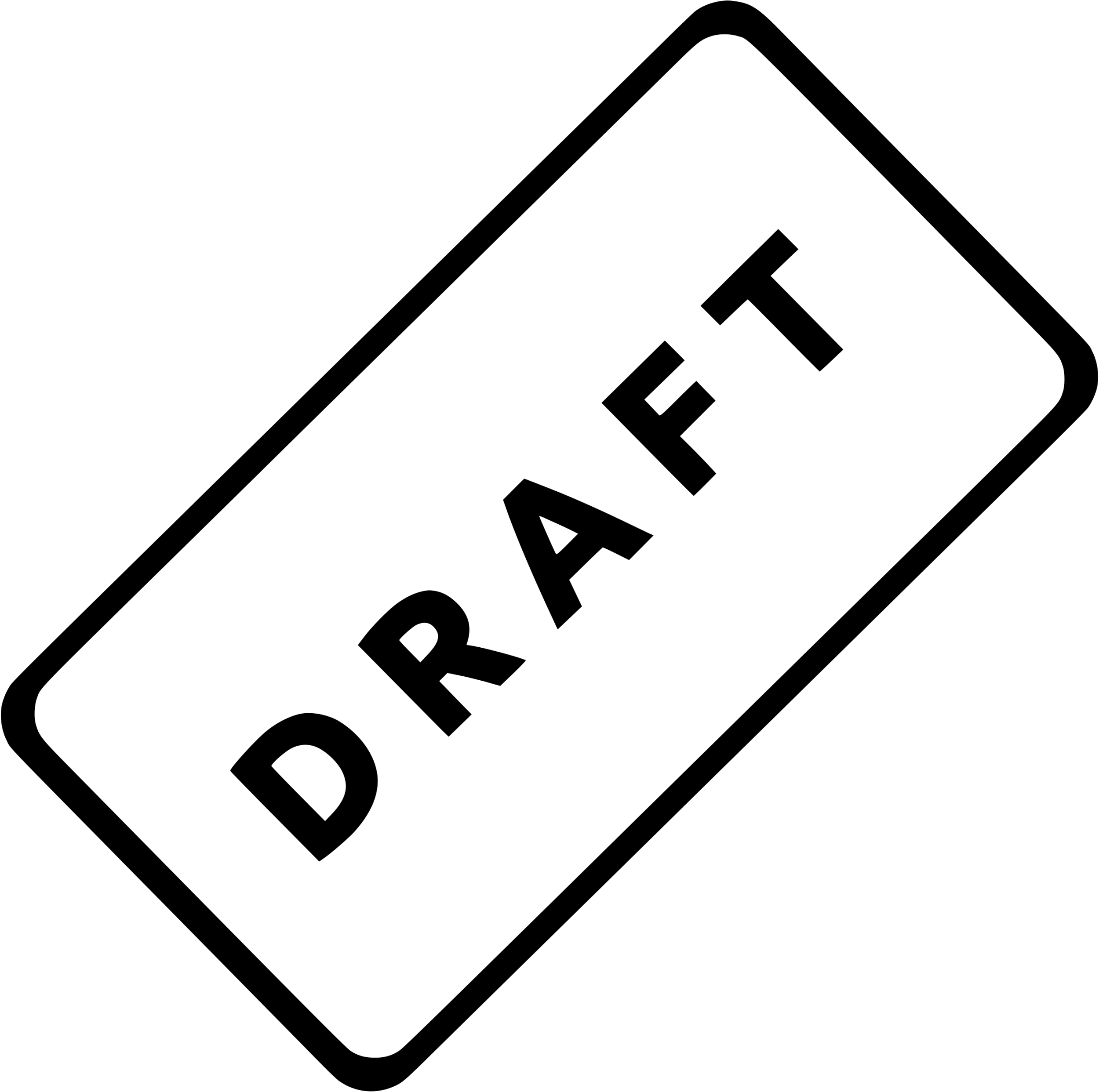 | 18. Shahjalal |
| 02. Wheat flour | 05. Wheat Flour | 19. Sunshine |
| 02. Wheat flour | 05. Wheat Flour | 20. Teer |
| 02. Wheat flour | 05. Wheat Flour | 88. Other brands (specify) |
| 02. Wheat flour | 05. Wheat Flour | 98. Don't know |
| 02. Wheat flour | 05. Wheat Flour | 99. Unbranded |
| 02. Wheat flour | 05. Wheat Flour | 999. Unknown |
| 02. Wheat flour | 88. Other types (specify) |  |
| 02. Wheat flour | 98. Don't know |  |
| 03. Salt | 01. Coarse Salt | 88. Other brands (specify) |
| 03. Salt | 01. Coarse Salt | 98. Don't know |
| 03. Salt | 01. Coarse Salt | 99. Unbranded |
| 03. Salt | 01. Coarse Salt | 999. Unknown |
| 03. Salt | 02. Fine Table Salt | 01. ACI Pure Salt |
| 03. Salt | 02. Fine Table Salt | 02. BMC Super |
| 03. Salt | 02. Fine Table Salt | 03. City pure |
| 03. Salt | 02. Fine Table Salt | 04. Confidence |
| 03. Salt | 02. Fine Table Salt | 05. Fresh |
| 03. Salt | 02. Fine Table Salt | 06. Kobir |
| 03. Salt | 02. Fine Table Salt | 07. Molla |
| 03. Salt | 02. Fine Table Salt | 08. Muskan |
| 03. Salt | 02. Fine Table Salt | 09. No.1 |
| 03. Salt | 02. Fine Table Salt | 10. Pran |
| 03. Salt | 02. Fine Table Salt | 11. Teer |
| 03. Salt | 02. Fine Table Salt | 88. Other brands (specify) |
| 03. Salt | 02. Fine Table Salt | 98. Don't know |
| 03. Salt | 02. Fine Table Salt | 99. Unbranded |
| 03. Salt | 02. Fine Table Salt | 999. Unknown |
| 03. Salt | 03. Pink Salt | 01. Himalayan |
| 03. Salt | 03. Pink Salt | 02. Shwapno |
| 03. Salt | 03. Pink Salt | 88. Other brands (specify) |
| 03. Salt | 03. Pink Salt | 98. Don't know |
| 03. Salt | 03. Pink Salt | 99. Unbranded |
| 03. Salt | 03. Pink Salt | 999. Unknown |
| 03. Salt | 04. Rock Salt | 01. AZA ideal |
| 03. Salt | 04. Rock Salt | 02. Grihini |
| 03. Salt | 04. Rock Salt | 03. Haiko |
| 03. Salt | 04. Rock Salt | 04. Marlta |
| 03. Salt | 04. Rock Salt | 05. Prapti Astha Moshla |
| 03. Salt | 04. Rock Salt | 06. Rahmania Black Salt |
| 03. Salt | 04. Rock Salt | 07. Rhoman |
| 03. Salt | 04. Rock Salt | 08. Roshni |
| 03. Salt | 04. Rock Salt | 09. Saad |
| 03. Salt | 04. Rock Salt | 10. Shwapno |
| 03. Salt | 04. Rock Salt | 11. Sonali |
| 03. Salt | 04. Rock Salt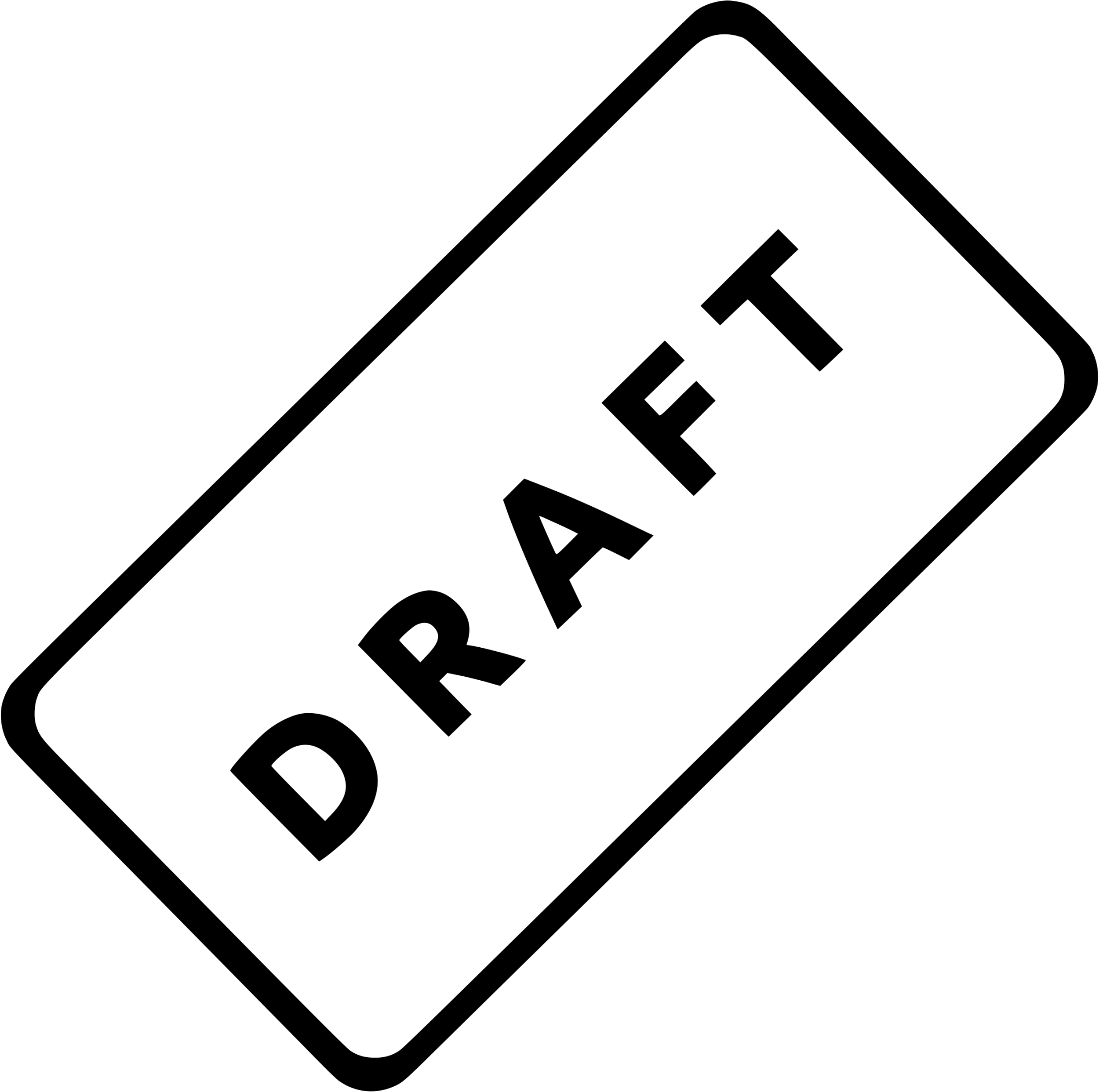 | 88. Other brands (specify) |
| 03. Salt | 04. Rock Salt | 98. Don't know |
| 03. Salt | 04. Rock Salt | 99. Unbranded |
| 03. Salt | 04. Rock Salt | 999. Unknown |
| 03. Salt | 88. Other types (specify) |  |
| 03. Salt | 98. Don't know |  |
| 04. Rice | 01. Aman Rice | 88. Other brands (specify) |
| 04. Rice | 01. Aman Rice | 98. Don't know |
| 04. Rice | 01. Aman Rice | 99. Unbranded |
| 04. Rice | 01. Aman Rice | 999. Unknown |
| 04. Rice | 02. Athash Rice | 01. Al Razzak |
| 04. Rice | 02. Athash Rice | 02. Amin |
| 04. Rice | 02. Athash Rice | 03. Bhai Bhai |
| 04. Rice | 02. Athash Rice | 04. Bhuiya |
| 04. Rice | 02. Athash Rice | 05. Durga |
| 04. Rice | 02. Athash Rice | 06. Food Department Provided Rice |
| 04. Rice | 02. Athash Rice | 07. Harin |
| 04. Rice | 02. Athash Rice | 08. National |
| 04. Rice | 02. Athash Rice | 09. Palki |
| 04. Rice | 02. Athash Rice | 10. Rabbi |
| 04. Rice | 02. Athash Rice | 11. SK Traders |
| 04. Rice | 02. Athash Rice | 12. Sonar chabi |
| 04. Rice | 02. Athash Rice | 88. Other brands (specify) |
| 04. Rice | 02. Athash Rice | 98. Don't know |
| 04. Rice | 02. Athash Rice | 99. Unbranded |
| 04. Rice | 02. Athash Rice | 999. Unknown |
| 04. Rice | 03. Atop Rice | 01. Horin marka |
| 04. Rice | 03. Atop Rice | 02. Jobbar Jote mils |
| 04. Rice | 03. Atop Rice | 03. Jora Tiya |
| 04. Rice | 03. Atop Rice | 04. Katari Atop |
| 04. Rice | 03. Atop Rice | 05. Nobanno |
| 04. Rice | 03. Atop Rice | 06. Professor Mizanur Rahman |
| 04. Rice | 03. Atop Rice | 07. Rajanighanda |
| 04. Rice | 03. Atop Rice | 08. Sena |
| 04. Rice | 03. Atop Rice | 09. Shwapno |
| 04. Rice | 03. Atop Rice | 88. Other brands (specify) |
| 04. Rice | 03. Atop Rice | 98. Don't know |
| 04. Rice | 03. Atop Rice | 99. Unbranded |
| 04. Rice | 03. Atop Rice | 999. Unknown |
| 04. Rice | 04. Aus Rice | 01. bpm |
| 04. Rice | 04. Aus Rice | 02. Harun and sons |
| 04. Rice | 04. Aus Rice | 88. Other brands (specify) |
| 04. Rice | 04. Aus Rice | 98. Don't know |
| 04. Rice | 04. Aus Rice | 99. Unbranded |
| 04. Rice | 04. Aus Rice | 999. Unknown |
| 04. Rice | 05. Banglamoti Rice | 01. ACI |
| 04. Rice | 05. Banglamoti Rice | 02. ACI Pure |
| 04. Rice | 05. Banglamoti Rice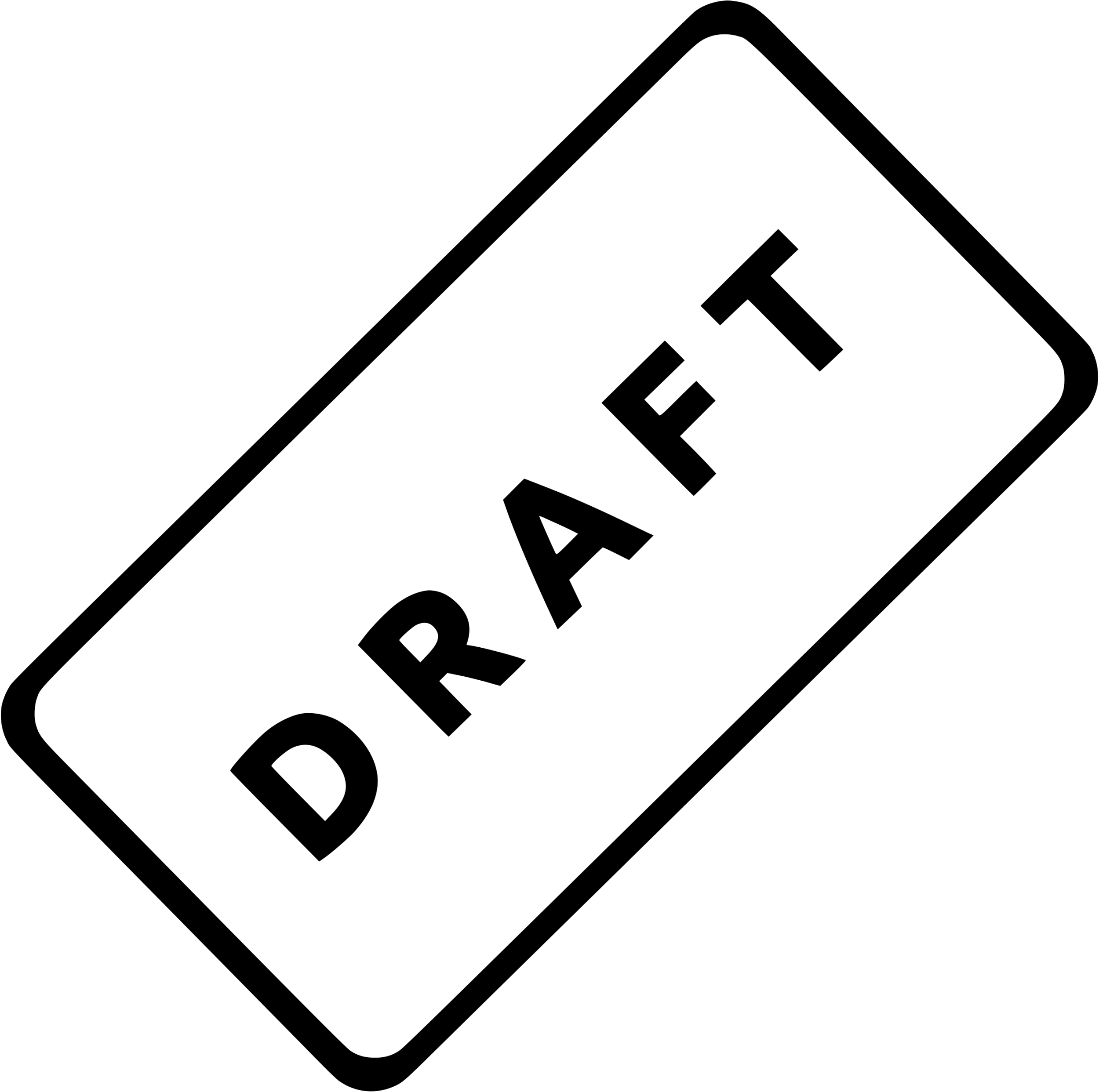 | 03. Arong Natural |
| 04. Rice | 05. Banglamoti Rice | 04. Krishan |
| 04. Rice | 05. Banglamoti Rice | 05. Pran (Rice) |
| 04. Rice | 05. Banglamoti Rice | 88. Other brands (specify) |
| 04. Rice | 05. Banglamoti Rice | 98. Don't know |
| 04. Rice | 05. Banglamoti Rice | 99. Unbranded |
| 04. Rice | 05. Banglamoti Rice | 999. Unknown |
| 04. Rice | 06. Bashful Rice | 01. Dhamrai Super |
| 04. Rice | 06. Bashful Rice | 88. Other brands (specify) |
| 04. Rice | 06. Bashful Rice | 98. Don't know |
| 04. Rice | 06. Bashful Rice | 99. Unbranded |
| 04. Rice | 06. Bashful Rice | 999. Unknown |
| 04. Rice | 07. Basmati Rice | 01. ACI |
| 04. Rice | 07. Basmati Rice | 02. Aroma |
| 04. Rice | 07. Basmati Rice | 03. Arong Natural |
| 04. Rice | 07. Basmati Rice | 04. Bushra |
| 04. Rice | 07. Basmati Rice | 05. Daawat |
| 04. Rice | 07. Basmati Rice | 06. Desi Bashmoti |
| 04. Rice | 07. Basmati Rice | 07. Fortune (Rice) |
| 04. Rice | 07. Basmati Rice | 08. Four Season |
| 04. Rice | 07. Basmati Rice | 09. Hafsa |
| 04. Rice | 07. Basmati Rice | 10. India Gate |
| 04. Rice | 07. Basmati Rice | 11. Kohinoor |
| 04. Rice | 07. Basmati Rice | 12. Kohinoor Classic |
| 04. Rice | 07. Basmati Rice | 13. Kohinoor Gold Queen |
| 04. Rice | 07. Basmati Rice | 14. Lazzat |
| 04. Rice | 07. Basmati Rice | 15. Mehran |
| 04. Rice | 07. Basmati Rice | 16. Metro |
| 04. Rice | 07. Basmati Rice | 17. Palki |
| 04. Rice | 07. Basmati Rice | 18. Pran |
| 04. Rice | 07. Basmati Rice | 19. Shazia |
| 04. Rice | 07. Basmati Rice | 20. Sheha |
| 04. Rice | 07. Basmati Rice | 21. Taj Mahal |
| 04. Rice | 07. Basmati Rice | 22. Tilda |
| 04. Rice | 07. Basmati Rice | 88. Other brands (specify) |
| 04. Rice | 07. Basmati Rice | 98. Don't know |
| 04. Rice | 07. Basmati Rice | 99. Unbranded |
| 04. Rice | 07. Basmati Rice | 999. Unknown |
| 04. Rice | 08. Birun Rice | 01. bpm |
| 04. Rice | 08. Birun Rice | 02. bpm sada birun |
| 04. Rice | 08. Birun Rice | 88. Other brands (specify) |
| 04. Rice | 08. Birun Rice | 98. Don't know |
| 04. Rice | 08. Birun Rice | 99. Unbranded |
| 04. Rice | 08. Birun Rice | 999. Unknown |
| 04. Rice | 09. Black Rice | 01. bpm |
| 04. Rice | 09. Black Rice | 02. Aci Nutrilife |
| 04. Rice | 09. Black Rice | 88. Other brands (specify) |
| 04. Rice | 09. Black Rice | 98. Don't know |
| 04. Rice | 09. Black Rice | 99. Unbranded |
| 04. Rice | 09. Black Rice | 999. Unknown |
| 04. Rice | 10. Boro Rice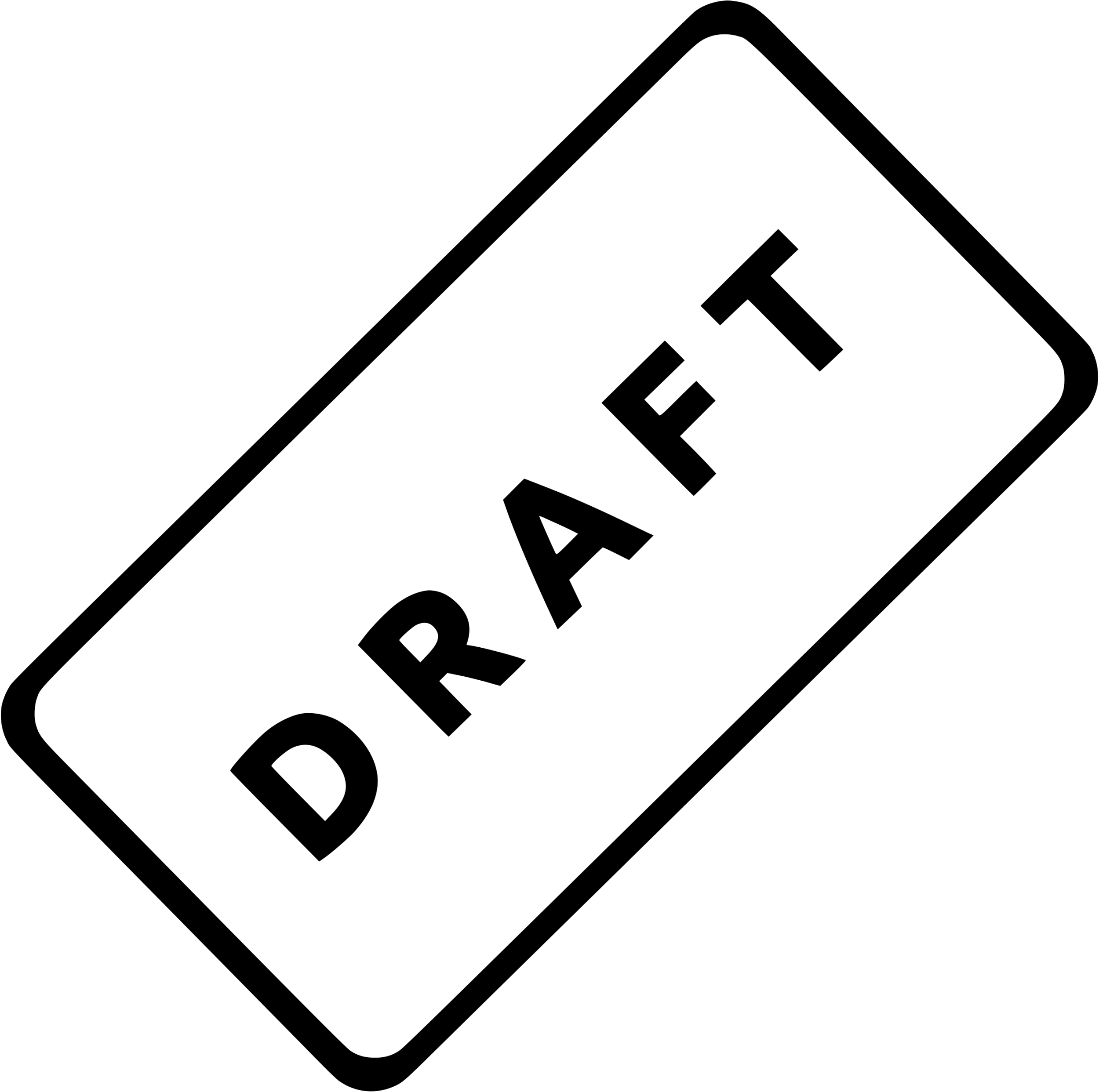 | 01. Dhamrai |
| 04. Rice | 10. Boro Rice | 88. Other brands (specify) |
| 04. Rice | 10. Boro Rice | 98. Don't know |
| 04. Rice | 10. Boro Rice | 99. Unbranded |
| 04. Rice | 10. Boro Rice | 999. Unknown |
| 04. Rice | 11. Cinigura Rice | 01. ACI |
| 04. Rice | 11. Cinigura Rice | 02. ACI Aroma |
| 04. Rice | 11. Cinigura Rice | 03. ACI Pure |
| 04. Rice | 11. Cinigura Rice | 04. Akij essential |
| 04. Rice | 11. Cinigura Rice | 05. Anuwar Special |
| 04. Rice | 11. Cinigura Rice | 06. Appel |
| 04. Rice | 11. Cinigura Rice | 07. Aroma |
| 04. Rice | 11. Cinigura Rice | 08. Aromatic |
| 04. Rice | 11. Cinigura Rice | 09. Arong Natural |
| 04. Rice | 11. Cinigura Rice | 10. Baburchi |
| 04. Rice | 11. Cinigura Rice | 11. Baburci |
| 04. Rice | 11. Cinigura Rice | 12. Bashundhara |
| 04. Rice | 11. Cinigura Rice | 13. Bpm |
| 04. Rice | 11. Cinigura Rice | 14. Chasi |
| 04. Rice | 11. Cinigura Rice | 15. Erfan |
| 04. Rice | 11. Cinigura Rice | 16. Essential |
| 04. Rice | 11. Cinigura Rice | 17. Fahim |
| 04. Rice | 11. Cinigura Rice | 18. Farmland |
| 04. Rice | 11. Cinigura Rice | 19. Fresh |
| 04. Rice | 11. Cinigura Rice | 20. Golden Key |
| 04. Rice | 11. Cinigura Rice | 21. Ispahani |
| 04. Rice | 11. Cinigura Rice | 22. Molla |
| 04. Rice | 11. Cinigura Rice | 23. Monjur |
| 04. Rice | 11. Cinigura Rice | 24. Munjur Special |
| 04. Rice | 11. Cinigura Rice | 25. Nahid |
| 04. Rice | 11. Cinigura Rice | 26. Parban |
| 04. Rice | 11. Cinigura Rice | 27. Pran |
| 04. Rice | 11. Cinigura Rice | 28. Pusti |
| 04. Rice | 11. Cinigura Rice | 29. Rajanighanda |
| 04. Rice | 11. Cinigura Rice | 030. Rupchanda |
| 04. Rice | 11. Cinigura Rice | 31. Shwapno |
| 04. Rice | 11. Cinigura Rice | 32. Sonali |
| 04. Rice | 11. Cinigura Rice | 33. Sonar Chabi |
| 04. Rice | 11. Cinigura Rice | 34. Sunshine |
| 04. Rice | 11. Cinigura Rice | 35. Teer |
| 04. Rice | 11. Cinigura Rice | 88. Other brands (specify) |
| 04. Rice | 11. Cinigura Rice | 98. Don't know |
| 04. Rice | 11. Cinigura Rice | 99. Unbranded |
| 04. Rice | 11. Cinigura Rice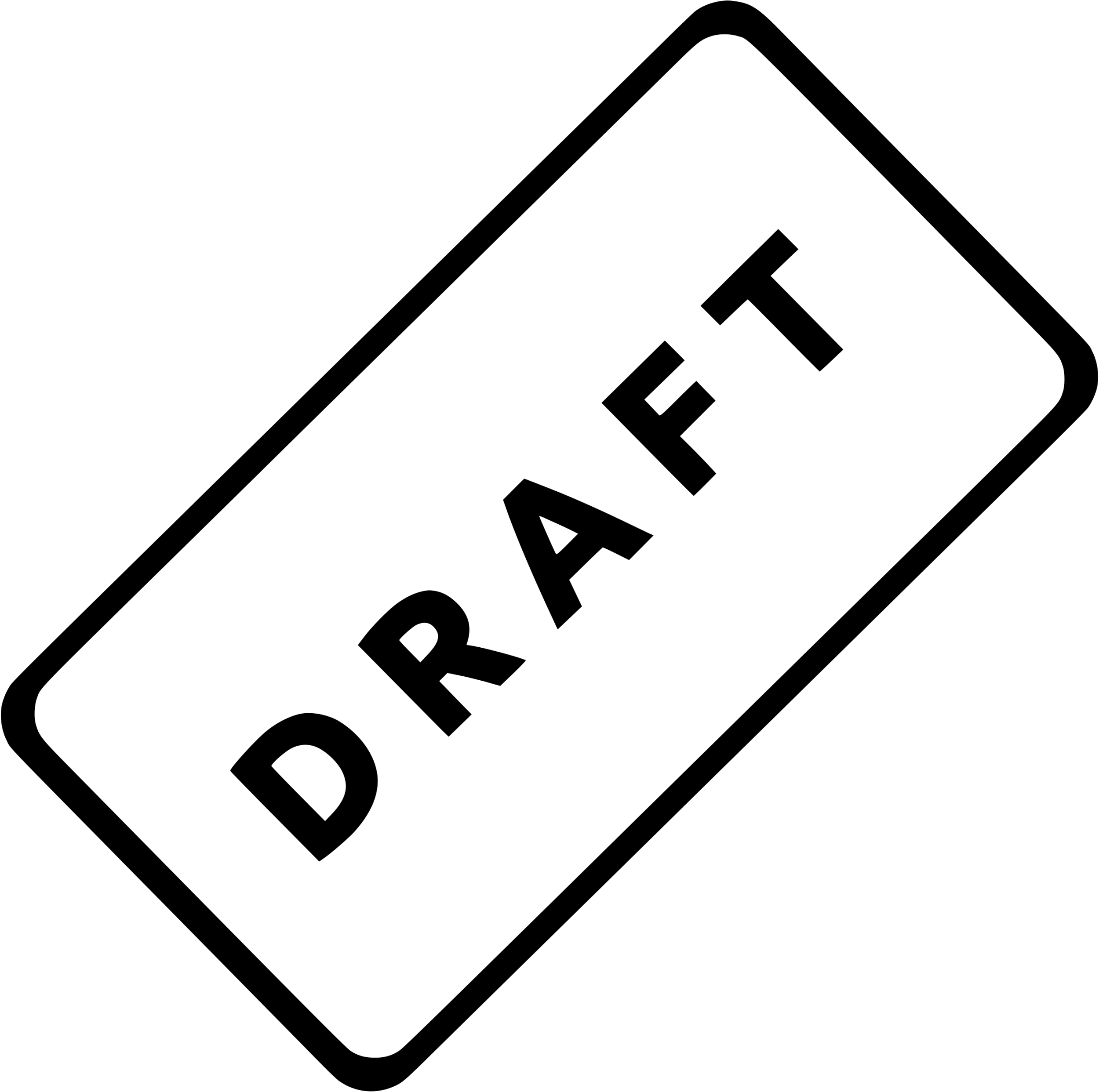 | 999. Unknown |
| 04. Rice | 12. Flattened Rice | 01. ACI |
| 04. Rice | 12. Flattened Rice | 02. Dolphin |
| 04. Rice | 12. Flattened Rice | 03. Mita |
| 04. Rice | 12. Flattened Rice | 04. Muslim |
| 04. Rice | 12. Flattened Rice | 05. New Mitali |
| 04. Rice | 12. Flattened Rice | 06. Pran |
| 04. Rice | 12. Flattened Rice | 07. Prapti |
| 04. Rice | 12. Flattened Rice | 08. Shwapno |
| 04. Rice | 12. Flattened Rice | 99. Unbranded |
| 04. Rice | 12. Flattened Rice | 10. Unknown |
| 04. Rice | 12. Flattened Rice | 11. Zamzam |
| 04. Rice | 12. Flattened Rice | 88. Other brands (specify) |
| 04. Rice | 12. Flattened Rice | 98. Don't know |
| 04. Rice | 12. Flattened Rice | 99. Unbranded |
| 04. Rice | 12. Flattened Rice | 999. Unknown |
| 04. Rice | 13. Guti Chal | 01. Diamond |
| 04. Rice | 13. Guti Chal | 88. Other brands (specify) |
| 04. Rice | 13. Guti Chal | 98. Don't know |
| 04. Rice | 13. Guti Chal | 99. Unbranded |
| 04. Rice | 13. Guti Chal | 999. Unknown |
| 04. Rice | 14. Hira | 01. Harun and sons |
| 04. Rice | 14. Hira | 88. Other brands (specify) |
| 04. Rice | 14. Hira | 98. Don't know |
| 04. Rice | 14. Hira | 99. Unbranded |
| 04. Rice | 14. Hira | 999. Unknown |
| 04. Rice | 15. Kajal Lata Rice | 01. Dolphin |
| 04. Rice | 15. Kajal Lata Rice | 88. Other brands (specify) |
| 04. Rice | 15. Kajal Lata Rice | 98. Don't know |
| 04. Rice | 15. Kajal Lata Rice | 99. Unbranded |
| 04. Rice | 15. Kajal Lata Rice | 999. Unknown |
| 04. Rice | 16. Kalijira Rice | 01. Akij essential |
| 04. Rice | 16. Kalijira Rice | 02. Arong Natural |
| 04. Rice | 16. Kalijira Rice | 03. Jahid Special |
| 04. Rice | 16. Kalijira Rice | 04. Pran |
| 04. Rice | 16. Kalijira Rice | 05. Shonar Tori |
| 04. Rice | 16. Kalijira Rice | 88. Other brands (specify) |
| 04. Rice | 16. Kalijira Rice | 98. Don't know |
| 04. Rice | 16. Kalijira Rice | 99. Unbranded |
| 04. Rice | 16. Kalijira Rice | 999. Unknown |
| 04. Rice | 17. Katari Boiled Rice | 01. ACI |
| 04. Rice | 17. Katari Boiled Rice | 02. Bashori |
| 04. Rice | 17. Katari Boiled Rice | 03. Gondhoraj |
| 04. Rice | 17. Katari Boiled Rice | 04. Jahanara |
| 04. Rice | 17. Katari Boiled Rice | 05. Manaf Super |
| 04. Rice | 17. Katari Boiled Rice | 06. Mujammel |
| 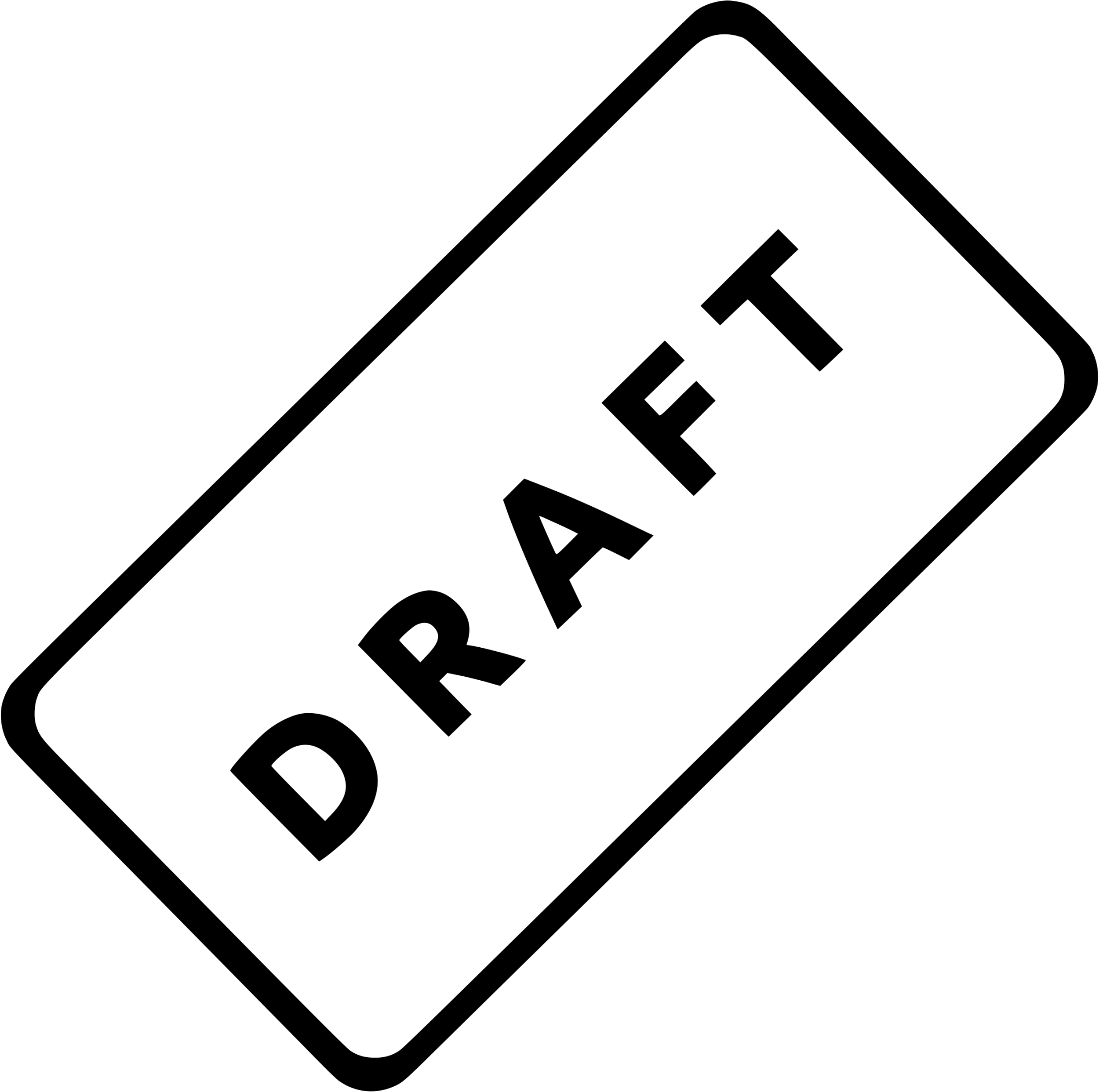04. Rice | 17. Katari Boiled Rice | 07. Nabanno |
| 04. Rice | 17. Katari Boiled Rice | 08. Shokti Premium |
| 04. Rice | 17. Katari Boiled Rice | 09. Sonar Horin |
| 04. Rice | 17. Katari Boiled Rice | 10. Tia Pakhi |
| 04. Rice | 17. Katari Boiled Rice | 88. Other brands (specify) |
| 04. Rice | 17. Katari Boiled Rice | 98. Don't know |
| 04. Rice | 17. Katari Boiled Rice | 99. Unbranded |
| 04. Rice | 17. Katari Boiled Rice | 999. Unknown |
| 04. Rice | 18. Katari Nazir | 01. Kibria |
| 04. Rice | 18. Katari Nazir | 88. Other brands (specify) |
| 04. Rice | 18. Katari Nazir | 98. Don't know |
| 04. Rice | 18. Katari Nazir | 99. Unbranded |
| 04. Rice | 18. Katari Nazir | 999. Unknown |
| 04. Rice | 19. Kaun Rice | 01. bpm |
| 04. Rice | 19. Kaun Rice | 88. Other brands (specify) |
| 04. Rice | 19. Kaun Rice | 98. Don't know |
| 04. Rice | 19. Kaun Rice | 99. Unbranded |
| 04. Rice | 19. Kaun Rice | 999. Unknown |
| 04. Rice | 20. Miniket Rice | 01. Abul Kayer |
| 04. Rice | 20. Miniket Rice | 02. ACI Pure |
| 04. Rice | 20. Miniket Rice | 03. Akij |
| 04. Rice | 20. Miniket Rice | 04. Akij essential |
| 04. Rice | 20. Miniket Rice | 05. Anuwar |
| 04. Rice | 20. Miniket Rice | 06. Arong Natural |
| 04. Rice | 20. Miniket Rice | 07. Fahim |
| 04. Rice | 20. Miniket Rice | 08. Jora Machranga |
| 04. Rice | 20. Miniket Rice | 09. Minicate Premium Bulk |
| 04. Rice | 20. Miniket Rice | 10. Minicate Standard |
| 04. Rice | 20. Miniket Rice | 11. Monjur |
| 04. Rice | 20. Miniket Rice | 12. Mujammel |
| 04. Rice | 20. Miniket Rice | 13. Nabil |
| 04. Rice | 20. Miniket Rice | 14. New Momena |
| 04. Rice | 20. Miniket Rice | 15. Padmaa setu |
| 04. Rice | 20. Miniket Rice | 16. Pran |
| 04. Rice | 20. Miniket Rice | 17. Prima |
| 04. Rice | 20. Miniket Rice | 18. Rajanighanda |
| 04. Rice | 20. Miniket Rice | 19. Rashid |
| 04. Rice | 20. Miniket Rice | 20. Rupchanda |
| 04. Rice | 20. Miniket Rice | 21. Shortage Miniket |
| 04. Rice | 20. Miniket Rice | 22. Shukran |
| 04. Rice | 20. Miniket Rice | 23. Teer |
| 04. Rice | 20. Miniket Rice | 88. Other brands (specify) |
| 04. Rice | 20. Miniket Rice | 98. Don't know |
| 04. Rice | 20. Miniket Rice | 99. Unbranded |
| 04. Rice | 20. Miniket Rice | 999. Unknown |
| 04. Rice | 21. Nazirshail Rice | 01. ACI |
| 04. Rice | 21. Nazirshail Rice | 02. Akij essential |
| 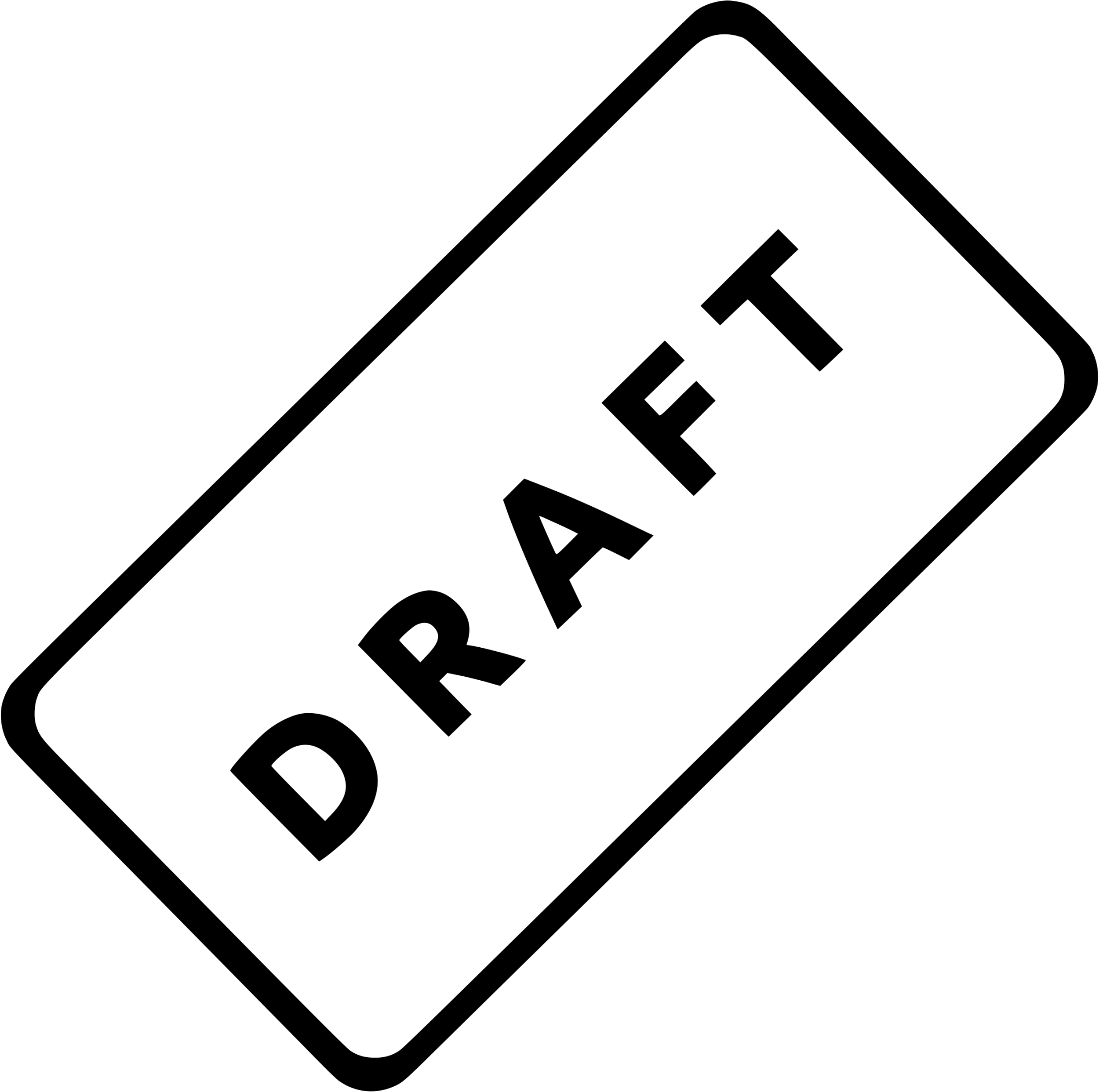04. Rice | 21. Nazirshail Rice | 03. Arong Natural |
| 04. Rice | 21. Nazirshail Rice | 04. Dhamrai |
| 04. Rice | 21. Nazirshail Rice | 05. Dhamrai Super Rice |
| 04. Rice | 21. Nazirshail Rice | 06. Fresh |
| 04. Rice | 21. Nazirshail Rice | 07. Khan Agro |
| 04. Rice | 21. Nazirshail Rice | 08. Kibria |
| 04. Rice | 21. Nazirshail Rice | 09. Pran |
| 04. Rice | 21. Nazirshail Rice | 10. Premium Bulk |
| 04. Rice | 21. Nazirshail Rice | 11. Rana |
| 04. Rice | 21. Nazirshail Rice | 12. Rupchanda |
| 04. Rice | 21. Nazirshail Rice | 13. Shukran |
| 04. Rice | 21. Nazirshail Rice | 14. Super Premium |
| 04. Rice | 21. Nazirshail Rice | 88. Other brands (specify) |
| 04. Rice | 21. Nazirshail Rice | 98. Don't know |
| 04. Rice | 21. Nazirshail Rice | 99. Unbranded |
| 04. Rice | 21. Nazirshail Rice | 999. Unknown |
| 04. Rice | 22. Paijam | 01. Anowar |
| 04. Rice | 22. Paijam | 02. Essential Auto Rice Mil |
| 04. Rice | 22. Paijam | 03. Kakoli |
| 04. Rice | 22. Paijam | 04. Molla |
| 04. Rice | 22. Paijam | 05. Noorjahan |
| 04. Rice | 22. Paijam | 06. Sonar Chabi |
| 04. Rice | 22. Paijam | 07. Swarna Dolphin Marka |
| 04. Rice | 22. Paijam | 88. Other brands (specify) |
| 04. Rice | 22. Paijam | 98. Don't know |
| 04. Rice | 22. Paijam | 99. Unbranded |
| 04. Rice | 22. Paijam | 999. Unknown |
| 04. Rice | 23. Puffed Rice | 01. Aafi |
| 04. Rice | 23. Puffed Rice | 02. ACI |
| 04. Rice | 23. Puffed Rice | 03. bpm |
| 04. Rice | 23. Puffed Rice | 04. Ifad |
| 04. Rice | 23. Puffed Rice | 05. Kishoyan |
| 04. Rice | 23. Puffed Rice | 06. Otithi |
| 04. Rice | 23. Puffed Rice | 07. Pran |
| 04. Rice | 23. Puffed Rice | 08. Prapti |
| 04. Rice | 23. Puffed Rice | 09. Rahul |
| 04. Rice | 23. Puffed Rice | 10. Ruchi |
| 04. Rice | 23. Puffed Rice | 11. Sera |
| 04. Rice | 23. Puffed Rice | 12. Shahin |
| 04. Rice | 23. Puffed Rice | 13. Sheha |
| 04. Rice | 23. Puffed Rice | 14. Shwapno |
| 04. Rice | 23. Puffed Rice | 15. Teer |
| 04. Rice | 23. Puffed Rice | 88. Other brands (specify) |
| 04. Rice | 23. Puffed Rice | 98. Don't know |
| 04. Rice | 23. Puffed Rice | 99. Unbranded |
| 04. Rice | 23. Puffed Rice | 999. Unknown |
| 04. Rice | 24. Red Binni Rice | 01. bpm |
| 04. Rice | 24. Red Binni Rice | 88. Other brands (specify) |
| 04. Rice | 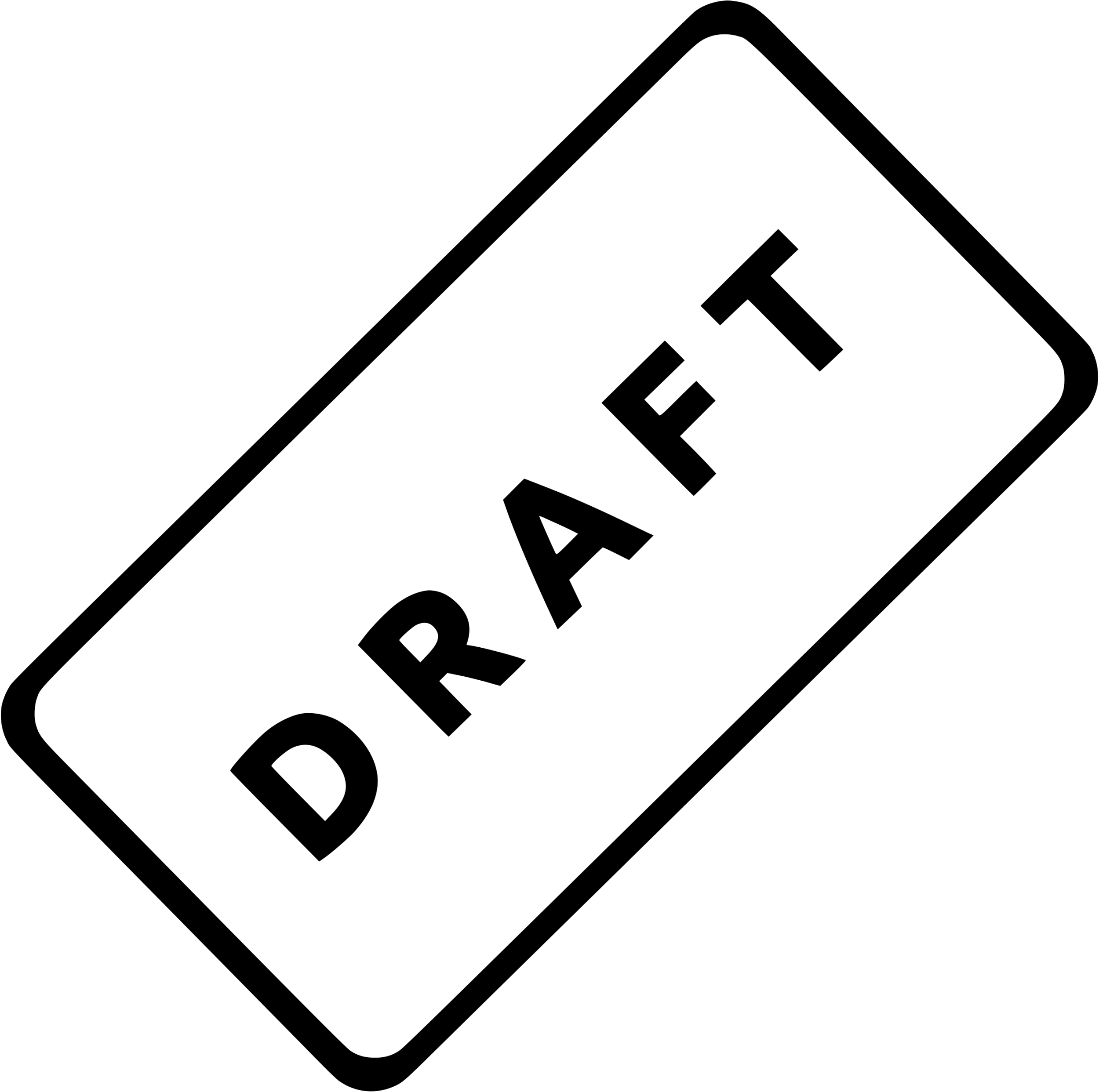24. Red Binni Rice | 98. Don't know |
| 04. Rice | 24. Red Binni Rice | 99. Unbranded |
| 04. Rice | 24. Red Binni Rice | 999. Unknown |
| 04. Rice | 25. Shampa Katari | 01. Manaf |
| 04. Rice | 25. Shampa Katari | 88. Other brands (specify) |
| 04. Rice | 25. Shampa Katari | 98. Don't know |
| 04. Rice | 25. Shampa Katari | 99. Unbranded |
| 04. Rice | 25. Shampa Katari | 999. Unknown |
| 04. Rice | 26. Unatrish (29) | 01. Dhamrai |
| 04. Rice | 26. Unatrish (29) | 02. Dhamrai Super |
| 04. Rice | 26. Unatrish (29) | 03. Dhamrai Super |
| 04. Rice | 26. Unatrish (29) | 04. Israt Super |
| 04. Rice | 26. Unatrish (29) | 05. Kishan |
| 04. Rice | 26. Unatrish (29) | 06. Kishan Special |
| 04. Rice | 26. Unatrish (29) | 07. Momena Special |
| 04. Rice | 26. Unatrish (29) | 08. New Momena Dhamrai Hasking Rice |
| 04. Rice | 26. Unatrish (29) | 88. Other brands (specify) |
| 04. Rice | 26. Unatrish (29) | 98. Don't know |
| 04. Rice | 26. Unatrish (29) | 99. Unbranded |
| 04. Rice | 26. Unatrish (29) | 999. Unknown |
| 04. Rice | 27. Untrish Haski Chaul | 01. Special Unatrish Haski Chaul |
| 04. Rice | 27. Untrish Haski Chaul | 88. Other brands (specify) |
| 04. Rice | 27. Untrish Haski Chaul | 98. Don't know |
| 04. Rice | 27. Untrish Haski Chaul | 99. Unbranded |
| 04. Rice | 27. Untrish Haski Chaul | 999. Unknown |
| 04. Rice | 28. Zirashail Rice | 01. ACI |
| 04. Rice | 28. Zirashail Rice | 02. ACI Pure |
| 04. Rice | 28. Zirashail Rice | 03. Khan |
| 04. Rice | 28. Zirashail Rice | 04. Nababodhu |
| 04. Rice | 28. Zirashail Rice | 05. New Momena |
| 04. Rice | 28. Zirashail Rice | 06. Rupchanda |
| 04. Rice | 28. Zirashail Rice | 07. Shakti |
| 04. Rice | 28. Zirashail Rice | 08. Shakti Premium |
| 04. Rice | 28. Zirashail Rice | 88. Other brands (specify) |
| 04. Rice | 28. Zirashail Rice | 98. Don't know |
| 04. Rice | 28. Zirashail Rice | 99. Unbranded |
| 04. Rice | 28. Zirashail Rice | 999. Unknown |


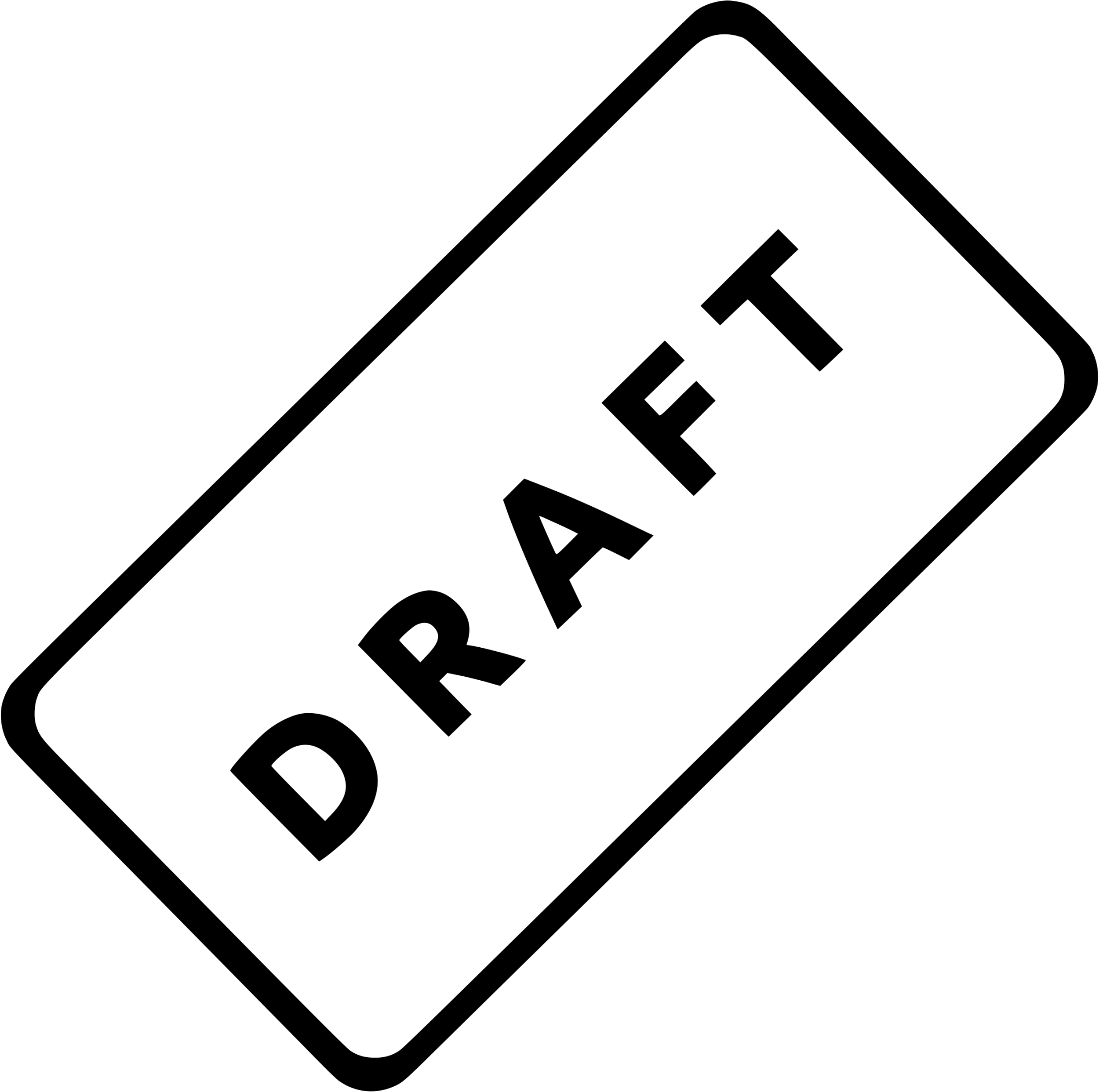
Module start time XX: XX

| **Interviewee reflection (IR)** | |
| --- | --- |
| IR.1 | How difficult was it to answer questions in this survey? |
| 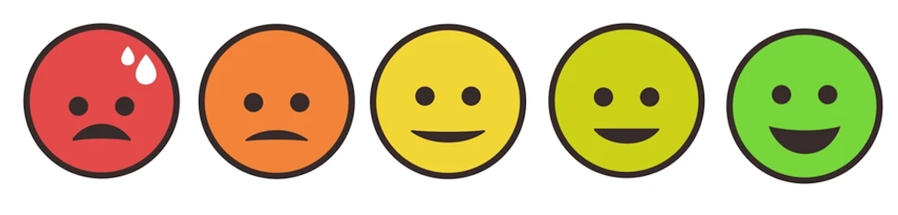    Very Hard Hard Average Easy Very easy | |
| IR.2 | How tiring was it to participate in this survey? |
| 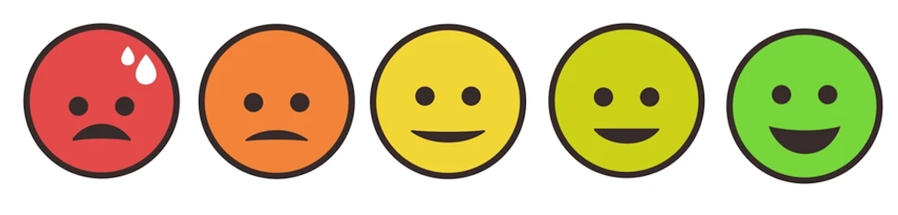  Very Hard Hard Average Easy Very easy | |

Module end time XX: XX

# **WOMAN’S QUESTIONNAIRE**

| **Women’s and consent (WC)** | | |
| --- | --- | --- |
| **Respondent: Each WRA 15-49y and married adolescent 10-14y in the household** | | |
| **Women’s consent:** *Administer consent form* | | |
| WC. 1 | Do you agree to participate in the study? | 1. No >> skip to end of survey 2. Yes |
| WC. 2 | Respondent Name | _____________  [Name of Respondent]  auto-filled |

Module start time XX: XX

| **Woman’s information (WI)** | | |
| --- | --- | --- |
| **Respondent: All WRA 15-49y and married adolescent 10-14y in the household** | | |
| CAPI instruction:   - Repeat this section for all names listed in S.N.2 and S.N 3 (married adolescent) of the respondent matrix. - Add Respondent ID ___ | | |
| Now, I would like to start by asking you about some information about yourself. | | |
| **Q. no** | **Q. label** | **Response** |
| WI.1 | What is your date of birth? | 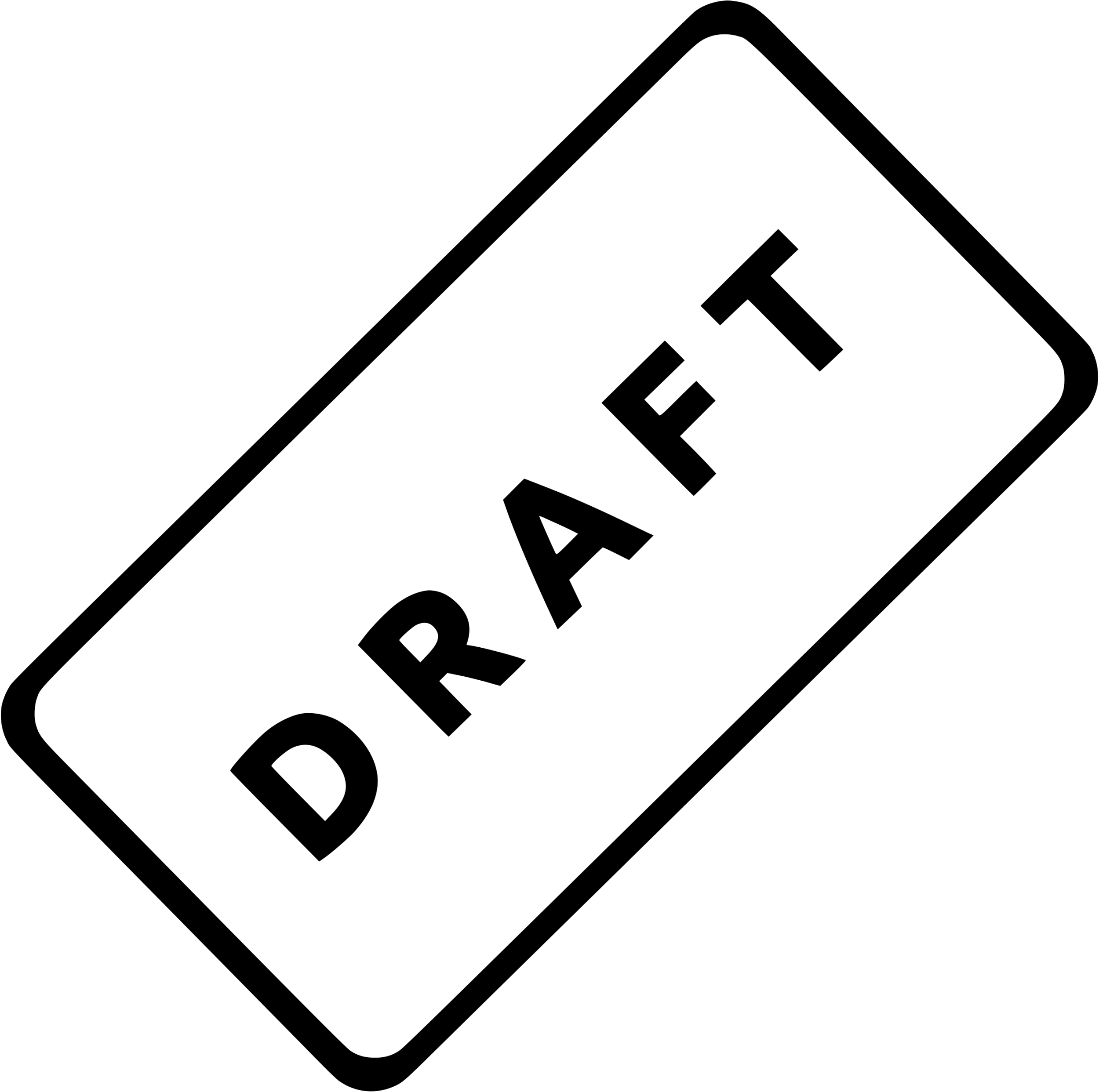  ___ ___/___ ___/ ___ ___ ___ ___  [ DD / [MM] / [YYYY]  98. Don’t know for DD and MM  9998. Don’t know for YYYY |
| WI.2 | How old were you at your last birthday? | ___ ___  (Record age in completed years)  (10-49 years)  98. Don’t know |
| WI.3 | What is your religion?  [SINGLE SELECT] | 1. Muslim 2. Hindu 3. Buddhist 4. Christian   88. Other  98. Don’t Know/ Don’t want to disclose |
| WI.4 | What is your ethnic group? | 1. Bengali 2. Bihari 3. Sawtal 4. Khasia 5. Rakhain 6. Bowm 7. Chak 8. Chakma 9. Khumi 10. Kheyang 11. Lusai/pankho 12. Marma 13. Mru(murong) 14. Tonchonga 15. Tripura 16. Bonojogi 17. Other (specify) |
| WI.5 | What is your highest completed education level or grade?  [SINGLE SELECT] | 1. No schooling 2. Literate without schooling 3. Pre-primary 4. Class 1 completed 5. Class 2 completed 6. Class 3 completed 7. Class 4 completed 8. Class 5 completed 9. Class 6 completed 10. Class 7 completed 11. 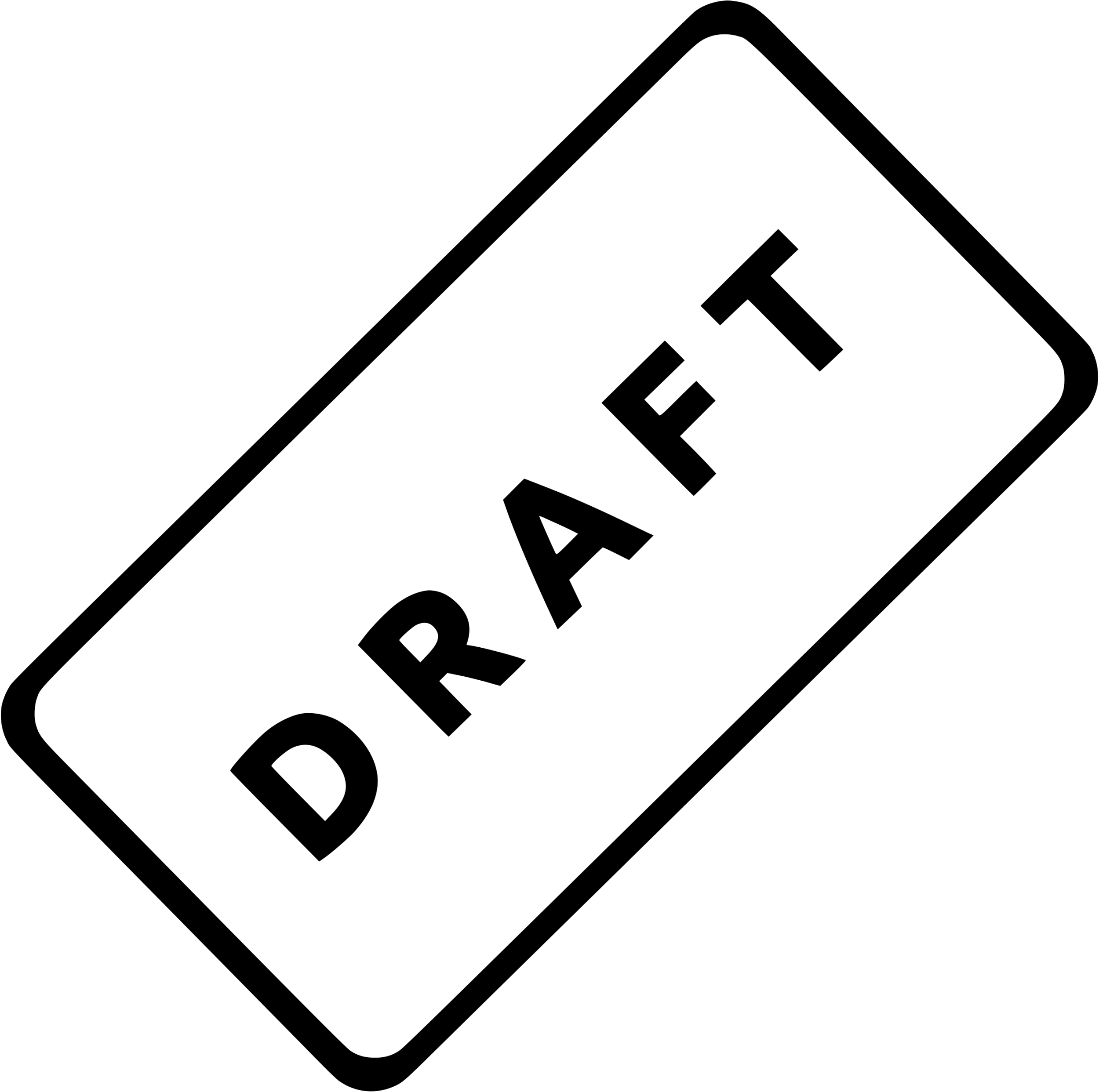Class 8 completed 12. Class 9 completed 13. Class 10 completed 14. Class 11 completed 15. Class 12 completed 16. Undergraduate degree completed 17. Postgraduate degree completed 18. Vocational studies completed 19. Other (Specify) 20. Don’t know |
| CAPI instruction: Skip WI.6 and WI.7 if completed age (WI.2) >19 | | |
| WI. 6 | At any time during the last school year did you attend school/college? | 1. No>>skip to WI.8 2. Yes |
| WI.7 | During the last school year, which class did  you attend? | ___ ___  (Record class/grade attended)  (1-12 class)  98. Don’t know |
| WI.8 | Aside from your own housework, have you done any work in the last seven days? | 1. No 2. Yes >>skip to WI.12 |
| WI.9 | As you know, some women take up jobs for which they are paid in cash or kind. Others sell things, have a small business or work on the family farm or in the family business. In the last seven days, have you done any of these things or any other work? | 1. No 2. Yes>>skip to WI.12 |
| WI.10 | Although you did not work in the last seven days, do you have any job or business from which you were absent for leave, illness, vacation, maternity leave, or any other such reason? | 1. No 2. Yes, on leave/ vacation >>skip to WI.12 3. Yes, had illness >>skip to WI.12 4. Yes, on maternity leave >>skip to WI.12 |
| WI.11 | Have you done any work in the last 12 months? | 1. No>>skip to WI.13 2. Yes |
| WI.12 | What is your occupation? That is, what kind of work do you mainly do? | 01. Student  02. Unpaid Household Work  03. Own enterprise/ business  04. Farming (Crop cultivation, livestock, aquaculture)  05. Casual farm labour (paid)  06. Casual non-farm labour (paid)  07. Self-employed  08. Salaried Employment  09. Retired with pension  10. Retired without pension  88. Other (Specify) |
| WI.13 | Do you own a mobile phone?  [SINGLE SELECT] | 1. No 2. Yes. Regular phone 3. Yes, smart phone   98. Don’t know |
| WI.14 | In the past 12 months, have you used the internet? | 1. No 2. Yes |
| WI.15 | How often do you use the internet? | 1. Almost every day 2. At least once a week 3. Less than once a week |
| WI.16 | Do you use a mobile phone for any financial transactions? | 1. No 2. Yes |
| WI.17 | How often do you watch TV? | 1. Almost every day 2. At least once a week 3. Less than once a week 4. Not at all |
| WI.18 | How often do you listen to radio? | 1. Almost every day 2. At least once a week 3. Less than once a week 4. Not at all |
| WI.19 | Are you a member of a mother’s group? | 1. 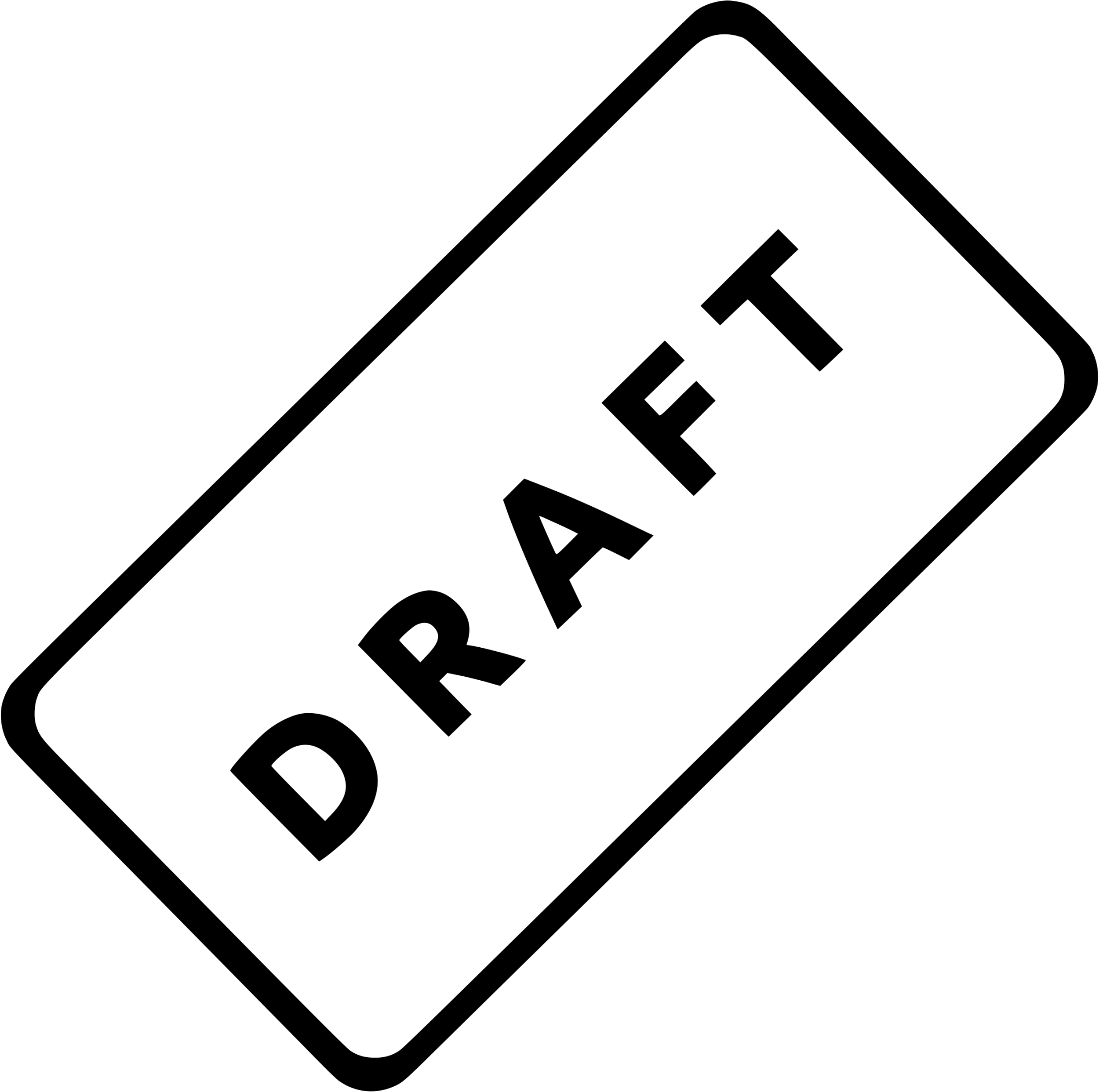No 2. Yes |

Module end time XX: XX

Module start time XX: XX

| **Barriers to health care (BC)** | | |
| --- | --- | --- |
| **Respondent: All WRA 15-49y and married adolescent 10-14y in the HH** | | |
| CAPI instruction:   - Repeat this section for all names listed in S.N.2 and S.N 3(married adolescent) of the respondent matrix. - Add Respondent ID ___ | | |
| Now, I would like to ask you some questions related to your experience with health care and assistance when you are sick | | |
| **Q. no** | **Q. label** | **Response** |
| BC.1 | **Barriers to healthcare: permission**  Many different factors can prevent women from getting medical advice or treatment for themselves. When you are sick and want to get medical advice or treatment, is getting permission to go to the doctor a problem or challenge for you? | 1. Not a problem 2. Small problem 3. Big problem |
| BC.2 | **Barriers to healthcare: cost**  Many different factors can prevent women from getting medical advice or treatment for themselves. When you are sick and want to get medical advice or treatment, is getting money needed for advice or treatment a problem or challenge for you? | 1. Not a problem 2. Small problem 3. Big problem |
| BC.3 | **Barriers to healthcare: distance**  Many different factors can prevent women from getting medical advice or treatment for themselves. When you are sick and want to get medical advice or treatment, is the distance to the health facility a problem or challenge for you? | 1. 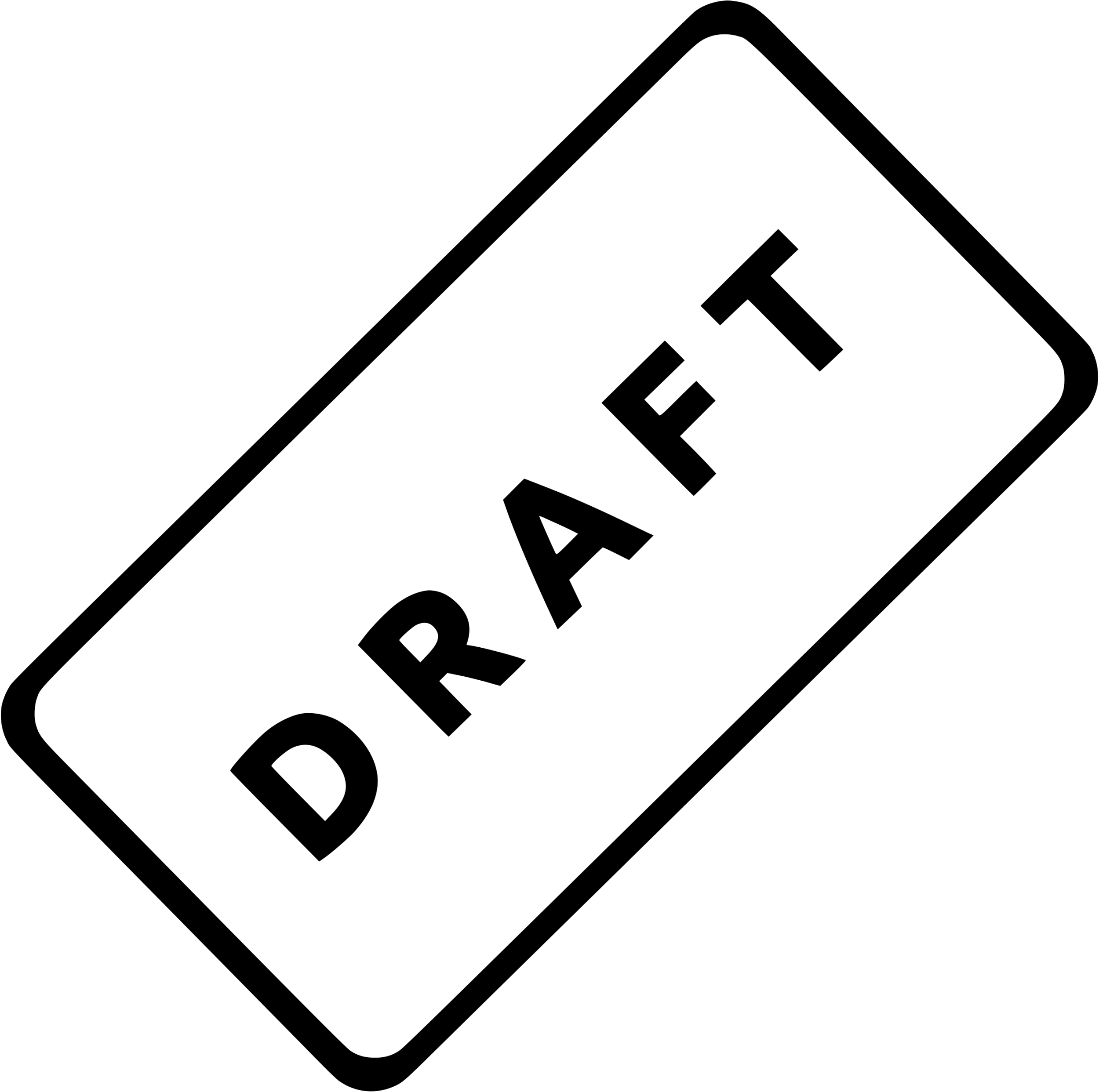Not a problem 2. Small problem 3. Big problem |
| BC.4 | **Barriers to healthcare: going alone**  Many different factors can prevent women from getting medical advice or treatment for themselves. When you are sick and want to get medical advice or treatment, is going alone a problem or challenge for you? | 1. Not a problem 2. Small problem 3. Big problem |
| BC.5 | Are there any adults in your household who can help you when you are sick? | 1. No 2. Yes |
| BC.6 | Are there any adult women in your household who can help if your child is sick? | 1. No 2. Yes 3. Not married/Don’t have a child |

Module end time XX: XX

Module start time XX: XX

| **Birth history (BH)** | | |
| --- | --- | --- |
| **Respondent: All WRA 15-49y and married adolescent 10-14y in the HH** | | |
| CAPI instruction:   - Repeat this section for all names listed in S.N.2 and S.N 3 (married adolescent) of the respondent matrix. - Add Respondent ID ___ | | |
| I would like to now ask details of your marital status. | | |
| **Q. no** | **Q. label** | **Response** |
| BH.1 | Are you now married, separated, deserted, divorced,  widowed, or have you never been married? | 1. Currently married 2. Separated 3. Deserted 4. Divorced 5. Widowed 6. Never married >>skip to WR.1 |
| BH.1.1 | How old were you when you first got married? | ___ ___  (Record age in completed years)  (10-49 years)   1. Don’t know |
| BH.1.2 | INSTRUCTION: IDENTIFY THE WOMAN’S HUSBAND AND CONFRIM HIS NAME | (Drop down of household member) |
| Now I would like to ask about all the births you have had during your life. By birth we mean both live and stillbirths. Livebirths are  All children who were born alive (that is, who showed signs of life by crying, breathing, or moving) even if they survived only for a few minutes. This includes any children who may not live with you or are no longer alive. Stillbirth means children who were born dead (that is, who showed no signs of life by crying, breathing or moving). We know these questions might be hard to answer, if you feel uncomfortable, please let us know and we can move to the next question. | | |
| BH.2 | Have you ever given birth? | 1. 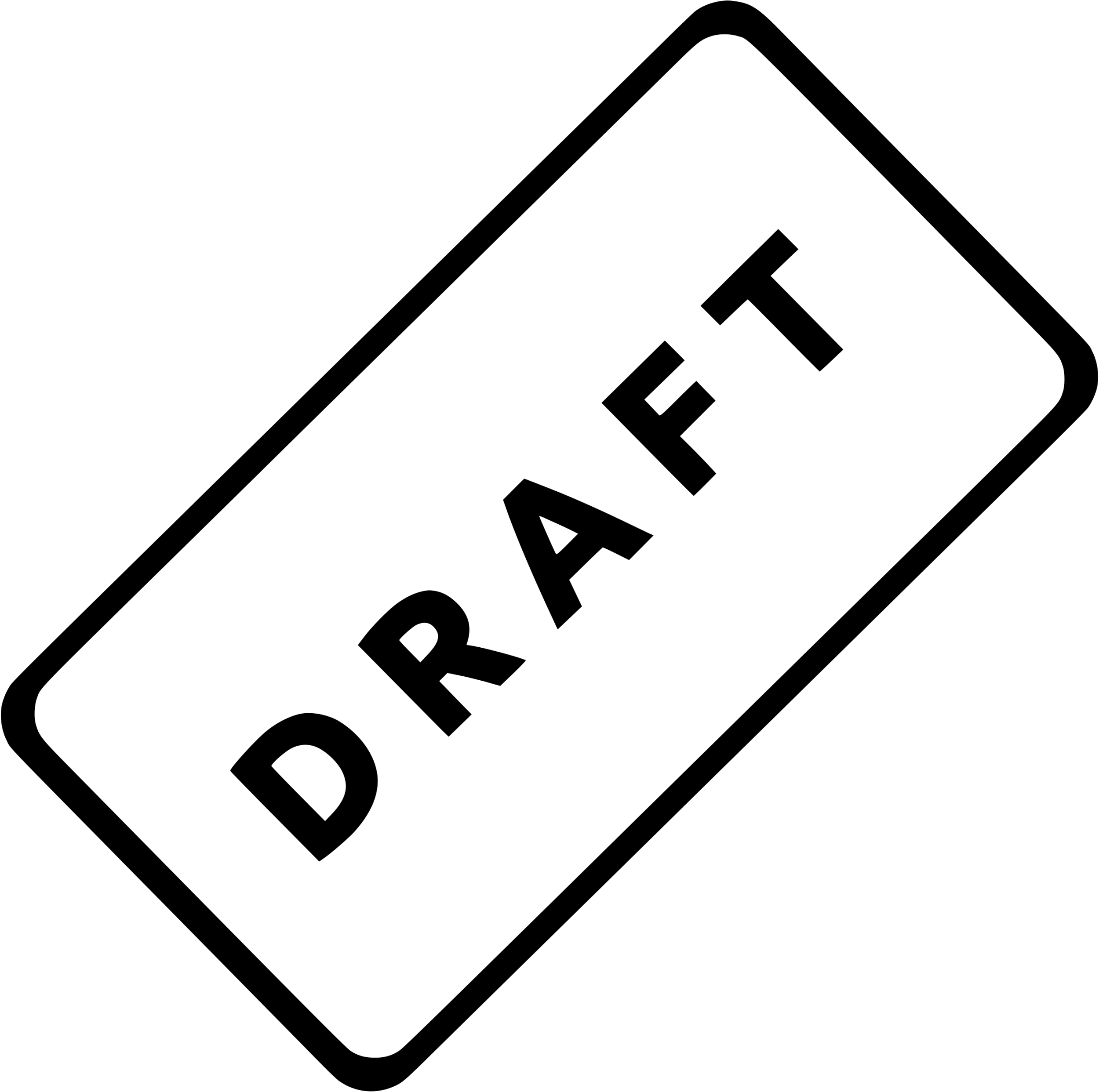No>>skip to BH.19 2. Yes |
| BH.3 | How old were you when you gave birth for the first time? | ___ ___  (Record age in completed years)  (10-49 years)   1. Don’t know |
| BH.3.1 | How many children have you given birth (live birth) to so far in your lifetime, even those who are not currently alive or do not live with you?  INSTRUCTION: DO NOT INCLUDE ADOPTED CHILDREN | ___ ___  (Record no. births)  (1-10 births) |
| BH.3.2 | Women sometimes have a pregnancy that does not result in a live birth. For example, can be born dead. Have you ever had a pregnancy that did not end in a live birth?  INSTRUCTION: IF MOTHER EXPRESSES DISCOMFORT, CHECK IF SHE IS OKAY TO CONTINUE. | ___ ___  (Record no. births)  (1-10 births) |
| BH.3.3 | INSTRUCTION: SUM TOTAL BIRTHS  CAPI Instruction: Add births in BH.3.1 and BH.3.2 | **Auto filled**  ___ ___  (Autofill total no. births)  (1-20 births) |
| BH.4 | Just to make sure that I have this right: you have had in total [INSERT total births from BH.3.3] births during your life. Is that  correct? | 1. No>>Probe and correct BH.3.1 and BH.3.2 2. Yes |
| CAPI Instruction: Skip following questions if BH.2=01; Repeat following questions based on total births in BH.4 | | |
| INSTRUCTION: RECORD TWINS AND TRIPLETS ON SEPARATE ROWS. | | |
| Now, I would like to ask you about each of your children starting with the youngest. | | |
| BH.5 | Tell me the name of the child.  (Start from the youngest), | (Drop down of household member)  In case of an unnamed child or if the name is not available in the roster enter following   1. LB-unnamed (Live birth but currently not alive and was unnamed) 2. SB-unnamed (Stillborn) 3. Live birth but currently not living in the household (enter name) |
| BH.6 | Was this birth twins or triplet?  (CAPI Instruction- If yes, repeat BH.5 to BH.11 for each child) | 1. No 2. Yes>>Repeat BH.5 |
| BH.7 | On what day, month and year was [INSERT NAME FROM BH.5] born?  INSTRUCTION: CHECK BIRTH REGISTRATION OR IMMUMMNIZATION CARD. IF NOT AVAILABLE, USE EVENTS CALENDAR TO HELP IDENTIFY DATE. | ___ ___ /___ ___ /___ ___ ___ ___  (DD/MM/YYYY)  98. Don’t know only for DD |
| BH.8 | Was [INSERT NAME FROM BH.5] livebirth or stillbirth?  INSTRUCTION: KEEP IN MIND FOLLOWING DEFINITIONS  Live Birth: This refers to a birth in which the infant shows any sign of life by crying, breathing, or moving) even if they survived only for a few minutes  Stillbirth: This refers to a birth in which the infant shows no signs of life by crying, breathing or moving | 1. Livebirth 2. Stillbirth>>skip to BH.11 |
| BH.9 | Is [INSERT NAME FROM BH.5] currently alive? | 1. No>>skip to BH.11 2. Yes |
| BH.10 | Does [INSERT NAME FROM BH.5] live with you? | 1. No 2. Yes |
| BH.11 | 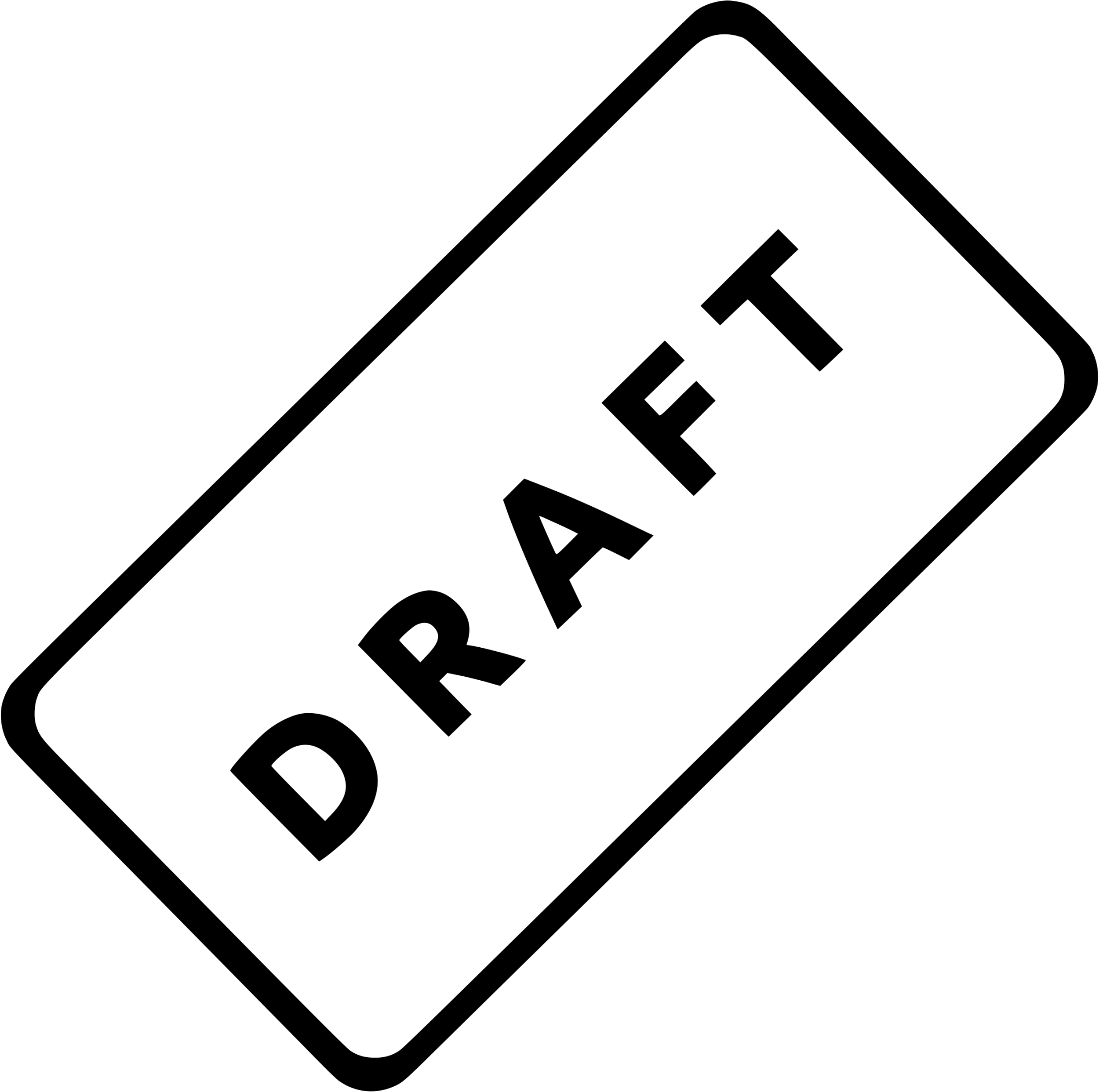Is [INSERT NAME FROM BH.5] boy or a girl? | 1. Boy 2. Girl |
| BH12 | CAPI Instruction- Calculate the most recent live birth in the last two years based on Date of Birth (BH.7) and birth outcome (BH.8=1)  Note- This is either 0 or 1 | **Auto filled**  ____  (Auto fill no. most recent live birth) |
| BH.12.1 | Most recent live birth in the last 2 years  CAPI Instruction- List the name of the most recent live birth in the last 2 years.  Note- This will only have one name. |  |
| BH.13 | CAPI Instruction- Calculate the most recent still birth in the last two years based on Date of Birth (BH.7) and birth outcome (BH.8=2)  Note- This is either 0 or 1 | **Auto filled**  ____  (Auto fill no. most recent still birth) |
| BH.13.1 | Most recent still birth in the last 2 years  CAPI Instruction- List the name of the most recent still birth in the last 2 years.  Note - This will only have one name or DOB. |  |
| BH.14 | CAPI Instruction- Calculate the number of prior livebirths in the last two years based on Date of Birth (BH.7) and birth outcomes (BH.8=1) | **Auto filled**  ___  (Auto fill no. prior livebirths in the last two years) |
| BH.14.1 | Prior livebirths in the last 2 years  CAPI Instruction- List the names of prior livebirths in the last 2 years, do not include the most recent livebirth.  Start from the most recent prior livebirths |  |
| BH.15 | CAPI Instruction- Calculate the number of prior stillbirths in the last two years based on Date of Birth (BH.7) and birth outcomes (BH.8=2) | 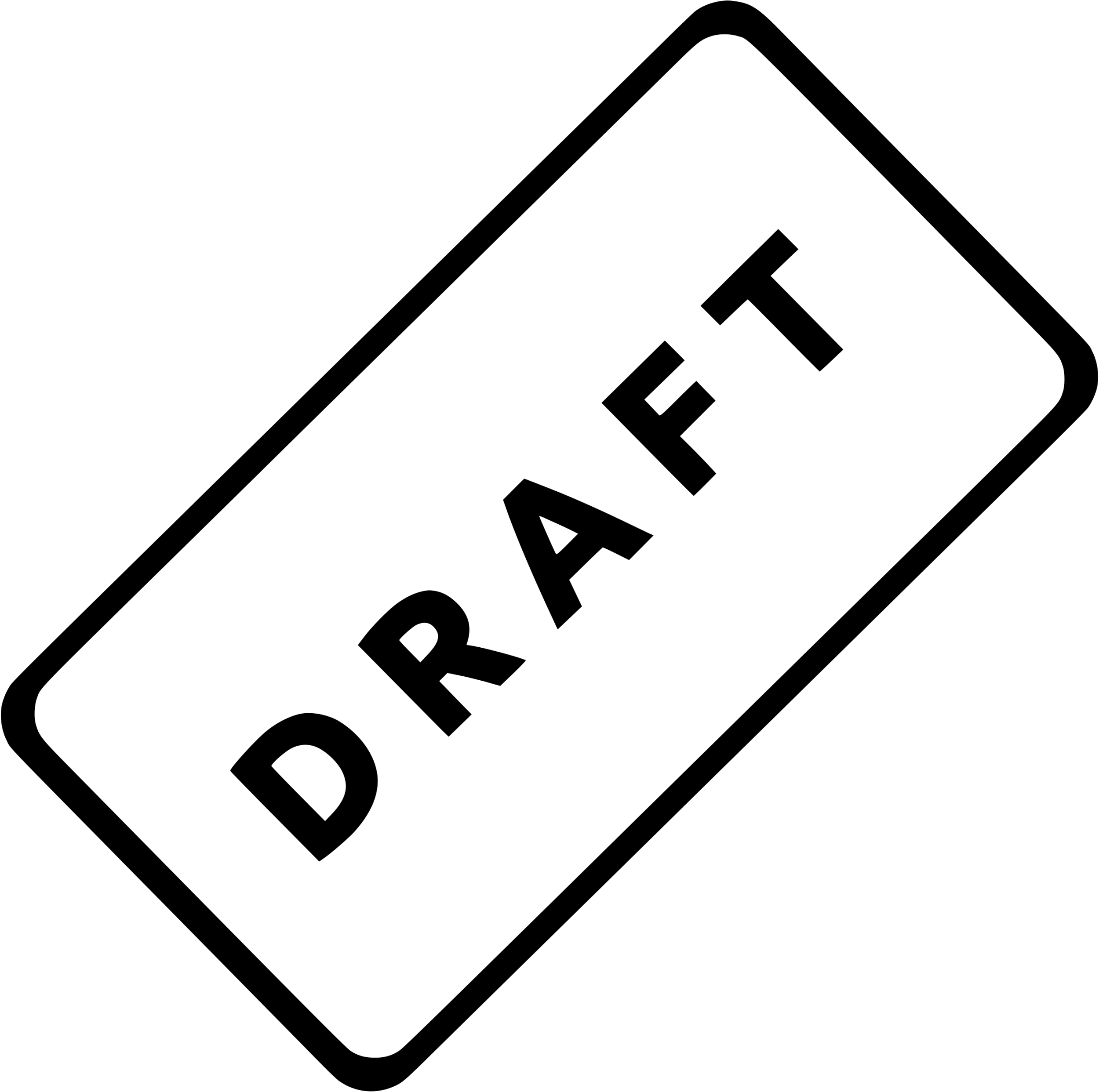**Auto filled**  ___  (Auto fill no. prior stillbirths in the last two years) |
| BH.15.1 | Prior stillbirths in the last 2 years  CAPI Instruction- List the names of the prior stillbirths in the last 2 years, do not include the most recent stillbirth  Start from the most recent prior stillbirth |  |
| BH.16 | CAPI Instruction- Calculate the number of children 24-59 months if BH.9=02 and BH.10=02 based on Date of Birth (BH.7) | **Auto filled**  ___  (Auto fill no. children 24-59 months) |
| BH.16.1 | CAPI Instruction- List the name of the children 24-59 months if BH.9=02 and BH.10=02 based on Date of Birth (BH.7)  Start from the youngest |  |
| BH.17 | CAPI Instruction- Calculate the number of children 5-9 years if BH.9=02 and BH.10=02 based on Date of Birth (BH.7) | **Auto filled**  ___  (Auto fill no. children 5-9 years) |
| BH.17.1 | CAPI Instruction List the name of the children 5-9 years if BH.9=02 and BH.10=02  Start from the youngest |  |
| BH.18 | CAPI Instruction- Calculate the number of children 10-19 years if BH.9=02 and BH.10=02 based on Date of Birth (BH.7) | **Auto filled**  ___  (Auto fill no. children 10-19 years) |
| BH.18.1 | CAPI Instruction- List the name of the children 10-19 years if BH.9=02 and BH.10=02  Start from the youngest |  |
| BH.19 | Are you currently pregnant? | 1. No 2. Yes >> skip to CP.1   98. Don’t know |

Module end time XX: XX

Module start time XX: XX

| **Women of Reproductive Age - General (WR)** | | |
| --- | --- | --- |
| **Respondent: All non-pregnant WRA 15-49y and all non-pregnant married adolescent 10-14y in the HH** | | |
| CAPI instructions:   - Repeat this section for all names listed in S.N.2 and S.N 3 (married adolescent) of the respondent matrix - Add Respondent ID ___ - Skip if BH.19=02 | | |
| Now, I would like to ask you about general health and nutrition services you may have received recently. | | |
| **Q. no** | **Q. label** | **Response** |
| WR.1 | In the last three months, were you given, or did you buy any iron tablets or syrup that contains iron?  INSTRUCTION: SHOW VISUAL AID OF MMS TABLET & FULLCARE, COMMON TYPES OF MULTIPLE MICRONUTRIENT SUPPLEMENTS, & COMMON TYPES OF IRON/IFA | 1. No>>skip to WR.5 2. Yes 3. Don’t know>>skip to WR.5 |
| WR.2 | 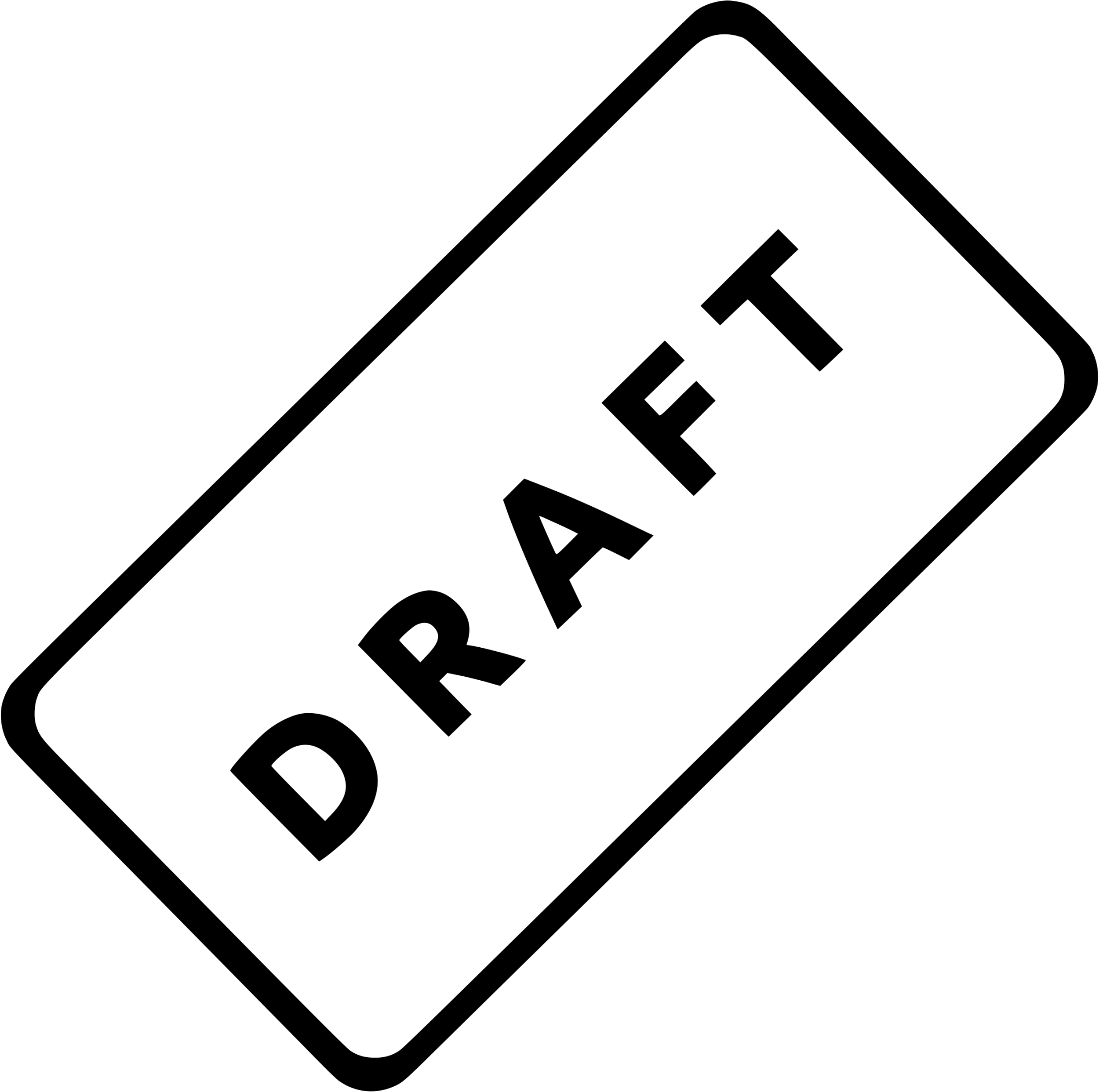In the last three months, were you given or did you buy any of the following? :   1. MMS TABLET OR FULLCARE?   INSTRUCTION: SHOW VISUAL AID OF MMS TABLET & FULLCARE   1. SUPPLEMENTS WITH MULTIPLE MICRONUTRIENTS?   INSTRUCTION: SHOW VISUAL AID OF COMMON TYPES OF MULTIPLE MICRONUTRIENT SUPPLEMENTS.  READ ALOUD: PLEASE THINK ABOUT THESE AND SIMILAR PRODUCTS; THE PICTURES ARE JUST EXAMPLES OF SUPPLEMENTS CONTAINING MMS   1. IRON TABLET OR SYRUPS?   INSTRUCTION: SHOW VISUAL AID OF COMMON TYPES OF IRON/IFA  READ ALOUD: PLEASE THINK ABOUT THESE AND SIMILAR PRODUCTS; THE PICTURES ARE JUST EXAMPLESOF TABLETS CONTAINING IRON/IFA  [PROBE: ANYTHING ELSE?]  [MULTI SELECT] | 1. MMS tablet, or FullCare, 2. Supplements with multiple micronutrients 3. Iron tablet/Iron folic acid 4. Other (specify) 5. Don’t know |
| WR.3 | How many days did you take [INSERT NAME OF PRODUCT FROM WR.2] in the last **month**?  [If answer is not numeric probe for proximate number of days]  INSTRUCTION: SHOW VISUAL AID  CAPI Instruction: Repeat this question for all the chosen responses in WR.2 | ___ ___  (Record no. of days)  (1-31 days)   1. Don’t know |
| WR.4 | Where did you get [INSERT TYPE FROM WR.2] from?  CAPI Instruction: Repeat this question for all the chosen responses in WR.2  PROBE: ANYWHERE ELSE?]  [MULTI SELECT]  INSTRUCTION: SHOW VISUAL AID | 1. Medical college hospital 2. Specialized govt hospital 3. District hospital 4. MCWC 5. Upazila health complex 6. UH & family welfare center 7. Community clinic 8. Satellite Clinic/EPIU outreach 9. NGO sector (NGO clinics, NGO worker) 10. Private medical college 11. Private hospital 12. Private clinic 13. Qualified doctor chamber 14. Unqualified doctor chamber 15. Pharmacy 16. Others (specify)   98. Don’t know |
| WR.5 | Did you receive tablets to treat intestinal worms in the last 6 months? | 1. 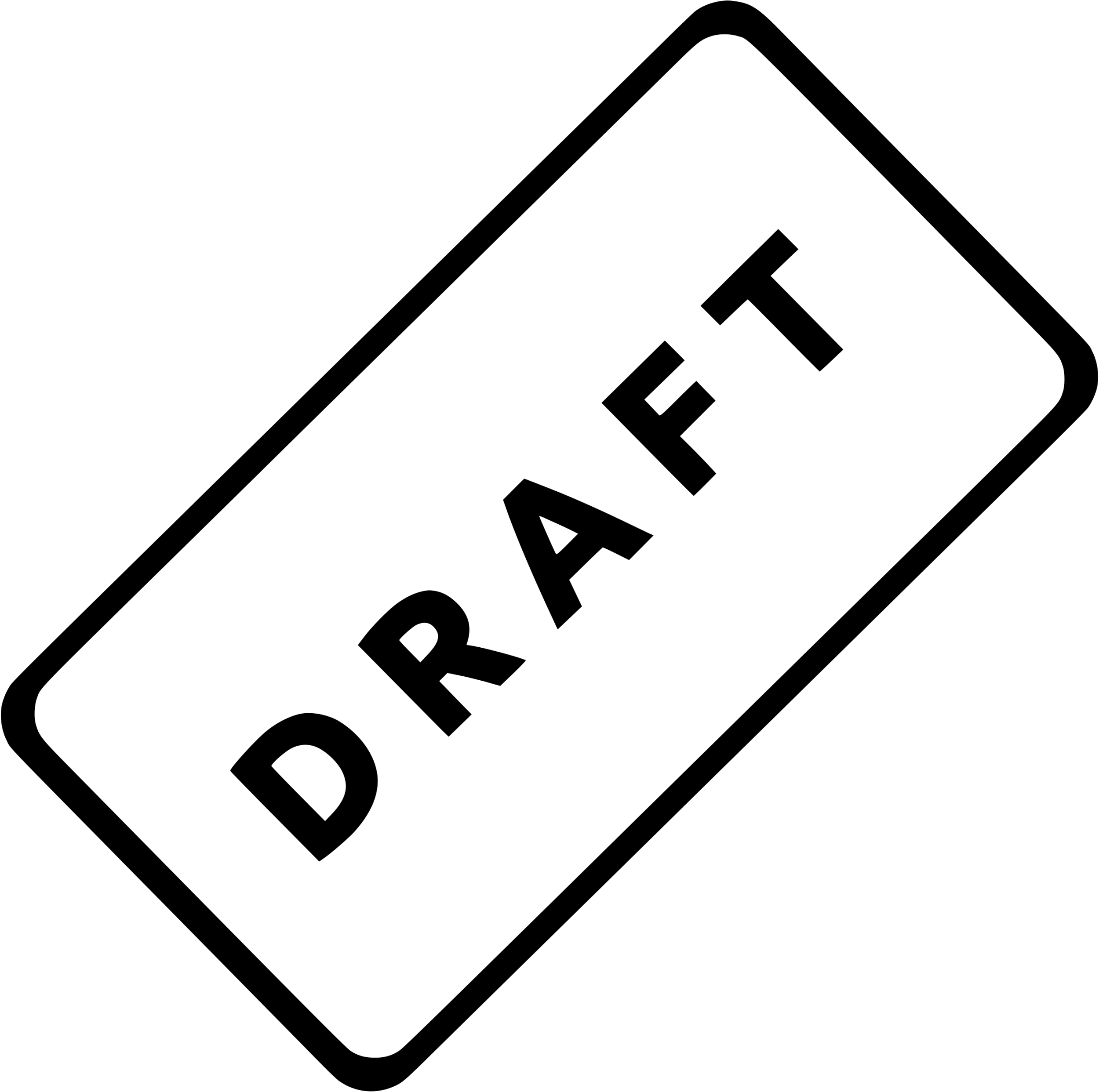No 2. Yes 3. Don’t know |
| I would like to talk about family planning- the various ways or methods that a couple can use to delay or avoid a pregnancy. | | |
| CAPI Instruction: Skip WR.6 and WR.7 if BH.1=2 or 3 or 4 or 5 or 6 and BH.19=2 | | |
| WR.6 | Are you currently using contraception? | 1. No>>skip to WR.8 2. Yes |
| WR.7 | What method are you using?  [PROBE: ANYTHING ELSE?]  [MULTI SELECT] | 1. Female sterilization 2. Male sterilization 3. IUD (Copper-T) 4. Injectables 5. Implants 6. Pill 7. Condom 8. Female condom 9. Emergency contraception 10. Standard days method 11. Lactational amenorrhea method 12. Rhythm method 13. Withdrawal 14. Other modern method 15. Other traditional method   98. Don’t know |
| CAPI Instruction: Skip WR.8 if BH.1=6 | | |
| WR.8 | Would you say that using (or not using) contraception is mainly your decision, mainly your (husband's/ partner's) decision, or did you both decide together? | 1. Mainly mine 2. Mainly husband/partner 3. Joint decision 4. Other (specify)   98. Don’t know |

Module end time XX: XX

Module start time XX: XX

| **Current pregnancy (CP) interventions** | | |
| --- | --- | --- |
| **Respondent: All currently pregnant WRA 15-49y or currently pregnant adolescent 10-14y** | | |
| CAPI instructions:   - Repeat this section for all names listed in S.N.2 and S.N 3 (married adolescent) of the respondent matrix.   Add Respondent ID ___   - Skip if BH.19=01 or 98 | | |
| Now, I would like to ask about the various health and nutrition services you may have received during your current pregnancy. | | |
| **Q. no** | **Q. label** | **Response** |
| CP.1 | When was your last menstrual date? | ___ ___ /___ ___ /___ ___ ___ ___  (DD/MM/YYYY)  98. Don’t know for DD and MM  9998. Don’t know for YYYY |
| CP.2 | How many months into your pregnancy you are? | ___  (Record no. months)  (0-9 months)  98. Don’t know |
| CP.3 | Have you seen anyone for antenatal care for this pregnancy? | 1. No>> skip to CP.8 2. Yes |
| CP.4 | Whom did you see?  [PROBE: ANYONE ELSE?]  [MULTI SELECT] | 1. 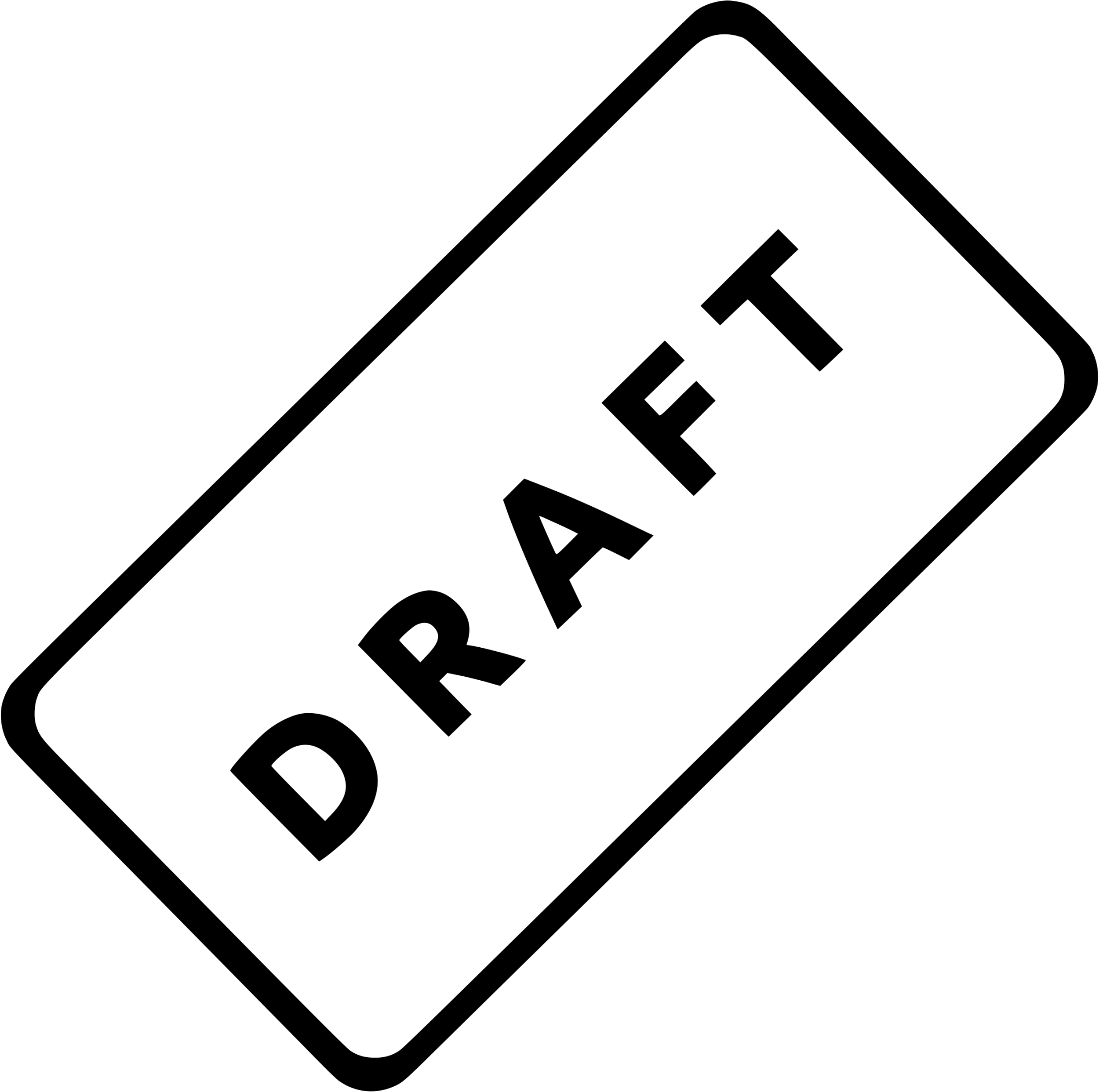Doctor/Nurse/Midwife 2. Paramedic 3. Family Welfare Visitor (FWV) 4. Community Skilled Birth Assistant (CSBA) 5. Sub-Assistant Community Medical Assistant (SACMO) 6. Community Health Care Provider (CHCP) 7. Health assistant 8. Family welfare assistant 9. NGO workers 10. Trained TBA 11. Untrained TBA 12. Unqualified doctor 13. Others (Specify) |
| CP.5 | Where did you receive antenatal care for this pregnancy?  [PROBE: ANYWHERE ELSE?]  [MULTI SELECT] | 1. Home 2. Medical college hospital 3. Specialized govt hospital 4. District hospital 5. MCWC 6. Upazila health complex & family welfare center 7. Union Health & family welfare center 8. Community clinic 9. Satellite Clinic/EPIU outreach 10. NGO sector 11. Private medical college 12. Private hospital 13. Private clinic 14. Qualified doctor chamber 15. Unqualified doctor chamber 16. Pharmacy 17. Others (Specify) |
| CP.6 | How many months pregnant were you when you first received antenatal care for this pregnancy? | ___  (Record no. months)  (1-9 months)  98. Don’t know |
| CP.7 | How many times did you receive antenatal care by any healthcare provider (such as doctor, nurse, paramedics, health worker) during this pregnancy? | ___ ___  (Record no. times)  (1-20 times)   1. Don’t know |
| CP.8 | During this pregnancy, were you given, or did you buy any tablets that contain calcium?  INSTRUCTION: SHOW VISUAL AID OF CALCIUM  READ ALOUD: PLEASE THINK ABOUT THESE AND SIMILAR PRODUCTS; THE PICTURES ARE JUST EXAMPLES OF TABLETS CONTAINING CALCIUM | 1. No>>skip to CP.13 2. Yes 3. Don’t know>>skip to CP.13 |
| CP.9 | How many months pregnant were you when you first started taking tablets that contain calcium during this pregnancy?  INSTRUCTION: SHOW VISUAL AID OF CALCIUM | ___  (Record no. months)  (0-9 months  98. Don’t know |
| CP.10 | How many days did you take tablets that contain calcium in the last **month**?  [If answer is not numeric probe for proximate number of days]  INSTRUCTION: SHOW VISUAL AID OF CALCIUM | ___ ___  (Record no. days)  (0-31 days)  98. Don’t know |
| CP.11 | Where have you gotten tablets that contain calcium from during this pregnancy?  [PROBE: ANYWHERE ELSE?]  [MULTI SELECT]  INSTRUCTION: SHOW VISUAL AID OF CALCIUM | 1. 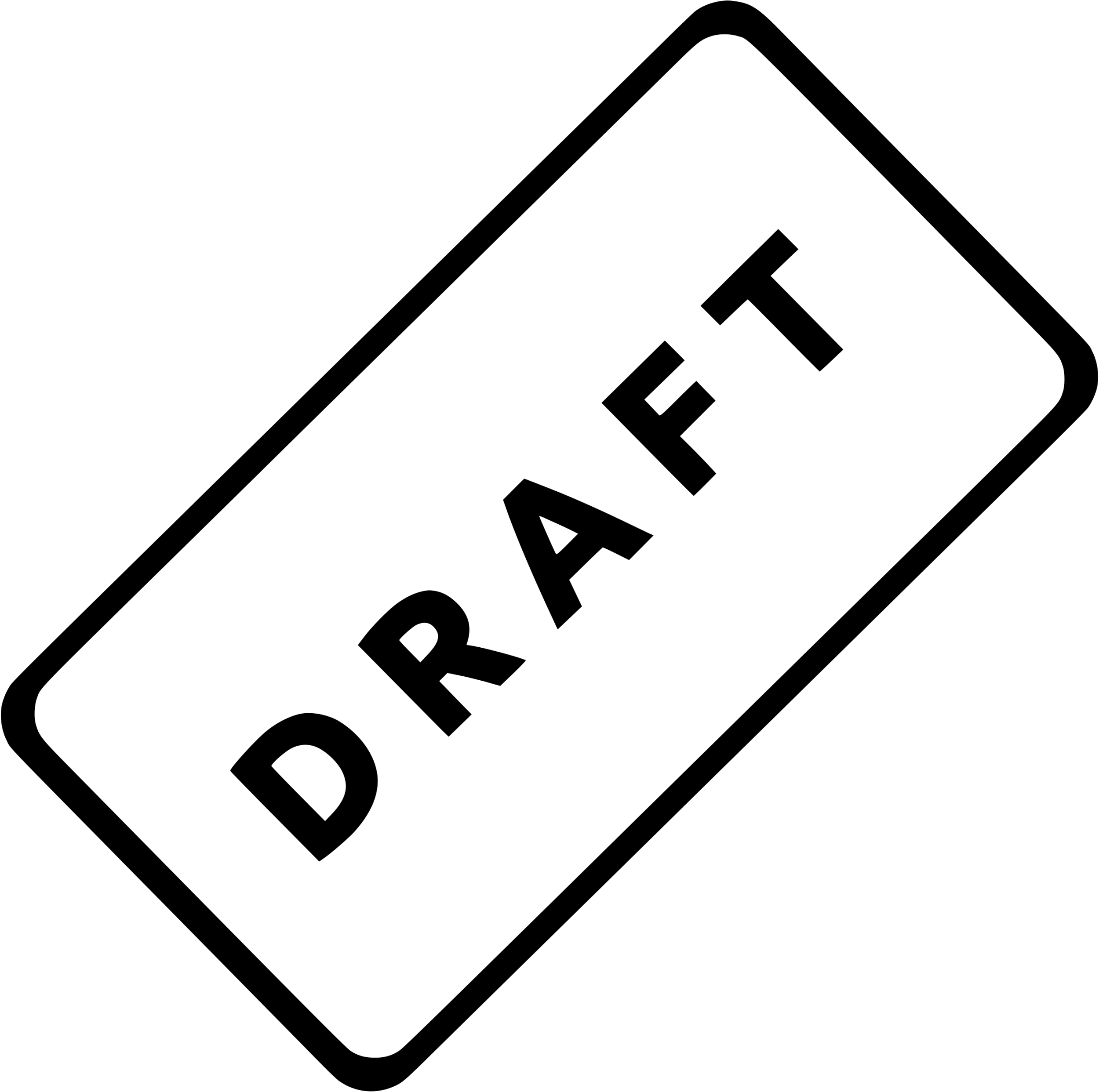Medical college hospital 2. Specialized govt hospital 3. District hospital 4. MCWC 5. Upazila health complex & family welfare center 6. Union Health & family welfare center 7. Community clinic 8. Satellite Clinic/EPIU outreach 9. NGO sector (NGO clinics, NGO worker) 10. Blue star provider 11. Private medical college 12. Private hospital 13. Private clinic 14. Qualified doctor chamber 15. Unqualified doctor chamber 16. Pharmacy 17. Retail shop 18. Community health worker 19. Family/Friend/Neighbor 20. Others (specify)   98. Don’t know |
| CP.12 | During this pregnancy has any health care provider or a health worker, or a nutrition worker talked with you about the following? |  |
| CP.12.1 | Taking tablets containing calcium | 1. No 2. Yes 3. Don’t know |
| CP.12.2 | Benefits of tablets containing calcium | 1. No 2. Yes 3. Don’t know |
| CP.12.3 | Side effects of tablets containing calcium | 1. No 2. Yes 3. Don’t know |
| CP.13 | During this pregnancy, were you given, or did you buy any tablet or syrup that contains iron?  INSTRUCTION: SHOW VISUAL AID OF MMS TABLET & FULLCARE, COMMON TYPES OF MULTIPLE MICRONUTRIENT SUPPLEMENTS & COMMON TYPES OF IRON/IFA | 1. No>>skip to CP.18 2. Yes 3. Don’t know>>skip to CP.18 |
| CP.14 | During this pregnancy, were you given or did you buy any of the following:   1. MMS TABLET OR FULLCARE?   INSTRUCTION: SHOW VISUAL AID OF MMS TABLET & FULLCARE   1. SUPPLEMENTS WITH MULTIPLE MICRONUTRIENTS?   INSTRUCTION: SHOW VISUAL AID OF COMMON TYPES OF MULTIPLE MICRONUTRIENT SUPPLEMENTS.  READ ALOUD: PLEASE THINK ABOUT THESE AND SIMILAR PRODUCTS; THE PICTURES ARE JUST EXAMPLES OF SUPPLEMENTS CONTAINING MMS  c)IRON TABLET OR SYRUPS?  INSTRUCTION: SHOW VISUAL AID OF COMMON TYPES OF IRON/IFA  READ ALOUD: PLEASE THINK ABOUT THESE AND SIMILAR PRODUCTS; THE PICTURES ARE JUST EXAMPLESOF TABLETS CONTAING IRON/IFA  [PROBE: ANYTHING ELSE?]  [MULTI SELECT] | 1. 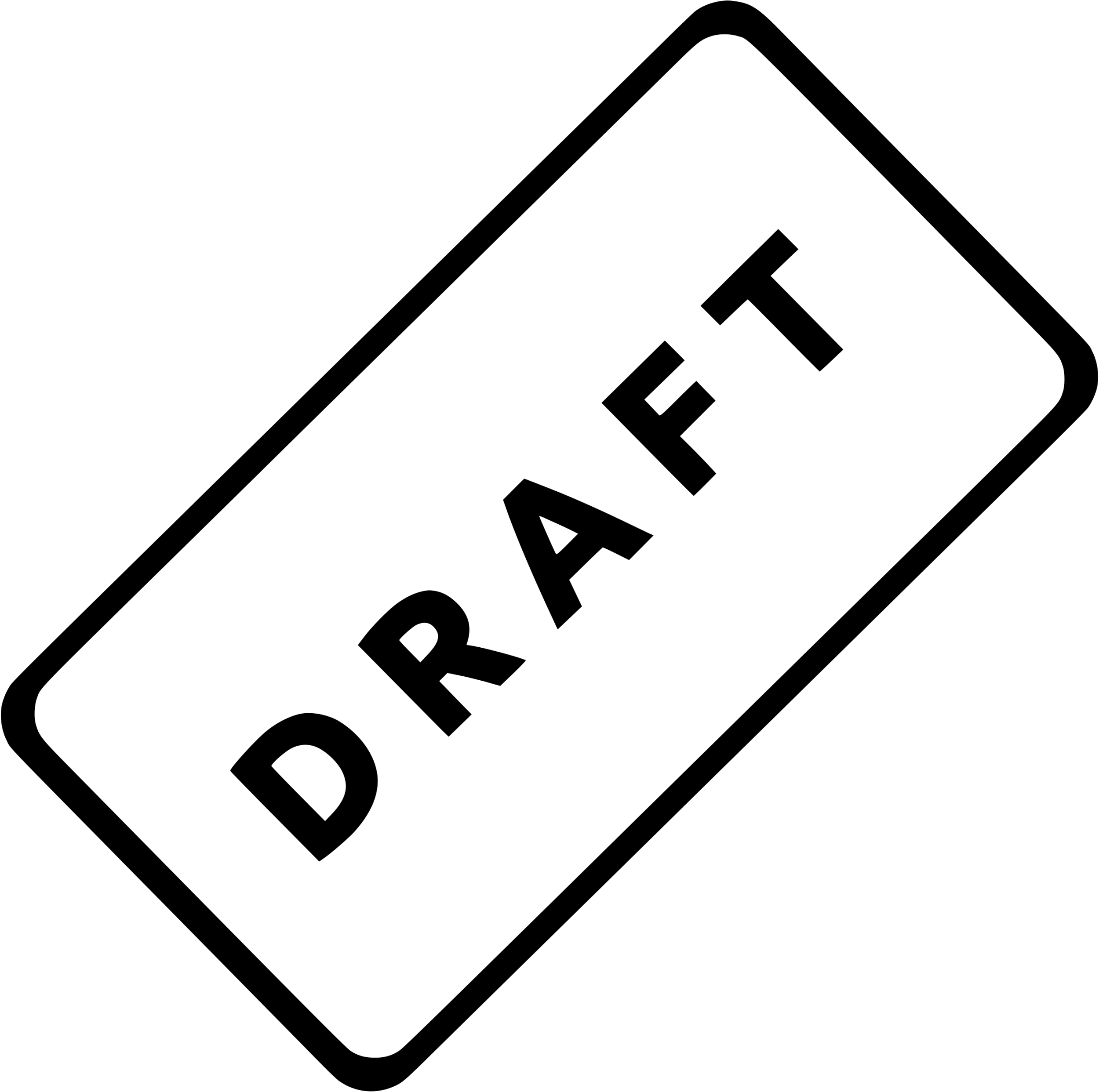MMS tablet or FullCare, 2. Supplements with multiple micronutrients 3. Iron tablet/Iron folic acid 4. Other (specify) 5. Don’t know |
| CP.15 | How many months pregnant were you when you first started taking [INSERT NAME OF PRODUCT FROM CP.15]?  INSTRUCTION: SHOW VISUAL AID  CAPI Instruction: Repeat this question for all the chosen responses in CP.15 | ___  (Record no. months)  (0-9 months  98. Don’t know |
| CP.16 | How many days did you take [INSERT NAME OF PRODUCT FROM CP.15] in the last **month**?  [If answer is not numeric probe for proximate number of days]  INSTRUCTION: SHOW VISUAL AID  CAPI Instruction: Repeat this question for all the chosen responses in CP.15 | ___ ___  (Record no. days)  (0-31 days)  98. Don’t know |
| CP.17 | Where have you gotten [INSERT NAME OF PRODUCT FROM CP.15] from during this pregnancy?  [PROBE: ANYWHERE ELSE?]  [MULTI-SELECT]  INSTRUCTION: SHOW VISUAL AID  CAPI Instruction: Repeat this question for all the chosen responses in CP.15 | 1. 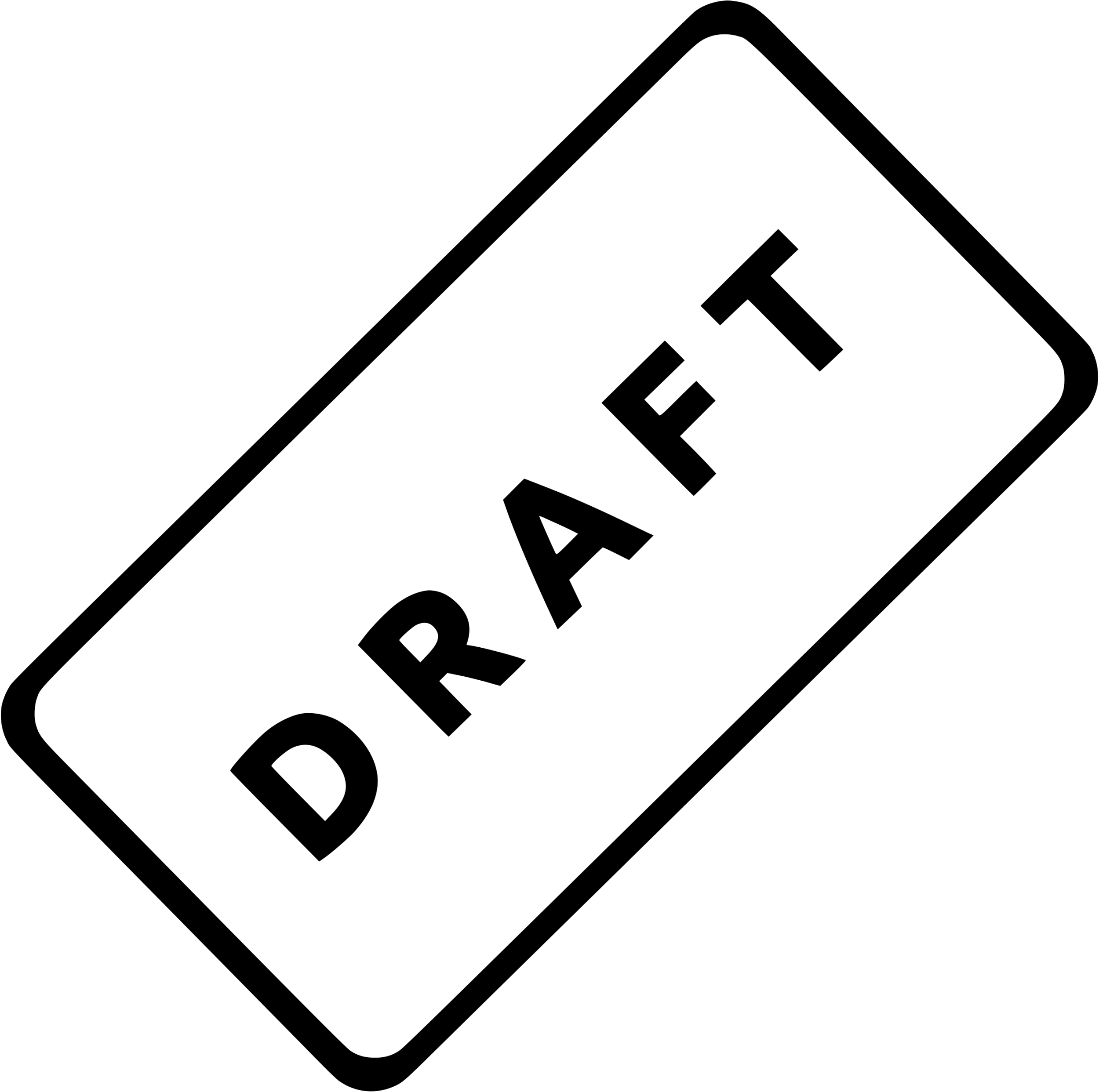Medical college hospital 2. Specialized govt hospital 3. District hospital 4. MCWC 5. Upazila health complex 6. UH & family welfare center 7. Community clinic 8. Satellite Clinic/EPIU outreach 9. NGO sector (NGO clinics, NGO worker) 10. Blue star provider 11. Private medical college 12. Private hospital 13. Private clinic 14. Qualified doctor chamber 15. Unqualified doctor chamber 16. Pharmacy 17. Retail shop 18. Community health worker 19. Family/Friend/Neighbor 20. Others (specify)   98. Don’t know |
| CP.18 | During this pregnancy has any health care provider or a health worker, or a nutrition worker talked with you about following? |  |
| CP.18.1 | Taking tablets or syrup that contain iron | 1. No 2. Yes 3. Don’t know |
| CP.18.2 | Benefits of tablets or syrup that contain iron | 1. No 2. Yes 3. Don’t know |
| CP.18.3 | Side effects of tablets or syrup that contain iron | 1. No 2. Yes 3. Don’t know |
| CP.19 | During this pregnancy, were you given, or did you buy a vitamin A capsule? | 1. No 2. Yes 3. Don’t know |
| CP.20 | During this pregnancy, were you given, or did you buy any tablets for intestinal worms? | 1. No 2. Yes 3. Don’t know |
| CP.21 | At any time before this pregnancy, were you given an injection in the arm to prevent the baby from getting tetanus after birth? | 1. No>>skip to CP.25 2. Yes 3. Don’t know>>skip to CP.25 |
| CP.22 | Before this pregnancy, how many times did you get a tetanus injection? | ___ >> skip to CP.24 if >1  (Record no of times)  (1-9 times)  98. Don’t know |
| CP.23 | How many years ago did you receive that tetanus injection? | ___ ___  (Record no. years)  (0-49 years ago)  98. Don’t know |
| CAPI Instruction: Skip CP.24 if CP.22=1 | | |
| CP.24 | How many years ago did you receive the last tetanus injection prior to this pregnancy? | ___ ___  (Record no. years)  (0-49 years ago)  98. Don’t know |
| CP.25 | As part of your antenatal care during this pregnancy did a health care provider do any of the following? |  |
| CP.25.1 | Measure your blood pressure | 1. No 2. Yes 3. Don’t know |
| CP.25.2 | Take a blood sample | 1. No>>skip to CP.26 2. Yes 3. Don’t know>>skip to CP.26 |
| CP.25.3 | Was the blood sample taken to test for your glucose/sugar level or diabetes? | 1. No 2. Yes 3. Don’t know |
| CP.25.4 | Was the blood sample taken to test for your hemoglobin level or anemia? | 1. No 2. Yes 3. Don’t know |
| CP.26 | During this pregnancy, have you ever been diagnosed as anemic (i.e. level of hemoglobin less than 11 g/dl in your blood sample)? | 1. 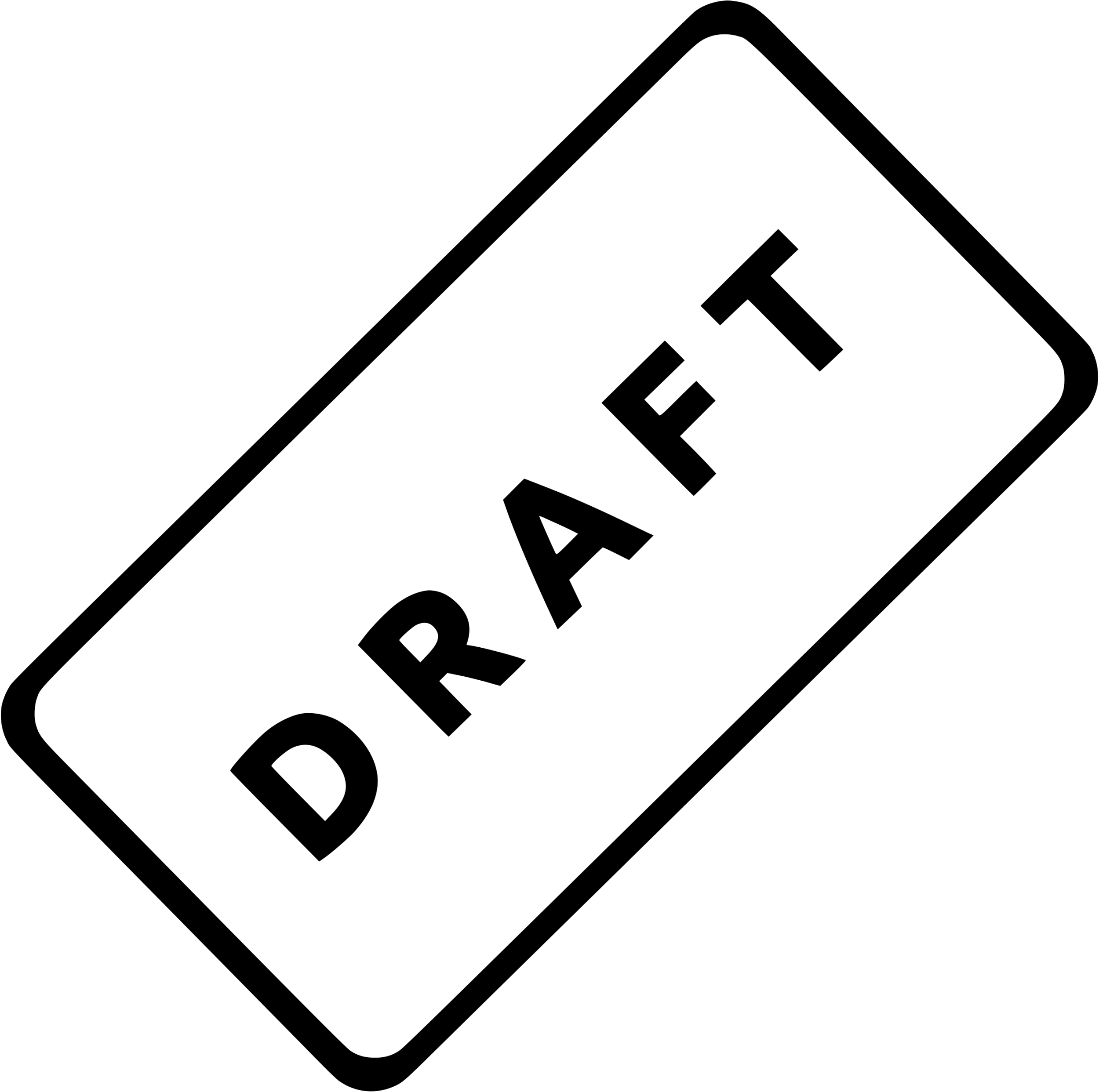No>>skip to CP.28 2. Yes 3. Don’t know>>skip to CP.28 |
| CP.27 | Did you receive any treatment for anemia? | 1. No 2. Yes 3. Don’t know |
| CP.28 | As part of your antenatal care during this pregnancy did a health care provider do the following? |  |
| CP.28.1 | Check your weight | 1. No>>skip to CP.29 2. Yes 3. Don’t know>>skip to CP.29 |
| CP.28.2 | Talk with you about your weight gain | 1. No 2. Yes 3. Don’t know |
| CP.29 | During this pregnancy, have you been told you are underweight by a health care provider? | 1. No>>skip to CP.31 2. Yes 3. Don’t know>>skip to CP.31 |
| CP.30 | When you were told you are underweight, did you receive any information on how to prepare nutritious foods like khichuri and halwa from a health care provider? | 1. No 2. Yes 3. Don’t know |
| CP.31 | During this pregnancy did a health care provider or a health worker or a nutrition worker discuss with you about following? |  |
| CP.31.1 | Eating additional amount of food and a variety of foods? | 1. No 2. Yes 3. Don’t know |
| CP.31.2 | Importance of institutional delivery | 1. No 2. Yes 3. Don’t know |
| CP.31.3 | Cord care | 1. No 2. Yes 3. Don’t know |
| CP.31.4 | Exclusive Breastfeeding | 1. No 2. Yes 3. Don’t know |
| CP.31.5 | Keeping the baby warm | 1. No 2. Yes 3. Don’t know |
| CP.32 | During this pregnancy, did you use a mosquito net regularly, sometimes, or never? | 1. Never 2. Regularly 3. Sometimes |
| CP.33 | During this pregnancy, did you take FP Fansidar to keep you from getting malaria? | 1. No 2. Yes 3. Don’t know |

Module end time XX: XX


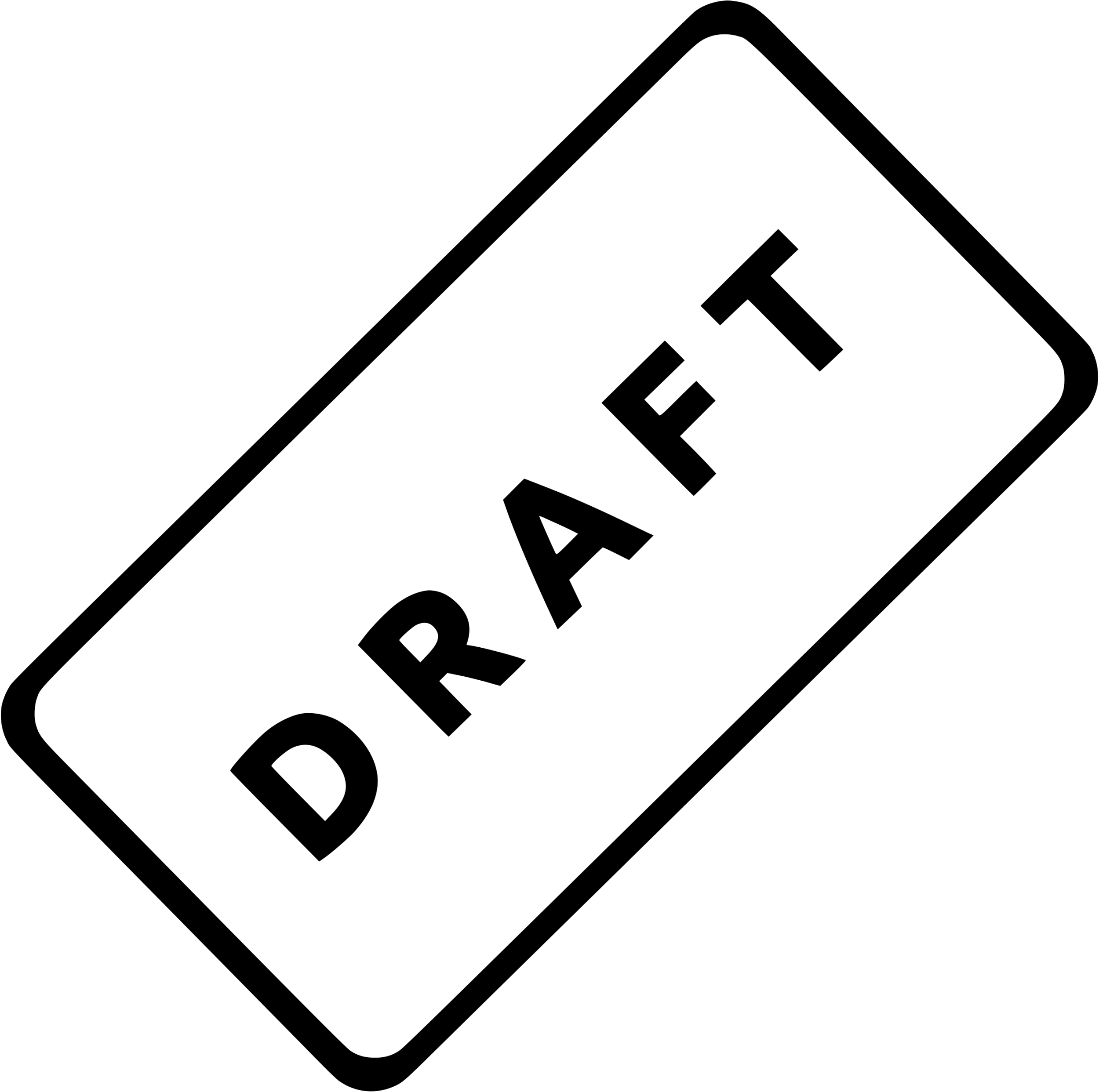
Module start time XX: XX

| **Antenatal care - previous pregnancy (PP)** | | |
| --- | --- | --- |
| **Respondent: All WRA 15-49y or married adolescent 10-14y with birth (live or still) in the last 2 years in the HH** | | |
| CAPI instructions:   - Repeat this section for all names listed in S.N.2 and S.N 3 (married adolescent) of the respondent matrix.   1. Add Respondent ID ___ - Skip if   1. BH.2=01 (Woman has never given a birth) or   2. BH.12=0 (Woman has no live birth in the last 2 years) or   3. BH.13=0 (Woman has no still birth in the last 2 years) - Administer all questions in this section for   1. All births that women have had in the last two years, i.e for the name in BH.12.1 and BH.13.1. BUT skip PP.27-PP.30 for name in BH.13.1 (stillbirth)   PP.0 Display name of the child or DOB in case of still birth | | |
| Now, I would like to ask about the various health and nutrition interventions you may have received during your pregnancy with [INSERT NAME IN PP.0] | | |
| **Q. no** | **Q. label** | **Response** |
| PP.1 | During your pregnancy with [INSERT NAME IN PP.0], did you see anyone for antenatal care? | 1. No>> skip to PP.6 2. Yes |
| PP.2 | Whom did you see?  [PROBE: ANYONE ELSE?]  [MULTI SELECT] | 1. Doctor/Nurse/Midwife 2. Paramedic 3. Family Welfare Visitor (FWV) 4. Community Skilled Birth Assistant (CSBA) 5. Sub-Assistant Community Medical Assistant (SACMO) 6. Community Health Care Provider (CHCP) 7. Health assistant 8. Family welfare assistant 9. NGO workers 10. Trained TBA 11. Untrained TBA 12. Unqualified doctor 13. Others (Specify) |
| PP.3 | Where did you receive antenatal care?  [PROBE: ANYWHERE ELSE]  [MULTI SELECT] | 1. Home 2. Medical college hospital 3. Specialized govt hospital 4. District hospital 5. MCWC 6. Upazila health complex & family welfare center 7. Union Health & family welfare center 8. Community clinic 9. Satellite Clinic/EPIU outreach 10. 10. NGO sector (NGO clinics, NGO worker) 11. Private medical college 12. Private hospital 13. Private clinic 14. Qualified doctor chamber 15. Unqualified doctor chamber 16. Pharmacy 17. Others (Specify) |
| PP.4 | How many months pregnant were you when you first received antenatal care during your pregnancy with [INSERT NAME IN PP.0]? | ___  (Record no. months)  (1-9 months)   1. Don’t know |
| PP.5 | How many times did you receive antenatal care during your pregnancy with [INSERT NAME IN PP.0]? | ___ ___  (Record no. times)  (1-20 times)   1. Don’t know |
| PP.6 | During your pregnancy with [INSERT NAME IN PP.0], were you given, or did you buy any tablets that contain calcium?  INSTRUCTION: SHOW VISUAL AID OF CALCIUM  READ ALOUD: PLEASE THINK ABOUT THESE AND SIMILAR PRODUCTS; THE PICTURES ARE JUST EXAMPLES OF TABLETS CONTAINING CALCIUM | 1. 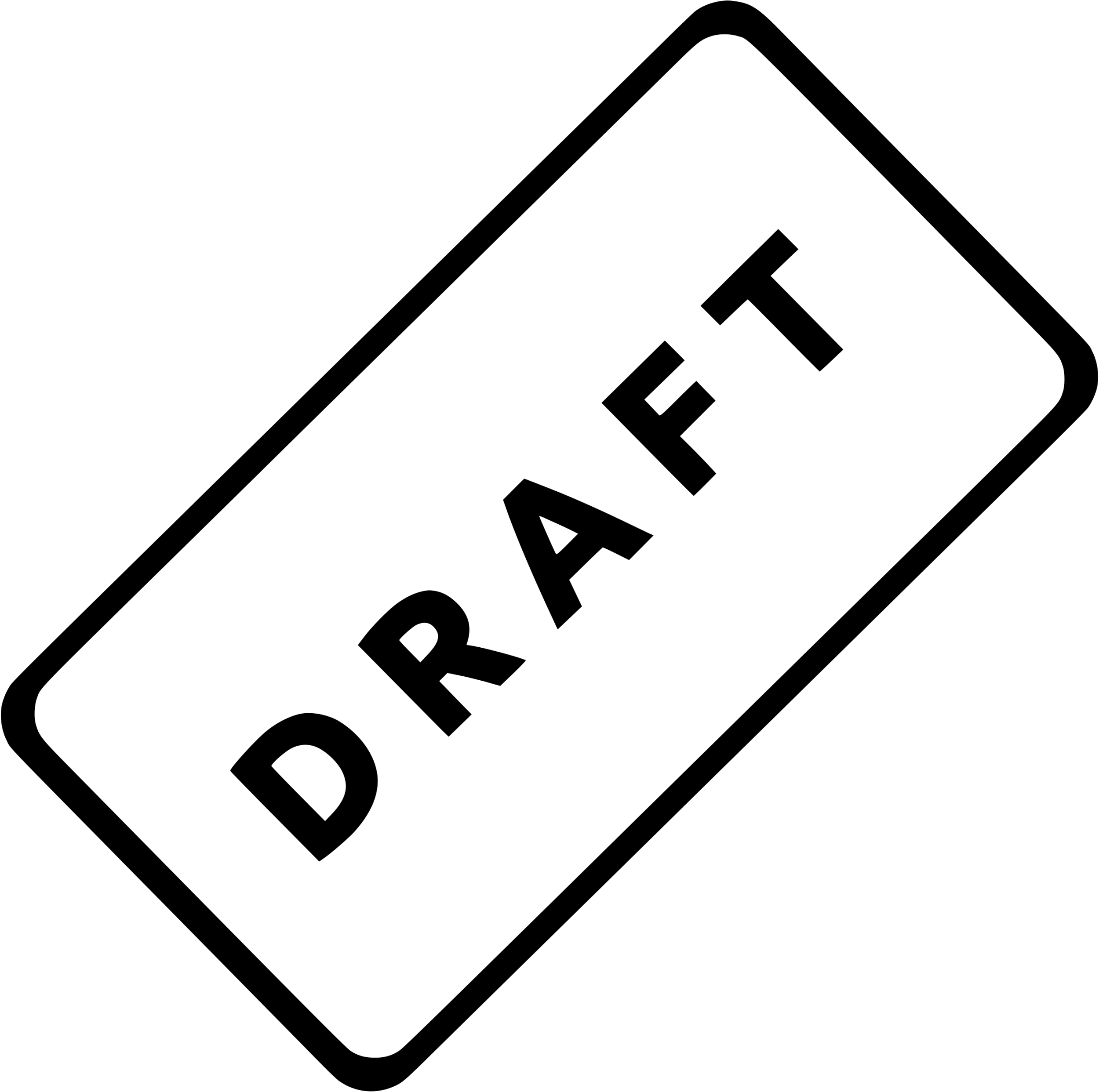No>>skip to PP.13 2. Yes 3. Don’t know>>skip to PP.13 |
| PP.7 | How many months pregnant were you when you first started taking tablets that contain calcium during your pregnancy with [INSERT NAME IN PP.0]?  INSTRUCTION: SHOW VISUAL AID OF CALCIUM | ___  (Record no. months)  (0-9 months  98. Don’t know |
| PP.8 | During your pregnancy with [INSERT NAME IN PP.0], how many **months** did you take tablets that contain calcium?  INSTRUCTION: SHOW VISUAL AID OF CALCIUM | ___  (Record no. months)  (0-9 months  98. Don’t know |
| PP.9 | During your whole pregnancy with [INSERT NAME IN PP.0], for how many **days** did you take tablets that contain calcium?  [If answer is not numeric probe for proximate number of days]  INSTRUCTION: SHOW VISUAL AID OF CALCIUM | ___ ___ ___  (Record no. days)  (0- 270 days)  998. Don’t know |
| PP.10 | During your pregnancy with [INSERT NAME IN PP.0], how many days a **MONTH** did you usually take tablets that contain calcium?  INSTRUCTION: SHOW VISUAL AID OF CALCIUM  INSTRUCTION: IF THE WOMAN DOES NOT REMEMBER, PROBE FOR THE APPROXIMATE NUMBER OF DAYS, E.G., BY ASKING HOW MANY MONTHS PREGNANT SHE WAS WHEN SHE BEGAN TAKING THE TABLETS AND WHETHER SHE TOOK THE TABLETS EVERY DAY AFTER THAT. | ___ ___  (Record no. days)  (0-31 days)  98. Don’t know |
| PP.11 | Where did you get tablets that contain calcium from during your pregnancy with [INSERT NAME IN PP.0]?  [PROBE: ANYWHERE ELSE?]  [MULTI SELECT]  INSTRUCTION: SHOW VISUAL AID OF CALCIUM | 1. Medical college hospital 2. Specialized govt hospital 3. District hospital 4. MCWC 5. Upazila health complex 6. UH & family welfare center 7. Community clinic 8. Satellite Clinic/EPIU outreach 9. NGO sector (NGO clinics, NGO worker) 10. Blue star provider 11. Private medical college 12. Private hospital 13. Private clinic 14. Qualified doctor chamber 15. Unqualified doctor chamber 16. Pharmacy 17. Retail shop 18. Community health worker 19. Family/Friend/Neighbor 20. Others (specify)   98. Don’t know |
| PP.12 | During your whole pregnancy with [INSERT NAME IN PP.0] did anyone like a health care provider or health worker, or a nutrition worker talk with you about the following? | 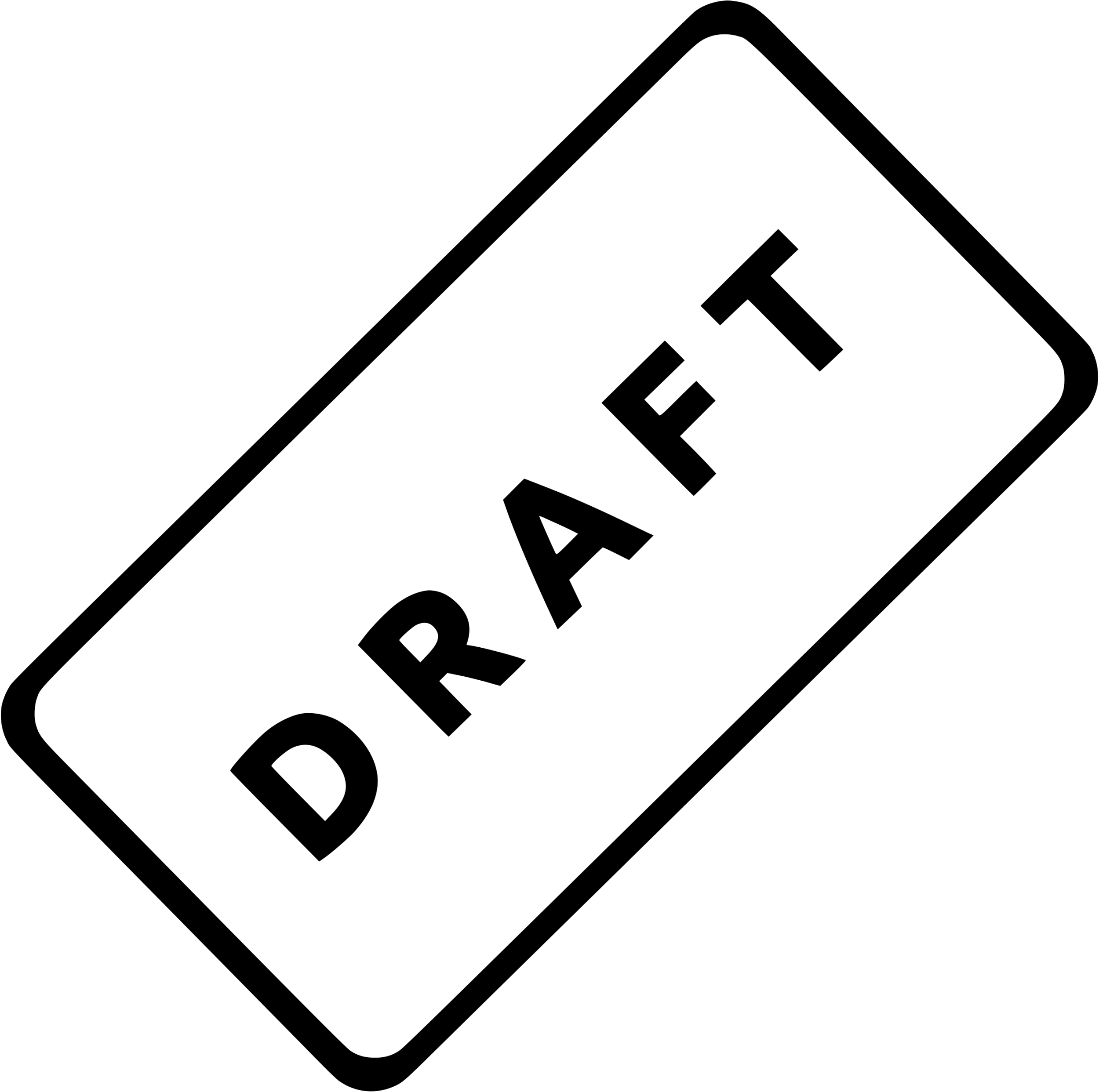 |
| PP.12.1 | Taking tablets that contain calcium | 1. No 2. Yes 3. Don’t know |
| PP.12.2 | Benefits of tablets that contain calcium | 1. No 2. Yes 3. Don’t know |
| PP.12.3 | Side effects of tablets that contain calcium | 1. No 2. Yes 3. Don’t know |
| PP.13 | During your pregnancy with [INSERT NAME IN PP.0], were you given, or did you buy any tablet or syrup that contains iron?  INSTRUCTION: SHOW VISUAL AID OF MMS TABLET & FULLCARE, COMMON TYPES OF MULTIPLE MICRONUTRIENT SUPPLEMENTS, & COMMON TYPES OF IRON/IFA | 1. No>> skip to PP.20 2. Yes 3. Don’t know>> skip to PP.20 |
| PP.14 | During your pregnancy with [INSERT NAME IN PP.0], were you given or did you buy any of the following? :   1. MMS TABLET OR FULLCARE?   INSTRUCTION: SHOW VISUAL AID OF MMS TABLET & FULLCARE   1. SUPPLEMENTS WITH MULTIPLE MICRONUTRIENTS?   INSTRUCTION: SHOW VISUAL AID OF COMMON TYPES OF MULTIPLE MICRONUTRIENT SUPPLEMENTS.  READ ALOUD: PLEASE THINK ABOUT THESE AND SIMILAR PRODUCTS; THE PICTURES ARE JUST EXAMPLES OF SUPPLEMENTS CONTAINING MMS   1. IRON TABLET OR SYRUPS?   INSTRUCTION: SHOW VISUAL AID OF COMMON TYPES OF IRON/IFA  READ ALOUD: PLEASE THINK ABOUT THESE AND SIMILAR PRODUCTS; THE PICTURES ARE JUST EXAMPLES OF SUPPLEMENTS CONTAINING IRON/IFA  [PROBE: ANYTHING ELSE?]  [MULTI SELECT] | 1. MMS tablet or FullCare, 2. Supplements with multiple micronutrients 3. Iron tablet/Iron folic acid 4. Other (specify) 5. Don’t know |
| PP.15 | How many months pregnant were you when you first started taking [NAME OF PRODUCT FROM PP.13] when you were pregnant with [INSERT NAME IN PP.0]?  INSTRUCTION: SHOW VISUAL AID  CAPI Instruction: Repeat this question for all the chosen responses in PP.14 | 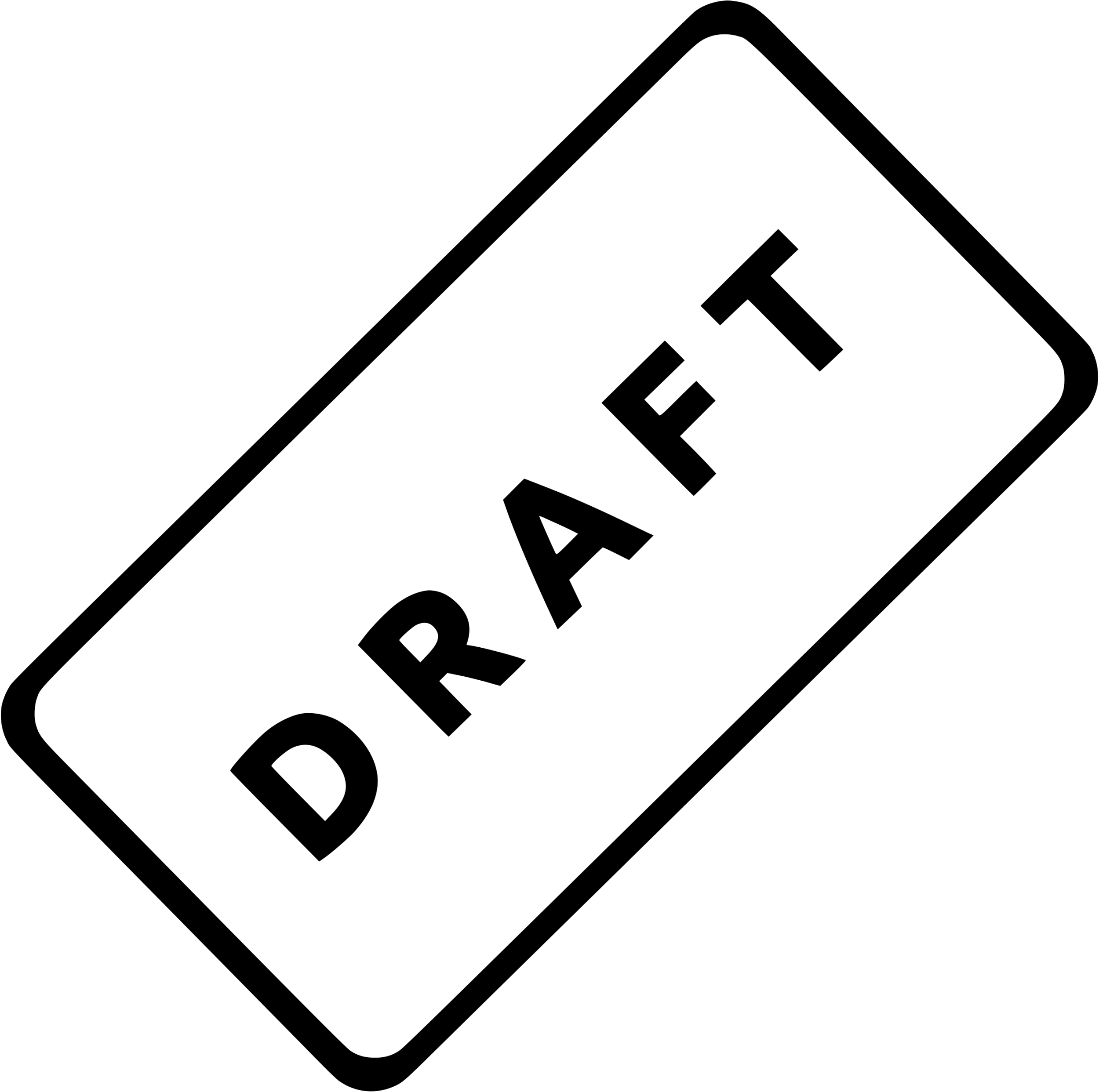  ___  (Record no. months)  (0-9 months)  98. Don’t know |
| PP.16 | During your pregnancy with [INSERT NAME IN PP.0], how many **months** did you take [NAME OF PRODUCT FROM PP.13]?  INSTRUCTION: SHOW VISUAL AID  CAPI Instruction: Repeat this question for all the chosen responses in PP.14 | ___  (Record no. months)  (0-9 months)  98. Don’t know |
| PP.17 | During the whole pregnancy with [INSERT NAME IN PP.0], for how many **days** did you take [NAME OF PRODUCT FROM PP.13]?  INSTRUCTION: SHOW VISUAL AID  CAPI Instruction: Repeat this question for all the chosen responses in PP.14  INSTRUCTION: IF ANSWER IS NOT NUMERIC, PROBE FOR APPROXIMATE NUMBER OF DAYS. | ___ ___ ___  (Record no. days)  (0-270 days)  988. Don’t know |
| PP.18 | During your pregnancy with [INSERT NAME IN PP.0], how many days a **MONTH** did you usually take [NAME OF PRODUCT FROM PP.13]?  INSTRUCTION: SHOW VISUAL AID  CAPI Instruction: Repeat this question for all the chosen responses in PP.14  INSTRUCTION: IF THE WOMAN DOES NOT REMEMBER, PROBE FOR THE APPROXIMATE NUMBER OF DAYS, E.G., BY ASKING HOW MANY MONTHS PREGNANT SHE WAS WHEN SHE BEGAN TAKING THE TABLETS AND WHETHER SHE TOOK THE TABLETS EVERY DAY AFTER THAT. | ___ ___  (Record no. days)  (0-31 days)  98. Don’t know |
| PP.19 | Where did you get [INSERT NAME OF PRODUCT FROM PP.13] from during your pregnancy with [INSERT NAME IN PP.0]?  [PROBE: ANYWHERE ELSE?]  [MULTI SELECT]  INSTRUCTION: SHOW VISUAL AID  CAPI Instruction: Repeat this question for all the chosen responses in PP.14 | 1. Medical college hospital 2. Specialized govt hospital 3. District hospital 4. MCWC 5. Upazila health complex 6. UH & family welfare center 7. 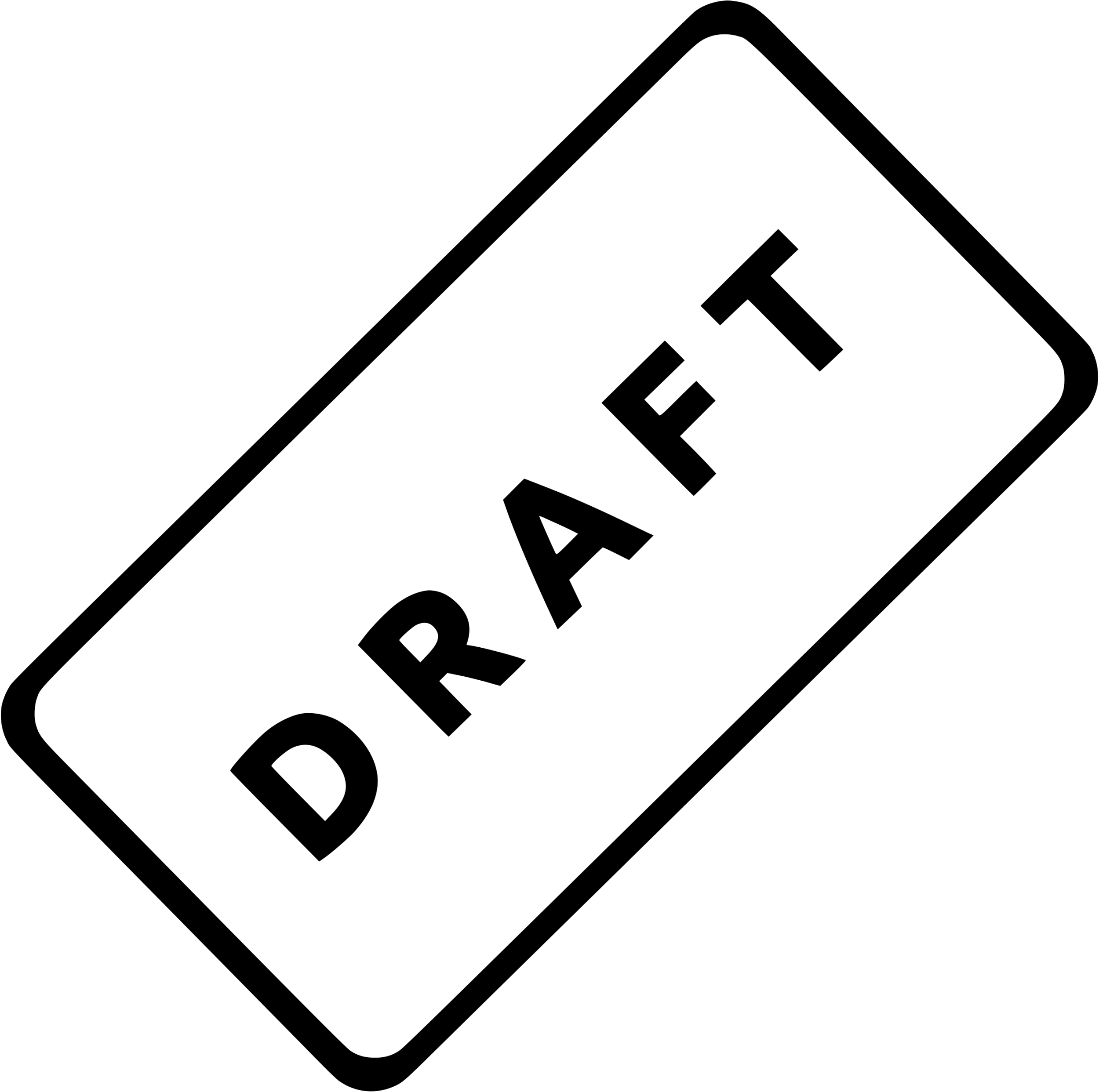Community clinic 8. Satellite Clinic/EPIU outreach 9. NGO sector (NGO clinics, NGO worker) 10. Blue star provider 11. Private medical college 12. Private hospital 13. Private clinic 14. Qualified doctor chamber 15. Unqualified doctor chamber 16. Pharmacy 17. Retail shop 18. Community health worker 19. Family/Friend/Neighbor 20. Others (specify)   98. Don’t know |
| PP.20 | During your pregnancy with [INSERT NAME IN PP.0] did any health care provider or a health worker, or a nutrition worker talk with you about the followings? |  |
| PP.20.1 | Taking tablets or syrup that contain iron | 1. No 2. Yes 3. Don’t know |
| PP.20.2 | Benefits of tablets or syrup that contain iron | 1. No 2. Yes 3. Don’t know |
| PP.20.3 | Side effects of tablets or syrup that contain iron | 1. No 2. Yes 3. Don’t know |
| PP.21 | During your pregnancy with [INSERT NAME IN PP.0] were you given, or did you buy vitamin A capsule? | 1. No 2. Yes 3. Don’t know |
| PP.22 | During your pregnancy with [INSERT NAME IN PP.0], were you given, or did you buy any tablets for intestinal worms? | 1. No 2. Yes 3. Don’t know |
| PP.23 | During your pregnancy with [INSERT NAME IN PP.0], were you given an injection in the arm to prevent the baby from getting tetanus after birth? | 1. No>>skip to PP.25 2. Yes 3. Don’t know>>skip to PP.25 |
| PP.24 | During your pregnancy with [INSERT NAME IN PP.0], how many times did you get a tetanus injection? | ___  (Record no. times)  (1-9 times)  98. Don’t know |
| PP.25 | Before your pregnancy with [INSERT NAME IN PP.0], did you receive any tetanus injections? | 1. No>>skip to PP.29 2. Yes 3. Don’t know>>skip to PP.29 |
| PP.26 | Before your pregnancy with [INSERT NAME IN PP.0], how many times did you receive a tetanus injection? | ___ skip to PP.28 if >1  (Record no. times)  (1-9 times)  98. Don’t know |
| PP.27 | How many years ago did you receive that tetanus injection? | ___ ___  (Record no. years)  (0-49 years ago)  98. Don’t know |
| CAPI Instruction: Skip PP.28 if PP.26=1 | | |
| PP.28 | How many years ago did you receive the last tetanus injection prior to this pregnancy? | 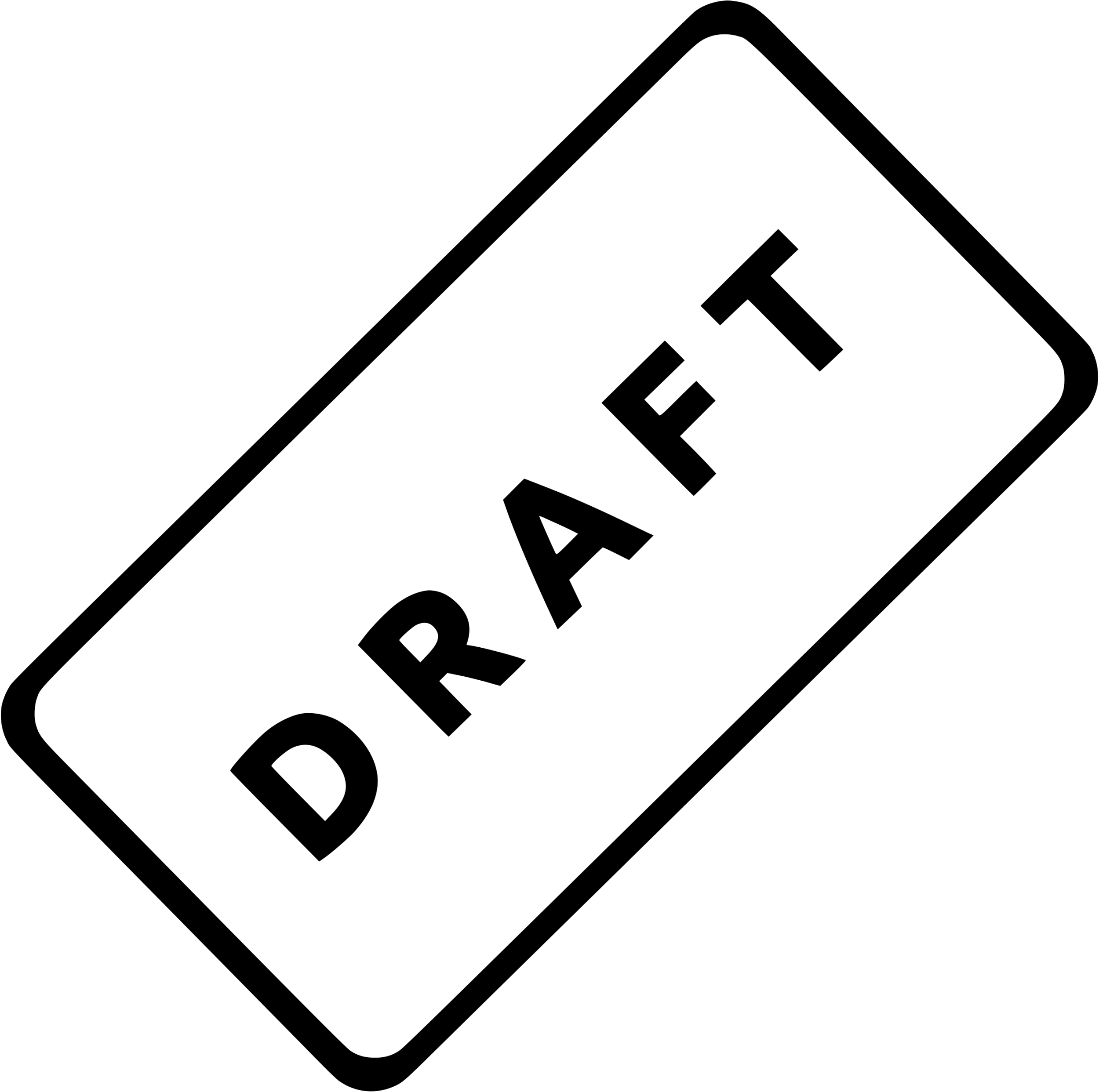___ ___  (Record no. years)  (0-49 years ago)  98. Don’t know |
| PP.29 | As part of antenatal care during your pregnancy with [INSERT NAME IN PP.0], did a health care provider do any of the following? |  |
| PP.29.1 | Measure your blood pressure | 1. No 2. Yes 3. Don’t know |
| PP.29.2 | Take a blood sample | 1. No>>skip to PP.30 2. Yes 3. Don’t know>>skip to PP.30 |
| PP.29.3 | Was the sample taken to test for your glucose/sugar level or diabetes | 1. No 2. Yes 3. Don’t know |
| PP.29.4 | Was the blood sample taken to test for your hemoglobin level or anemia | 1. No 2. Yes   Don’t know |
| PP.30 | During your pregnancy with [INSERT NAME IN PP.0], were you ever diagnosed with anemia (i.e level of hemoglobin is less than 11 g/dl in your blood sample)? | 1. No>>skip to PP.33 2. Yes   98. Don’t know>>skip to PP.33 |
| PP.31 | Did you receive any treatment for anemia? | 1. No 2. Yes   98. Don’t know |
| PP.32 | As part of antenatal care during your pregnancy with [INSERT NAME IN PP.0], did a health care provider do the following? |  |
| PP.32.1 | Check your weight | 1. No>>skip to PP.33 2. Yes 3. Don’t know>>skip to PP.33 |
| PP.32.2 | Talk with you about your weight gain | 1. No 2. Yes 3. Don’t know |
| PP.33 | During your pregnancy with [INSERT NAME IN PP.0],have you been told you are underweight? | 1. No>>skip to PP.35 2. Yes 3. Don’t know>>skip to PP.35 |
| PP.34 | When you were told you are underweight, did you receive any information on how to prepare nutritious foods like khichuri and halwa from a health care provider? | 01. No  02. Yes   1. Don’t know |
| PP.35 | During your pregnancy with [INSERT NAME IN PP.0], did a health care provider or a health worker, or a nutrition worker discuss with you about following? |  |
| PP.35.1 | Eating an additional amount of food and a variety of foods | 1. No 2. Yes 3. Don’t know |
| PP.35.2 | Importance of institutional delivery | 1. No 2. Yes 3. Don’t know |
| PP.35.3 | Cord care | 1. No 2. Yes 3. Don’t know |
| PP.35.4 | Exclusive Breastfeeding | 1. No 2. Yes 3. Don’t know |
| PP.35.5 | Keeping the baby warm | 1. No 2. Yes 3. Don’t know |
| PP.36 | During your pregnancy with [INSERT NAME IN PP.0], did you use a mosquito net regularly, sometimes, or never? | 1. Never 2. Regularly 3. Sometimes |
| PP.37 | During your pregnancy with [INSERT NAME IN PP.0], did you take FP Fansidar to keep you from getting malaria? | 1. 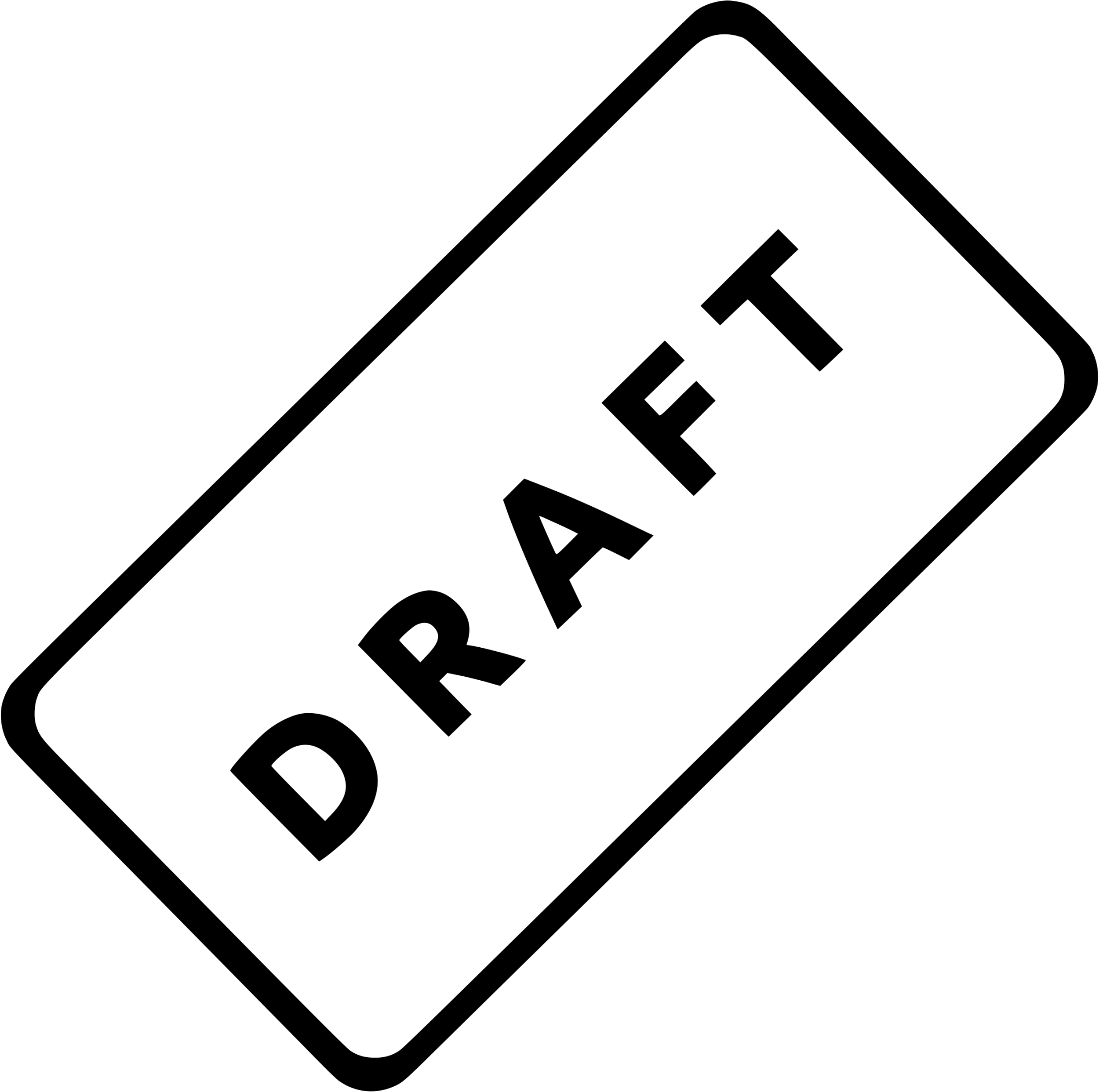No 2. Yes 3. Don’t know |

Module end time XX: XX

Module start time XX: XX

| **Delivery care and postnatal care (DC)** | | |
| --- | --- | --- |
| **Respondent: All WRA 15-49y and married adolescent 10-14y with birth (live or still) in the last 2 years** | | |
| CAPI instructions-   - Repeat this section for all names listed in S.N.2 and S.N 3 (married adolescent) of the respondent matrix.  1. Add Respondent ID ___  - Skip if  1. BH.2=01 (Woman has not given birth) or 2. BH.12=0 (Woman has no live birth in the last 2 years) or 3. BH.13=0 (Woman has no still birth in the last 2 years) or  - Ask DC.1-DC.33 for all most recent birth that women have had in the last two years, i.e for the name in BH.12.1 - Ask DC.1, DC.2, DC.3 and DC.13-15, DC19-21, DC.26-28, DC.33 for most recent stillbirth i.e for name in BH.13.1 and BH.8=02 - Ask DC.1-DC.3, DC.7-DC.9 for all prior live births. i.e. for all names in BH.14.1 and BH.09=02 - Ask DC.1-DC.3 for all prior stillbirths i.e for names in BH.15.1 and BH.8=02   DC.0=Display the name of the child (or DOB in case of still birth) | | |
| Now, I would like to ask you about the health and nutrition interventions you may have received during the birth of [INSERT FIRST NAME IN DC.0]. | | |
| **Q. no** | **Q. label** | **Response** |
| DC.1 | Where did you give birth to [INSERT NAME IN DC.0]?  [SINGLE SELECT] | 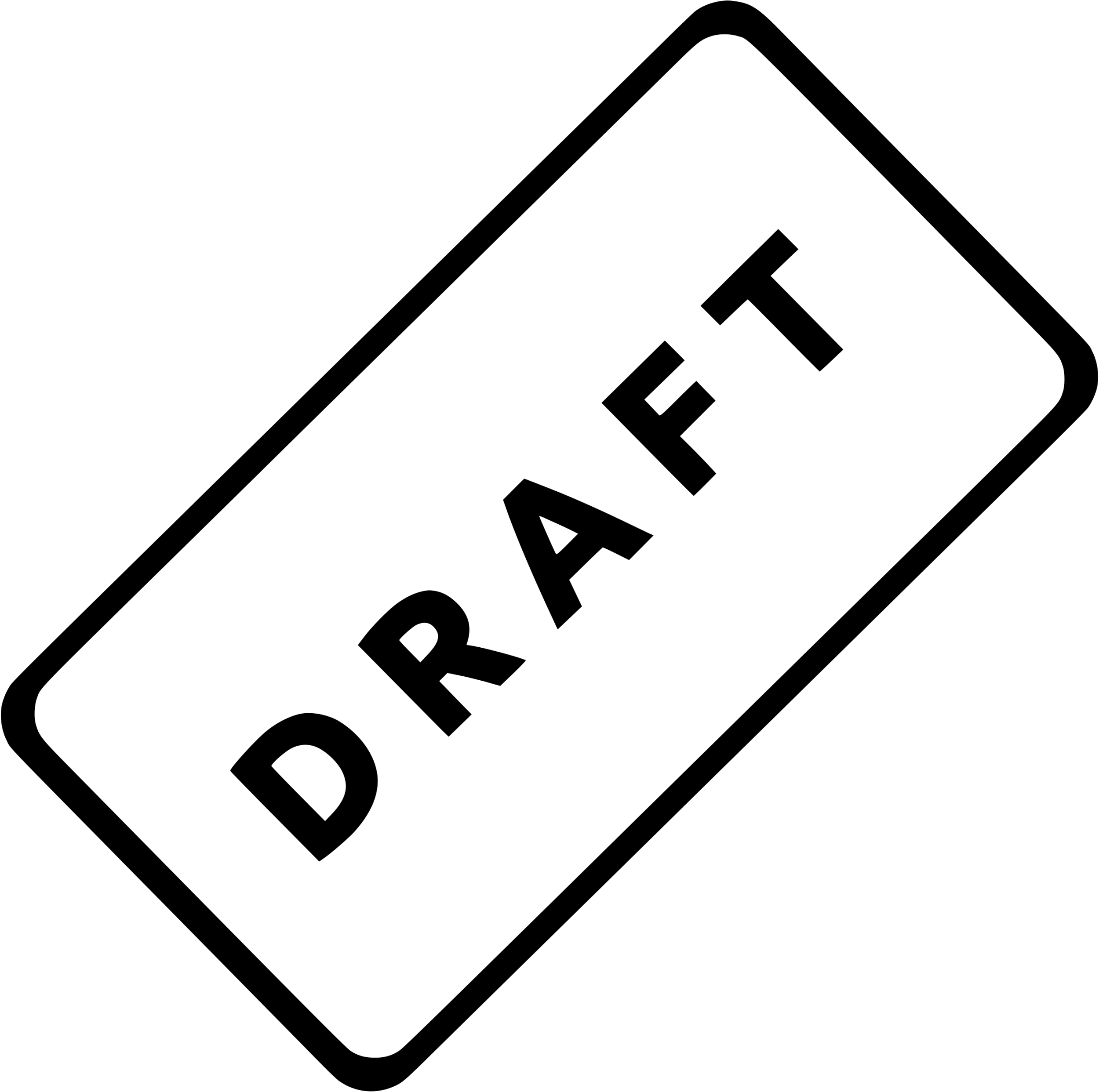**HOME**   1. Your home 2. Parent's home 3. Other home   **PUB. HEALTH SECTOR**   1. Govt./Municipal hospital 2. Govt. dispensary 3. UHC/UHP/UFWC 4. CHC/Rural Hospital/Block PHC 5. PHC/Add. PHC 6. Subcenter 7. Other public sector health facility 8. NGO/Trust hospital/ Clinic 9. PVT. HEALTH SECTOR 10. Pvt. hospital/maternity home/clinic 11. Other Pvt. sector health facility 12. Other |
| DC.2 | Who assisted with the delivery of [INSERT NAME IN DC.0]?  Anyone else?  [MULTI SELECT] | **Health Personnel**   1. Doctor 2. ANM/nurse/midwife/LHV 3. Other Health personnel   **Other person**   1. Dhai 2. Friend/relative 3. No one 4. Other |
| DC.3 | Was [INSERT NAME IN DC.0] delivered by caesarean, that is, did they cut your belly open to take the baby out? | 1. No 2. Yes 3. Don’t know |
| DC.4 | After the birth, was [INSERT NAME IN DC.0] put on your chest? | 1. No 2. Yes 3. Don’t know |
| DC.5 | 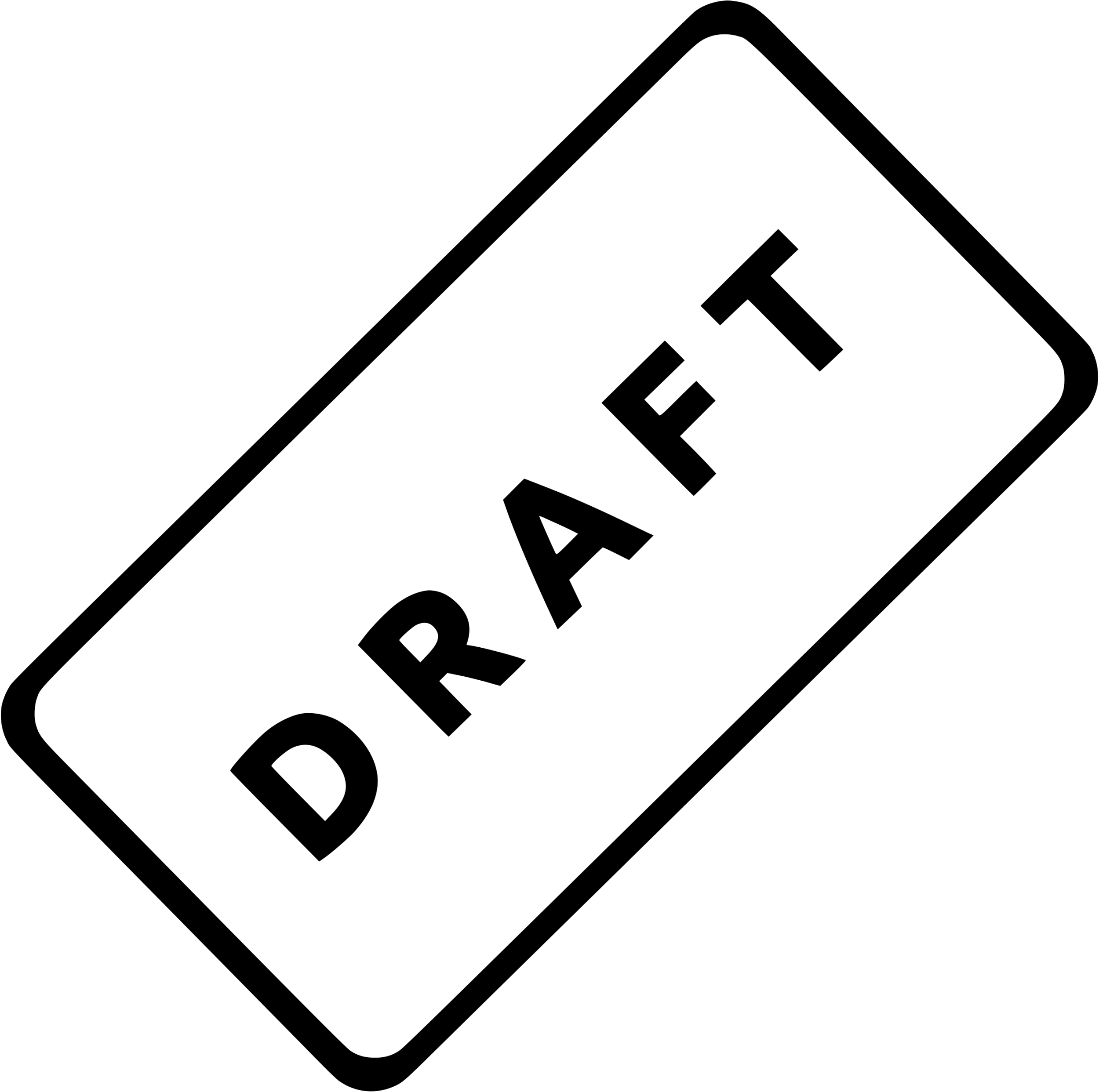Was [INSERT NAME IN DC.0]'s bare skin touching your bare skin? | 1. No 2. Yes 3. Don’t know |
| DC.6 | How long after birth was [INSERT NAME IN DC.0] put on the bare skin? | 1. Immediately 2. Hours ___ ___ (0-24) 3. Don’t know |
| DC.7 | Was [INSERT NAME IN DC.0] weighed at birth? | 1. No 2. Yes 3. Don’t know |
| DC.8 | How much did [INSERT NAME IN DC.0] weigh?  INSTRUCTION: REQUEST TO SHOW A VACCINATION CARD | ___ ___ ___ ___  (Record grams)  (500-9990 grams)  9998. Don’t know |
| DC.9 | Was the birthweight recorded in the previous question from a vaccination card or any other document, or was it reported by the respondent?  INSTRUCTION: DO NOT ASK THIS QUESTION TO THE RESPONDENT | 1. From a vaccination card/other document 2. Reported (word of mouth) |
| Ask DC.10 and DC.11 if weight (DC.8) <2500 grams | | |
| DC.10 | Did anyone (such as doctor, nurse, paramedics, health worker, nutrition worker) explain to you about caring for a low birth weight baby? | 1. No>>skip to DC.12 2. Yes 3. Don’t know>>skip to DC.12 |
| DC.11 | Did they discuss any of the following? |  |
| DC.11.1 | Early skin to skin contact | 1. No 2. Yes 3. Don’t know |
| DC.11.2 | Continuous skin to skin contact | 1. No 2. Yes 3. Don’t know |
| DC.11.3 | Prolonged skin to skin contact | 1. No 2. Yes 3. Don’t know |
| DC.11.4 | Frequent and exclusive breastfeeding | 1. No 2. Yes 3. Don’t know |
| DC.11.5 | Other safe ways to feed your child | 1. No 2. Yes 3. Don’t know |
| DC.12 | How long after [INSERT NAME IN DC.0] was delivered did you stay in the health facility? | 1. Hours ___ ___ (0-24 hours) 2. Days ___ ___ (0-50 days) 3. Weeks ___ ___ (0-10 weeks) 4. Don’t know |
| Ask DC.13 to DC.25 if delivery took place in a health facility. i.e if response to DC.1 = 4 to 14 | | |
| I would like to know about the checks on your health after delivery whether in a health facility. For example, someone asking you questions about your health or examining you. | | |
| DC.13 | Before you left the facility, did anyone check on your  health? | 1. No>>Skip to DC.16 2. Yes 3. Don’t know>>Skip to DC.16 |
| DC.14 | How long after delivery did the first check take place?  INSTRUCTION: IF LESS THAN ONE DAY, RECORD HOURS; IF LESS THAN ONE WEEK, RECORD DAYS. | 1. Hours ___ ___ (0-24 hours) 2. Days ___ ___ (0-50 days) 3. Weeks ___ ___ (0-10 weeks) 4. Don’t know |
| DC.15 | Who checked on your health at that time?  INSTRUCTION: PROBE FOR MOST QUALIFIED PERSON. | 1. Doctor 2. ANM/nurse/midwife/LHV 3. Other Health personnel 4. Dhai 5. Friend/relative 6. No one 7. Other (Specify)   98. Don’t know |
| I would like to talk to you about checks on [INSERT NAME IN DC.0] health. For example, someone examining [INSERT NAME IN DC.0], checking the cord, or talking to you about how to care for [INSERT NAME IN DC.0] | | |
| DC.16 | Before [INSERT NAME IN DC.0] left the facility, did anyone check on [INSERT NAME IN DC.0]'s health? | 1. No>>Skip to DC.19 2. Yes 3. Don’t know>>Skip to DC.19 |
| DC.17 | How long after delivery was [INSERT NAME IN DC.0]’s health first checked? | 1. Hours ___ ___ (0-24 hours) 2. Days ___ ___ (0-50 days) 3. Weeks ___ ___ (0-10 weeks) 4. Don’t know |
| DC.18 | Who checked on [INSERT NAME IN DC.0] health at that time? | 1. Doctor 2. ANM/nurse/midwife/LHV 3. Other Health personnel 4. Dhai 5. Friend/relative 6. No one 7. Other (Specify)   98. Don’t know |
| Now I would like to talk to you about what happened after you left the facility. | | |
| DC.19 | Did anyone check on your health after you left the facility? | 1. No>>Skip to DC.22 2. Yes   98. Don’t know>>Skip to DC.22 |
| DC.20 | How long after delivery did that check take place?  INSTRUCTION: IF LESS THAN ONE DAY, RECORD HOURS; IF LESS THAN ONE WEEK, RECORD DAYS. | 1. Hours ___ ___ (0-24 hours) 2. Days ___ ___ (0-50 days) 3. Weeks ___ ___ (0-10 weeks) 4. Don’t know |
| DC.21 | Who checked on your health at that time?  INSTRUCTION: PROBE FOR MOST QUALIFIED PERSON. | 1. 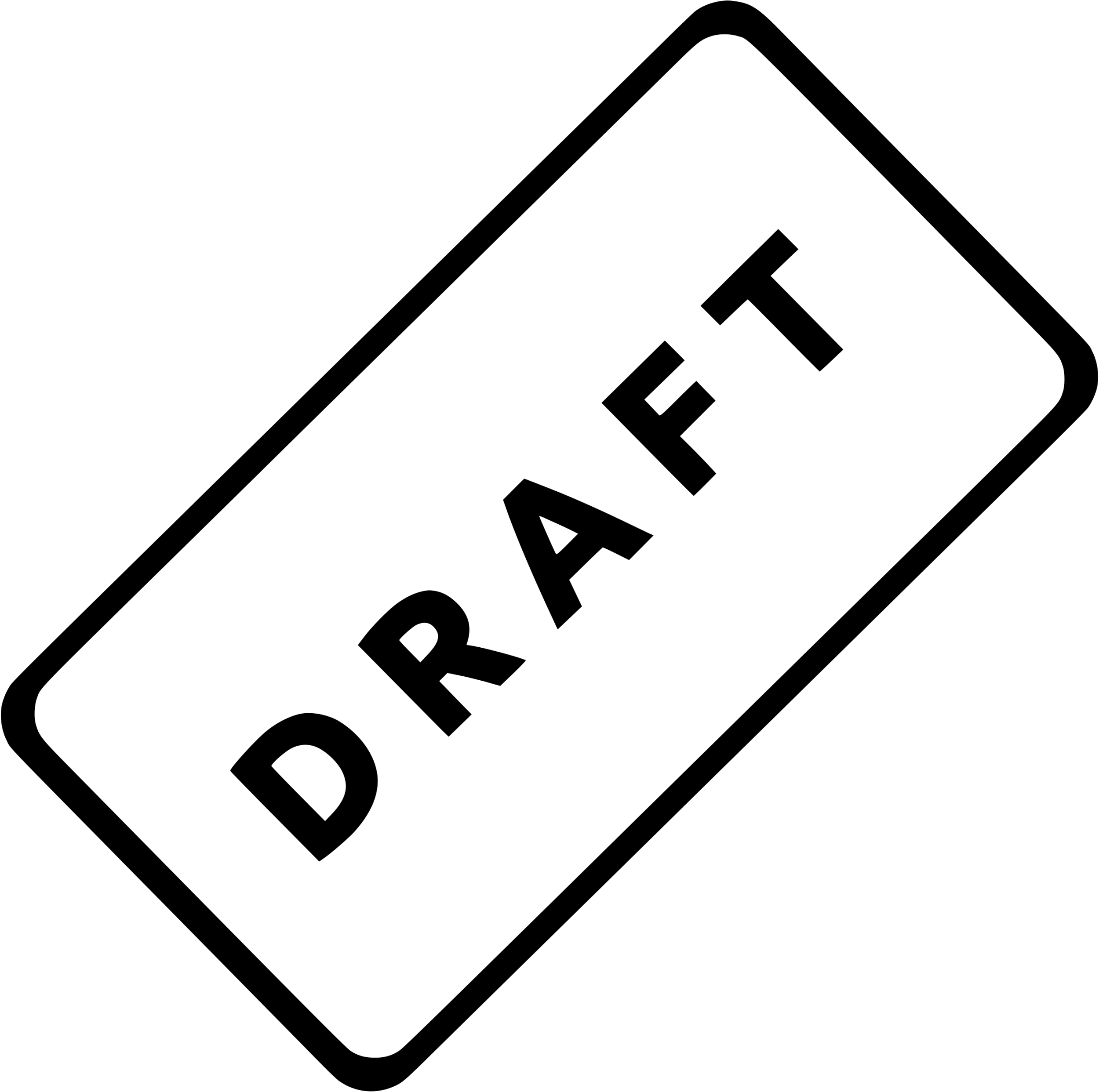 Doctor 2. ANM/nurse/midwife/LHV 3. Other Health personnel 4. Dhai 5. Friend/relative 6. No one 7. Other (Specify) |
| DC.22 | After [INSERT NAME IN DC.0] left the facility did any health care provider (such as doctor, nurse, paramedics, health worker) check on [INSERT NAME IN DC.0]’s health? | 1. No>>Skip to DC.25 2. Yes   98. Don’t know>>Skip to DC.25 |
| DC.23 | How long after the birth of [INSERT NAME IN DC.0] did that check take place?  INSTRUCTION: IF LESS THAN ONE DAY, RECORD HOURS; IF LESS THAN ONE WEEK, RECORD DAYS. | 1. Hours ___ ___ (0-24 hours) 2. Days ___ ___ (0-50 days) 3. Weeks ___ ___ (0-10 weeks) 4. Don’t know |
| DC.24 | Who checked on [INSERT NAME IN DC.0] health at that time?  INSTRUCTION: PROBE FOR THE MOST QUALIFIED PERSON | 1. Doctor 2. ANM/nurse/midwife/LHV 3. Other Health personnel 4. Dhai 5. Friend/relative 6. No one 7. Other (Specify)   98. Don’t know |
| DC.25 | After [INSERT NAME IN DC.0] was born, were you given any leaflets or supplies that promote breastmilk substitute or any formula milk while you were in the hospital? | 1. No 2. Yes, leaflet from formula company promoting formula feeding or other supplies 3. Yes, A gift or samples to take home including formula, bottles or other supplies 4. Other (specify)   98. Don’t know |
| Ask DC.26 to DC.30 if the delivery took place at home. i.e if response to DC.1 = 1 to 3 | | |
| I would like to talk to you about checks on your health after delivery, for example, someone asking you questions about your health or examining you. | | |
| DC.26 | Did anyone check on your health after you gave birth to [INSERT NAME IN DC.0]? | 1. No>>Skip to DC.29 2. Yes   98. Don’t know>>Skip to DC.29 |
| DC.27 | How long after delivery did the first check take place?  INSTRUCTION: IF LESS THAN ONE DAY, RECORD HOURS; IF LESS THAN ONE WEEK, RECORD DAYS. | 1. 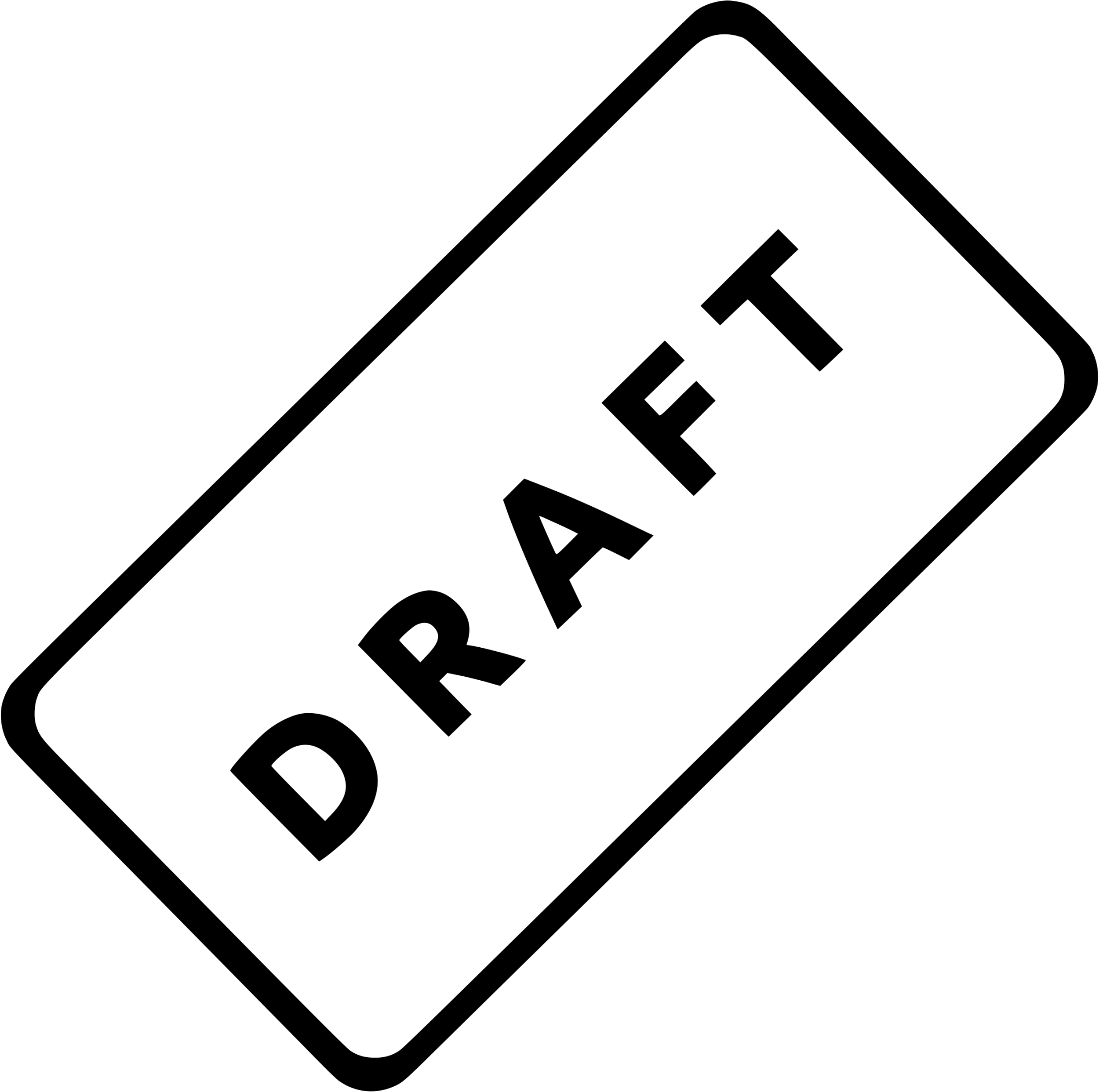Hours ___ ___ (0-24 hours) 2. Days ___ ___ (0-50 days) 3. Weeks ___ ___ (0-10 weeks) 4. Don’t know |
| DC.28 | Who checked on your health at that time?  INSTRUCTION: PROBE FOR MOST QUALIFIED PERSON. | 1. Doctor 2. ANM/nurse/midwife/LHV 3. Other Health personnel 4. Dhai 5. Friend/relative 6. No one 7. Other (Specify)   98. Don’t know |
| I would like to talk to you about checks on [INSERT NAME IN DC.0] health. For example, someone examining [INSERT NAME IN DC.0], checking the cord, or talking to you about how to care for [INSERT NAME IN DC.0] | | |
| DC.29 | After [INSERT NAME IN DC.0] was born did any health care provider (such as doctor, nurse, paramedics, health worker) check on [INSERT NAME IN DC.0]’s health? | 1. No>>Skip to DC.32 2. Yes   98. Don’t know>>Skip to DC.32 |
| DC.30 | How long after the birth of [INSERT NAME IN DC.0] did that check take place?  INSTRUCTION: IF LESS THAN ONE DAY, RECORD HOURS; IF LESS THAN ONE WEEK, RECORD DAYS. | 1. Hours ___ ___ (0-24 hours) 2. Days ___ ___ (0-50 days) 3. Weeks ___ ___ (0-10 weeks) 4. Don’t know |
| DC.31 | Who checked on [INSERT NAME IN DC.0] health at that time? | 1. Doctor 2. ANM/nurse/midwife/LHV 3. Other Health personnel 4. Dhai 5. Friend/relative 6. No one 7. Other (Specify)   98. Don’t know |
| DC.32 | During the first 2 days after [INSERT NAME IN DC.0]’s birth did any health care provider do the following |  |
| DC.32.1 | Examine the cord | 1. No 2. Yes   98. Don’t know |
| DC.32.2 | Measure [INSERT NAME IN DC.0]’s temperature | 1. No 2. Yes   98. Don’t know |
| DC.32.3 | Tell you how to recognize if your baby needs immediate medical attention | 1. No 2. Yes   98. Don’t know |
| DC.32.4 | Talk with you about breastfeeding | 1. No 2. Yes   98. Don’t know |
| DC.32.5 | Observe [INSERT NAME IN DC.0] breastfeeding to see if you are doing it correctly | 1. No 2. Yes   98. Don’t know |
| DC.33 | During the first 2 days after birth, did any healthcare provider do the following? |  |
| DC.33.1 | Measure your blood pressure | 1. No 2. Yes   98. Don’t know |
| DC.33.2 | Discuss your vaginal bleeding with you | 1. No 2. Yes   98. Don’t know |
| DC.33.3 | Discuss family planning with you | 1. No 2. Yes   98. Don’t know |


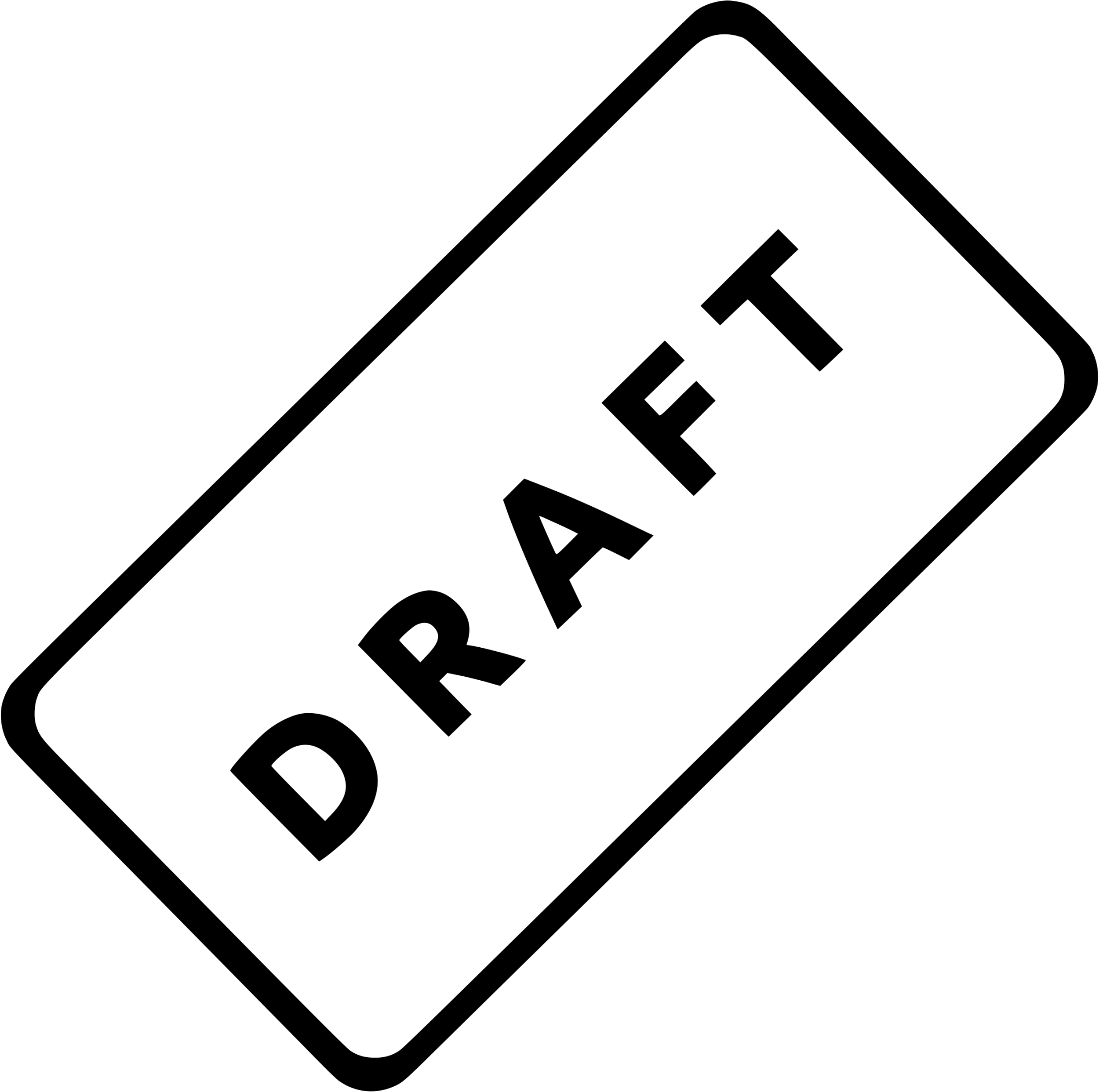
Module end time XX: XX

Module start time XX: XX

| **Nutrition support to mother (NS)** | | |
| --- | --- | --- |
| **Respondent: All WRA 15-49y with most recent livebirth in the last 2 years in the HH** | | |
| CAPI instructions:   - Repeat this section for all names listed in S.N.2 and S.N 3 (married adolescent) of the respondent matrix.   1. Add Respondent ID ___ - Skip if   1. BH.2=01 (Woman has not given birth) or   2. BH.12=0 (Woman has no live birth in the last 2 years) or   3. BH.13=0 (Woman has no still birth in the last 2 years) or - Administer for the most recent live birth this woman has had in the last two years, i.e name in BH.12.1 if BH.08=01 - Administer for all prior live births this woman has had in the last two years, i.e name in BH.14.1 if BH.08=01   NS.0=Display the name of the child | | |
| Now, I would like to ask about the nutrition support you may have received while breastfeeding (INSERT NAME IN BH.12.1) or during the first two years of (INSERT NAME IN NS.0)'s life. | | |
| **Q. no** | **Q. label** | **Response** |
| NS.1 | When you were breastfeeding [INSERT NAME IN NS.0], were you given, or did you buy any tablets or syrups that contain iron?  INSTRUCTION: SHOW VISUAL AID OF MMS TABLET & FULLCARE, COMMON TYPES OF MULTIPLE MICRONUTRIENT SUPPLEMENTS & COMMON TYPES OF IRON/IFA  Read aloud: Please think about these and similar products; the pictures are just examples | 1. 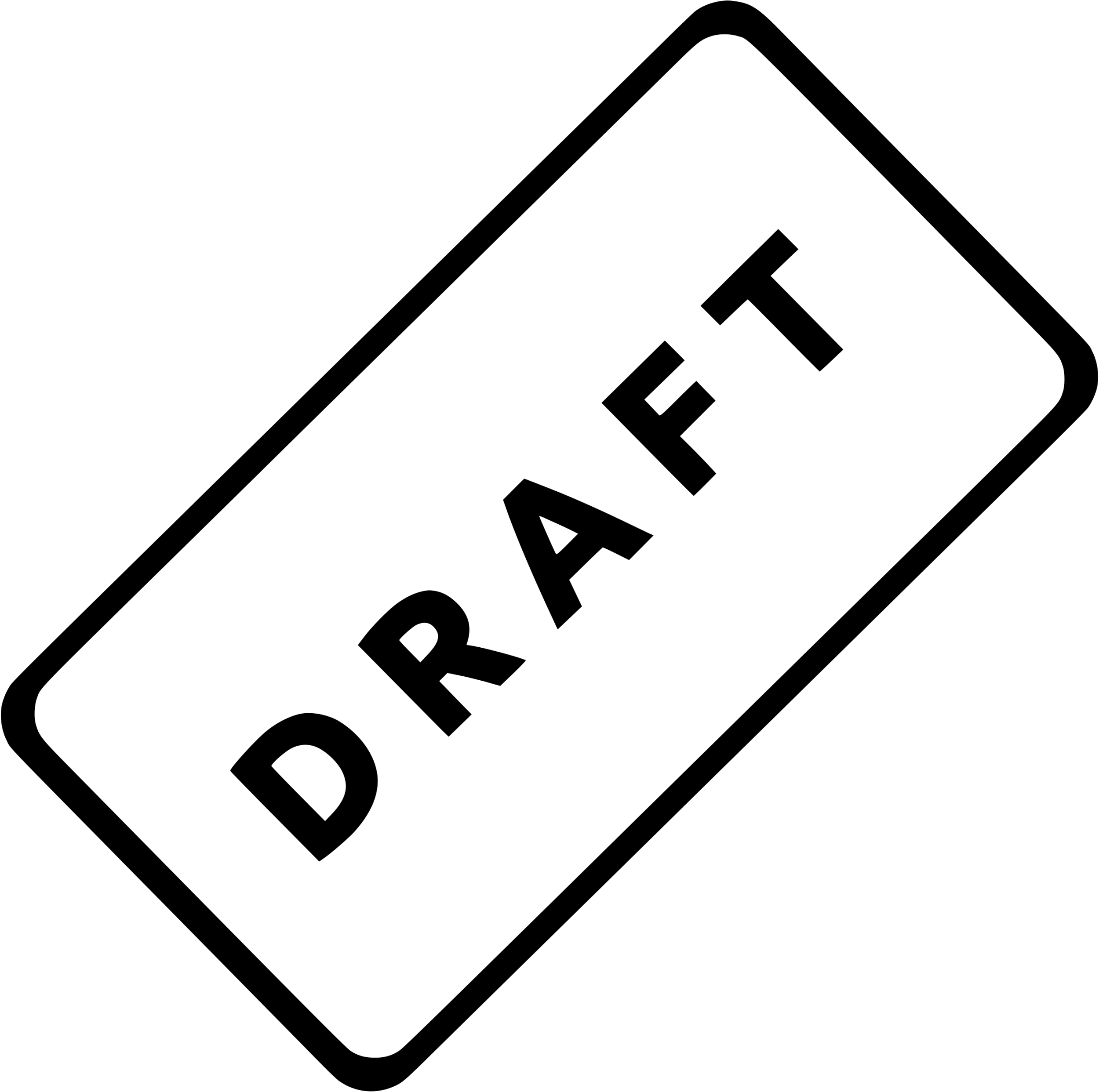No >> skip to NS.3 2. Yes   98. Don’t know >> skip to NS.3 |
| NS.2 | When you were breastfeeding [INSERT NAME IN NS.0], how many months did you take tablets or syrups that contain iron? | ___  (Record no. months)  (1-6 months)  98. Don’t know |
| NS.3 | When you were breastfeeding [INSERT NAME IN NS.0], were you ever diagnosed as undernourished? | 1. No >> skip to NS.5 2. Yes   98. Don’t know >> skip to NS.5 |
| NS.4 | Did you receive any information on how to prepare nutritious foods like khichuri and halwa from a health care provider when you were told you were underweight while breastfeeding? | 1. No 2. Yes   98. Don’t know |
| NS.5 | Were you offered paid maternity leave by your employer in the first six months after you gave birth to [INSERT NAME FROM NS.0]? | 1. No 2. Yes 3. I was not working |

Module end time __ __

Module start time XX: XX

| **Diet Quality Questionnaire (DQQ)-Woman** | | |
| --- | --- | --- |
| **Respondent: All WRA 15-49y and married adolescent 10-14y in the HH** | | |
| CAPI instruction:   - Repeat this section for all names listed in S.N.2 and S.N 3 (married adolescent) of the respondent matrix. - Add Respondent ID ___ | | |
| Now I’d like to ask you some yes-or-no questions about foods and drinks that you consumed yesterday during the day or night, whether you had it at home or somewhere else. First, I would like you to think about yesterday, from the time you woke up through the night. Think to yourself about the first thing you ate or drank after you woke up in the morning … Think about where you were when you had any food or drink in the middle of the day… Think about where you were when you had any evening meal … and any food or drink you may have had in the evening or late-night and any other snacks or drinks you may have had between meals throughout the day or night.  I am interested in whether you had the food items I will mention even if they were combined with other foods.  Please listen to the list of foods and drinks, and if you ate or drank ANY ONE OF THEM, say yes. | | |
|  | Yesterday, did you eat any of the following foods: |  |
| WDQQ.1 | Rice, paratha, or pa ruti? | 01=No, 02=Yes |
| WDQQ.2 | Roti, corn, or popcorn? | 01=No, 02=Yes |
| WDQQ.3 | Potato, plantain, arum, or sweet potato? | 01=No, 02=Yes |
| WDQQ.4 | Daal, 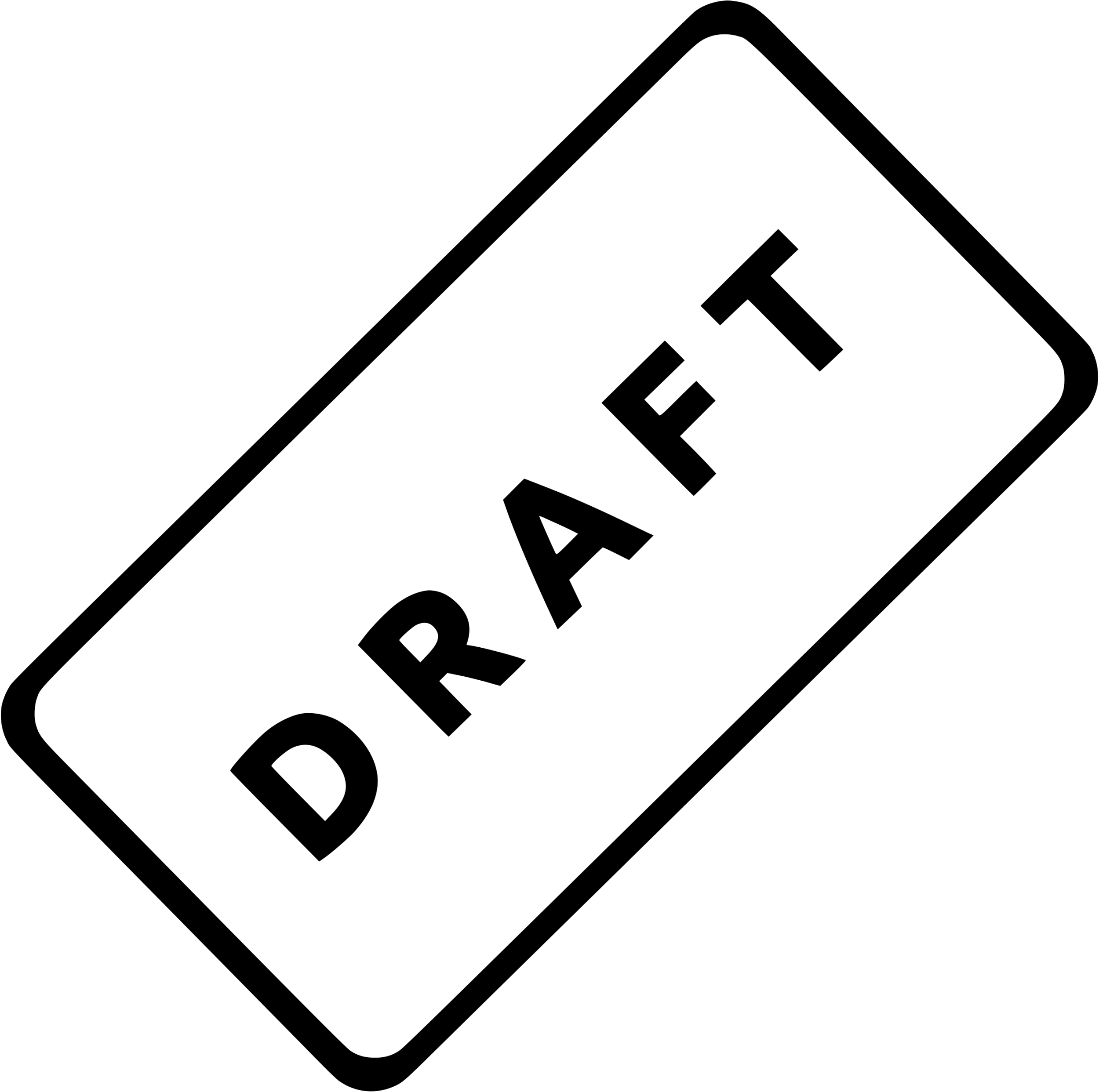chickpeas, or khichuri? | 01=No, 02=Yes |
|  | Yesterday, did you eat any of the following vegetables: |  |
| WDQQ.5 | Carrots or pumpkin? | 01=No, 02=Yes |
| WDQQ.6.1 | Lal shak, pui shak, amaranth, spinach, or any other shak? | 01=No, 02=Yes |
| WDQQ.7.1 | Eggplant, lady finger, cauliflower, cabbage, long beans, green beans, or tomatoes? | 01=No, 02=Yes |
| WDQQ.7.2 | Bottle gourd, pointed gourd, bitter gourd, bitter melon, or ash gourd? | 01=No, 02=Yes |
| WDQQ.7.3 | White radish, kohlrabi, taro shoots, or green papaya? | 01=No, 02=Yes |
|  | Yesterday, did you eat any of the following fruits: |  |
| WDQQ.8 | Ripe mango, ripe papaya, or orange musk melon? | 01=No, 02=Yes |
| WDQQ.9 | Orange, malta, or pomelo? | 01=No, 02=Yes |
| WDQQ.10.1 | Guava, pineapple, ripe banana, watermelon, jackfruit, custard apple, or apple? | 01=No, 02=Yes |
| WDQQ.10.2 | Jamrul, star fruit, koromcha, jujube, Java plum, litchi, or amra? | 01=No, 02=Yes |
|  | Yesterday, did you eat any of the following sweets: |  |
| WDQQ.11 | Sweet biscuits, cakes, misti pitha, halwa, or jilapi? | 01=No, 02=Yes |
| WDQQ.12 | Mishti, chocolate, or ice cream? | 01=No, 02=Yes |
|  | Yesterday, did you eat any of the following foods of animal origin: |  |
| WDQQ.13 | Eggs? | 01=No, 02=Yes |
| WDQQ.14 | Paneer or cheese? | 01=No, 02=Yes |
| WDQQ.15 | Yogurt or lassi? | 01=No, 02=Yes |
| WDQQ.16 | Sausages? | 01=No, 02=Yes |
| WDQQ.17 | Beef or goat meat? | 01=No, 02=Yes |
| WDQQ.19 | Chicken, chicken liver, pigeon, duck, or quail? | 01=No, 02=Yes |
| WDQQ.20 | Fish or dried fish? | 01=No, 02=Yes |
|  | Yesterday, did you eat any of the following other foods: |  |
| WDQQ.21 | Peanuts or jackfruit seeds? | 01=No, 02=Yes |
| WDQQ.22 | Chips or chanachur? | 01=No, 02=Yes |
| WDQQ.23 | Instant noodles such as Maggi noodles or Pran's Mr. Noodles? | 01=No, 02=Yes |
| WDQQ.24 | Puri, singara, samucha, pakora, piaju, beguni, fried chicken, or chop? | 01=No, 02=Yes |
|  | Yesterday, did you have any of the following beverages: |  |
| WDQQ.25 | Milk? | 01=No, 02=Yes |
| WDQQ.26 | Tea with sugar, coffee with sugar, chocolate milk, Horlicks, Milo, Complan or Ovaltine? | 01=No, 02=Yes |
| WDQQ.27 | Fruit juice, packet juice such as Frooto or Tang, or shorbot? | 01=No, 02=Yes |
| WDQQ.28 | Soft drinks such as Pepsi, Mojo, Sprite, or Fanta, or energy drinks such as Tiger? | 01=No, 02=Yes |
|  | Yesterday, did you get food from any place like... |  |
| WDQQ.29 | KFC, CP, Pizza Hut, Helvetia, Burger King, Herfy, or other places that serve pizza or burgers? | 01=No, 02=Yes |

Module end time XX: XX

| Module start time XX: XX | | | | | |
| --- | --- | --- | --- | --- | --- |
| **Nutrition sensitive social protection programs - CASH (SPC)** | | | | | |
| **Respondent- WRA 15-49 years. If a HH has more than 1 WRA than a respondent should be selected randomly** | | | | | |
| Now, I would like to ask you about various external cash assistance programs provided to households and their members. By external assistance,I mean support that comes from government or non-governmental organizations such as religious, charitable or community organizations. This excludes the support of the family, other relatives, friends, or neighbors. | | | | | |
| SPC.1 | In the past 12 months, has any member of your household received any cash or monetary assistance from the government or any other non-governmental organizations?  [SINGLE SELECT] | 1. No >> *skip to section SPF.1* 2. Yes 3. Don’t know>> *skip to section SPF.1* | | | |
| SPC.2 | In the past 12 months, who in your household received cash or monetary assistance from the government or any other non-governmental organizations?  Record member ID from HR.1  Entire household = 96  Don’t’ know = 98  [SINGLE SELECT] | Mem 1 | Mem 2 | Mem 3 | Mem 4 |
|  |  | Mem ID | Mem ID | Mem ID | Mem ID |
|  |  |  |  |  |  |
| SPC.3 | With the cash or monetary assistance that [INSERT NAME FROM SPC.2] received, did they also receive following?  **[read aloud**]   1. Nutrition or health counseling 2. Told to go to a health facility to receive health or nutrition services 3. Tablets to treat intestinal worms 4. Iron tablets or other nutrient supplements 5. Food with extra nutrients added to it to benefit health 6. Other (specify)   [MULTI-SELECT] | 1. No 2. Yes 3. Don’t know | 1. No 2. Yes 3. Don’t know | 1. No 2. Yes 3. Don’t know | 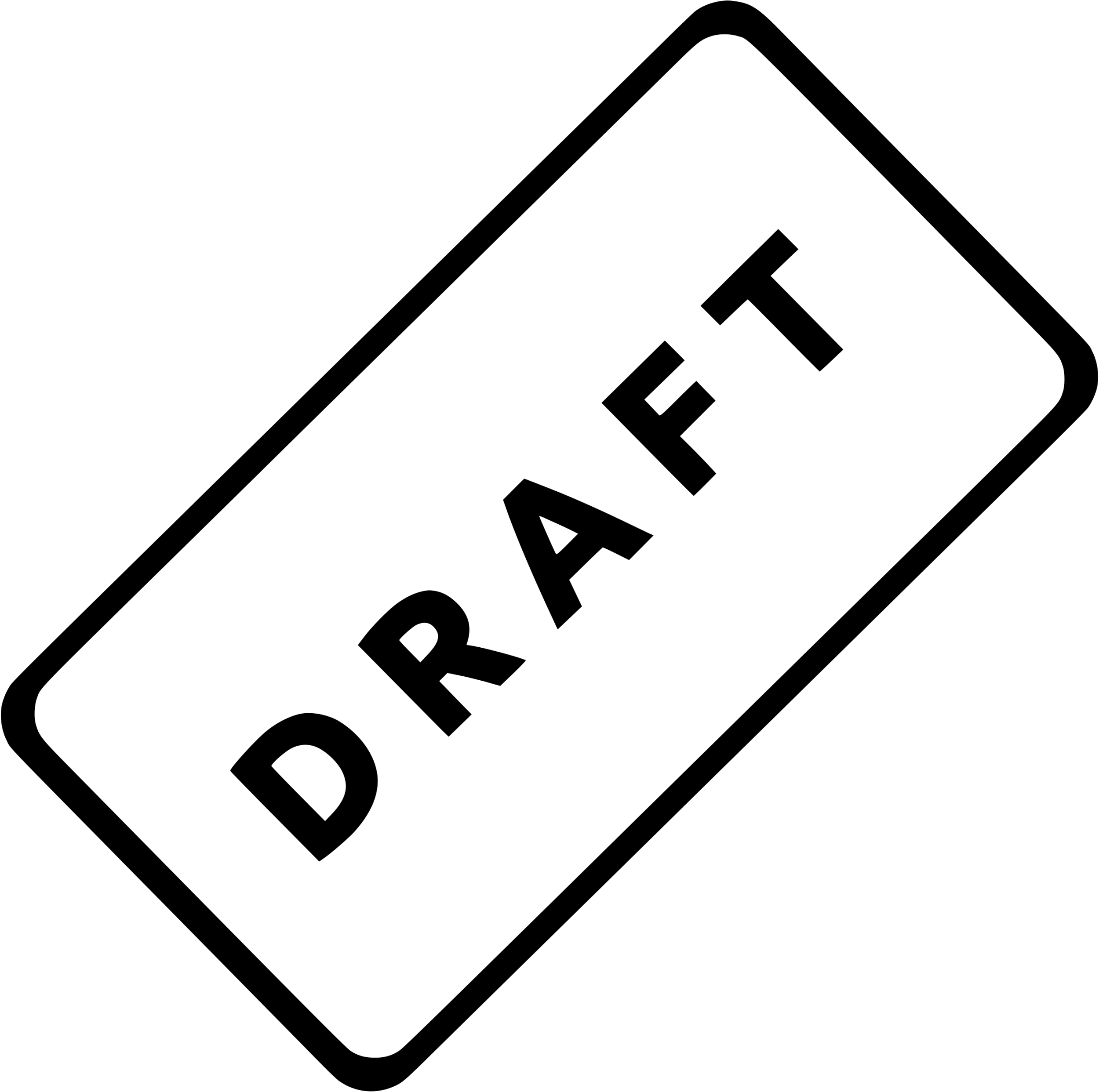   1. No 2. Yes 3. Don’t know |
| SPC.4 | To receive the cash or monetary assistance, did [INSERT NAME FROM SPC.2] or anyone in your household have to do any specific activity such as attend a meeting, take a child to the health facility or anything else? If so, what was it? | 1. No   Yes, (specify) _________ | 1. No   Yes, (specify) ___________ | 1. No   02. Yes, (specify) _____________ | 1. No   02. Yes, (specify) ______________ |
| SPC.5 | When was the last time [INSERT NAME FROM SPC.2] received cash or monetary assistance from the government or any other non-governmental organizations? | [MM/YYYY] | [MM/YYYY] | [MM/YYYY] | [MM/YYYY] |
| SPC.6 | How often did [INSERT NAME FROM SPC.2] receive cash or monetary assistance from the government or any other non-governmental organizations?  [SINGLE SELECT] | 1. Daily 2. Few times a week but not daily 3. Weekly 4. Monthly 5. Annually 6. Other (specify) 7. Don’t know | 1. Daily 2. Few times a week but not daily 3. Weekly 4. Monthly 5. Annually 6. Other (specify) 7. Don’t know | 1. Daily 2. Few times a week but not daily 3. Weekly 4. Monthly 5. Annually 6. Other (specify) 7. Don’t know | 1. Daily 2. Few times a week but not daily 3. Weekly 4. Monthly 5. Annually 6. Other (specify) 7. Don’t know |

Module end time XX: XX

Module start time XX: XX

| **Nutrition sensitive social protection programs - FOOD (SPF)** | | | | | |
| --- | --- | --- | --- | --- | --- |
| **Respondent- WRA 15-49 years. If a HH has more than 1 WRA than respondent should be selected randomly** | | | | | |
| Now, I would like to ask you about various external food assistance programs provided to households and their members. By external assistance, I mean support that comes from government or non-governmental organizations such as religious, charitable or community organizations. This excludes the support of the family, other relatives, friends, or neighbors. | | | | | |
| SPF.1 | In the past 12 months, has any member of your household received any free food or subsidized food from the government or any other non-government organizations?  [SINGLE SELECT] | 1. No >> *skip to section SPI.1* 2. Yes 3. Don’t know>> *skip to section SPI.1* | | | |
| SPF.2 | In the past 12 months, who in your household received free food or subsidized food from the government or any other non-government organizations?  Record member ID from HR.1  Entire household = 96  Don’t’ know = 98  [SINGLE SELECT] | Mem 1 | Mem 2 | Mem 3 | Mem 4 |
|  |  | Mem ID | Mem ID | Mem ID | Mem ID |
|  |  |  |  |  | 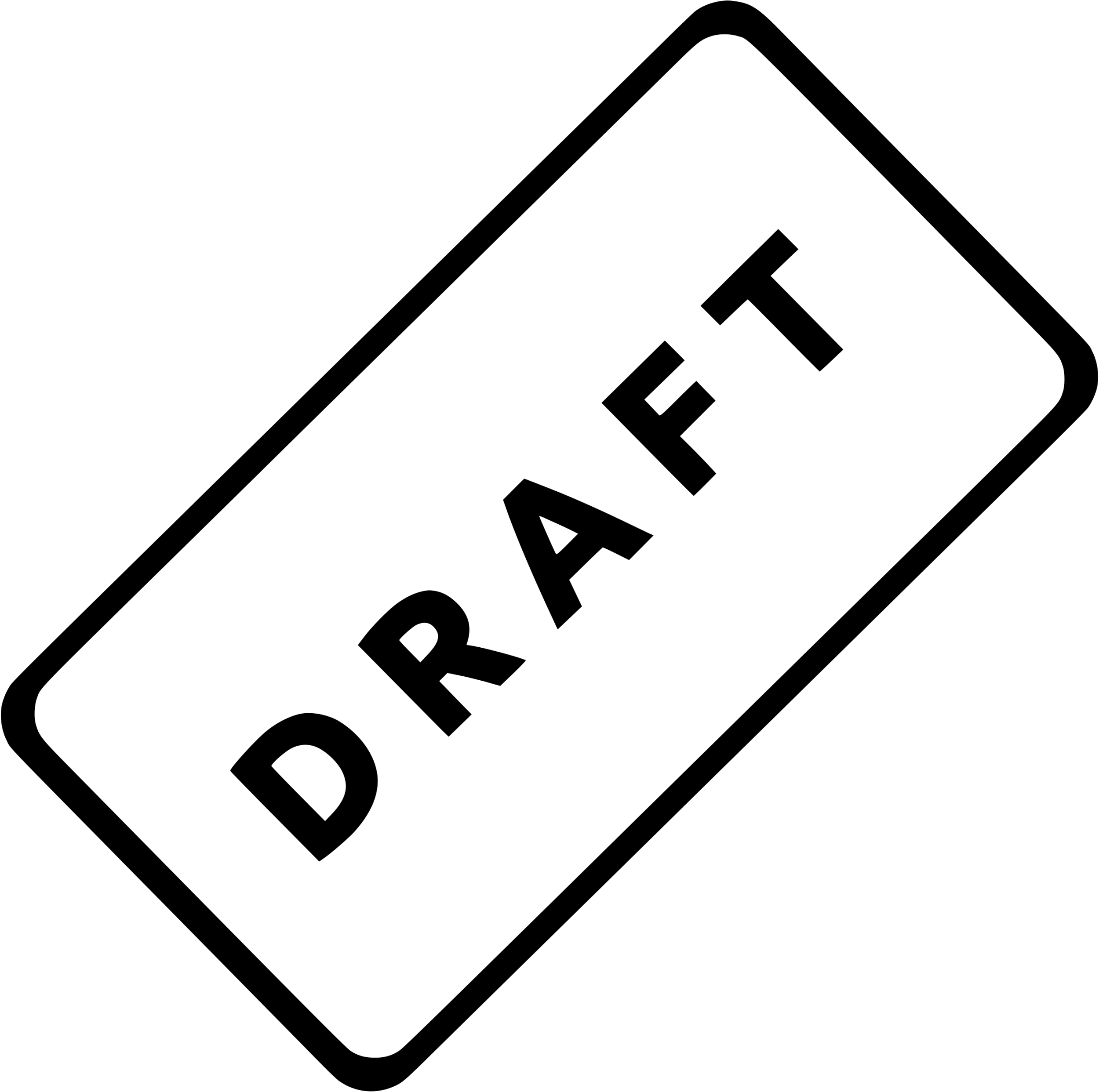 |
| SPF.3 | With the free food or subsidized food that [INSERT NAME FROM SPF.2] received, did they also receive the following?  [READ ALOUD]   1. Nutrition or health counseling 2. Told to go to a health facility to receive health or nutrition services 3. Tablets to treat intestinal worms 4. Iron tablets or other nutrient supplements 5. Food with extra nutrients added to it to benefit health 6. Other (specify)   [MULTI-SELECT] | 1. No 2. Yes 3. Don’t know | 1. No 2. Yes 3. Don’t know | 1. No 2. Yes 3. Don’t know | 1. Yes 2. No 3. 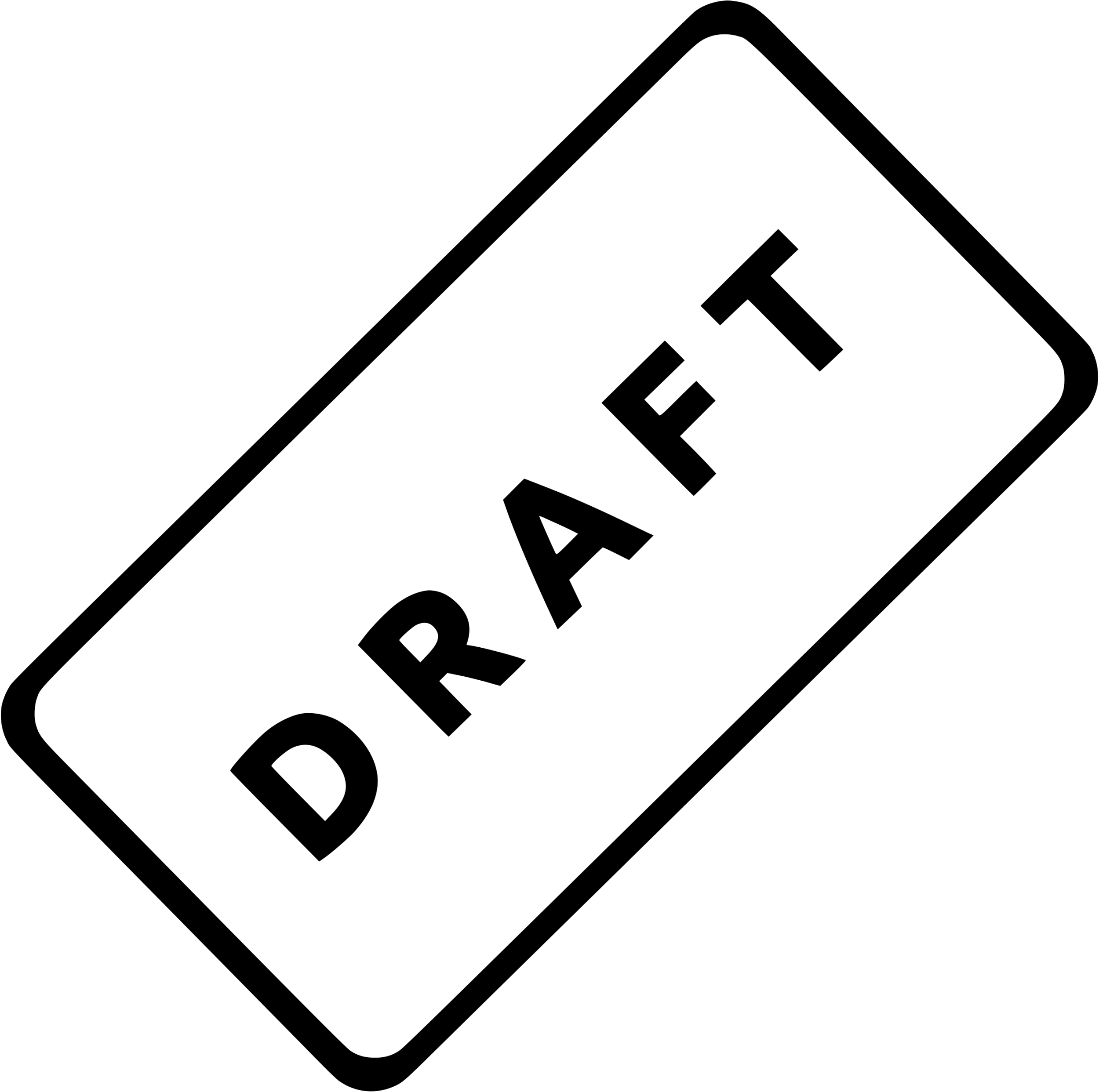Don’t know |
| SPF.4 | To receive the free food or subsidized food, did [INSERT NAME FROM SPF.2] or anyone in your household have to do any specific activity such as attend a meeting, take a child to the health facility or anything else? If so, what was it? | 1. No 2. Yes, (specify)   ___________ | 1. No   02. Yes,(specify) _____________ | 1. No 2. Yes, (specify)   ___________ | 1. No 2. Yes, (specify)   ___________ |
| SPF.5 | When was the last time [INSERT NAME FROM SPF.2] received free food or subsidized food from the government or any other non-governmental organizations? | [MM/YYYY] | [MM/YYYY] | [MM/YYYY] | [MM/YYYY] |
| SPF.6 | How often did INSERT NAME FROM SPF.2] receive free food or subsidized food from the government or any other non-governmental organization?  [SINGLE SELECT] | 1. Daily 2. Few times a week but not daily 3. Weekly 4. Monthly 5. Annually 6. Other (specify) 7. Don’t know | 1. Daily 2. Few times a week but not daily 3. Weekly 4. Monthly 5. Annually 6. Other (specify) 7. Don’t know | 1. Daily 2. Few times a week but not daily 3. Weekly 4. Monthly 5. Annually 6. Other (specify) 7. Don’t know | 1. Daily 2. Few times a week but not daily 3. Weekly 4. Monthly 5. Annually 6. Other (specify) 7. Don’t know |

Module end time XX: XX

Module start time XX: XX

| **Nutrition sensitive social protection programs – IN-KIND (SPI)** | | | | | |
| --- | --- | --- | --- | --- | --- |
| **Respondent: Head of household** | | | | | |
| Now, I would like to ask you about various external in-kind assistance (for example: improved seed) provided to households and their members. By external assistance, I mean support that comes from government or non-governmental organizations such as religious, charitable or community organizations. This excludes the support of the family, other relatives, friends, or neighbors. | | | | | |
| SPI.1 | In the past 12 months, has any member of your household received any in-kind from the government or any other non-governmental organizations?  [SINGLE SELECT] | 1. No >> *skip to section SFY.1* 2. Yes, specify __________________ 3. Don’t know>> *skip to section SFY.1* | | | |
| SPI.2 | In the past 12 months, who in your household received in-kind transfer from the government or any other non-governmental organizations?  Record member ID from HR.1  Entire household = 96  Don’t’ know = 98  [SINGLE SELECT] | Mem 1 | Mem 2 | Mem 3 | Mem 4 |
|  |  | Mem ID | Mem ID | Mem ID | Mem ID |
|  |  |  |  |  |  |
| SPI.3 | With the in-kind transfer that [INSERT NAME FROM SPI.2] received, did they also receive the following?  [READ ALOUD]  **[read aloud**]   1. Nutrition or health counseling 2. Told to go to a health facility to receive health or nutrition services 3. Tablets to treat intestinal worms 4. Iron tablets or other nutrient supplements 5. Food with extra nutrients added to it to benefit health 6. Other (specify)   [MULTI-SELECT] | 1. No 2. Yes 3. Don’t know | 1. No 2. Yes 3. Don’t know | 1. No 2. Yes 3. Don’t know | 1. 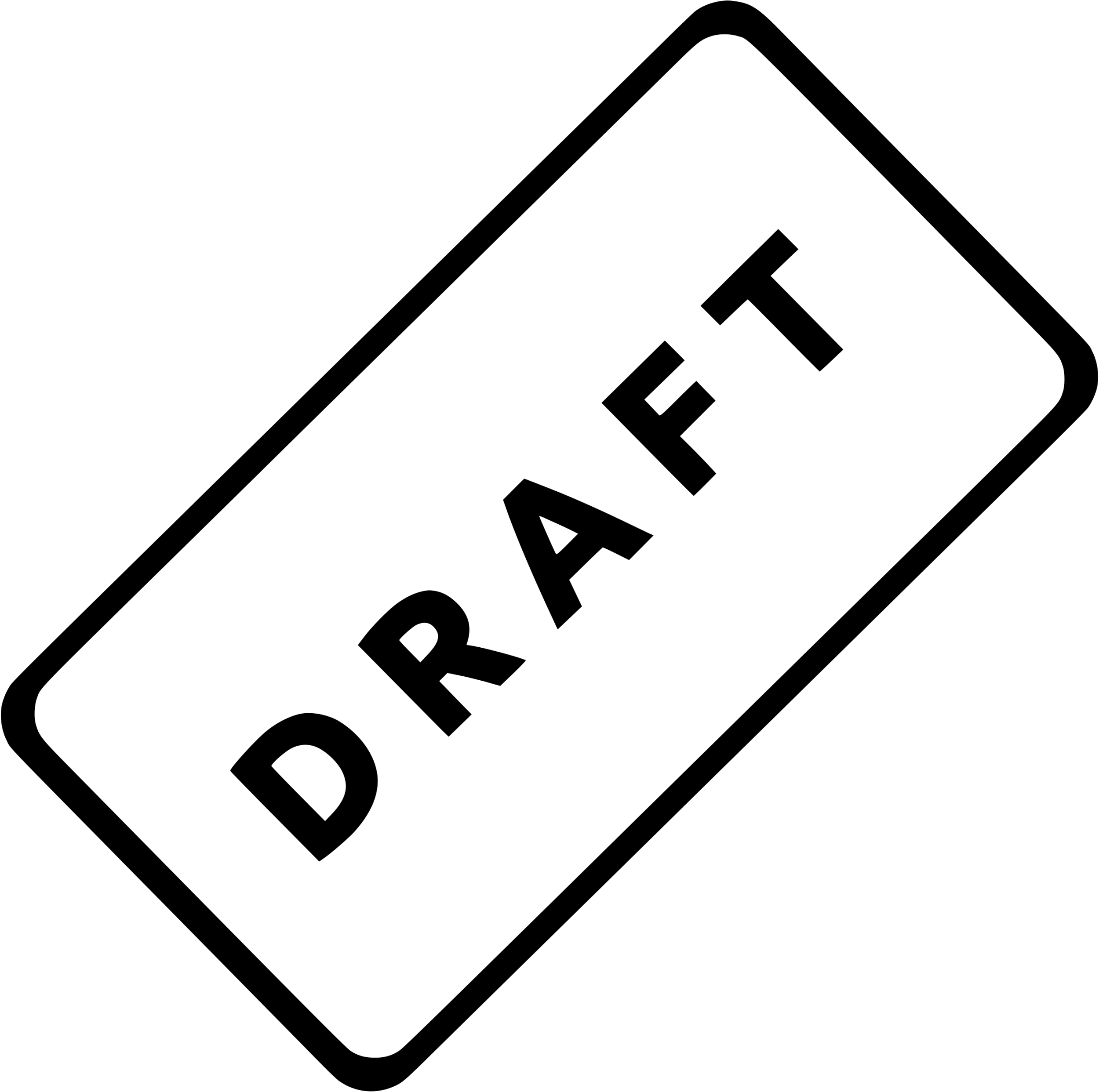Yes 2. No 3. Don’t know |
| SPI.4 | To receive the in-kind transfer, did [INSERT NAME FROM SPI.2] or anyone in your household have to do any specific activity such as attend a meeting, take a child to the health facility or anything else? If so, what was it? | 1. No 2. Yes, (specify)   ___________ | 1. No   02. Yes,(specify) _____________ | 1. No 2. Yes, (specify)   ___________ | 1. No 2. Yes, (specify)   ___________ |
| SPI.5 | When was the last time [INSERT NAME FROM SPI.2] received in-kind transfer from the government or any other non-governmental organization? | [MM/YYYY] | [MM/YYYY] | [MM/YYYY] | [MM/YYYY] |
| SPI.6 | How often did INSERT NAME FROM SPI.2] receive the in-kind transfer from the government or any other non-governmental organization?  [SINGLE SELECT] | 1. Daily 2. Few times a week but not daily 3. Weekly 4. Monthly 5. Annually 6. Other (specify) 7. Don’t know | 1. Daily 2. Few times a week but not daily 3. Weekly 4. Monthly 5. Annually 6. Other (specify) 7. Don’t know | 1. Daily 2. Few times a week but not daily 3. Weekly 4. Monthly 5. Annually 6. Other (specify) 7. Don’t know | 1. 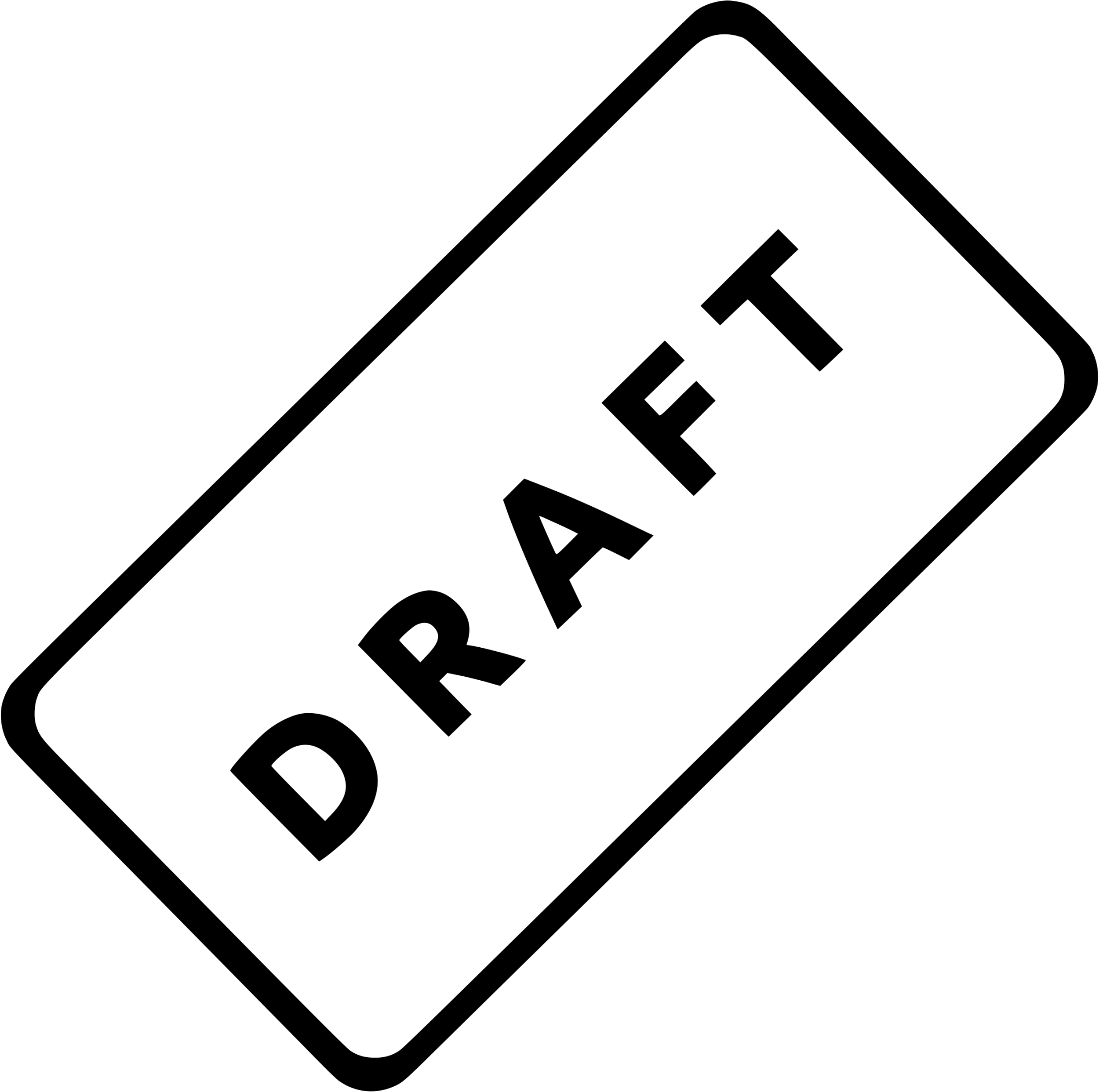Daily 2. Few times a week but not daily 3. Weekly 4. Monthly 5. Annually 6. Other (specify) 7. Don’t know |

Module end time XX: XX

Module start time XX: XX

| **School Feeding (SFY)** | | | | | |
| --- | --- | --- | --- | --- | --- |
| **Respondent- WRA 15-49 years. If a HH has more than 1 WRA than respondent should be selected randomly** | | | | | |
| Now, I would like to ask you about the meal that your child receives from school. | | | | | |
| SFY.1 | In the past 12 months, has any member of your household received free food from school? | 1. No>>skip to CI.1 2. Yes 3. Don’t know>>skip to CI.1 | | | |
| SFY.2 | Which child in your household received free food from school?  Record member ID from HR.1  Don’t’ know = 98  [SINGLE SELECT] | Mem 1 | Mem 2 | Mem 3 | Mem 4 |
|  |  | Mem ID | Mem ID | Mem ID | Mem ID |
|  |  |  |  |  |  |
| SFY.3 | With the free food that [INSERT NAME FROM SFY.2] received, did they also receive following?  **READ ALOUD**   1. Nutrition or health education 2. Told to go to a health facility to receive health or nutrition services 3. Tables to treat intestinal worms 4. Iron tablets or other nutrient supplements 5. Food with extra nutrients added to it to benefit health 6. Other (specify)   [MULTI SELECT] | 1. No 2. Yes 3. Don’t know | 1. No 2. Yes 3. Don’t know | 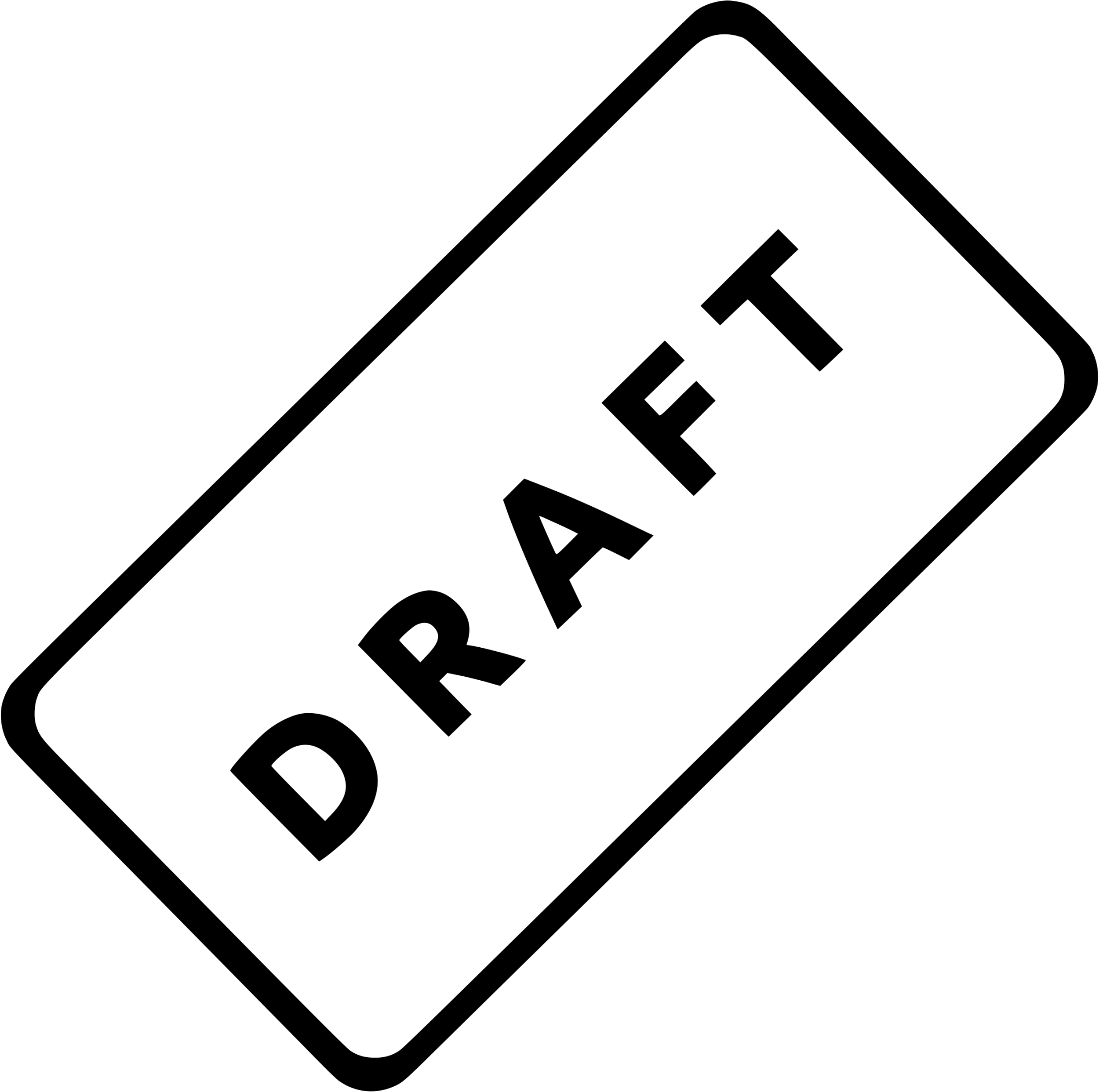   1. No 2. Yes 3. Don’t know | 1. No 2. Yes 3. Don’t know |
| SFY.4 | When was the last time [INSERT NAME FROM SFY.2] received free food from the school? | [MM/YYYY] | [MM/YYYY] | [MM/YYYY] | [MM/YYYY] |
| SFY.5 | How often did INSERT NAME FROM SFY.2] receive free food from the school?  [SINGLE SELECT] | 1. Daily 2. Few times a week but not daily 3. Weekly 4. Monthly 5. Annually 6. Other (specify) 7. Don’t know | 1. Daily 2. Few times a week but not daily 3. Weekly 4. Monthly 5. Annually 6. Other (specify) 7. Don’t know | 1. Daily 2. Few times a week but not daily 3. Weekly 4. Monthly 5. Annually 6. Other (specify) 7. Don’t know | 1. Daily 2. Few times a week but not daily 3. Weekly 4. Monthly 5. Annually 6. Other (specify) 7. Don’t know |

Module end time XX: XX

Module start time XX: XX

| **Child Immunization (CI), Children 0-23m** | | |
| --- | --- | --- |
| **Respondent: All WRA 15-49y and adolescent with a child 0-23m and a primary caregiver of a child 0-23m whose biological mother is either not alive or does not live in the household** | | |
| CAPI instructions:   - Repeat this section for all names listed in S.N.2, S.N 3 and S.N 4 of the respondent matrix.   Add Respondent ID __   - Skip if   1. BH.2=01 (Woman has never given a birth) or   2. BH.12=0 (Woman has no live birth in the last 2 years) or   3. BH.13=0 (Woman has no still birth in the last 2 years) or   4. BH.12.1!=. and BH.8=01 and BH.9=01 (Most recent livebirth in the last 2 years is not currently alive) - Repeat this section for all currently living children 0-23 months. For name in BH.12.1 if (BH.8=01 and BH.9=02) all name in BH.14.1 if BH.9=02   CI.0 Display the name of the child | | |
| Now, we would like to ask you some questions related to vaccination of [INSERT THE NAME IN CI.0] | | |
| **Q. no** | **Q. label** | **Response** |
| CI.1 | Do you have a card or other document where [INSERT THE NAME IN CI.0] vaccinations are written down? | 1. Yes, has only a card 2. Yes, has only another document 3. Yes, has card and other document 4. No, no card and no other document>>skip to CI.4 |
| CI.2 | May I see the card or other document where [INSERT THE NAME IN CI.0]'s vaccinations are written down? | 1. Yes, only card seen 2. Yes, only other document seen 3. Yes, card and other document seen 4. No, no card and no other document seen>>skip to CI.4 |
| CI.3 | 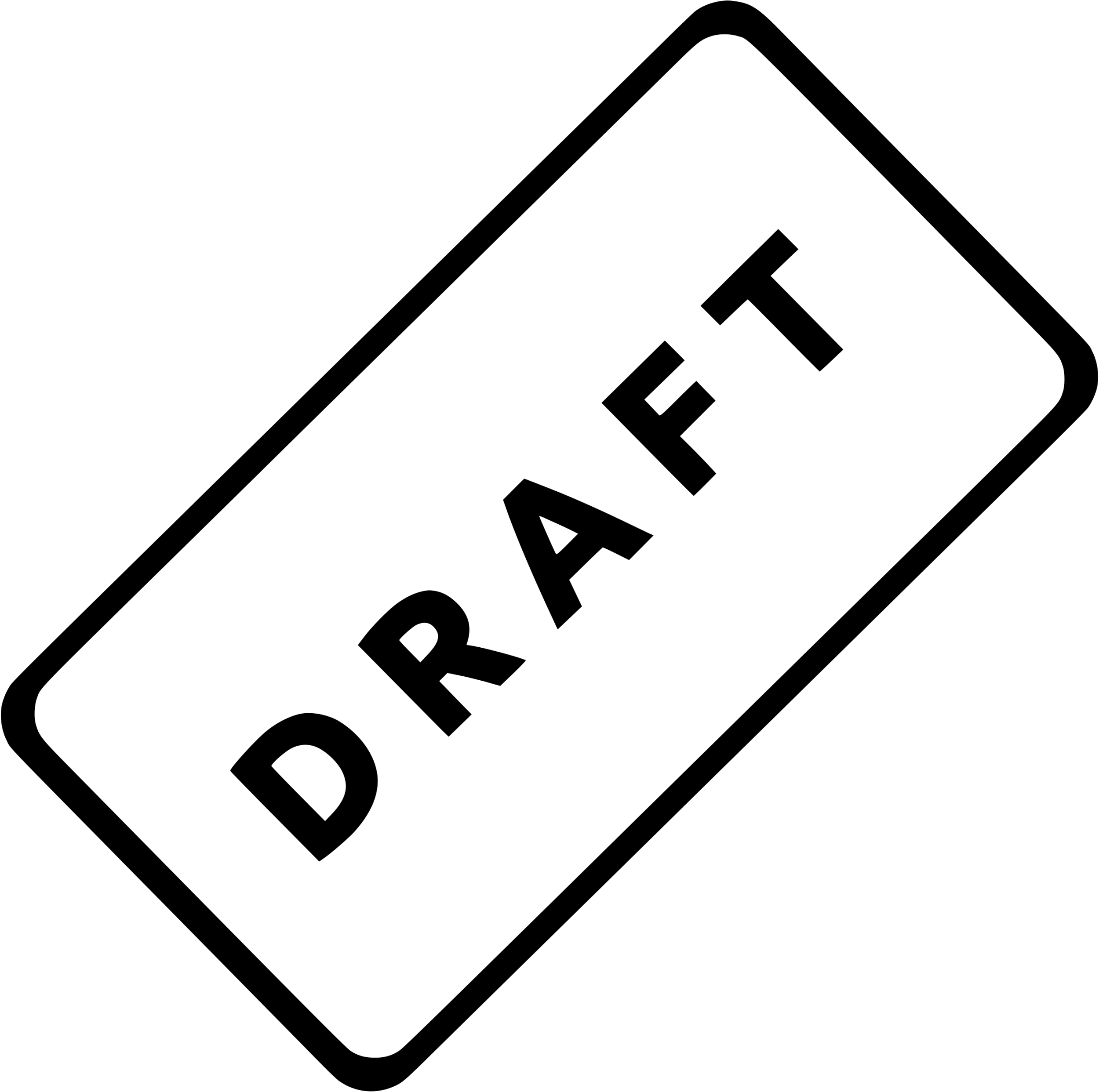INSTRUCTION: COPY DATES FROM THE CARD.  WRITE ‘44' IN ‘DAY' COLUMN IF CARD SHOWS THAT A DOSE WAS GIVEN, BUT NO DATE IS   \|  \| DAY \| \| MONTH \| \| YEAR \| \| \| \| \| --- \| --- \| --- \| --- \| --- \| --- \| --- \| --- \| --- \| \| BCG \|  \|  \|  \|  \|  \|  \|  \|  \| \| PENTA 1 \|  \|  \|  \|  \|  \|  \|  \|  \| \| PENTA 2 \|  \|  \|  \|  \|  \|  \|  \|  \| \| PENTA 3 \|  \|  \|  \|  \|  \|  \|  \|  \| \| OPV/POLIO 1 \|  \|  \|  \|  \|  \|  \|  \|  \| \| OPV/POLIO 2 \|  \|  \|  \|  \|  \|  \|  \|  \| \| OPV/POLIO 3 \|  \|  \|  \|  \|  \|  \|  \|  \| \| PCV/PNEUMOCOCCAL 1 \|  \|  \|  \|  \|  \|  \|  \|  \| \| PCV/PNEUMOCOCCAL 2 \|  \|  \|  \|  \|  \|  \|  \|  \| \| PCV/PNEUMOCOCCAL 3 \|  \|  \|  \|  \|  \|  \|  \|  \| \| IPV \|  \|  \|  \|  \|  \|  \|  \|  \| \| fIPV 6 WEEKS \|  \|  \|  \|  \|  \|  \|  \|  \| \| fIPV 14 WEEKS \|  \|  \|  \|  \|  \|  \|  \|  \| \| MR AT 9 MONTHS \|  \|  \|  \|  \|  \|  \|  \|  \| \| MR AT 15 MONTHS \|  \|  \|  \|  \|  \|  \|  \|  \| \| VITAMIN A (MOST RECENT) \|  \|  \|  \|  \|  \|  \|  \|  \| | |
| CI.4 | Has [INSERT THE NAME IN CI.0] ever received a BCG vaccination against tuberculosis, that is, an injection in the left upper arm or shoulder that usually causes a scar? | 1. No 2. Yes 3. Don’t know |
| CI.5 | Has [INSERT THE NAME IN CI.0] ever received a pentavalent vaccination, that is, an injection given in the thigh at the same time as polio drops and PCV? | 1. No>>skip to CI.6 2. Yes 3. Don’t know>>skip to CI.6 |
| CI.5A | How many times did [INSERT THE NAME IN CI.0] receive the pentavalent vaccine? | ___  (Record no. times)  (0-5 times)  Don’t know |
| CI.6 | Has [INSERT THE NAME IN CI.0] ever received oral polio vaccine, that is, about two drops in the mouth to prevent polio? | 1. No>>skip to CI.7 2. Yes 3. Don’t know>>skip to CI.7 |
| CI.6A | Did [INSERT THE NAME IN CI.0] receive the first oral polio vaccine in the first two weeks after birth or later? | 1. No 2. Yes 3. Don’t know |
| CI.6B | How many times did [INSERT THE NAME IN CI.0] receive the oral polio vaccine? | 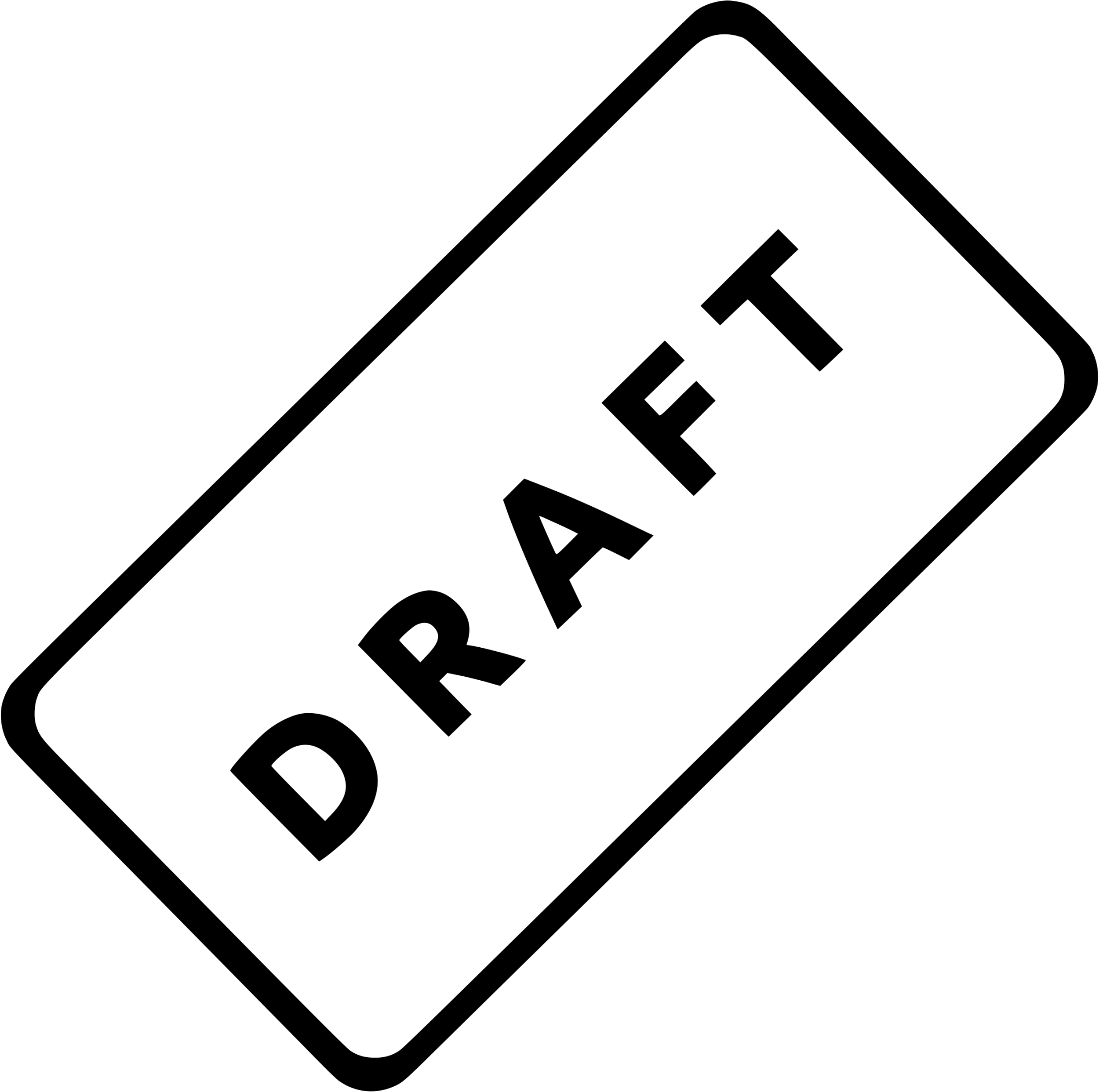  ___  (Record no. times)  (0-5 times)  Don’t know |
| CI.7 | Has [INSERT THE NAME IN CI.0] ever received a pneumococcal vaccination, that is, an injection in the thigh to  prevent pneumonia? | 1. No>>skip to CI.8 2. Yes 3. Don’t know>>skip to CI.8 |
| CI.7A | How many times did [INSERT THE NAME IN CI.0] receive pneumococcal vaccination? | ___  (Record no. times)  (0-5 times)  Don’t know |
| CI.8 | Has [INSERT THE NAME IN CI.0] ever received an IPV vaccination, that is, an injection in the thigh to prevent polio? | 1. No 2. Yes 3. Don’t know |
| CI.9 | Has [INSERT THE NAME IN CI.0] ever received a measles-rubella vaccination, that is, an injection into the muscles of the left thigh to prevent measles? | 1. No>>skip to CI.11 2. Yes 3. Don’t know>>skip to CI.11 |
| CI.10 | How many times did [INSERT THE NAME IN CI.0] receive the measles-rubella vaccine? | ___  (Record no. times)  (0-5 times)  Don’t know |
| CI.11 | Did [INSERT THE NAME IN CI.0] receive any polio vaccine from the National Immunization Days (NIDs)? | 1. No 2. Yes 3. Don’t know |
| CI.12 | Did [INSERT THE NAME IN CI.0] receive any measles-rubella vaccine from the National Measles-Rubella Campaign? | 1. No 2. Yes 3. Don’t know |

Module end time XX: XX

Module start time XX: XX

| **Diet Quality Questionnaire (DQQ), Child 6-23m** | | | |
| --- | --- | --- | --- |
| **Respondent: All WRA 15-49y and adolescent with a child 6-23m and a primary caregiver of a child 6-23m whose biological mother is either not alive or does not live in the household** | | | |
| CAPI instructions-   - Repeat this section for all names listed in S.N.2, S.N 3 and S.N 4 (only 6-23m) of the respondent matrix.   Add Respondent ID __   - Skip if   1. BH.2=01 (Woman has not given birth) or   2. BH.12=0 (Woman has no live birth in the last 2 years) or   3. BH.13=0 (Woman has no still birth in the last 2 years) or   4. BH.12.1!=. and BH.8=01 and BH.9=01 (Most recent livebirth in the last 2 years is not currently alive) - Restrict this section for 6-23 months children - Repeat for all currently living children 0-23 months. For name in BH.12.1 if (BH.8=01 and BH.9=02) all name in BH.14.1 if BH.9=02   CDQQ.0 Display the name of the child | | | |
| CDQQ.1 | **Was [INSERT THE NAME IN CDQQ.0] ever breastfed?** | 01=No, 02=Yes,98=DK | |
|  | *Note for interviewer: If answer is "no", skip to question 5. Do not record any answers for questions 2, 3, or 4.* |  | |
| CDQQ.2 | **How long after birth was [INSERT THE NAME IN CDQQ.0] first put to the breast?** |  | |
|  | If immediately, circle "000" |  | |
|  | If less than one hour, record "00" hours |  | |
|  | If less than 24 hours, record hours |  | |
|  | Otherwise, record days |  | |
| CDQQ.3 | **In the first 2 days after delivery, was [INSERT THE NAME IN CDQQ.0] given anything other than breastmilk to eat or drink – anything at all like water, infant formula or powdered milk, honey, or sugar water?** | 01=No, 02=Yes,98=DK | |
| CDQQ.4 | **Was [INSERT THE NAME IN CDQQ.0] breastfed yesterday during the day or at night?** | 01=No, 02=Yes,98=DK | |
| CDQQ.5 | **Did [INSERT THE NAME IN CDQQ.0] drink anything from a bottle with a nipple yesterday during the day or at night?** | 01=No, 02=Yes,98=DK | |
| CDQQ.6 | Now I would like to ask you about liquids that [INSERT THE NAME IN CDQQ.0] may have had yesterday during the day or at night. Please tell me about all drinks, whether [INSERT THE NAME IN CDQQ.0] had them at home, or somewhere else. Yesterday during the day or at night, did [INSERT THE NAME IN CDQQ.0] have... | 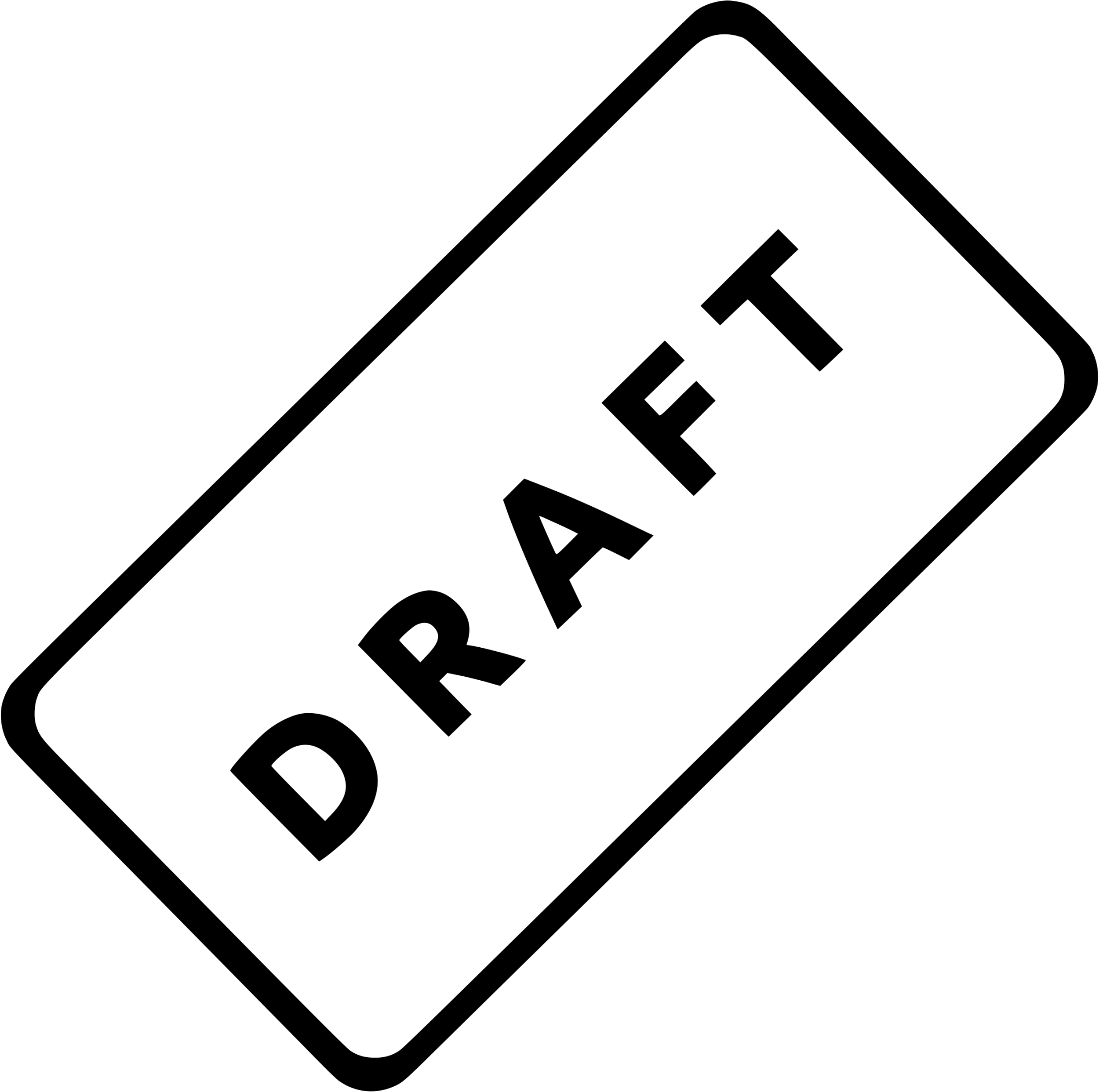 | |
| CDQQ.6A | Plain water? | 01=No, 02=Yes,98=DK | |
| CDQQ.6B | Gura dudh such as Lactogen, Nido, Bimil, Biomil, or Nan? | 01=No, 02=Yes,98=DK | |
| CDQQ.6Bnum | IF YES: How many times did (NAME) drink gura dudh? (IF 7 OR MORE TIMES, RECORD '7'). | #, or 98=DK | |
| CDQQ.6C.25 | **Fresh milk, packaged fluid milk, or dried milk such as Dano or Marks?** | 01=No, 02=Yes,98=DK | |
| CDQQ.6Cnum | IF YES: How many times did (NAME) drink milk? (IF 7 OR MORE TIMES, RECORD '7'). | #, or 98=DK | |
| CDQQ.6Cswt.26 | IF YES: Was any of the milk a sweet or flavoured type of milk? | 01=No, 02=Yes,98=DK | |
| CDQQ.6E | **Horlicks, Milo, Complan or Ovaltine?** |  | |
| CDQQ.6F.27 | **Fruit juice, packet juice such as Frooto or Tang, or shorbot?** | 01=No, 02=Yes,98=DK | |
| CDQQ.6G.28 | **Soft drinks such as Pepsi, Mojo, Sprite, or Fanta, or energy drinks such as Tiger?** | 01=No, 02=Yes,98=DK | |
| CDQQ.6H | **Tea, coffee, or herbal drinks?** | 01=No, 02=Yes,98=DK | |
| CDQQ.6Hswt.26 | IF YES: was the drink sweetened? | 01=No, 02=Yes,98=DK | |
| CDQQ.6I | Clear broth or clear soup? | 01=No, 02=Yes,98=DK | |
| CDQQ.6J | Any other liquids? | 01=No, 02=Yes,98=DK | |
|  | IF YES: What was the liquid or what were the liquids? _______ |  | |
| CDQQ.6Jswt | IF YES: Was the drink sweetened? | 01=No, 02=Yes,98=DK | |
| CDQQ.7 | Now I would like to ask you about foods that [INSERT THE NAME IN CDQQ.0] had yesterday during the day or at night. I am interested in foods your child ate whether at home or somewhere else. Please think about snacks and small meals as well as main meals.  I 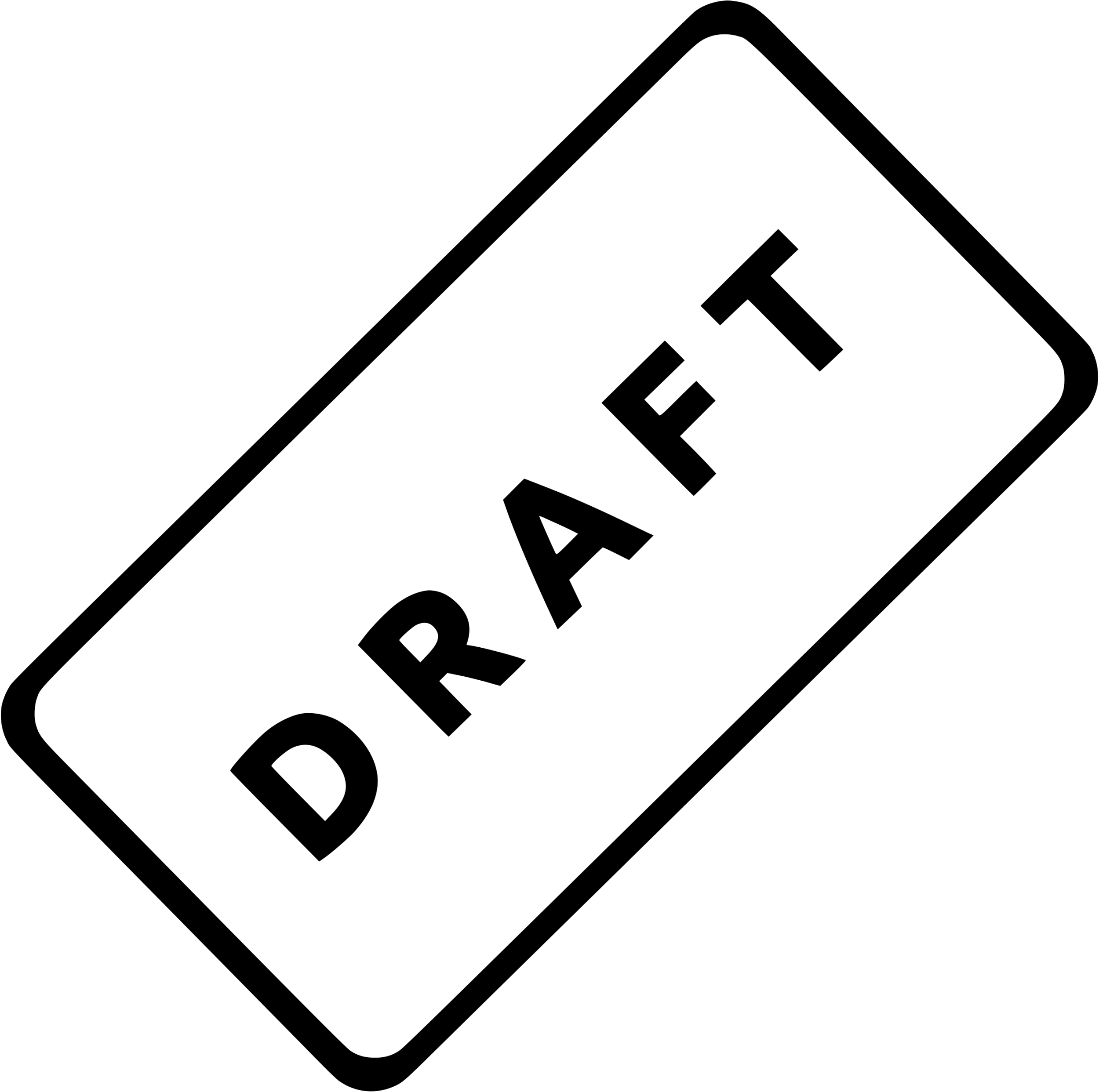will ask you about different types of foods, and I would like to know whether your child ate the food even if it was combined with other foods. Please do not answer ‘yes’ for any food or ingredient used in a small amount to add flavour to a dish.  Yesterday during the day or at night, did [INSERT THE NAME IN CDQQ.0] eat: | | |
| CDQQ.7.15 | Yogurt or lassi? | |  |
| CDQQ.7.15num | IF YES: How many times did [INSERT THE NAME IN CDQQ.0] have yogurt? | | #, or 98=DK |
| CDQQ.6D | IF YES: How many times did [INSERT THE NAME IN CDQQ.0] have any lassi? | | #, or 98=DK |
| CDQQ.6Dswt | IF YES: Was it a sweet type of drink? | | 01=No, 02=Yes,98=DK |
|  | Yesterday, did [INSERT THE NAME IN CDQQ.0] eat any of the following foods: | |  |
| CDQQ.7.1 | Rice, paratha, pa ruti, semai, payesh, or packaged cereal such as Cerelac? | | 01=No, 02=Yes,98=DK |
| CDQQ.7.2 | Roti, corn, or popcorn? | | 01=No, 02=Yes,98=DK |
| CDQQ.7.3 | Potato, plantain, arum, or sweet potato? | | 01=No, 02=Yes,98=DK |
| CDQQ.7.4 | Daal, chickpeas, or khichuri? | | 01=No, 02=Yes,98=DK |
|  | Yesterday, did [INSERT THE NAME IN CDQQ.0] eat any of the following vegetables: | |  |
| CDQQ.7.5 | Carrots or pumpkin? | | 01=No, 02=Yes,98=DK |
| CDQQ.7.6.1 | Lal shak, pui shak, amaranth, spinach, or any other shak? | | 01=No, 02=Yes,98=DK |
| CDQQ.7.7.1 | Eggplant, lady finger, cauliflower, cabbage, long beans, green beans, or tomatoes? | | 01=No, 02=Yes,98=DK |
| CDQQ.7.7.2 | Bottle gourd, pointed gourd, bitter gourd, bitter melon, or ash gourd? | | 01=No, 02=Yes,98=DK |
| CDQQ.7.7.3 | White radish, kohlrabi, taro shoots, or green papaya? | | 01=No, 02=Yes,98=DK |
|  | Yesterday, did [INSERT THE NAME IN CDQQ.0] eat any of the following fruits: | |  |
| CDQQ.7.8 | Ripe mango, ripe papaya, or orange musk melon? | | 01=No, 02=Yes,98=DK |
| CDQQ.7.9 | Orange, malta, or pomelo? | | 01=No, 02=Yes,98=DK |
| CDQQ.7.10.1 | Guava, pineapple, ripe banana, watermelon, jackfruit, custard apple, or apple? | | 01=No, 02=Yes,98=DK |
| CDQQ.7.10.2 | Jamrul, star fruit, koromcha, jujube, Java plum, litchi, or amra? | | 01=No, 02=Yes,98=DK |
|  | Yesterday, did [INSERT THE NAME IN CDQQ.0] eat any of the following sweets: | |  |
| CDQQ.7.11 | Sweet biscuits, cakes, misti pitha, halwa, or jilapi? | | 01=No, 02=Yes,98=DK |
| CDQQ.7.12 | Mishti, chocolate, or ice cream? | | 01=No, 02=Yes,98=DK |
|  | Yesterday, did [INSERT THE NAME IN CDQQ.0] eat any of the following foods of animal origin: | |  |
| CDQQ.7.13 | Eggs? | | 01=No, 02=Yes,98=DK |
| CDQQ.7.14 | Paneer or cheese? | | 01=No, 02=Yes,98=DK |
| CDQQ.7org | Liver or gizzard? | | 01=No, 02=Yes,98=DK |
| CDQQ.7.16 | Sausages? | | 01=No, 02=Yes,98=DK |
| CDQQ.7.17 | Beef or goat meat? | | 01=No, 02=Yes,98=DK |
| CDQQ.7.19 | Chicken, chicken liver, pigeon, duck, or quail? | | 01=No, 02=Yes,98=DK |
| CDQQ.7.20 | Fish or dried fish? | | 01=No, 02=Yes,98=DK |
|  | Yesterday, did [INSERT THE NAME IN CDQQ.0] eat any of the following other foods: | |  |
| CDQQ.7.21 | Peanuts or jackfruit seeds? | | 01=No, 02=Yes,98=DK |
| CDQQ.7.22 | Chips or chanachur? | | 01=No, 02=Yes,98=DK |
| CDQQ.7.23 | Instant noodles such as Maggi noodles or Pran's Mr. Noodles? | | 01=No, 02=Yes,98=DK |
| CDQQ.7.24 | Puri, singara, samucha, pakora, piaju, beguni, fried chicken, or chop? | | 01=No, 02=Yes,98=DK |
| CDQQ.7R | Any other solid, semi-solid, or soft food? | | 01=No, 02=Yes,98=DK |
|  | IF YES: What was the food? | |  |
|  | Yesterday, did [INSERT THE NAME IN CDQQ.0] eat food from any place like... | |  |
| CDQQ.7.29 | KFC, CP, Pizza Hut, Helvetia, Burger King, Herfy, or other places that serve pizza or burgers? | | 01=No, 02=Yes,98=DK |
| CHECK | **Note for interviewer**/**CAPI instruction**: If not a single "yes" for foods is recorded (7-7R), ask 7S. | |  |
|  | If at least one "yes" for foods (7-7R), skip to 8. | |  |
| CDQQ.7S | Did [INSERT THE NAME IN CDQQ.0] eat any solid, semi-solid, or soft food yesterday during the day or night? | | 01=No, 02=Yes,98=DK |
| CDQQ.8 | How many times did [INSERT THE NAME IN CDQQ.0] eat any solid, semi-solid or soft foods yesterday during the day or night? If 7 or more times, record “7” | | #, or 98=DK |


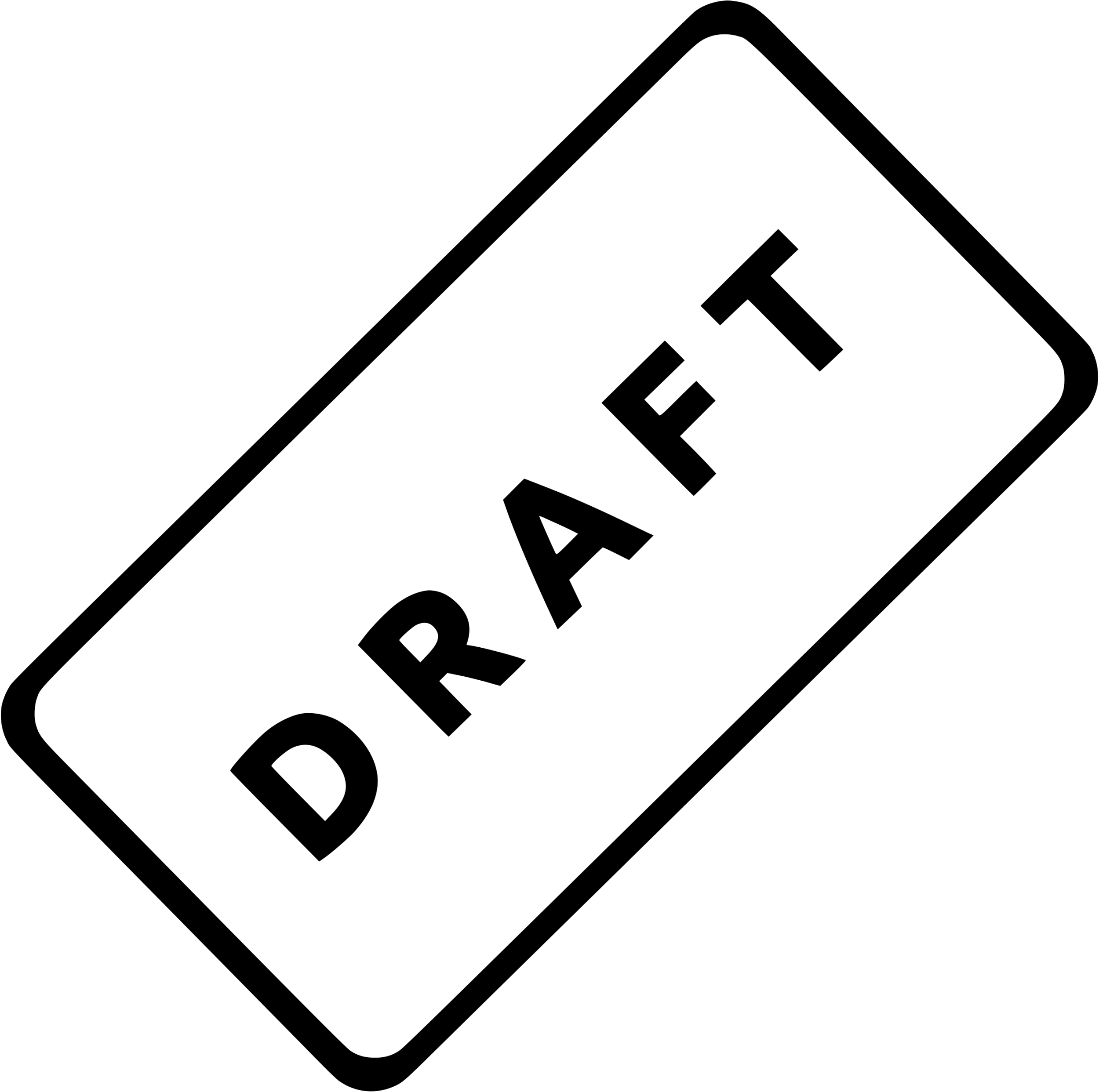


Module end time XX: XX

Module start time XX: XX

| **Early childhood (EC), Child 0-59m** | | |
| --- | --- | --- |
| **Respondent: All WRA 15-49y and adolescent with a child 0-59mand a primary caregiver of a child 0-59m whose biological mother is either not alive or does not live in the household** | | |
| CAPI instructions:   - Repeat this section for all names listed in S.N.2, S.N 3, S.N 4 and S.N 5 of the respondent matrix.   Add Respondent ID ___   - Skip if   1. BH.3=01 (Woman has never given a birth) or   2. BH.12=0 (Woman has no live birth in the last 2 years) or   3. BH.13=0 (Woman has no still birth in the last 2 years) or   4. BH.12!=. and BH.8=01 and BH.9=01 (Most recent livebirth in the last 2 years is not currently alive) or   5. BH.14!=. and BH.8=01 and BH.9=01 (Prior livebirth in the last 2 years is not currently alive) or   6. BH.15!=. and BH.8=01 and BH.9=01 or BH.10=01 (Prior livebirth in the last 5 years is not currently alive or does not live with woman) - Repeat this section for all currently living children 0-59 months listed in   1. BH.12.1, If BH.8=01 and BH.9=02 and BH.10=02   2. BH.14.1, If BH.9=02 and BH.10=02   3. BH.16.1, If BH.9=02 and BH.10=02   EC.0 Display the name of the child | | |
| Now, I would like to ask you about the various health and nutrition interventions that [INSERT FIRST NAME IN EC.0] may have received. | | |
| **Q. no** | **Q. label** | **Response** |
| EC.1 | Within the last six months, was [INSERT THE NAME IN EC.0] given a vitamin A dose like (this/any of these)? | 1. No 2. Yes 3. Don’t know |
| EC.2 | Within the last six months, was [INSERT THE NAME IN EC.0] given any tablet to treat intestinal worms? | 1. No 2. Yes 3. Don’t know |
| EC.3 | In the last six months, was [INSERT THE NAME IN EC.0] given Small Quantity-Lipid based Nutritional Supplements (SQ-LNS)? | 1. No 2. Yes 3. Don’t know |
| EC.4 | In the last 7 days, was [INSERT THE NAME IN EC.0] given an tablet or syrup or sprinkles containing iron? | 1. No 2. Yes 3. Don’t know |
| CAPI instruction: Ask EC.5 and EC.6 for child 0-23 months | | |
| EC.5 | In the last 6 months, did anyone (such as doctor, nurse, paramedics, health worker, nutrition worker) talk with you about how or what to feed [INSERT THE NAME IN EC.0] | 1. 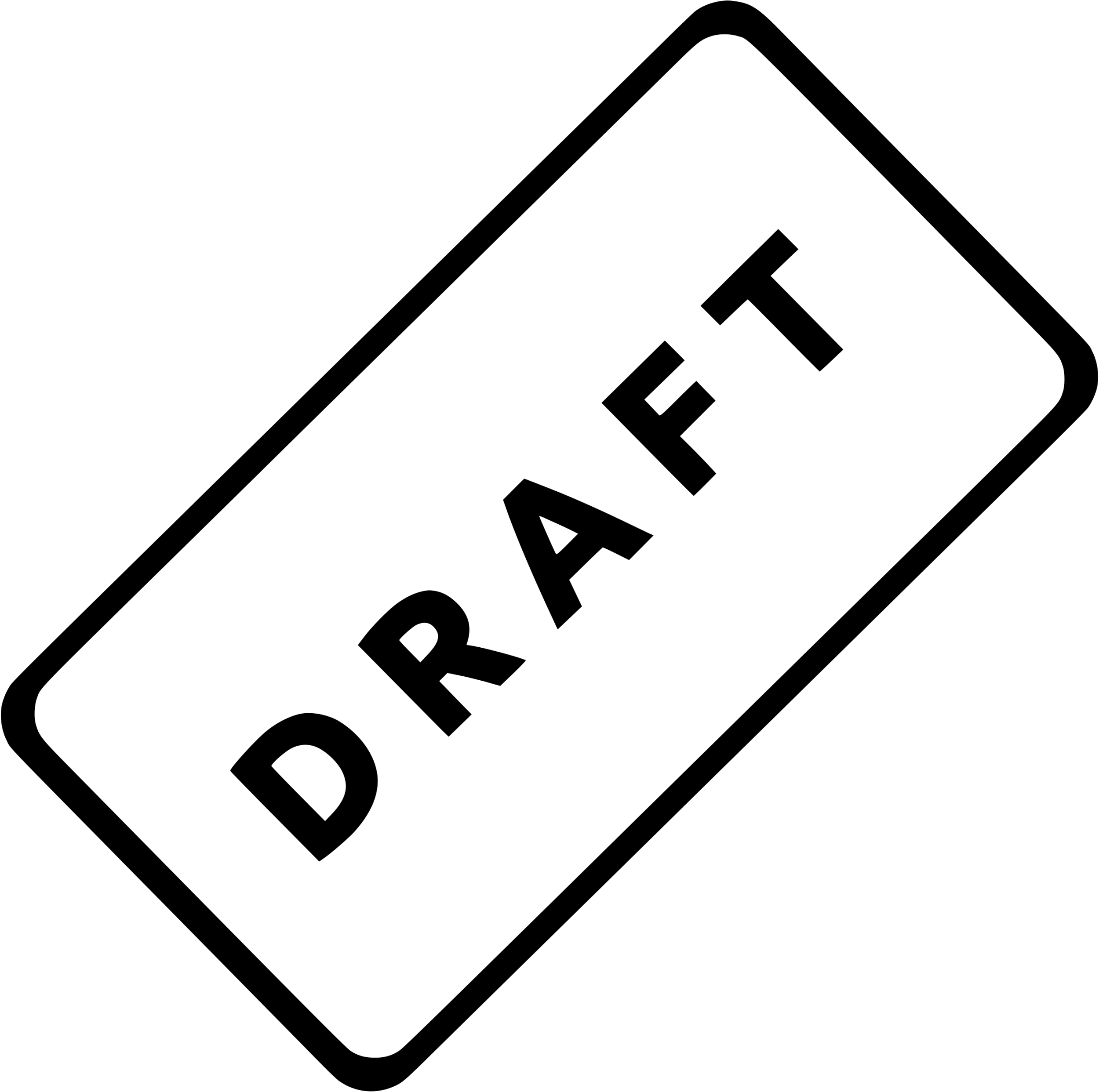No>> skip to EC.7 2. Yes 3. Don’t know>> skip to EC.7 |
| EC.6 | What did they talk with you about?  [MULTI SELECT] | 1. Breastfeeding/not feeding water or other liquids before six months 2. Introducing soft or solid food when the baby reaches six months of age 3. Giving a variety of foods 4. How often to feed foods 5. Giving animal source foods (e.g. eggs, milk, meat, fish) 6. Giving fruits, and vegetables 7. Not feeding sugar-sweetened beverages 8. Others (specify) 9. Don’t know |
| EC.7 | Has [INSERT THE NAME IN EC.0] had diarrhea in the last 2 weeks? | 1. No>> skip to EC.10 2. Yes 3. Don’t know>> skip to EC.10 |
| EC.7.1 | How many liquid or loose stools did [INSERT THE NAME IN EC.0] have over a 24 hour period, from sunrise to sunrise, on the day when they had the greatest number of loose stools? | ___ ___  (Record no. times)  (0-20 times)   - - 1. Don’t know |
| EC.7.2 | During the time when [INSERT THE NAME IN EC.0] was sick with diarrhea, how many days did your child have fever? | ___ ___  (Record no. days)  (0-20 days)   1. Don’t know |
| EC.7.3 | During the time when [INSERT THE NAME IN EC.0] was sick with diarrhea, for how many days did they experience vomiting? | ___ ___  (Record no. days)  (0-20 days)   1. Don’t know |
| EC.8 | Was [INSERT THE NAME IN EC.0] given ORS at any time since [INSERT THE NAME IN EC.0] started having the diarrhea? | 1. No 2. Yes 3. Don’t know |
| EC.9 | Was [INSERT NAME IN EC.0] given Zinc tablet at any time since [INSERT THE NAME IN EC.0] started having the diarrhea? | 1. 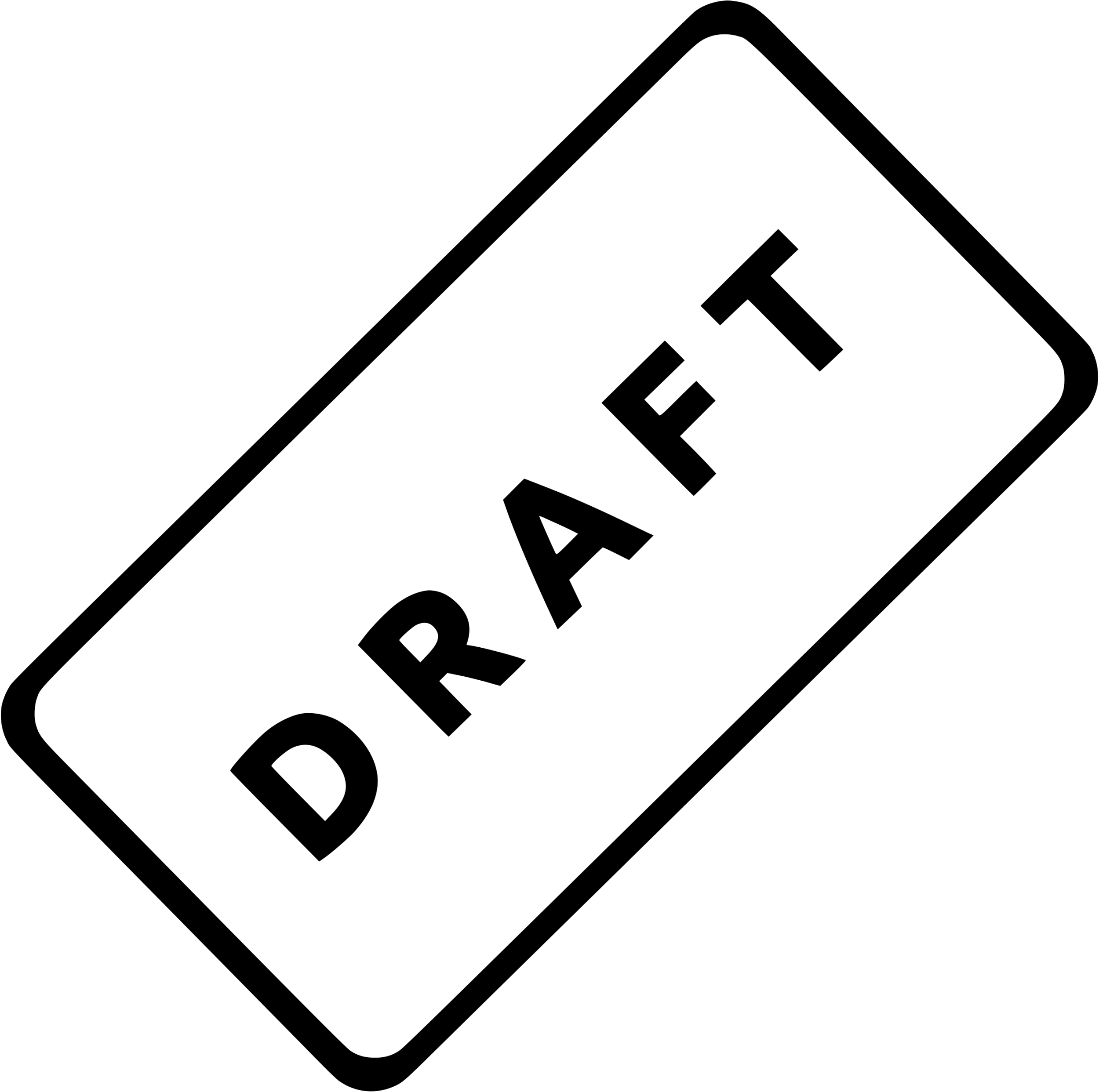No 2. Yes 3. Don’t know |
| EC.10 | In the last 3 months, has [INSERT THE NAME IN EC.0]'s weight/height or Mid Upper Arm Circumference (MUAC) been measured by anyone such as doctor, nurse, paramedics, health worker, nutrition worker? |  |
| EC.10.1 | Weight | 1. No 2. Yes 3. Don’t know |
| EC.10.2 | Height | 1. No 2. Yes 3. Don’t know |
| EC.10.3 | MUAC | 1. No 2. Yes 3. Don’t know |
| Only ask EC.11-15 if answer to either of EC.10.1 and EC.10.2 and EC.10.3 is ‘02’=Yes | | |
| EC.11 | After any of the times [INSERT THE NAME IN EC.0] was measured, did anyone such as doctor, nurse, paramedics, health worker, nutrition worker talk with you about how [INSERT THE NAME IN EC.0] is growing? | 1. No 2. Yes 3. Don’t know |
| EC.12 | After any of the times [INSERT THE NAME IN EC.0] was measured, did anyone such as doctor, nurse, paramedics, health worker, nutrition worker tell you that [INSERT THE NAME IN EC.0] was undernourished? | 1. No>> skip to ODQQ-7 2. Yes 3. Don’t know>> skip to ODQQ-7 |
| EC.13 | After [INSERT THE NAME IN EC.0] was identified as undernourished , did you receive any advice on how to feed your child by anyone such as doctor, nurse, health worker, nutrition worker? | 1. No 2. Yes 3. Don’t know |
| EC.14 | After [INSERT THE NAME IN EC.0] was identified as undernourished in the last 3 months, did you receive any information on to prepare kichuri or halwa for him/her? | 1. No 2. Yes 3. Don’t know |
| EC.15 | After [INSERT THE NAME IN EC.0] was identified as undernourished in the last 3 months, did [INSERT THE NAME IN EC.0] receive any other treatment in any health facility or a rehabilitation center? | 1. No 2. Yes 3. Don’t know |

Module end time XX: XX

Module start time XX: XX

| **Diet Quality Questionnaire (DQQ), Older child 2-9 years** | | |
| --- | --- | --- |
| **Respondent: All WRA 15-49y and adolescent with a child 2-9 years old, and a primary caregiver of a child 2-9 years old whose biological mother is either not alive or does not live in the household.** | | |
| CAPI instructions:   - Repeat this section for all names listed in S.N.2, S.N 3, S.N 5 and S.N 6 (2-9 years) of the respondent matrix.   Add Respondent ID ___   - 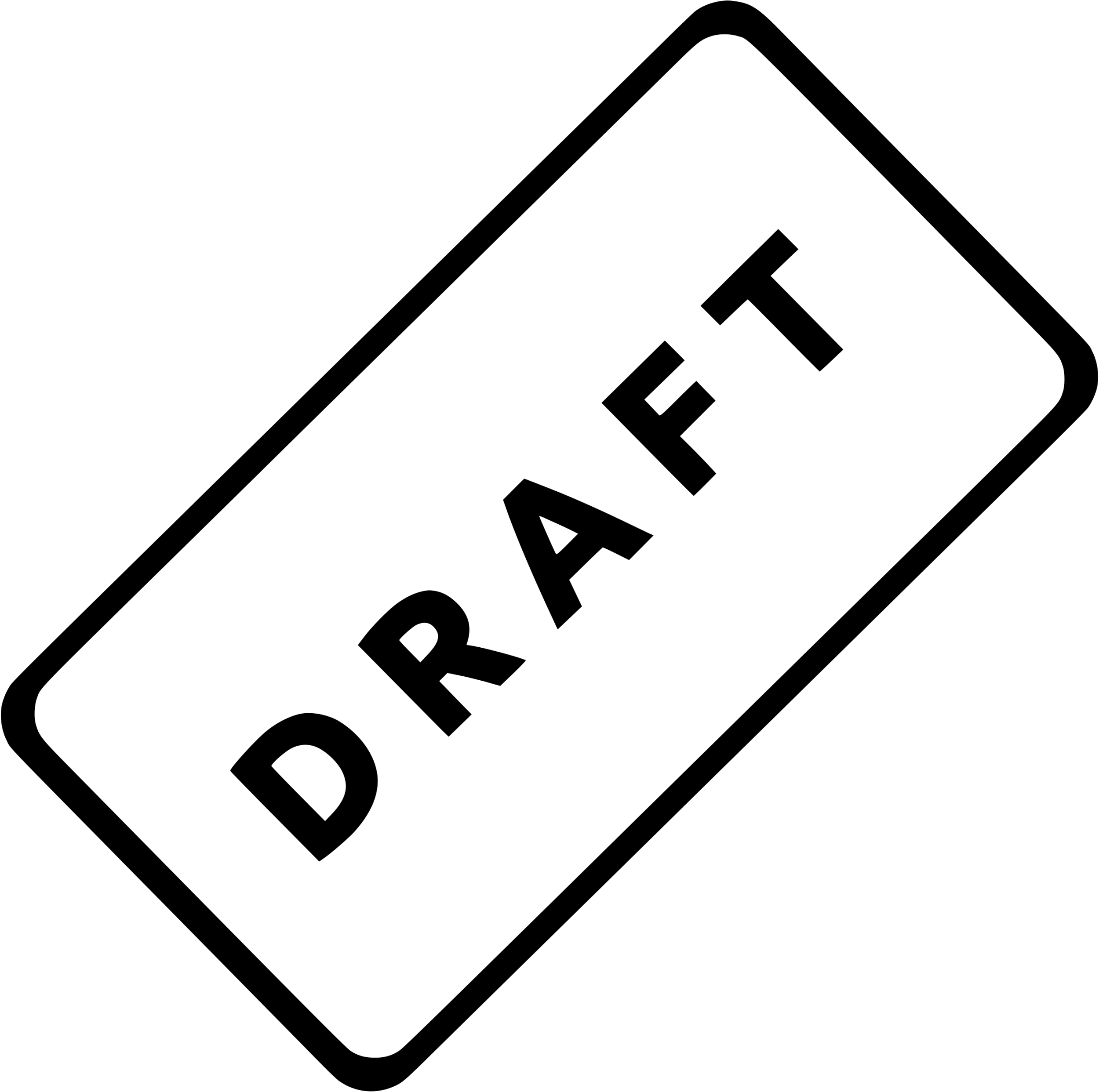Skip if   1. BH.2=01 (Woman has not given birth) or   2. BH.16!=. and BH.9=01 or BH.10=01 (Prior livebirth is not currently alive or does not live with woman)   3. BH.17!=. and BH.9=01 or BH.10=01 (Prior livebirth is not currently alive or does not live with woman) - Repeat for all currently living children 2-9 years. i.e all name listed in BH.16.1 and BH.17.1 (and if BH.9=02 and BH.10=02)   ODQQ.0 Display the name of the child | | |
| ODQQ.7 | Now I would like to ask you about foods that [INSERT THE NAME IN CDQQ.0] had yesterday during the day or at night. I am interested in foods your child ate whether at home or somewhere else. Please think about snacks and small meals as well as main meals.  I will ask you about different types of foods, and I would like to know whether your child ate the food even if it was combined with other foods. Please do not answer ‘yes’ for any food or ingredient used in a small amount to add flavour to a dish.  Yesterday during the day or at night, did [INSERT THE NAME IN CDQQ.0] eat: | |
| ODQQ.7.15 | Yogurt or lassi? | #, or 98=DK |
| ODQQ.7.15num | IF YES: How many times did [INSERT THE NAME IN CDQQ.0] have yogurt? | #, or 98=DK |
| ODQQ.6D | IF YES: How many times did [INSERT THE NAME IN CDQQ.0] have any lassi? | #, or 98=DK |
| ODQQ.6Dswt | IF YES: Was it a sweet type of drink? | 01=No, 02=Yes,98=DK |
|  | Yesterday, did [INSERT THE NAME IN CDQQ.0] eat any of the following foods: |  |
| ODQQ.7.1 | Rice, paratha, pa ruti, semai, payesh, or packaged cereal such as Cerelac? | 01=No, 02=Yes,98=DK |
| ODQQ.7.2 | Roti, corn, or popcorn? | 01=No, 02=Yes,98=DK |
| ODQQ.7.3 | Potato, plantain, arum, or sweet potato? | 01=No, 02=Yes,98=DK |
| ODQQ.7.4 | Daal, chickpeas, or khichuri? | 01=No, 02=Yes,98=DK |
|  | Yesterday, did [INSERT THE NAME IN CDQQ.0] eat any of the following vegetables: |  |
| ODQQ.7.5 | Carrots or pumpkin? | 01=No, 02=Yes,98=DK |
| ODQQ.7.6.1 | Lal shak, pui shak, amaranth, spinach, or any other shak? | 01=No, 02=Yes,98=DK |
| ODQQ.7.7.1 | Eggplant, lady finger, cauliflower, cabbage, long beans, green beans, or tomatoes? | 01=No, 02=Yes,98=DK |
| ODQQ.7.7.2 | Bottle gourd, pointed gourd, bitter gourd, bitter melon, or ash gourd? | 01=No, 02=Yes,98=DK |
| ODQQ.7.7.3 | White radish, kohlrabi, taro shoots, or green papaya? | 01=No, 02=Yes,98=DK |
|  | Yesterday, did [INSERT THE NAME IN CDQQ.0] eat any of the following fruits: |  |
| ODQQ.7.8 | Ripe mango, ripe papaya, or orange musk melon? | 01=No, 02=Yes,98=DK |
| ODQQ.7.9 | Orange, malta, or pomelo? | 01=No, 02=Yes,98=DK |
| ODQQ.7.10.1 | Guava, pineapple, ripe banana, watermelon, jackfruit, custard apple, or apple? | 01=No, 02=Yes,98=DK |
| ODQQ.7.10.2 | Jamrul, star fruit, koromcha, jujube, Java plum, litchi, or amra? | 01=No, 02=Yes,98=DK |
|  | Yesterday, did [INSERT THE NAME IN CDQQ.0] eat any of the following sweets: |  |
| ODQQ.7.11 | Sweet biscuits, cakes, misti pitha, halwa, or jilapi? | 01=No, 02=Yes,98=DK |
| ODQQ.7.12 | Mishti, chocolate, or ice cream? | 01=No, 02=Yes,98=DK |
|  | Yesterday, did [INSERT THE NAME IN CDQQ.0] eat any of the following foods of animal origin: |  |
| ODQQ.7.13 | Eggs? | 01=No, 02=Yes,98=DK |
| ODQQ.7.14 | Paneer or cheese? | 01=No, 02=Yes,98=DK |
| ODQQ.7org | Liver or gizzard? | 01=No, 02=Yes,98=DK |
| ODQQ.7.16 | Sausages? | 01=No, 02=Yes,98=DK |
| ODQQ.7.17 | Beef or goat meat? | 01=No, 02=Yes,98=DK |
| ODQQ.7.19 | Chicken, chicken liver, pigeon, duck, or quail? | 01=No, 02=Yes,98=DK |
| ODQQ.7.20 | Fish or dried fish? | 01=No, 02=Yes,98=DK |
|  | Yesterday, did [INSERT THE NAME IN CDQQ.0] eat any of the following other foods: |  |
| ODQQ.7.21 | Peanuts or jackfruit seeds? | 01=No, 02=Yes,98=DK |
| ODQQ.7.22 | Chips or chanachur? | 01=No, 02=Yes,98=DK |
| ODQQ.7.23 | Instant noodles such as Maggi noodles or Pran's Mr. Noodles? | 01=No, 02=Yes,98=DK |
| ODQQ.7.24 | Puri, singara, samucha, pakora, piaju, beguni, fried chicken, or chop? | 01=No, 02=Yes,98=DK |
| ODQQ.7R | Any other solid, semi-solid, or soft food? | 01=No, 02=Yes,98=DK |
|  | IF YES: What was the food? |  |
|  | Yesterday, did [INSERT THE NAME IN CDQQ.0] eat food from any place like... |  |
| 7ODQQ.29 | KFC, CP, Pizza Hut, Helvetia, Burger King, Herfy, or other places that serve pizza or burgers? | 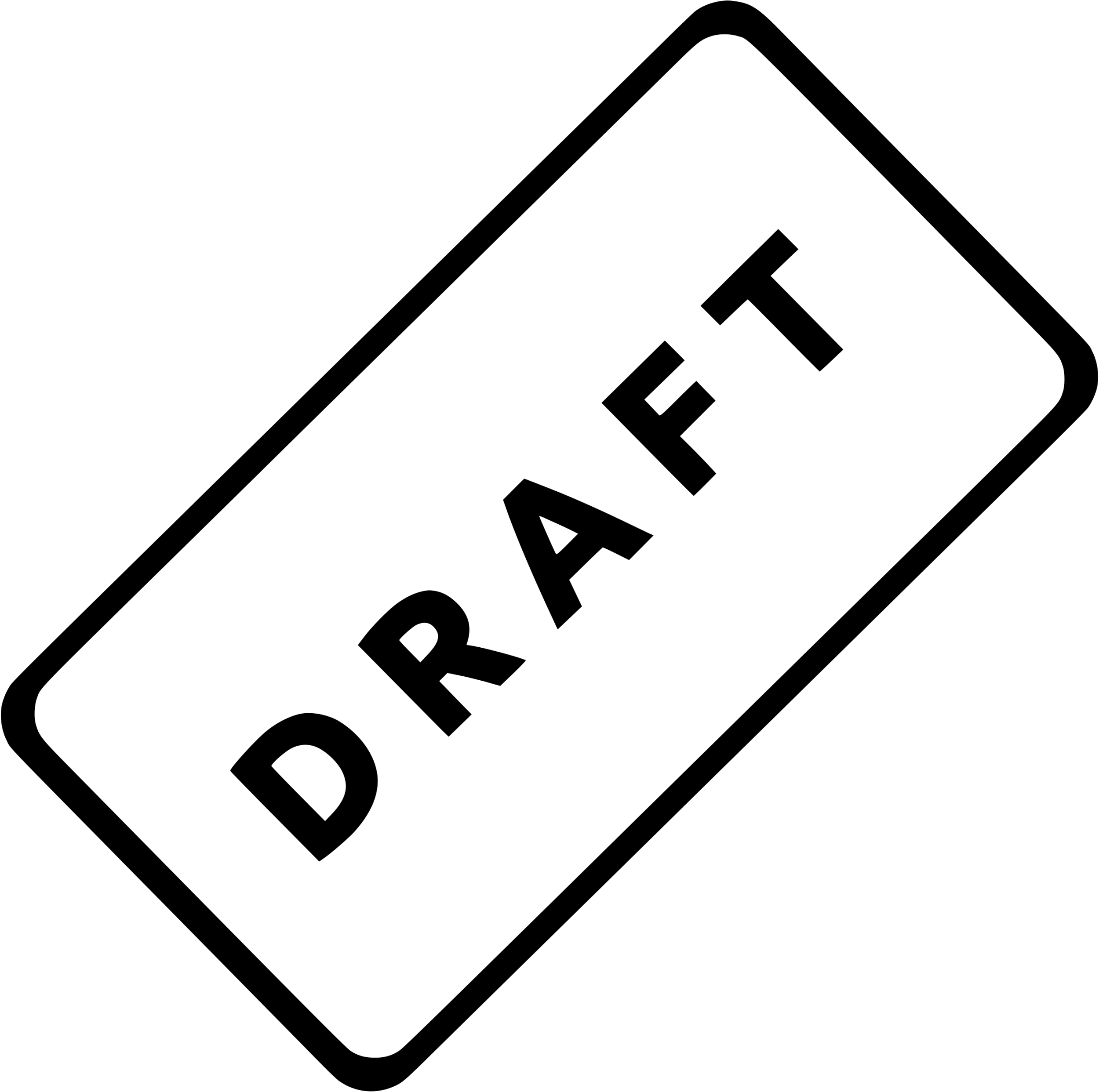01=No, 02=Yes,98=DK |
| CHECK | **Note for interviewer**/**CAPI instruction**: If not a single "yes" for foods is recorded (7-7R), ask 7S. |  |
|  | If at least one "yes" for foods (7-7R), skip to 8. |  |
| ODQQ.7S | Did [INSERT THE NAME IN CDQQ.0] eat any solid, semi-solid, or soft food yesterday during the day or night? | 01=No, 02=Yes,98=DK |
| ODQQ.8 | How many times did [INSERT THE NAME IN CDQQ.0] eat any solid, semi-solid or soft foods yesterday during the day or night? If 7 or more times, record “7” | #, or 98=DK |

Module end time XX: XX

Module start time XX: XX

| **School aged children (SC), Child 5-9y** | | |
| --- | --- | --- |
| **Respondent: All WRA 15-49y and adolescent with a child 5-9y and a primary caregiver of a child 5-9y whose biological mother is either not alive or does not live in the household** | | |
| 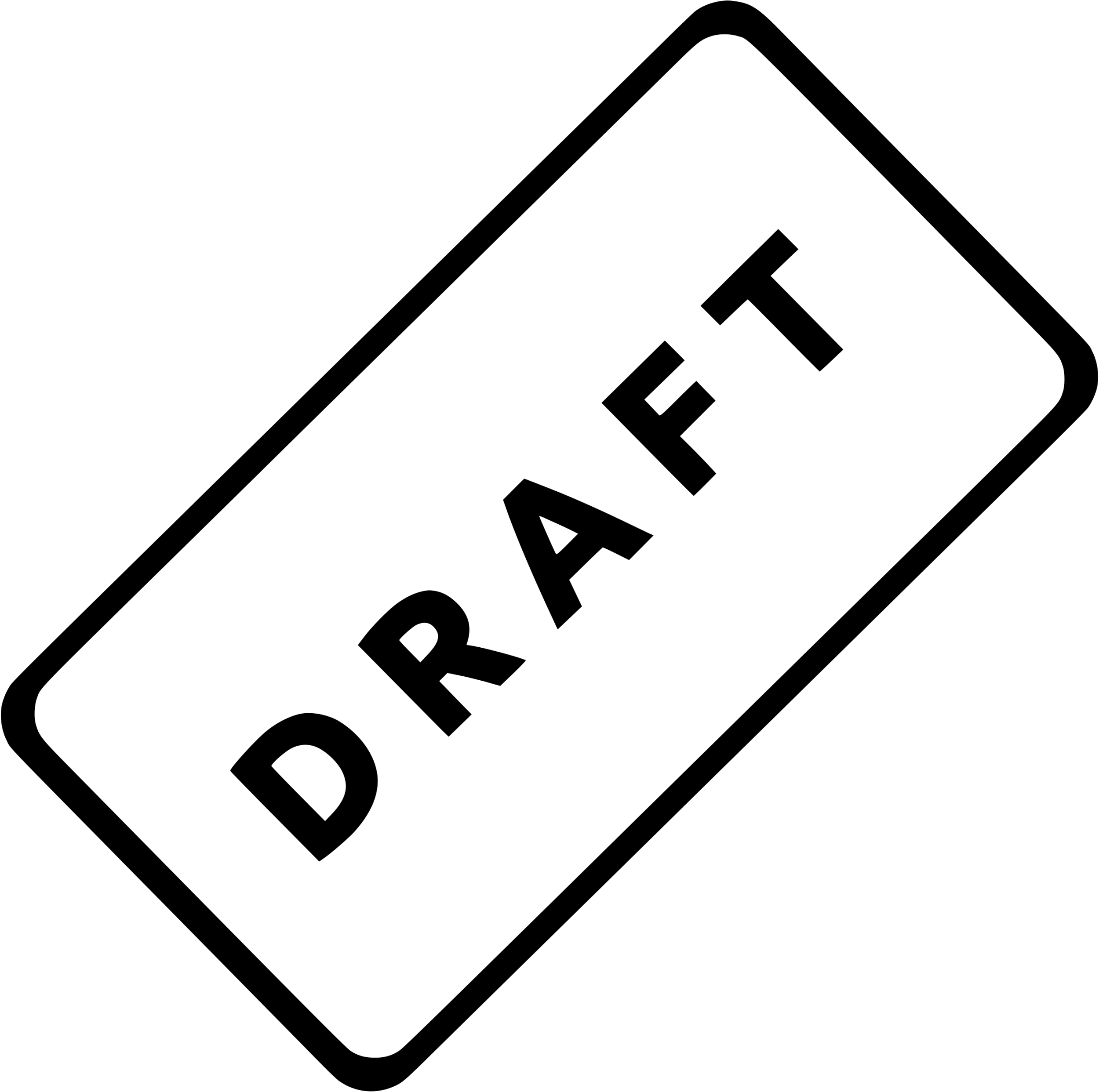CAPI instructions-   - Repeat this section for all names listed in S.N.2, S.N 3 and S.N 6 of the respondent matrix.   Add Respondent ID ___   - Skip if   1. BH.2=01 (Woman has never given a birth) or   2. BH.17!=. and BH.9=01 or BH.10=01 (Prior livebirth in the last 5 years is not currently alive or does not live with woman) - Repeat this section for all live births (in the last 5-9 years) of this woman (i.e for all name listed in BH.17 if BH.9=02 and BH.10=02)   SC.0 Display the name of the child | | |
| Now, I would like to ask you about various health and nutrition interventions that [INSERT THE NAME IN SC.0] may have received. | | |
| SC.1 | In the last 7 days, was [INSERT THE NAME IN SC.0] given an a tablet or syrup or sprinkles containing iron? | 1. No 2. Yes 3. Don’t know |
| SC.2 | In the last six months, was [INSERT THE NAME IN SC.0] given any tablet to treat intestinal worms? | 1. No 2. Yes 3. Don’t know |
| SC.3 | Has anyone (such as health worker or nutrition worker) talked with you about feeding [INSERT THE NAME IN SC.0] five different food groups daily or a diverse diet? | 1. No 2. Yes   98. Don’t know |
| SC.4 | Have you ever heard or seen information about feeding [INSERT THE NAME IN SC.0] five different food groups daily or a diverse diet? | 1. No 2. Yes   98. Don’t know |
| SC.5 | Have you ever heard or seen information about avoiding certain foods such as soft drinks, energy drinks or sweets, biscuits, chips, namki, bhujia for [INSERT THE NAME IN SC.0]? | 1. No 2. Yes   98. Don’t know |


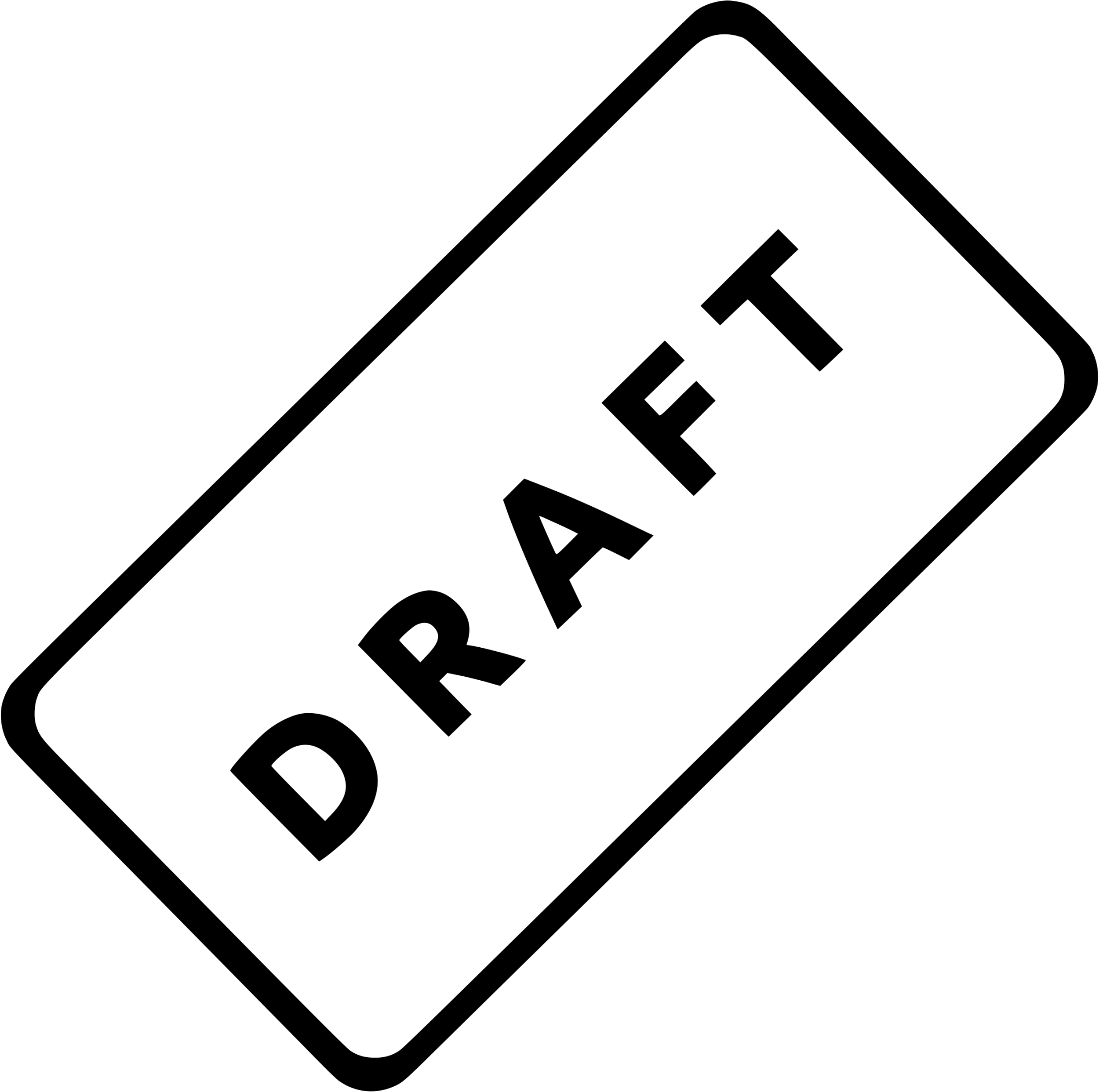
Module start time XX: XX

| **Interviewee tion (IR)** | |
| --- | --- |
| IR.1 | How difficult was it to answer questions in this survey? |
| 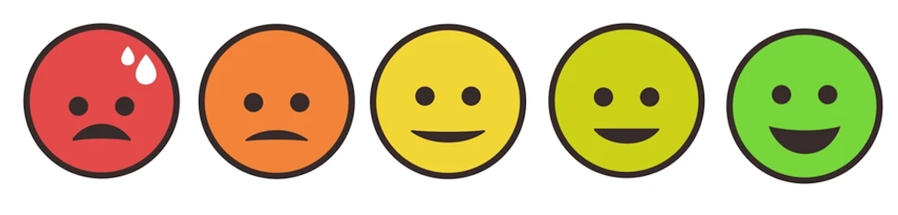    Very Hard Hard Average Easy Very easy | |
| IR.2 | How tiring was it to participate in this survey? |
| 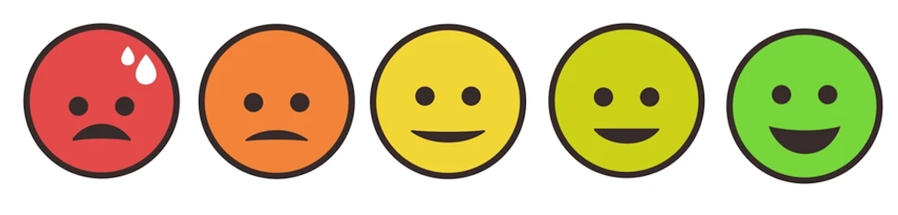  Very Hard Hard Average Easy Very easy | |

Module end time XX: XX

# **ADOLESCENT QUESTIONNAIRE**


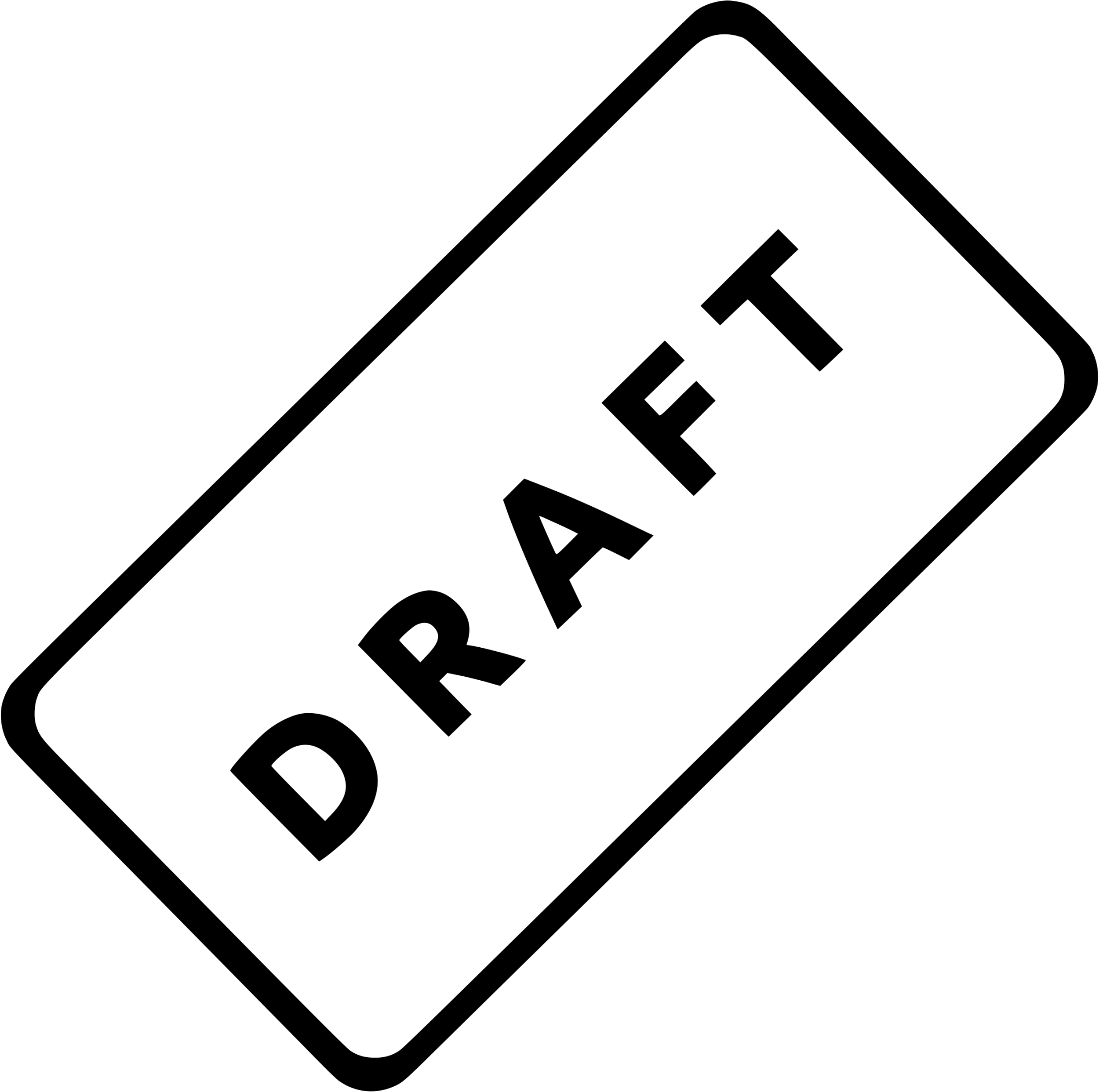
CAPI Instruction: Include flags for difficult questions that are populated in Module IR

| **ADOLESCENT CONSENT(AC)**  *Administer consent form* | | |
| --- | --- | --- |
| **Respondent: All adolescents (all gender), 10-19 years of age** | | |
| INSTRUCTIONS: ASK TO SPEAK TO THE PARENT OF THE ADOLESCENT 10-19 YEARS OF AGE USING CONSENT FORM TO CONFIRM PARENTAL CONSENT. NEXT, ASK TO SPEAK THE ADOLESCENT 10-19 YEARS OF AGE IF PARENTAL CONSENT IS PROVIDED TO COMPLETE ASSENT PROCESS AND START INTERVIEW. | | |
| CAPI instruction: Repeat this section for all names listed in S.N.4. Add Respondent ID __ | | |
| **Adolescent Questionnaire Consent** | | |
| AC.1 | Do you give permission for your child to participate in this study? | 1. No >> skip to end of survey 2. Yes |
| AC.2 | Do you agree to participate in the study? | 1. No >> skip to end of survey   Yes |
| AC. 3 | Respondent Name | _____________  [Name of Respondent]  auto-filled |

Module start time XX: XX

| **Adolescent interventions (AD), 10-19y** |
| --- |
| **Respondent: All adolescents (all gender), 10-19 years of age** |
| CAPI instruction:   - Repeat this section for all names listed in S.N.4. Add Respondent ID __ |
| I would like to ask you about various health and nutrition interventions that you as an adolescent may have received. |

| **Q. no** | **Q. label** | **Response** |
| --- | --- | --- |
| AD.1 | In the last three months, were you given, or did you buy any tablet or syrup that contains iron?  INSTRUCTION: SHOW VISUAL AID OF MMS TABLET & FULLCARE, COMMON TYPES OF MULTIPLE MICRONUTRIENT SUPPLEMENTS, & COMMON TYPES OF IRON/IFA | 1. No>>skip to AD.4 2. Yes 3. Don’t know>>skip to AD.4 |
| AD.1.1 | In the last week, were you given, or did you buy any tablet or syrup that contains iron? | 1. No 2. Yes 3. Don’t know |
| AD.2 | In the last three months, were you given or did you buy any of the following? :   1. MMS TABLET OR FULLCARE?   INSTRUCTION: SHOW VISUAL AID OF MMS TABLET & FULLCARE   1. SUPPLEMENTS WITH MULTIPLE MICRONUTRIENTS?   INSTRUCTION: SHOW VISUAL AID OF COMMON TYPES OF MULTIPLE MICRONUTRIENT SUPPLEMENTS.  READ ALOUD: PLEASE THINK ABOUT THESE AND SIMILAR PRODUCTS; THE PICTURES ARE JUST EXAMPLES OF SUPPLEMENTS CONTAINING MMS   1. IRON TABLET OR SYRUPS?   INSTRUCTION: SHOW VISUAL AID OF COMMON TYPES OF IRON/IFA  READ ALOUD: PLEASE THINK ABOUT THESE AND SIMILAR PRODUCTS; THE PICTURES ARE JUST EXAMPLESOF TABLETS CONTAINING IRON/IFA  [PROBE: ANYTHING ELSE?]  [MULTI SELECT] | 1. 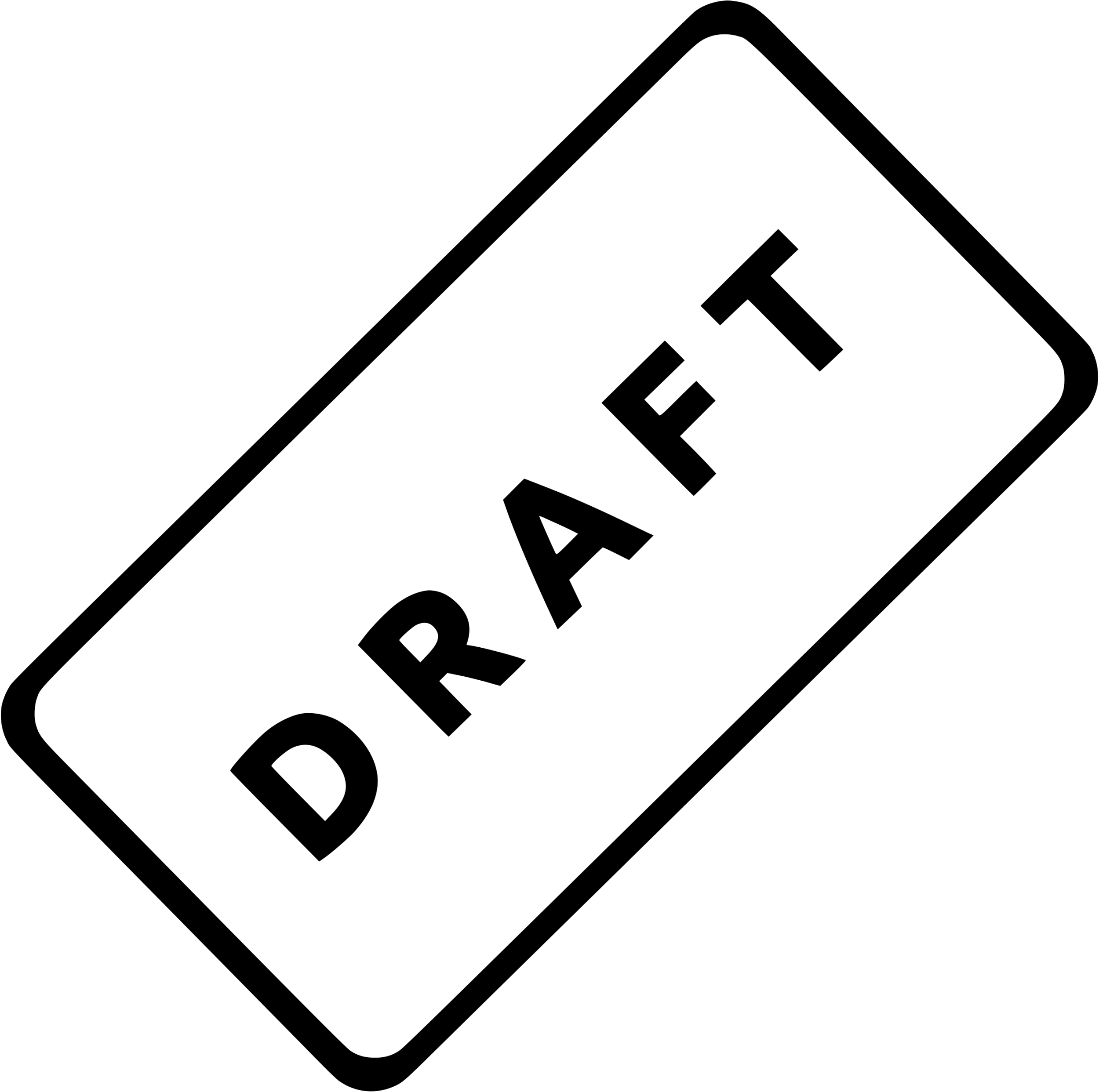MMS tablet, FullCare, 2. Supplements with multiple micronutrients 3. Iron tablet/Iron folic acid 4. Other (specify) 5. Don’t know |
| AD.3 | How many days did you take [INSERT TYPE FROM AD.2] in the last **month**?  INSTRUCTION: SHOW VISUAL AID  CAPI Instruction: Repeat this question for all the chosen responses in AD.2 | ___ ___  (Record no. tablets)  (0-90 tablets)   1. No. tablets |
| AD.4 | Did you receive tablets to treat intestinal worms in the last 6 months from school or health facility)? | 1. No 2. Yes   98. Don’t know |
| AD.5 | Have you received food supplements in the last one month from school or health centers or any other programs? | 1. No 2. Yes   98. Don’t know |
| AD.6 | Have you ever heard or seen information about eating five different food groups or eating a diverse diet? | 1. No 2. Yes   98. Don’t know |
| AD.7 | Have you ever heard or seen information about avoiding certain foods such as soft drinks, energy drinks or sweets, biscuits, chips, namki, bhujia? | 1. 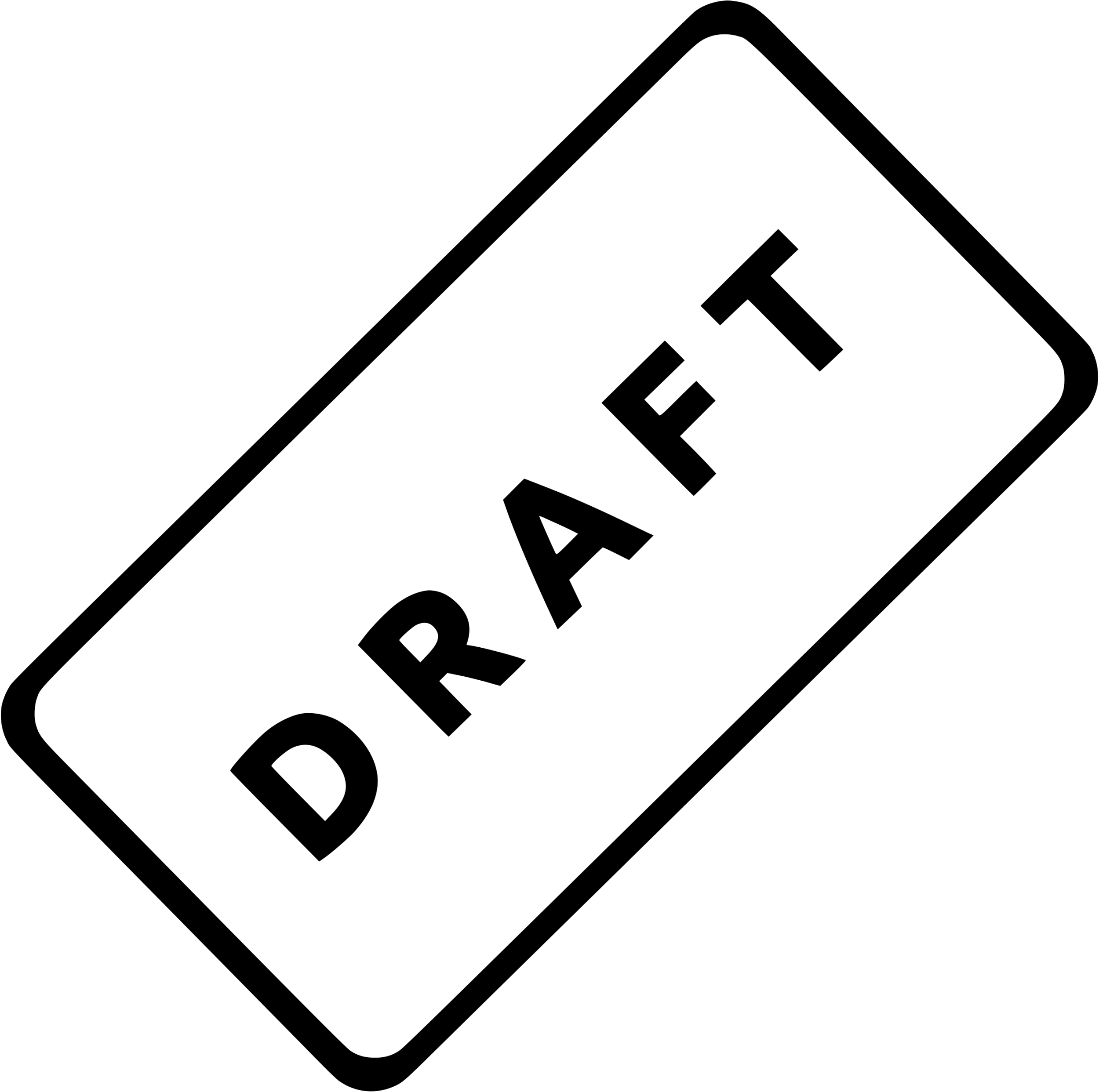No 2. Yes   98. Don’t know |
| Now, I would like to ask you about any meals that you receive at school. | | |
| AD.8 | In the past 12 months did you receive free food from a school feeding program, whether distributed at school or elsewhere? | 1. No>>skip to IR.1 2. Yes   98. Don’t know>>skip to IR.1 |
| AD.9 | Was the free food you receive fortified or food with extra nutrients? | 1. No 2. Yes   98. Don’t know |
| AD.10 | In the past 12 months, with the free food that you received, did you also receive: ______________ | 1. No 2. Yes   98. Don’t know |
| AD.10.1 | Nutrition or health counseling? | 1. No 2. Yes   98. Don’t know |
| AD.10.2 | Told to go to a health facility to receive health or nutrition services? | 1. No 2. Yes 3. Don’t know |
| AD.10.3 | Tablets to treat intestinal worms? | 1. No 2. Yes 3. Don’t know |
| AD.10.4 | Iron tablets or other nutrient supplements? | 1. No 2. Yes 3. Don’t know |
| AD.10.5 | Food with extra nutrients added to it to benefit health | 1. No 2. Yes 3. Don’t know |
| AD.10.6 | Other (Specify) | 1. No 2. Yes 3. Don’t know |

Module end time XX: XX

Module start time XX: XX

| **Diet Quality Questionnaire (DQQ)-Adolescent** | | |
| --- | --- | --- |
| **Respondent: Unmarried adolescent girls 10-14y in the HH adolescent boys 11-19y** | | |
| CAPI instruction:   - Repeat this section for all names listed in S.N 3 (unmarried adolescent) of the respondent matrix. - Add Respondent ID __ | | |
| 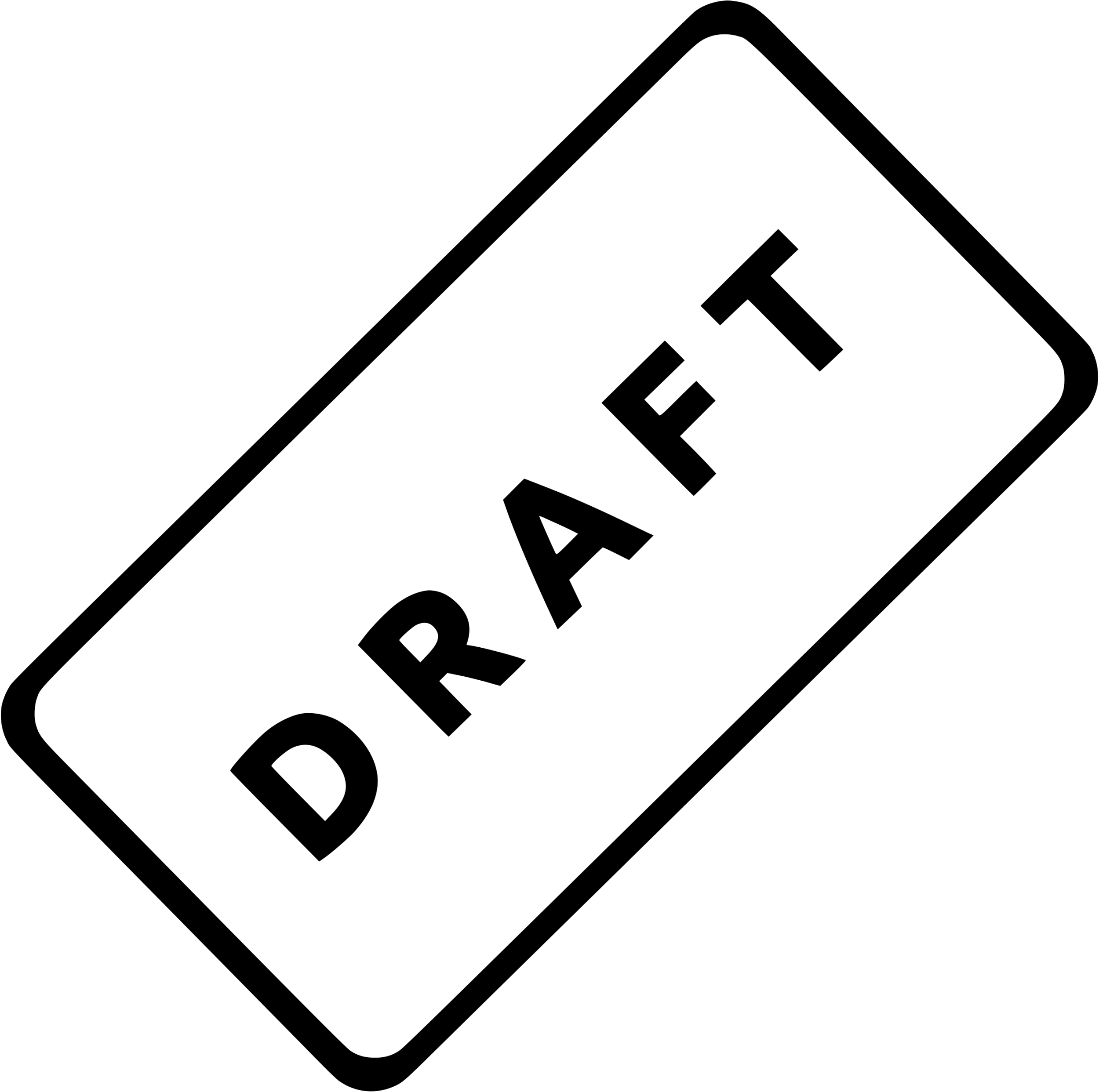Now I’d like to ask you some yes-or-no questions about foods and drinks that you consumed yesterday during the day or night, whether you had it at home or somewhere else. First, I would like you to think about yesterday, from the time you woke up through the night. Think to yourself about the first thing you ate or drank after you woke up in the morning … Think about where you were when you had any food or drink in the middle of the day … Think about where you were when you had any evening meal … and any food or drink you may have had in the evening or late-night... and any other snacks or drinks you may have had between meals throughout the day or night.  I am interested in whether you had the food items I will mention even if they were combined with other foods.  Please listen to the list of foods and drinks, and if you ate or drank ANY ONE OF THEM, say yes. | | |
|  | Yesterday, did you eat any of the following foods: |  |
| ADQQ.1 | Rice, paratha, or pa ruti? | 01=No, 02=Yes |
| ADQQ.2 | Roti, corn, or popcorn? | 01=No, 02=Yes |
| ADQQ.3 | Potato, plantain, arum, or sweet potato? | 01=No, 02=Yes |
| ADQQ.4 | Daal, chickpeas, or khichuri? | 01=No, 02=Yes |
|  | Yesterday, did you eat any of the following vegetables: |  |
| ADQQ.5 | Carrots or pumpkin? | 01=No, 02=Yes |
| ADQQ.6.1 | Lal shak, pui shak, amaranth, spinach, or any other shak? | 01=No, 02=Yes |
| ADQQ.7.1 | Eggplant, lady finger, cauliflower, cabbage, long beans, green beans, or tomatoes? | 01=No, 02=Yes |
| ADQQ.7.2 | Bottle gourd, pointed gourd, bitter gourd, bitter melon, or ash gourd? | 01=No, 02=Yes |
| ADQQ.7.3 | White radish, kohlrabi, taro shoots, or green papaya? | 01=No, 02=Yes |
|  | Yesterday, did you eat any of the following fruits: |  |
| ADQQ.8 | Ripe mango, ripe papaya, or orange musk melon? | 01=No, 02=Yes |
| ADQQ.9 | Orange, malta, or pomelo? | 01=No, 02=Yes |
| ADQQ.10.1 | Guava, pineapple, ripe banana, watermelon, jackfruit, custard apple, or apple? | 01=No, 02=Yes |
| ADQQ.10.2 | Jamrul, star fruit, koromcha, jujube, Java plum, litchi, or amra? | 01=No, 02=Yes |
|  | Yesterday, did you eat any of the following sweets: |  |
| ADQQ.11 | Sweet biscuits, cakes, misti pitha, halwa, or jilapi? | 01=No, 02=Yes |
| ADQQ.12 | Mishti, chocolate, or ice cream? | 01=No, 02=Yes |
|  | Yesterday, did you eat any of the following foods of animal origin: |  |
| ADQQ.13 | Eggs? | 01=No, 02=Yes |
| ADQQ.14 | Paneer or cheese? | 01=No, 02=Yes |
| ADQQ.15 | Yogurt or lassi? | 01=No, 02=Yes |
| ADQQ.16 | Sausages? | 01=No, 02=Yes |
| ADQQ.17 | Beef or goat meat? | 01=No, 02=Yes |
| ~~ADQQ.18~~ | ~~N/A~~ | ~~01=No, 02=Yes~~ |
| ADQQ.19 | Chicken, chicken liver, pigeon, duck, or quail? | 01=No, 02=Yes |
| ADQQ.20 | Fish or dried fish? | 01=No, 02=Yes |
|  | Yesterday, did you eat any of the following other foods: |  |
| ADQQ.21 | Peanuts or jackfruit seeds? | 01=No, 02=Yes |
| ADQQ.22 | Chips or chanachur? | 01=No, 02=Yes |
| ADQQ.23 | Instant noodles such as Maggi noodles or Pran's Mr. Noodles? | 01=No, 02=Yes |
| ADQQ.24 | Puri, singara, samucha, pakora, piaju, beguni, fried chicken, or chop? | 01=No, 02=Yes |
|  | Yesterday, did you have any of the following beverages: |  |
| ADQQ.25 | Milk? | 01=No, 02=Yes |
| ADQQ.26 | Tea with sugar, coffee with sugar, chocolate milk, Horlicks, Milo, Complan or Ovaltine? | 01=No, 02=Yes |
| ADQQ.27 | Fruit juice, packet juice such as Frooto or Tang, or shorbot? | 01=No, 02=Yes |
| ADQQ.28 | Soft drinks such as Pepsi, Mojo, Sprite, or Fanta, or energy drinks such as Tiger? | 01=No, 02=Yes |
|  | Yesterday, did you get food from any place like... |  |
| ADQQ.29 | KFC, CP, Pizza Hut, Helvetia, Burger King, Herfy, or other places that serve pizza or burgers? | 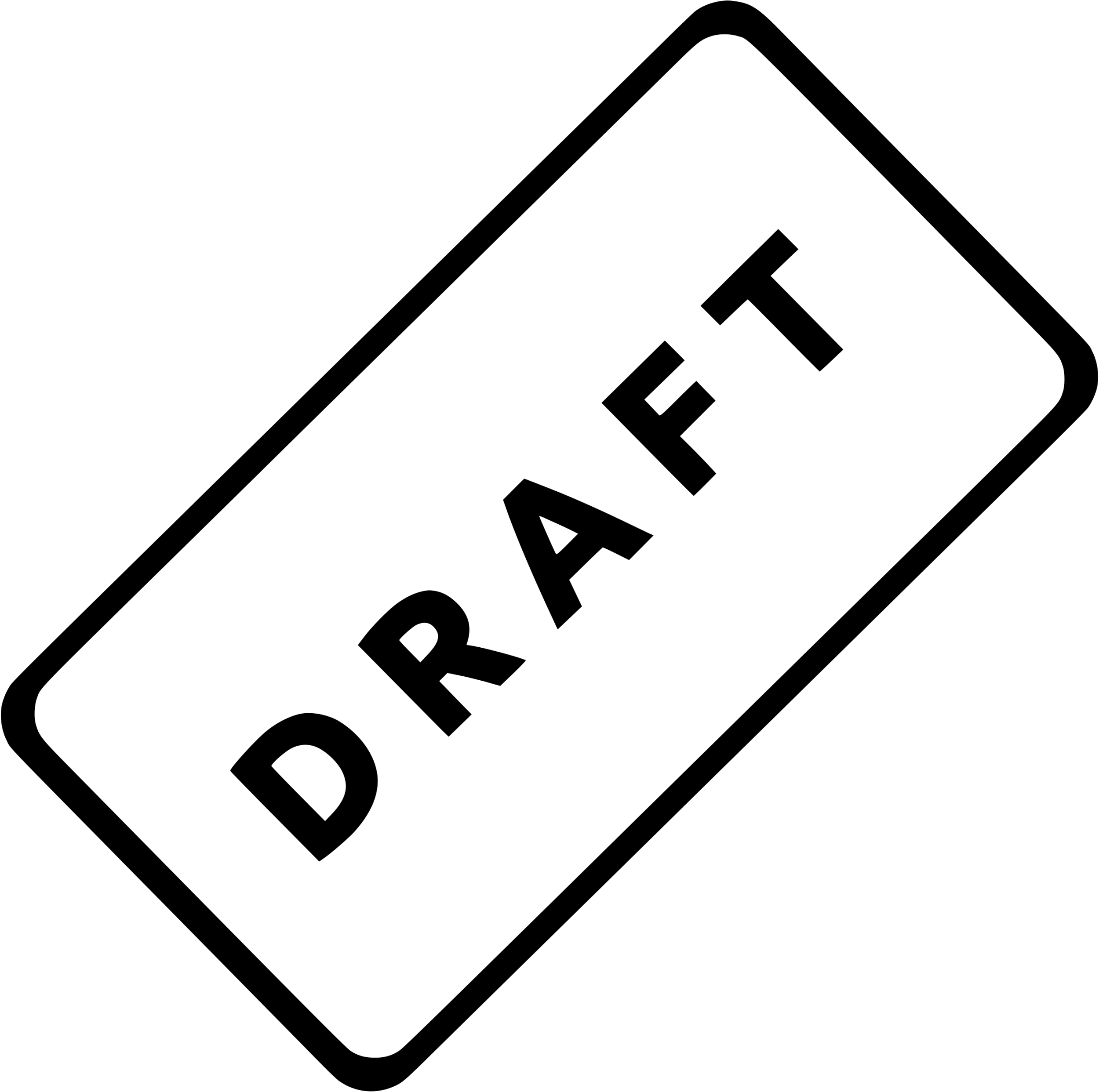01=No, 02=Yes |

Module end time XX: XX

Module start time XX: XX

| **Interviewee reflection (IR)** | |
| --- | --- |
| IR.1 | How difficult was it to answer questions in this survey? |
| 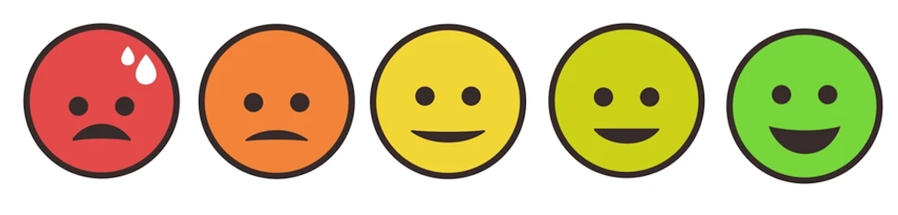    Very Hard Hard Average Easy Very easy | |
| IR.2 | How tiring was it to participate in this survey? |
| 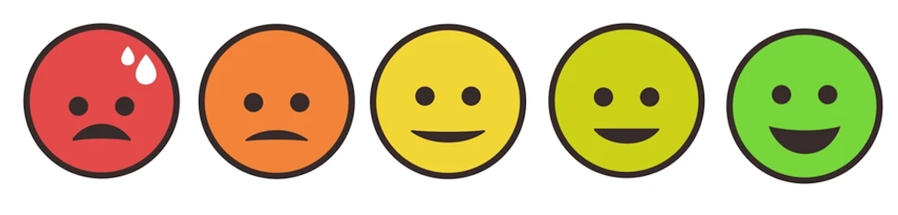  Very Hard Hard Average Easy Very easy | |

Module end time XX: XX
