# Supplementary material for: Methodological considerations and cost to measure coverage of multisectoral nutrition interventions: protocol for the One Nutrition Coverage Survey in Bangladesh
Source: BMJ Open. 2025 Dec 29;15(12):e099314. doi: 10.1136/bmjopen-2025-099314 (PMC12750752; doi:10.1136/bmjopen-2025-099314)
Supplement: Supplementary file 2 [file bmjopen-2025-099314.reviewer_comments.pdf]

PEER REVIEW HISTORY

BMJ Open publishes all reviews undertaken for accepted manuscripts. Reviewers are asked to complete a checklist review form and are provided with free text boxes to elaborate on their assessment. These free text comments are reproduced below.

ARTICLE DETAILS

Title (Provisional)

Methodological considerations and cost to measure coverage of multi-sectoral nutrition interventions: Protocol for the One Nutrition Coverage Survey in Bangladesh

Authors

Manohar, Swetha; Nguyen, Phuong Hong; Neupane, Sumanta; Munos, Melinda Kay; Heidkamp, Rebecca; Banerjee, Archis; Pandya, Niharika; Kim, Sunny S.

VERSION 1 - REVIEW

**Reviewer**	**1**
**Name**	**Sharn, Amy**
**Affiliation**	**Abbott Nutrition, Global Medical Affairs and Research**
**Date**	**10-Mar-2025**
**COI**	**I am an employee of Abbott Laboratories.**

Thank you for submitting your manuscript to BMJ Open. Within this PDF I've included some feedback that I believe to be helpful to improve your manuscript. Overall, the manuscript is well written and provides an opportunity to better understand the reach of current nutrition programs.

Overall

- I believe 'coverage' may be better stated as 'reach' of the RE-AIM Framework (Reach, Effectiveness, Adoption, Implementation, Maintenance) defined as "the absolute number, proportion, and representativeness of individuals who participate in a given program" from my understanding of this manuscript. If not, please provide justification as to why 'coverage' fits better.

Abstract/Key Questions

- How is the term 'essential nutrition intervention' defined? Is this being used interchangeably with 'multi-sectoral interventions'? As written, this is unclear why these are essential methodologies or how you define in the context of your work and objectives outside of the paper.

Methods

- **Key Nutrition Interventions Across the Life Course**
    - The sentence with the mention of the WHO needs a citation.

    - Acronyms such as WHO and WASH need to be defined/spelled out at their first appearance.
- **Landscaping Nutrition Policies and Programs**
    - Table 2 should be renumbered Table 1 as this is the first table mentioned in the manuscript.
- **Survey Design**
    - How was the survey developed? Were any local scientists and/or community champions involved in its design?
    - Will this work include survey validation?
- **Survey Setting**
    - The sentence beginning with "Despite significant progress" should be rephrased to use patient-first language (experience/have stunting v 'stunted')
- **Household Selection**
    - Who will be doing the recruiting? Will any local community champions be involved?
- **Study Participants**
    - For females who may be both an adolescent and woman of reproductive age/may have children (eg 19), how will these individuals be categorized?
- **Data Collection**
    - Acronyms such as DHS, MICS, BIHS, NSA, LSFF should be defined/spelled out at first use.
    - Is the Diet Quality Questionnaire validated for use in a Bangladesh population/diet?
- **Data Quality Control and Data Management**
    - GPS should be defined/spelled out at its first appearance.
- **Costing Data**
    - Will any scales on usability or promotion of the survey be used to speak to the ease of use/likelihood to use the survey again?
    - Will staffing costs be included?
- **Data Analysis**
    - Will any validation work of the survey be done or any analyses for internal consistency (e.g., Cronbach's alpha)?
    - How will nutrient intake data be analyzed?

Figures/Tables

- **Table 1 – Multi-Sectoral Nutrition Interventions by Target Population, Based on Global and National Nutrition Polices and Programs**
    - What does the shading mean? This is unclear as formatted.
- **Table 3 – Summary of Data Source and Type of Analysis for Monetary and Non-Monetary Costs**
    - Will staff full-time equivalents or staff hours be included as part of monetary costs?

      The cost by various study phases is unclear what that is composed of.

**Reviewer**	**2**
**Name**	**Iizuka, Katsumi**
**Affiliation**	**Fujita Health University Hospital, Clinical Nutrition**
**Date**	**03-Aug-2025**
**COI**	**None**

The protocol is clear in both its background and objectives. However, the methods section is difficult to understand. Wouldn't it be better to describe the statistical methods and survey questions a bit more clearly?

VERSION 1 - AUTHOR RESPONSE

REVIEWER 1

- Thank you for submitting your manuscript to BMJ Open. Within this PDF I've included some feedback that I believe to be helpful to improve your manuscript. Overall, the manuscript is well written and provides an opportunity to better understand the reach of current nutrition programs.

RESPONSE: Thank you for the positive assessment of our paper. We have addressed each of the reviewer's comments in detail below.

- I believe 'n' may be better stated as 'reach' of the RE-AIM Framework (Reach, Effectiveness, Adoption, Implementation, Maintenance) defined as "the absolute number, proportion, and representativeness of individuals who participate in a given program" from my understanding of this manuscript. If not, please provide justification as to why 'coverage' fits better.

RESPONSE: We acknowledge that "reach" and "coverage" are often used interchangeably. While reach refers to the *absolute number* of people who participate in or are affected by an intervention, coverage denotes the *proportion* of the target population that receives it among those who need it (Line 61-62). The definition of coverage used in our paper aligns with prevailing usage in global health and nutrition research and practice - for example, by the UN Sustainable Development Goals; WHO-UNICEF Technical Expert Advisory Groups on Nutrition Monitoring (TEAM), on Mother and Newborn Information for Tracking Outcomes and Results (MONITOR), and on Child Health Accountability Tracking (CHAT); WHO's Global Strategy for Women, Children's and Adolescent Health; Countdown to 2030; and the Improving Coverage Measurement (ICM) Core Group, and the IMPROVE project — as well as with definitions in Munos et al. (2022) and Marsh et al. (2020), which build on an established literature on coverage measurement. We note that the RE-AIM framework uses a broader definition of "reach," collapsing both absolute numbers and proportions under that term. However, our work focuses on coverage as defined above.

<u>Reference:</u>

Munos, M, Maïga A, Sawadogo-Lewis T, Wilson E, Ako O, Mkuwa S, Ngalesoni F, Brenner JL, Matovelo D, Ouili I, Soura A, Bougma M, Sheffel A, Hobbs AJ, Walker N. The RADAR coverage tool: developing a toolkit for rigorous household surveys for reproductive, maternal, newborn, and child health & nutrition indicators. Glob Health Action. 2022 Jun 30;15(sup1):2006419. doi: 10.1080/16549716.2021.2006419.

Marsh AD, Muzigaba M, Diaz T, Requejo J, Jackson D, Chou D, Cresswell JA, Guthold R, Moran AC, Strong KL, Banerjee A, Soucat A; Effective Coverage Think Tank Group. Effective coverage measurement in maternal, newborn, child, and adolescent health and nutrition: progress, future prospects, and implications for quality health systems. Lancet Glob Health. 2020 May;8(5):e730-e736. doi: 10.1016/S2214-109X(20)30104-2.

- How is the term 'essential nutrition intervention' defined? Is this being used interchangeably with 'multi-sectoral interventions'? As written, this is unclear why these are essential methodologies or how you define in the context of your work and objectives outside of the paper.

RESPONSE: Essential nutrition interventions are defined by the World Health Organization as evidence-based interventions that if implemented at scale could "reduce infant and child mortality, improve physical and mental growth and development and improve productivity" (WHO 2013). They used a life-course approach and are primarily focused on the preconception to first two years of life given that this is critical and nutritionally vulnerable phase of life. The interventions recommended in our study span sectors from health, agriculture, education, social protection and thereby we characterize them as multi-sectoral nutrition interventions. Changes have been made to Lines 110-117, 274 to offer more clarity on the underpinnings of these interventions.

Reference: World Health Organization. Essential nutrition actions: mainstreaming nutrition through the life-course [Internet]. Geneva: World Health Organization; 2013. 199 p. Available from: https://iris.who.int/handle/10665/326261

- Methods: Key Nutrition Interventions Across the Life Course. The sentence with the mention of the WHO needs a citation.

RESPONSE: The reference has been added to Line 110.

- Acronyms such as WHO and WASH need to be defined/spelled out at their first appearance.

RESPONSE: WHO and WASH have been spelled out in Lines 110 and 116, respectively, when they are used for the first time. All acronyms have been spelled out at their first appearance.

- Landscaping Nutrition Policies and Programs: Table 2 should be renumbered Table 1 as this is the first table mentioned in the manuscript.

RESPONSE: Thank you for catching this typo. This has been changed on Line 135.

- Survey Design: How was the survey developed? Were any local scientists and/or community champions involved in its design?

RESPONSE: The survey was designed with inputs from the Bangladesh Bureau of Statistics, other local researchers, and representatives of several UN and civil society groups that are listed under the acknowledgement section, Lines 640-648. Further, we have added a sentence to the 'Survey design' section, Lines 184-185, to clarify this in the manuscript text.

- Will this work include survey validation?

RESPONSE: This work incorporates new (nutrition-sensitive social protection (NSSP)) and improved (large-scale food fortification (LSFF)), maternal micronutrient supplementation) measures that have gone through face and construct validation; work that was undertaken by the DataDENT project (Lines 294-304). Manuscripts for this work are under preparation or review. Additionally, the survey questionnaires and other instruments will undergo through expert review and pre-testing to ensure relevance and clarity of questions and procedures. While estimates resulting from this survey will be compared to similar existing survey data, this survey does not include validation of accuracy. Lines 302-304 have been expanded to reflect this.

- Survey Setting: The sentence beginning with “Despite significant progress” should be rephrased to use patient-first language (experience/have stunting v ‘stunted’)

RESPONSE: Line 191- language has been updated.

- Household Selection: Who will be doing the recruiting? Will any local community champions be involved?

RESPONSE: Recruitment will be undertaken by the survey field team – enumerators led by the field supervisor (Line 233-234), and local community leader will be consulted to ensure sensitization of the community about the survey and securement of appropriate protocol (Lines 229-230).

- Study Participants: For females who may be both an adolescent and woman of reproductive age/may have children (eg 19), how will these individuals be categorized?

RESPONSE: Unmarried females (10-19 years) will be considered adolescents, and females 15-49 years of age will be considered women of reproductive age accounting for the fact they are likely to bear children in the years after marriage. Thus, adolescents 15-19 years fall into both categories and will be asked the relevant questions for both target populations. Line 241 has been updated to reflect this.

- Data Collection: Acronyms such as DHS, MICS, BIHS, NSA, LSFF should be defined/spelled out at first use.

RESPONSE: BIHS has been spelled out - see lines 294-295. All other acronyms have been spelled out at their first appearance. For DHS and MICS– see lines 72-73; for NSA and LSFF – see line 81-82.

- Is the Diet Quality Questionnaire validated for use in a Bangladesh population/diet?

RESPONSE: Yes, this tool has been adapted for used in Bangladesh. The Diet Quality Questionnaire (DQQ) has been shown to have cross-cultural validity and was specifically designed to be a low cost tool for use in population-based surveys. It is now used across countries in 83 Demographic and Health Surveys and in the Gallup World Poll to measure diet quality. These details have been included in the manuscript, Lines 329-330.

References:
Herforth AW et al. Development of the Diet Quality Questionnaire for Measurement of Dietary Diversity and Other Diet Quality Indicators. Curr Dev Nutr. 2024 Jun 21;8(8):103798. doi: 10.1016/j.cdnut.2024.103798).

Herforth AW, et al. Adaptation of the Diet Quality Questionnaire as a Global Public Good for Use in 140 Countries. Curr Dev Nutr. 2024 Nov 2;9(Suppl 1):104499. doi: 10.1016/j.cdnut.2024.104499).

- Data Quality Control and Data Management: GPS should be defined/spelled out at its first appearance.

RESPONSE: GPS has been spelled out at its first appearance - see line 360-361.

- Costing Data: Will any scales on usability or promotion of the survey be used to speak to the ease of use/likelihood to use the survey again?

RESPONSE: No specific scales on usability or promotion will be used. However, as outlined in the data analysis section (lines 394-396), questions asked to respondents about survey fatigue and estimated interview times (time per module and for the entire survey) will be computed, and these results will provide information on ease of use/survey burden and feasibility of survey implementation.

- Costing Data: Will staffing costs be included?

RESPONSE: Yes, staffing costs will be included. We have included some of the itemized monetary costs that will be included in Lines 420 - 421.

- Will any validation work of the survey be done or any analyses for internal consistency (e.g., Cronbach's alpha)?

RESPONSE: Where relevant and possible, analyses for internal consistency will be conducted Where relevant and possible, analyses for internal consistency will be conducted (for example, agreement between male household head versus WRA reporting of receipt of social protection transfers, estimates of IFA adherence in pregnancy when measured among currently pregnant women vs. those with a recent live birth). But this will not be possible for all indicators because the survey will not include multiple questions of the same concepts.

- How will nutrient intake data be analyzed?

RESPONSE: Since this is a coverage survey of multisectoral interventions, data on nutrient intake was not collected. The Diet Quality Questionnaire only was added because it is a short module validated for use in multiple low-and-middle-income country settings, including Bangladesh, and often included in multi-topic surveys to capture diet quality. Data on quantity of foods consumed are not collected by this tool, and therefore, no nutrient intake data will be analyzed.

- Figures/Tables: Table 1 – What does the shading mean? This is unclear as formatted.

RESPONSE: Turquoise-shaded cells indicate the target populations for each intervention (listed on the left) and which policies include those interventions. This explanation is now added as a note under the table.

- Table 3 – Summary of Data Source and Type of Analysis for Monetary and Non-Monetary Costs: Will staff full-time equivalents or staff hours be included as part of monetary costs?

RESPONSE: Monetary costs will include salaries (based on hours of work), per diem, logistics, travel, equipment, supplies, and consulting fees. Details are included in Table 3.

- The cost by various study phases is unclear what that is composed of.

RESPONSE: Details related to cost have been added in Table 3.

REVIEWER 2

- The protocol is clear in both its background and objectives. However, the methods section is difficult to understand. Wouldn’t it be better to describe the statistical methods and survey questions a bit more clearly?

RESPONSE: Thank you for your review of our protocol. We refer you to Lines 215-271 (sampling methods and sample size), data analysis (Lines 399-444) and, Table 2 and Figure 2 for an expanded overview of the statistical methods. Additionally, we have added the draft survey questionnaires into the Supplemental material (Supplemental material I) so that readers can have a more detailed understanding of what the survey will measure.
